# Supplementary material for: Targeting CD301 + macrophages inhibits endometrial fibrosis and improves pregnancy outcome
Source: EMBO Mol Med. 2023 Jul 31;15(9):e17601. doi: 10.15252/emmm.202317601 (PMC10493587; doi:10.15252/emmm.202317601)
Supplement: Supplementary file 1 — Appendix [file EMMM-15-e17601-s009.pdf]

APPENDIX

Targeting CD301+ macrophages inhibits endometrial fibrosis and improves pregnancy outcome

Haining Lv[1,2†], Haixiang Sun[2†], Limin Wang[1†], Simin Yao[1], Dan Liu[1], Xiwen Zhang[1], Zhongrui Pei[3], Jianjun Zhou[2], Huiyan Wang[1], Jianwu Dai[4], Guijun Yan[2], Lijun Ding[2], Zhiyin Wang[1], Chenrui Cao[1], Guangfeng Zhao[1,5*], Yali Hu[1,5,6*]

Table of contents

Appendix Figure S1 2
Appendix Figure S2 3
Appendix Figure S3 5
Appendix Figure S4 6
Appendix Figure S5 7
Appendix Figure S6 8
Appendix Figure S7 9
Appendix Figure S8 10
Appendix Table S1 11
Appendix Table S2 12
Appendix Table S3 12
Appendix Table S4 13
Appendix Table S5 13
Appendix Table S6 14

Appendix Figure S1

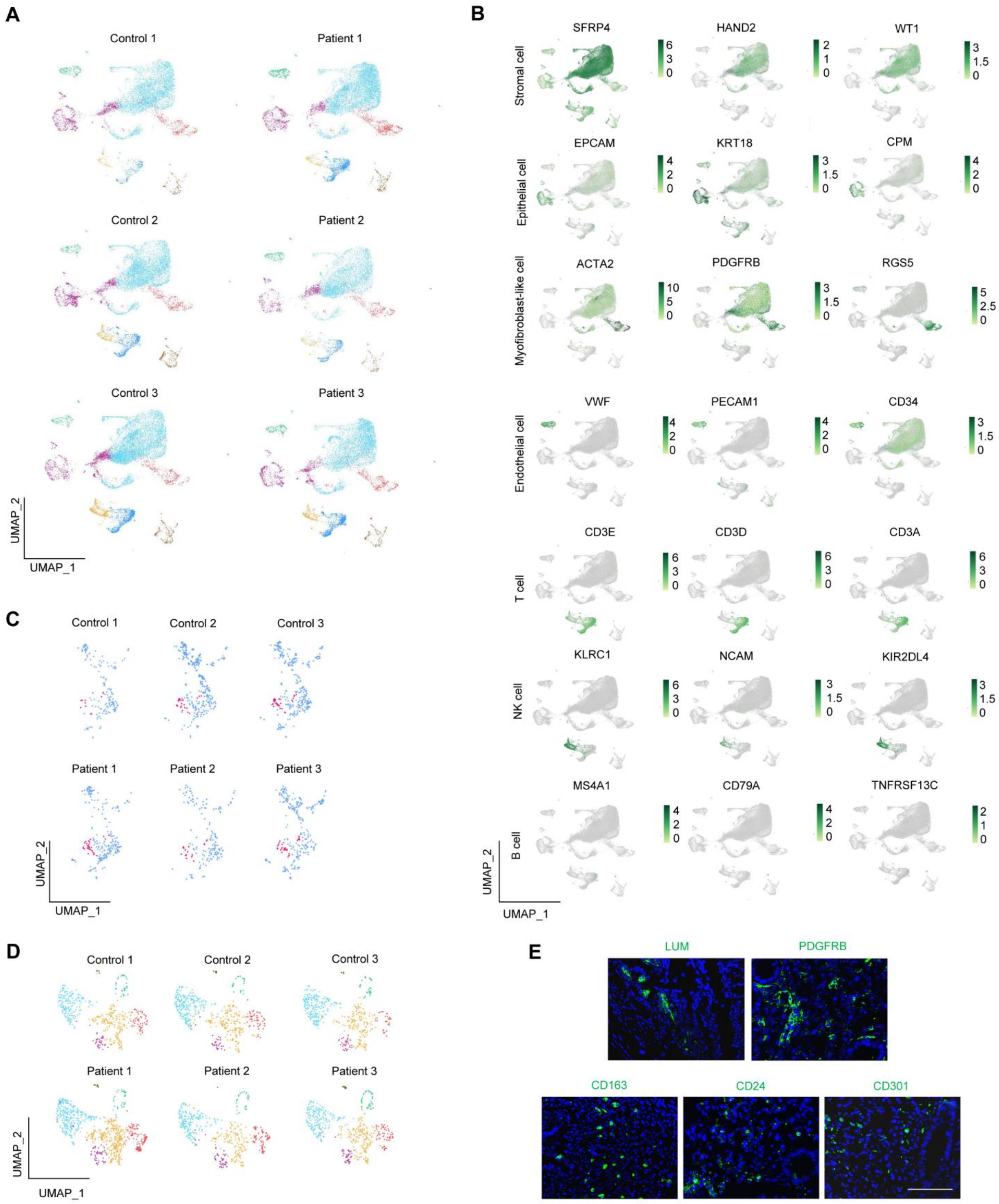

Appendix Figure S1 Endometrial cells atlas.

A. The distribution of each type of cells by UMAP in the controls (n=3 samples) and IUA patients (n=3 samples).
B. The distribution of marker genes for each type of cells (n=6 samples).
C. The distribution of dendritic cells and macrophages by UMAP from controls (n=3 samples) and IUA patients (n=3 samples).
D. The distribution of subtypes of myofibroblast-like cells by UMAP in controls (n=3 samples) and IUA patients (n=3 samples).
E. Localization of LUM, PDGFRB, CD163, and CD24 in endometrium of normal controls and CD301 in endometrium of IUA patients examined by immunofluorescence. Scale bar: 100μm.

Appendix Figure S2

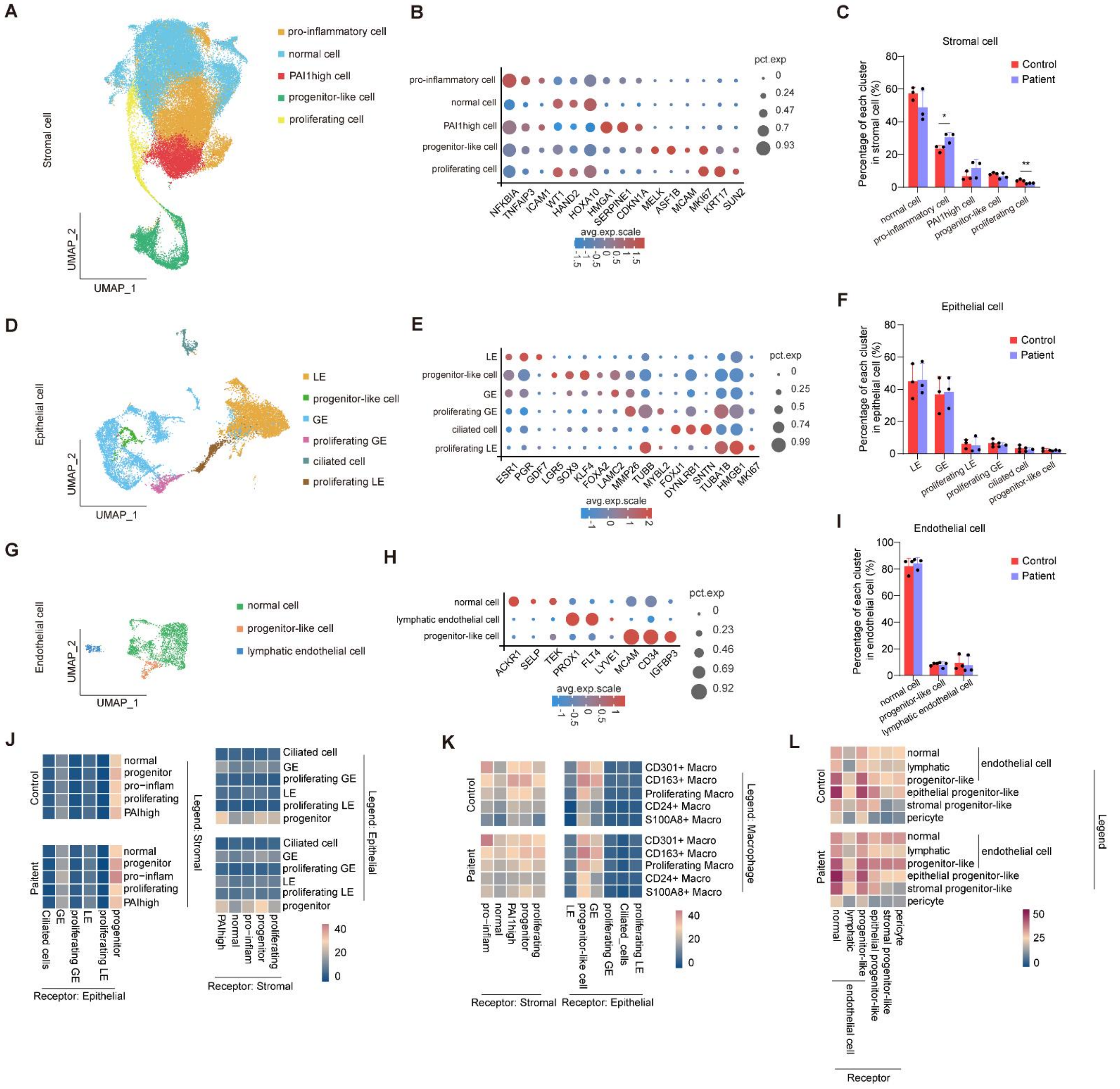

Appendix Figure S2 Characteristics of subpopulations of stromal cells, epithelial cells and endothelial cells.

A. The UMAP plot embedding subpopulations of stromal cells annotated by cell type/state from controls and patients with IUA (n=6 samples).

B. Bubble diagram showing the expression of marker genes for each cell subtype of stromal cells (n=6 samples).

C. The percentage of each cell subtype in total stromal cells between controls and patients with IUA (n=3 samples for each group).

D. The UMAP plot embedding subpopulations of epithelial cells annotated by cell type/state from controls and patients with IUA (n=6 samples).

E. Bubble diagram showing the expression of marker genes for each cell subtype of epithelial cells (n=6 samples).

F. The percentage of each cell subtype in total epithelial cells between controls and patients with IUA (n=3 samples for each group).

G. The UMAP plot embedding subpopulations of endothelial cells annotated by cell type/state from controls

and patients with IUA (n=6 samples).

H. Bubble diagram showing the expression of marker genes for each cell subtype of endothelial cells (n=6 samples).

I. The percentage of each cell subtype in total endothelial cells between controls and patients with IUA (n=3 samples for each group).

J. The abundance of connection between subpopulation of stromal cells and epithelial cells utilizing CellPhoneDB in controls and patients with IUA (n=3 samples for each group).

K. The abundance of connection between subpopulation of macrophages (as legends' cells) and subpopulation of stromal cells or epithelial cells (as receptors' cells) utilizing CellPhoneDB in controls and patients with IUA (n=3 samples for each group).

L. The abundance of connection among subpopulation of endothelial cells, epithelial progenitor-like cells, stromal progenitor-like cells, and pericytes utilizing CellPhoneDB in controls and patients with IUA (n=3 samples for each group).

Data are presented as mean ± SEM. (C, F and I) Two-tailed Student's t-test. *, P<0.05; **, P<0.01.

Appendix Figure S3

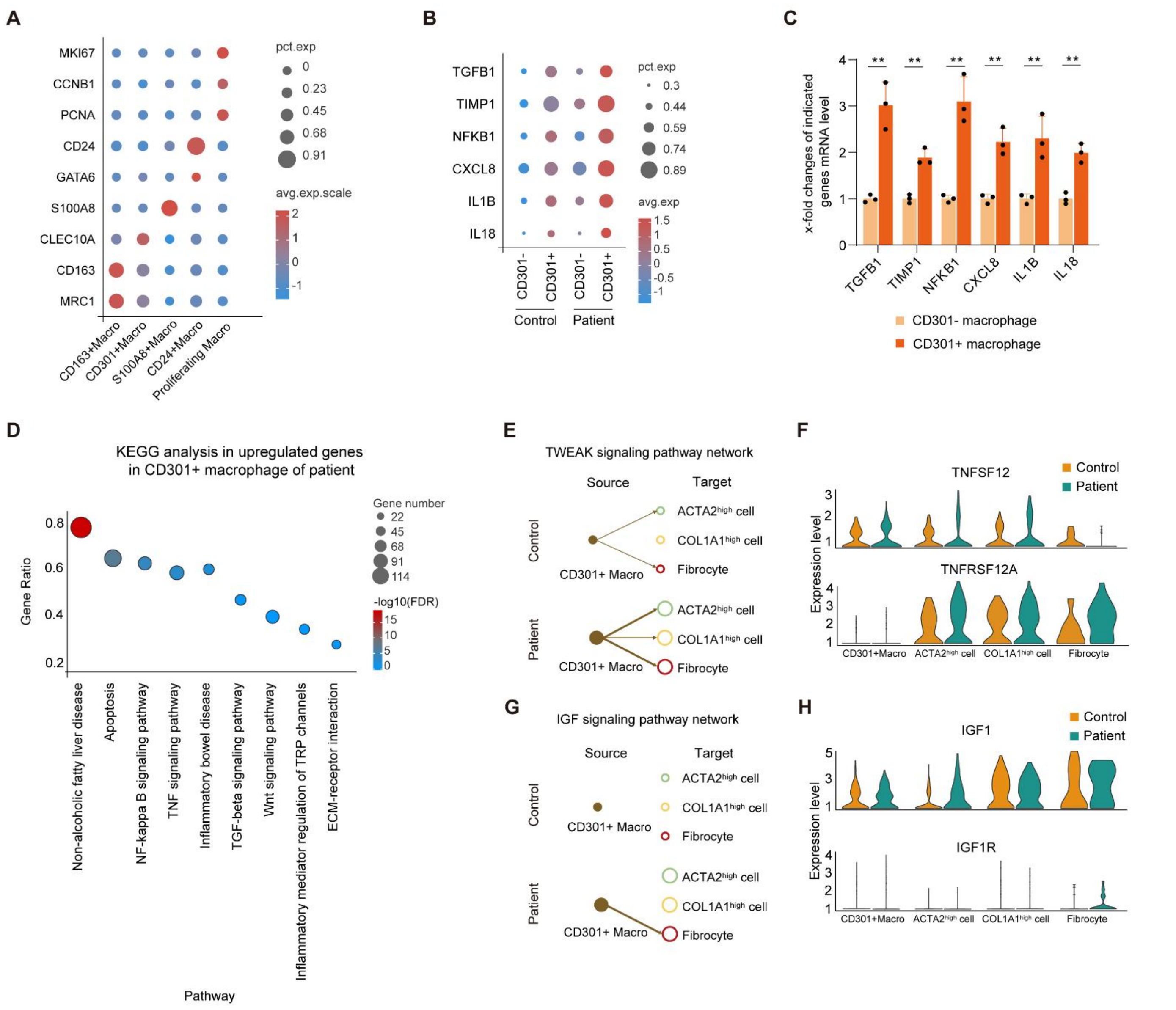

Appendix Figure S3 Characteristics of macrophages and the communication between CD301+ macrophages and myofibroblast-like cells

A. Bubble diagram showing the expression of marker genes for each subpopulation of macrophages (n=6 samples).
B. Bubble diagram showing the genes related to inflammation which are significantly high-expressed in CD301+ macrophages (n=3 samples for each group).
C. qRT-PCR analysis of indicated gene expression in sorted endometrial CD301- macrophages and CD301+ macrophages (n=3 biological replicates for each group).
D. KEGG analysis in significantly upregulated genes in CD301+ macrophages of patients compared to controls using scRNA-seq data (n=3 samples for each group).
E. Hierarchy diagram showing the interaction from CD301+ macrophages to pro-fibrotic subpopulations of myofibroblast-like cells in TWEAK signaling pathway network analyzed by CellChat (n=3 samples for each group). The size of circle diameter represents relative number of cells and the size of line represents relative strength of subpopulation interaction between controls and patients
F. Gene expression patterns of TWEAK signaling pathway in CD301+ macrophages, ACTA2^{high} cells, COL1A1^{high} cells and fibrocytes analyzed by CellChat (n=3 samples for each group).
G. Hierarchy diagram showing the interaction from CD301+ macrophages to pro-fibrotic subpopulations of myofibroblast-like cells in IGF signaling pathway network analyzed by CellChat (n=3 samples for each group). The size of circle diameter represents relative number of cells and the size of line represents relative strength of subpopulation interaction between controls and patients.

H. Gene expression patterns of IGF signaling pathway in CD301+ macrophages, ACTA2^{high} cells, COL1A1^{high} cells and fibrocytes analyzed by CellChat (n=3 samples for each group).

Data are presented as mean ± SEM. (C) Two-tailed Student's t-test. **, P<0.01.

Appendix Figure S4

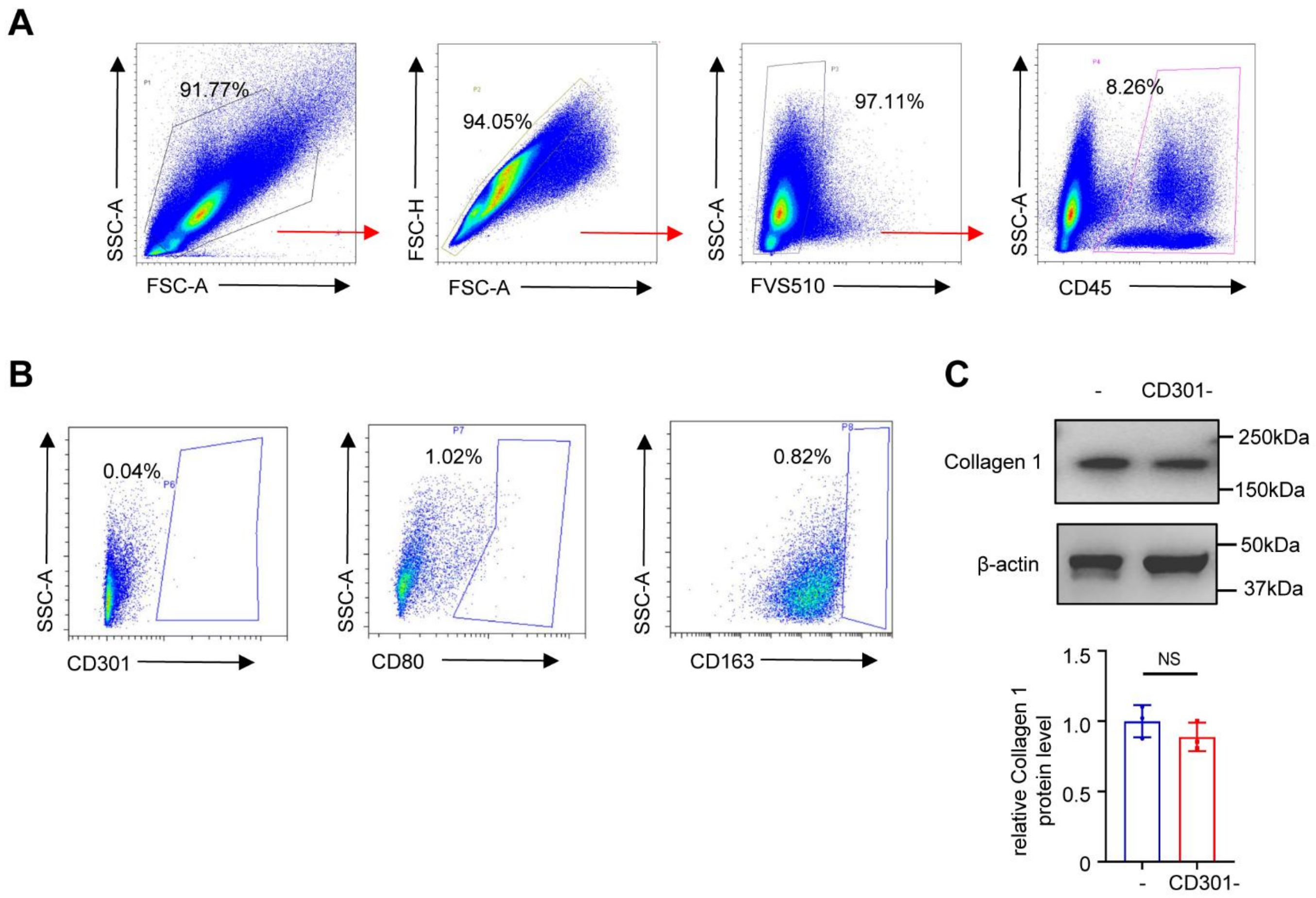

Appendix Figure S4 Flow cytometric analysis of human endometrial tissue and the effect of CD301- macrophages on stromal cells.

A. Flow cytometry gating strategy for FVS510-CD45+ cells.

B. Flow cytometric analysis of isotype of CD301, CD80 and CD163 expression in endometrium.

C. Western blot analysis of Collagen 1 expression in hESCs only and hESCs treated with the supernatant of CD301-macrophages for 48h (n=3 technical replicates for each group).

Data are presented as mean ± SEM. (C) Two-tailed Student's t-test. NS, not significant.

Appendix Figure S5

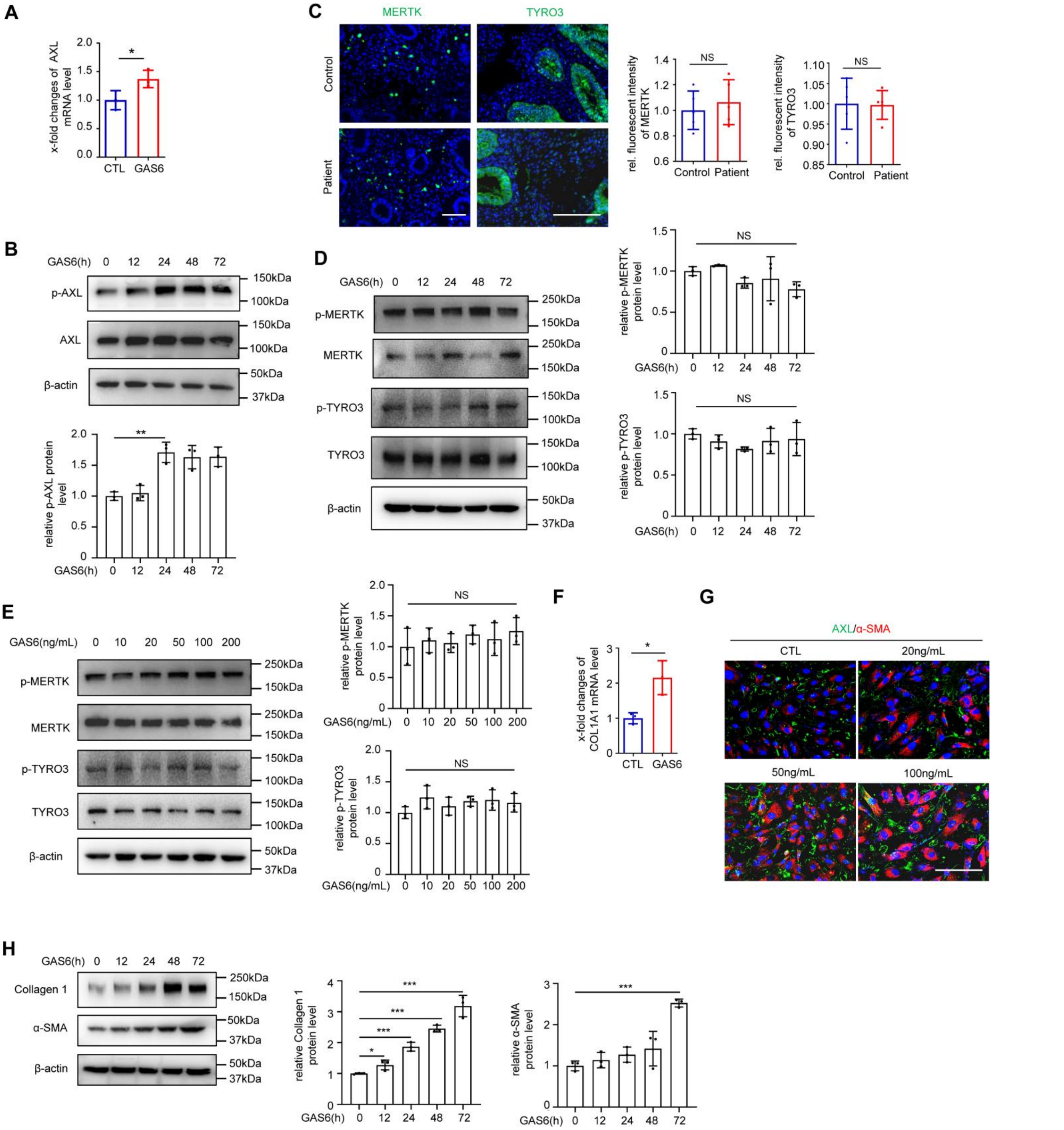

Appendix Figure S5. Expression of MERTK and TYRO3 in human endometrial tissue and cells.

A. qRT-PCR analysis of AXL expression in hESCs treated with or without GAS6 (50ng/mL) for 72h (n=3 biological replicates for each group).

B. Western blot analysis of phospho-AXL (p-AXL) and AXL in hESCs treated with GAS6 (50ng/mL) at time of 12, 24, 48 and 72h (n=3 technical replicates for each group).

C. Localization of MERTK and TYRO3 in endometrium of normal controls and patients with IUA (n=5 samples for each group). Scale bar: 100μm.

D. Western blot analysis of phosphorylated MERTK, TYRO3 (pMERTK, pTYRO3) and MERTK, TYRO3 in hESCs treated with GAS6 (50ng/mL) at time of 12, 24, 48 and 72h (n=3 technical replicates for each group).

E. Western blot analysis of phosphorylated MERTK, TYRO3 (pMERTK, pTYRO3) and MERTK, TYRO3 in hESCs treated with GAS6 at dose of 10, 20, 50, 100 and 200ng/mL (n=3 technical replicates for each

group).
F. The mRNA level of COL1A1 expression in hESCs after treatment with GAS6 (50ng/mL) for 72h analyzed by qRT-PCR (n=3 biological replicates for each group).
G. Localization of AXL and α-SMA in hESCs after treatment with GAS6 at dose of 20, 50 and 100ng/mL for 72h. Scale bar: 50μm.
H. Western blot analysis of α-SMA and Collagen 1 in hESCs treated with GAS6 (50ng/mL) at time of 12, 24, 48 and 72h (n=3 technical replicates for each group).

Data are presented as mean ± SEM. (A, C and F) Two-tailed Student's t-test. (B, D, E, H) One-way ANOVA with Tukey's post hoc analysis. *, P<0.05; **, P<0.01; ***, P<0.001. NS, not significant.

Appendix Figure S6

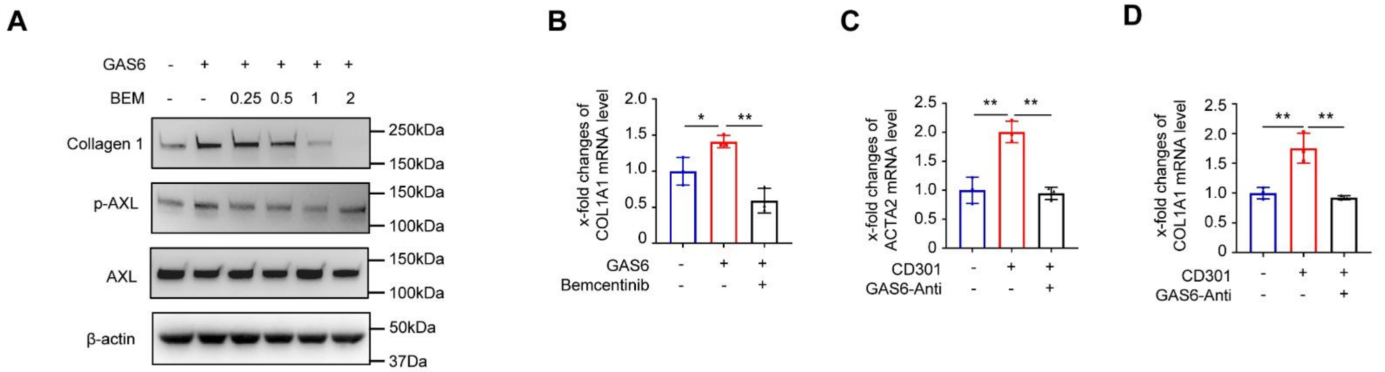

Appendix Figure S6. Expression of downstream genes under the treatment of Bemcentinib or GAS6 antibody.

A. Western blot analysis of Collagen 1, phosphorylated AXL and AXL in hESCs treated with Bemcentinib at the dose of 0, 0.25, 0.5, 1, 2μM for 1 hour, followed by GAS6 (50ng/mL) for 72h.
B. The mRNA level of COL1A1 expression in hESCs after treatment with Bemcentinib (1μM) for 1 hour, followed by GAS6 (50ng/mL) for 72h (n=3 biological replicates for each group).
C. qRT-PCR analysis of ACTA2 expression in hESCs treated with the supernatants of CD301+ and CD301- macrophages and GAS6 neutralizing antibody (2.5μg/mL) for 72h (n=3 biological replicates for each group).
D. qRT-PCR analysis of COL1A1 expression in hESCs treated with the supernatants of CD301+ and CD301- macrophages and GAS6 neutralizing antibody (2.5μg/mL) for 72h (n=3 biological replicates for each group).

Data are presented as mean ± SEM. (B-D) One-way ANOVA with Tukey's post hoc analysis. *, P<0.05; **, P<0.01.

Appendix Figure S7

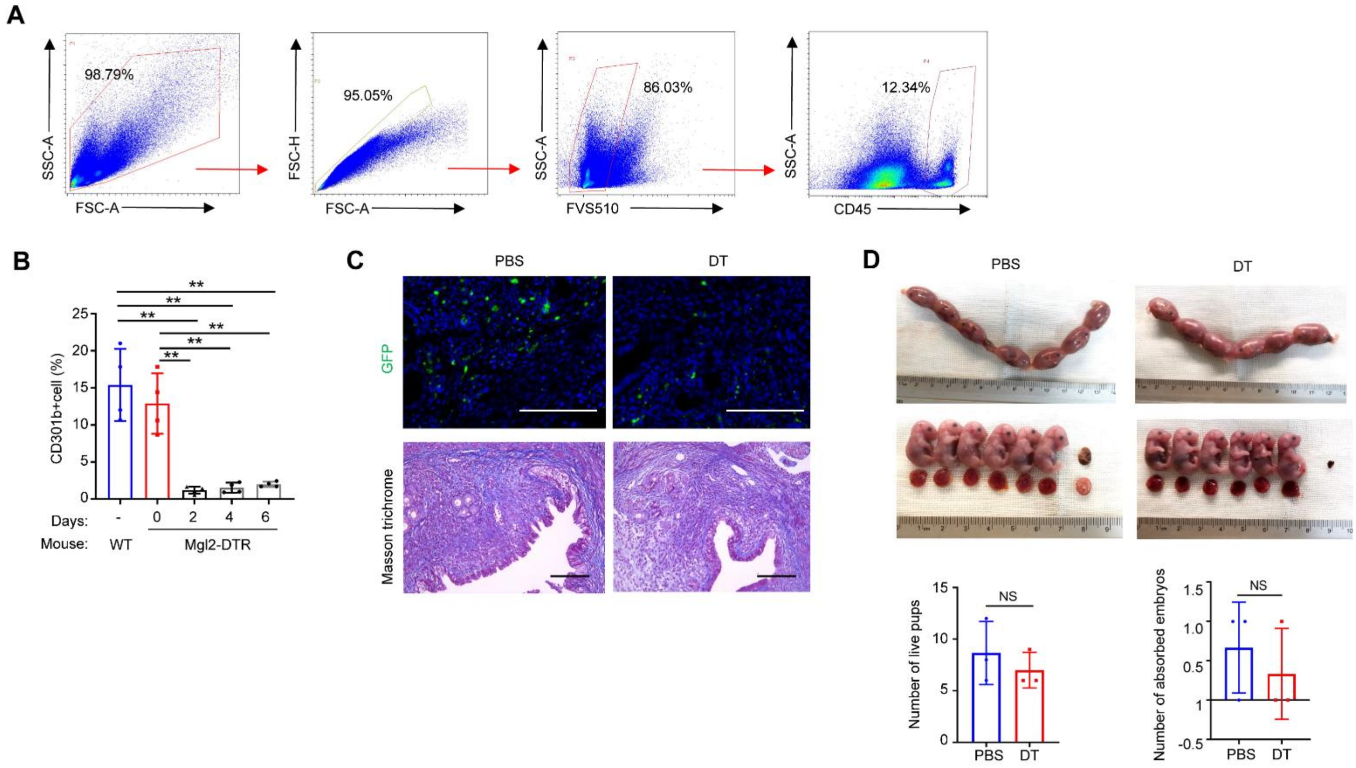

Appendix Figure S7. Effects of depletion of CD301b+ macrophages on the fibrotic phenotype and the number of live pups and absorbed embryos in mice.

A. Flow cytometry gating strategy for FVS510-CD45+ cells of murine uterine cells.
B. Flow cytometric analysis of proportion of CD301b+ cells for 0, 2, 4, 6 days after single injection of DT in the uterus of Mgl2-DTR and Wild-type mice (n=4 mice for each group).
C. Representative images of GFP-Mgl2 expression and Masson trichrome staining in endometrium of Mgl2-DTR mice injected by DT or PBS four days later (n=3 mice for each group). Scale bar, 100μm.
D. The number of live pups and absorbed embryos at 18.5 dpc in mice of PBS and DT group (n=3 mice for each group).

Data are presented as mean ± SEM. (B) One-way ANOVA with Tukey's post hoc analysis. (D) Two-tailed Student's t-test. NS, not significant.

Appendix Figure S8

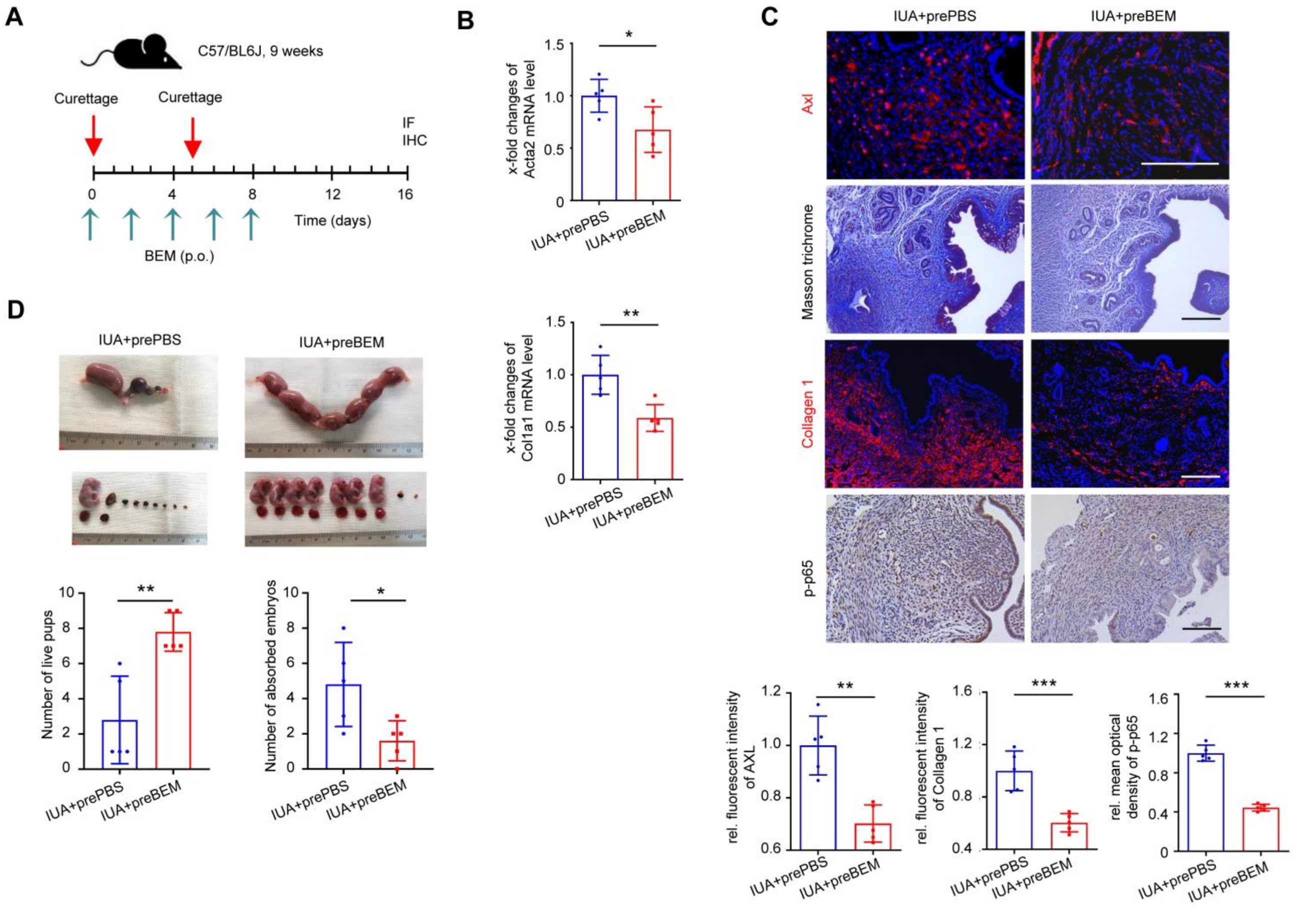

Appendix Figure S8. Preventive (pre) effects of Bemcentinib on endometrial fibrosis.

A. Outline of the design of Bemcentinib (BEM) administration during the model induction.

B. qRT-PCR analysis of Acta2 and Col1a1 expression in the uterus of IUA+prePBS and IUA+preBEM mice (n=5 mice for each group).

C. Representative images of immunostaining for AXL, Collagen 1, p-p65 and Masson trichrome staining in the endometrium of IUA+prePBS group and IUA+preBEM group (n=5 mice for each group).

D. The number of live pups and absorbed embryos at 18.5 dpc between IUA+prePBS group and IUA+preBEM group (n=5 mice for each group).

Scale bar: 100μm. Data are presented as mean ± SEM. (B-D) Two-tailed Student's t-test. *, P<0.05; **, P<0.01; ***, P<0.001.

Appendix Table S1 - Clinical characteristics of women included in scRNA-seq analysis [a)].

	Control-1	Control-2	Control-3	Patient-1	Patient-2	Patient-3
Age (year)	29	31	27	25	33	28
Times for D&C	0	0	0	2	5	3
Times for TCRA	0	0	0	3	3	1
Parity (time)	0	0	0	0	0	0
Length of menstrual cycle (day)	28	30	30	31	30	30
Day after menstruation (day)	13	13	14	14	14	14
Follicle size on the day of hysteroscopy (mm)	18*18*17	17*16*17	16*16*18	16*17*16	17*18*17	16*18*7
EM on the day of hysteroscopy (mm)	8.4	8.1	9.7	5.8	6.4	6.1
Serum progesterone (nmol/L)	0.34	0.14	0.21	0.21	0.31	0.35
AFS score	0	0	0	12	10	10

a) D&C, dilation and curettage; TCRA, transcervical resection of adhesion; EM, endometrial thickness; AFS: American Fertility Society.

Appendix Table S2 - Cell numbers of each type of cells in scRNA-seq analysis.

Cluster	Control-1	Control-2	Control-3	Patient-1	Patient-2	Patient-3
Stromal cell	5006	5911	7621	6984	4587	5766
Epithelial cell	1288	1498	1466	1208	935	1441
Myofibroblast-like cell	434	517	527	750	466	592
Endothelial cell	302	216	237	412	299	289
T cell	302	927	1127	1109	510	1343
Macrophages/DC	247	339	311	243	192	275
Natural killer cell	115	336	629	175	152	467
B cell	19	20	21	10	10	12
Mast cell	12	26	24	10	33	22
Total	7725	9790	11963	10901	7184	10207

Appendix Table S3 - Cell numbers of macrophage/DC in scRNA-seq analysis.

Cluster	Control-1	Control-2	Control-3	Patient-1	Patient-2	Patient-3
Macrophage	233	302	281	227	169	251
DC	14	37	30	16	23	24

Appendix Table S4 - Patient clinical information b).

Items	Control (n = 48)	Patient (n = 48)	*P* value
Age (year)	30.29 ±0.60	31.04 ± 0.77	> 0.05
Times for D&C	1.50 ± 0.24	2.96 ± 0.23	< 0.05
Times for TCRA	0	2.18 ± 0.21	-
Maximum endometrial thickness (mm)	10.23±0.38	6.49±0.33	< 0.05
Length of menstrual cycle (day)	30.21±0.23	30.59±0.53	> 0.05
Day after menstruation	12.18±0.41	13.04±0.41	> 0.05
Serum progesterone(day)	0.43±0.12	0.41±0.59	> 0.05
AFS score	0	10.21±0.26	< 0.05
Diagnosis	Tubal infertility	Intrauterine adhesion	

b) D&C, dilation and curettage; TCRA, transcervical resection of adhesion; AFS, American Fertility Society.

Appendix Table S5 - Primer sequences.

Gene name	Forward Primer (5'-3')	Reverse Primer (5'- 3')
18S rRNA	CTTTGGTCGCTCGCTCCTC	CTGACCGGGTTGGTTTTGAT
AXL	GTGGGCAACCCAGGGAATATC	GTACTGTCCCGTGTCGGAAAG
COL1A1	GAGGGCCAAGACGAAGACATC	CAGATCACGTCATCGCACAAC
TGFB1	CAAGCAGAGTACACACAGCAT	TGCTCCACTTTTAACTTGAGCC
TIMP1	ACCACCTTATACCAGCGTTATGA	GGTGTAGACGAACCGGATGTC
NFKB1	GAAGCACGAATGACAGAGGC	GCTTGGCGGATTAGCTCTTTT
CXCL8	ACTGAGAGTGATTGAGAGTGGAC	AACCCTCTGCACCCAGTTTTC
IL1B	ATGATGGCTTATTACAGTGGCAA	GTCGGAGATTCGTAGCTGGA
IL18	TCTTCATTGACCAAGGAAATCGG	TCCGGGGTGCATTATCTCTAC
ACTA2	CTATGAGGGCTATGCCTTGCC	GCTCAGCAGTAGTAACGAAGGA
Col1a1	CTGGCGGTTCAGGTCCAAT	TTCCAGGCAATCCACGAGC
Acta2	CCCAGACATCAGGGAGTAATGG	TCTATCGGATACTTCAGCGTCA

Appendix Table S6 - Antibodies used for immunohistochemistry (IHC), immunofluorescence (IF), western blot (WB), Flow cytometry (FC).

Antibody	Source	Identifier
Anti-mouse CD45 APC-Cy7	BD Biosciences	Cat# 557659, clone 30-F11; RRID: AB_396774
Anti-mouse CD11b FITC	BD Biosciences	Cat#557396, clone M1/70; RRID: AB_396679
Anti-mouse F4/80 Alexa Fluor 647	BD Biosciences	Cat#565853, clone T45-2342; RRID: AB_2744474
Anti-mouse CD301b PE	BioLegend	Cat#146803, clone URA-1; RRID: AB_2562943
Anti-mouse CD163 BV421	BioLegend	Cat#155309, clone S15049I; RRID: AB_2814063
Anti-mouse CD86 PE-Cy7	BD Biosciences	Cat#560582, clone GL1; RRID: AB_1727518
Rat IgG2a, κ PE	BioLegend	Cat#400507, clone RTK2758
FVS 510	BD Biosciences	Cat#564406; RRID: AB_2869572
Anti-human CD45 PE-Cy7	BioLegend	Cat#368532, clone 2D1; RRID: AB_2715892
Anti-human CD14 FTIC	BioLegend	Cat#301804, clone M5E2; RRID: AB_314186
Anti-human CD80 PE	BioLegend	Cat#305208, clone 2D10; RRID: AB_314504
Mouse IgG1, κ PE	BioLegend	Cat#400111, clone MOPC-21
Anti-human CD163 BV650	BD Biosciences	Cat#563888, clone GHI/61; RRID: AB_2738468
Mouse IgG1, κ BV650	BD Biosciences	Cat#563231, clone X40; RRID: AB_2869470
Anti-human CD301 APC	BioLegend	Cat#354706, clone H039G3; RRID: AB_11219389
Mouse IgG2a, κ APC	BioLegend	Cat#400221, clone MOPC-173; RRID: AB_2891178
Goat anti-GAS6	R&D Systems	Cat# AF885; RRID: AB_2108079
Goat anti-GAS6	R&D Systems	Cat# AB885; RRID: AB_354376
Goat anti-GAS6	R&D Systems	Cat# AF986; RRID: AB_2263130
Rabbit anti-CCL8	Abcam	Cat# ab9671; RRID: AB_308749
Rabbit anti-AXL	Cell Signaling Technology	Cat#8661, clone C89E7; RRID: AB_11217435
Rabbit anti-Phospho-AXL (Tyr779)	Cell Signaling Technology	Cat#96453
Mouse anti-AXL	Abcam	Cat# ab89224, clone MM0098-2N33; RRID: AB_2049189
Goat anti-AXL	R&D Systems	Cat# AF854; RRID: AB_355663
Mouse anti-CD14	Abcam	Cat# ab182032, clone 4B4F12
HRP-rabbit anti-β-actin	Abclonal	Cat# AC028; RRID: AB_2769861
Rabbit anti-MERTK	Abcam	Cat# ab52968, clone Y323; RRID: AB_2143584
Rabbit anti-Phospho-MERTK (Y749+Y753+Y754)	Abcam	Cat#ab14921; RRID: AB_2250636
Rabbit anti-TYRO3	Abcam	Cat# ab109231, clone EPR4308
Rabbit anti-Phospho-TYRO3 (Tyr681)	Biorbyt	Cat#orb186274
Mouse anti-CD68	Abcam	Cat# ab955, clone KP1; RRID: AB_307338
Rabbit anti-CD301	Novus	Cat# NBP1-84591; RRID: AB_11031743
Mouse anti-α-SMA	Abcam	Cat# ab7817, clone 1A4; RRID: AB_262054

Rabbit anti- α-SMA	Abcam	Cat# ab5694; RRID: AB_2223021
Mouse anti-Collagen 1	Abcam	Cat# ab6308, clone COL-1; RRID: AB_305411
Rabbit anti-Collagen 1	Proteintech	Cat# 14695-1-AP; RRID: AB_2082037
Rabbit anti-Phospho-NF-κB p65 (Ser 536)	Cell Signaling Technology	Cat#3033, clone 93H1; RRID: AB_331284
Rabbit anti- NF-κB p65	Cell Signaling Technology	Cat#8242, clone D14E12; RRID: AB_10859369
Rabbit anti-Phospho-NF-κB p65 (Ser 536)	Abcam	Cat#ab86299; RRID: AB_1925243
Goat anti-CD301a/b	R&D Systems	Cat#AF4297; RRID: AB_2248147
Rabbit anti-Fibronectin	Abcam	Cat#ab2413; RRID: AB_2262874
Rabbit anti-CTGF	Cell Signaling Technology	Cat#86641; RRID: AB_2800085
Rabbit anti-CD24	Abcam	Cat#ab278509
Rabbit anti-LUM	Abcam	Cat#ab168348; RRID: AB_2920864
Alexa Fluor 594 Donkey anti-rabbit	Abcam	Cat#ab150076; RRID: AB_2782993
Alexa Fluor 594 Donkey anti-mouse	Abcam	Cat#ab150108; RRID: AB_2732073
Alexa Fluor 488 Donkey anti-rabbit	Abcam	Cat# ab150073; RRID: AB_2636877
Alexa Fluor 488 Donkey anti-mouse	Abcam	Cat# ab150105; RRID: AB_2732856
Alexa Fluor 488 Donkey anti-goat	Abcam	Cat# ab150129; RRID: AB_2687506
Alexa Fluor 594 Donkey anti-goat	Abcam	Cat# ab150132; RRID: AB_2810222
Alexa Fluor 488 Goat anti-rat	Abcam	Cat# ab150157; RRID: AB_2722511
