# Supplementary figures and images for: Targeting CD301 + macrophages inhibits endometrial fibrosis and improves pregnancy outcome (part 1 of 2)
Source: EMBO Mol Med. 2023 Jul 31;15(9):e17601. doi: 10.15252/emmm.202317601 (PMC10493587; doi:10.15252/emmm.202317601)

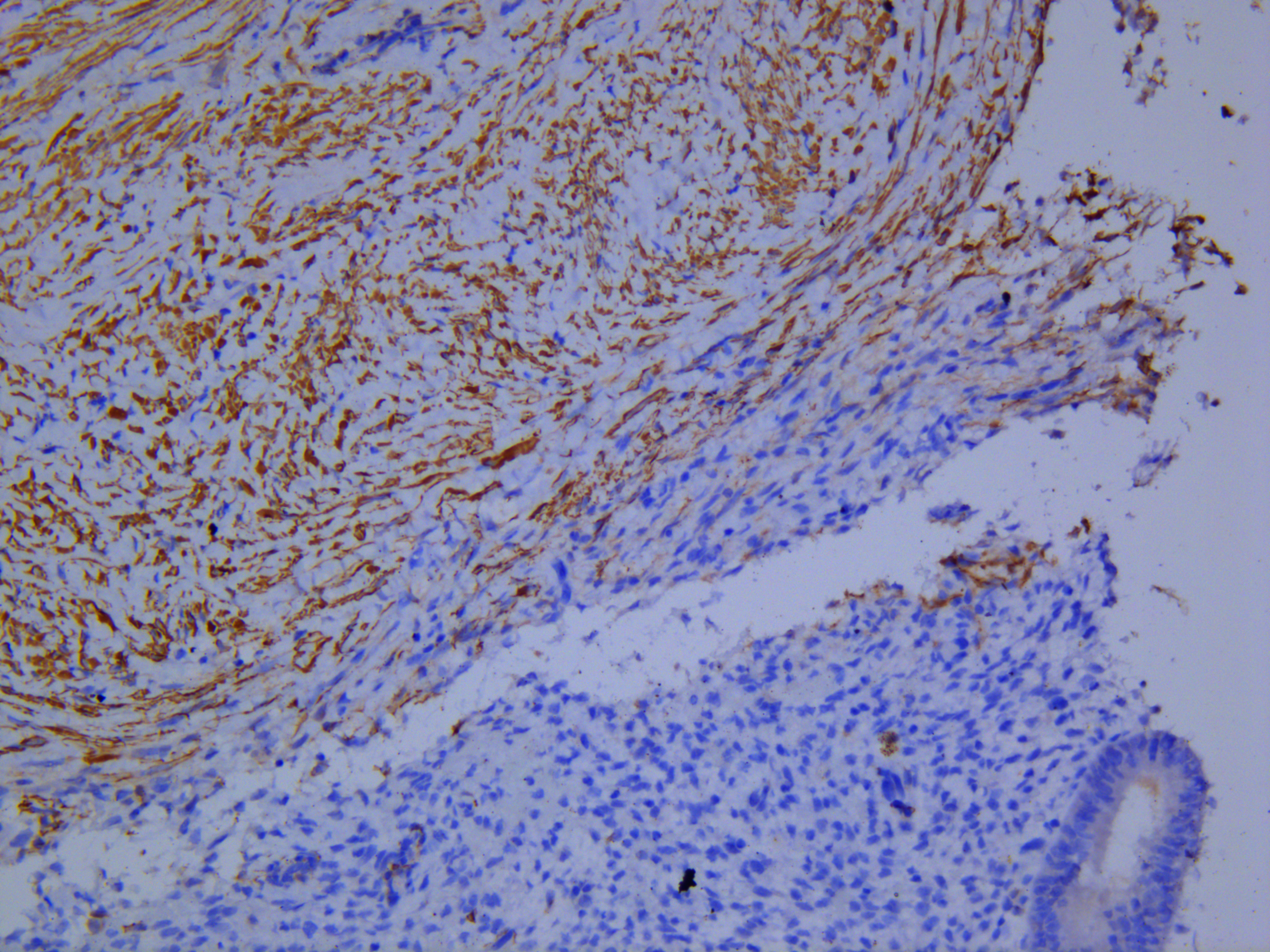

Supplement: Supplementary file 2 — Source Data for Figure 1 [file EMMM-15-e17601-s004.zip › Figure 1-2/1E/1E-a-SMA-Patient.tif]

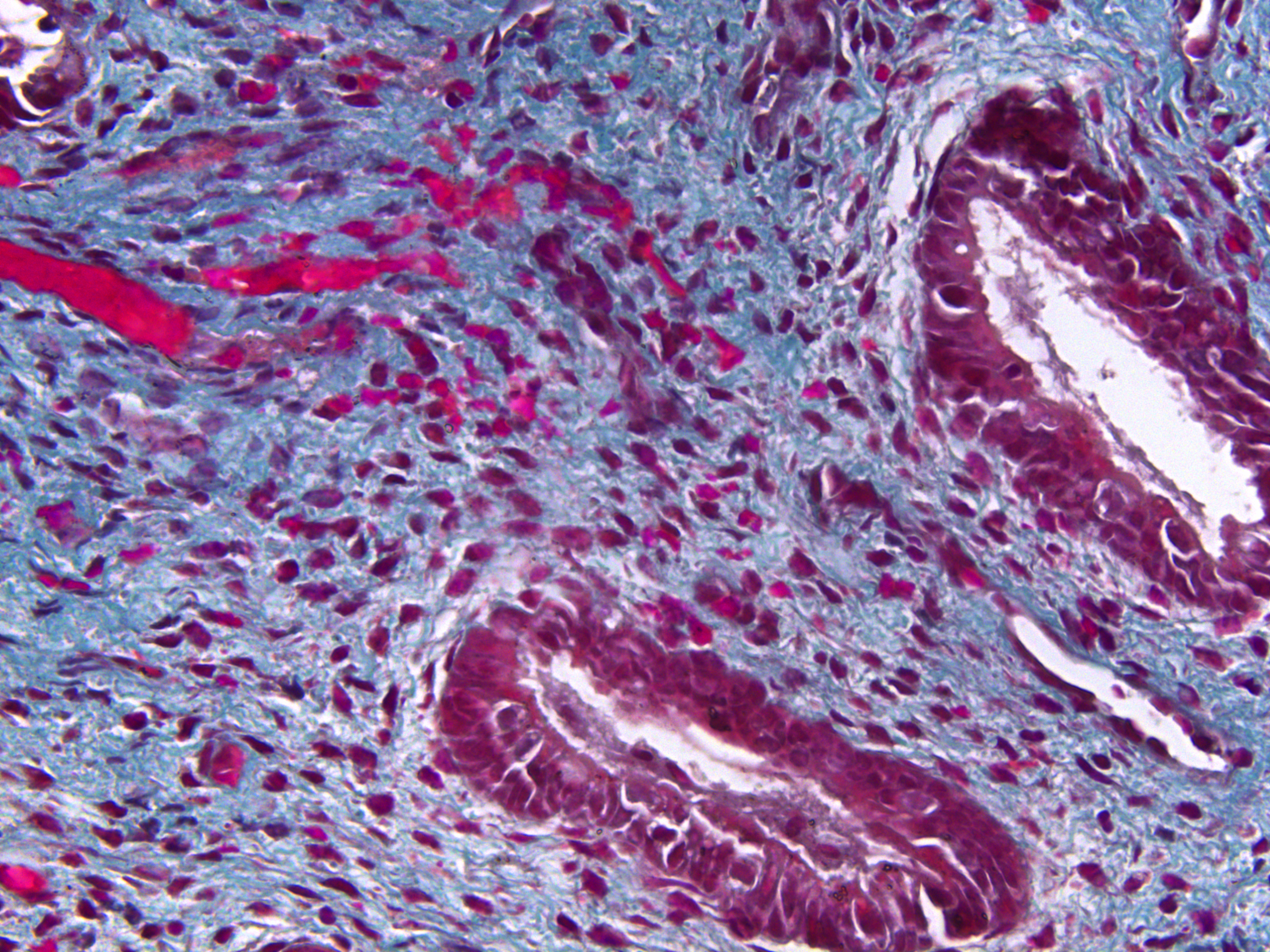

Supplement: Supplementary file 2 — Source Data for Figure 1 [file EMMM-15-e17601-s004.zip › Figure 1-2/1E/1E-Masson-Patient.tif]

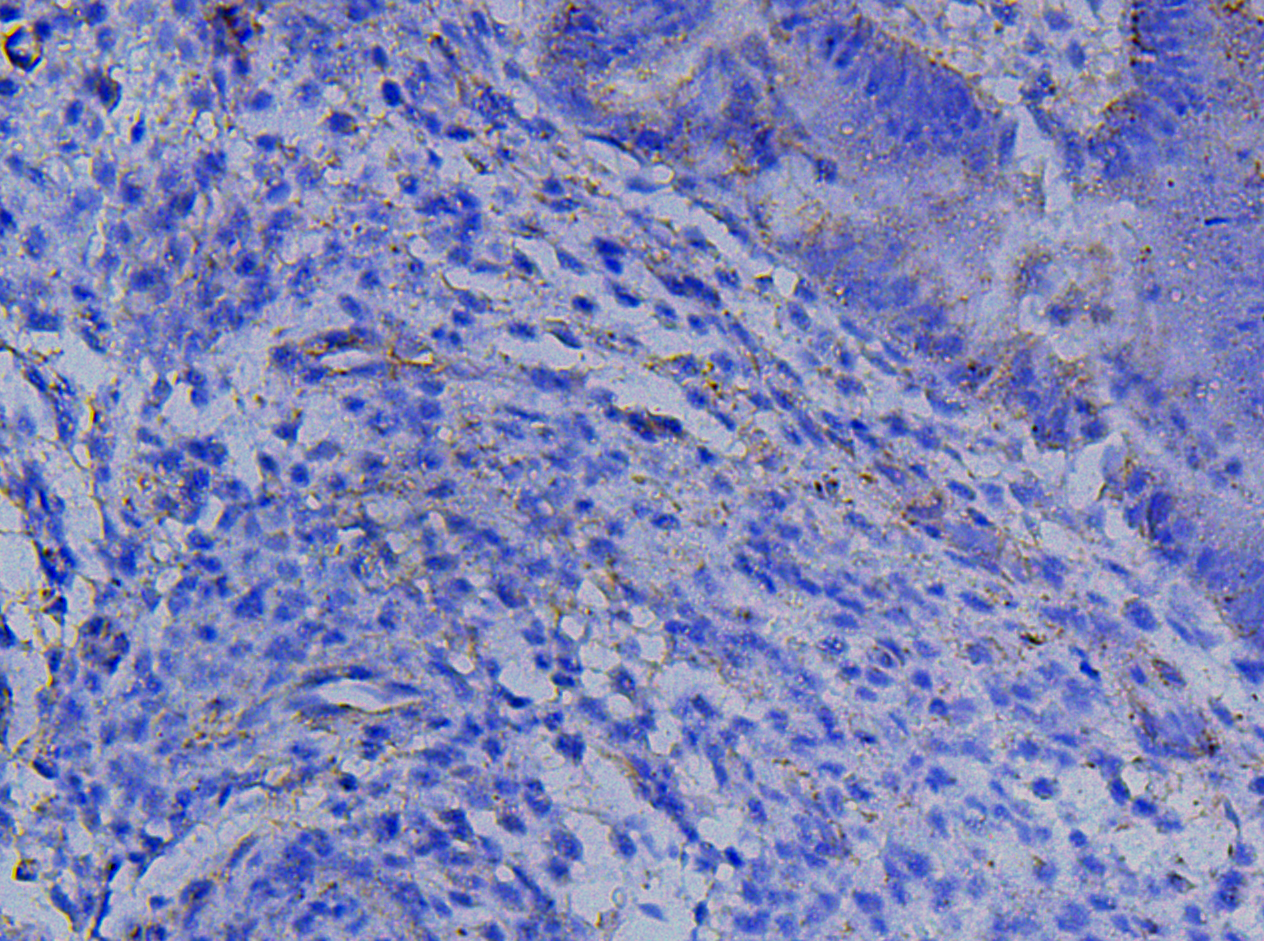

Supplement: Supplementary file 2 — Source Data for Figure 1 [file EMMM-15-e17601-s004.zip › Figure 1-2/1E/1E-Collagen1-Control.tif]

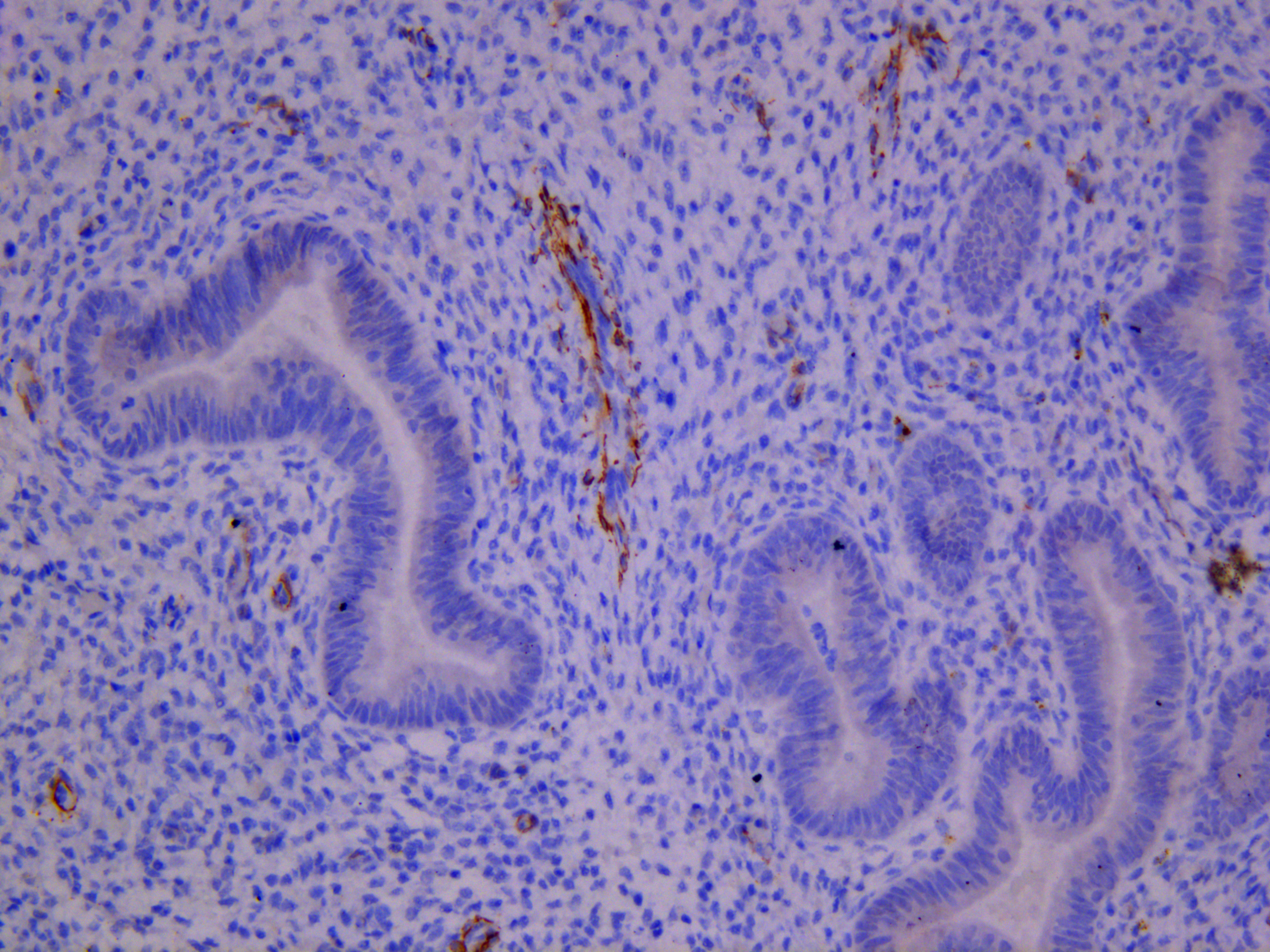

Supplement: Supplementary file 2 — Source Data for Figure 1 [file EMMM-15-e17601-s004.zip › Figure 1-2/1E/1E-a-SMA-Control.tif]

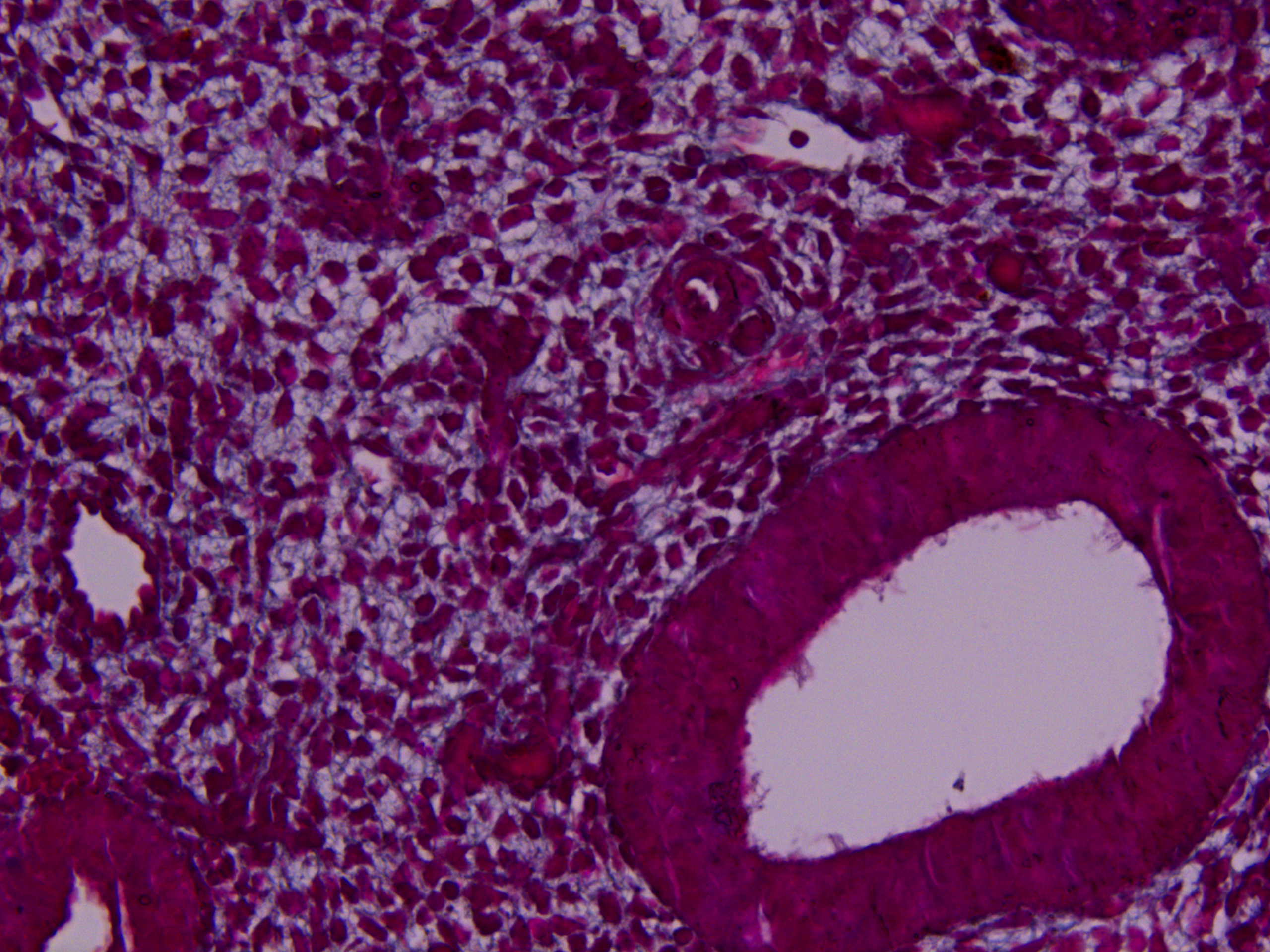

Supplement: Supplementary file 2 — Source Data for Figure 1 [file EMMM-15-e17601-s004.zip › Figure 1-2/1E/1E-Masson-Control.tif]

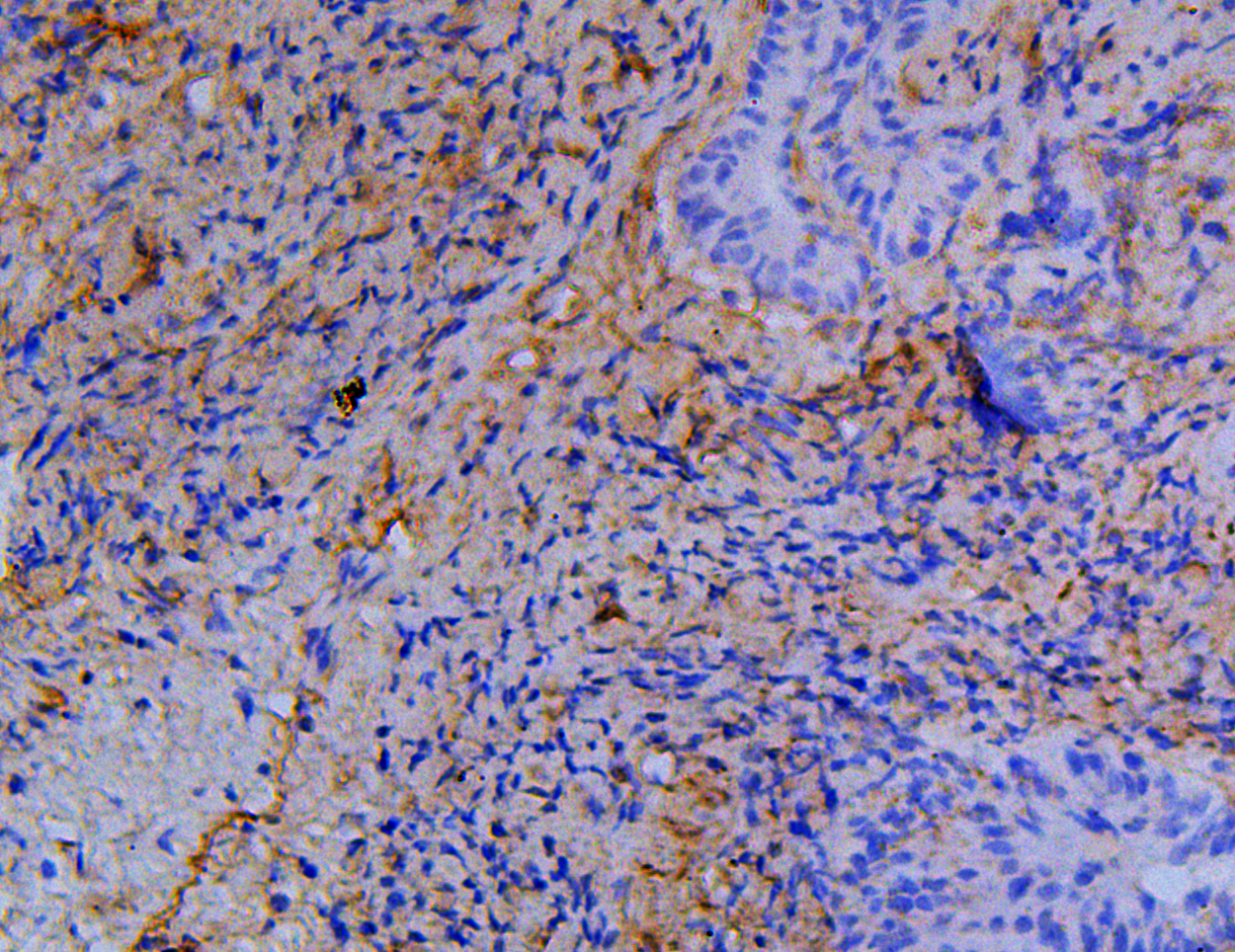

Supplement: Supplementary file 2 — Source Data for Figure 1 [file EMMM-15-e17601-s004.zip › Figure 1-2/1E/1E-Collagen1-Patient.tif]

**Fibronectin**

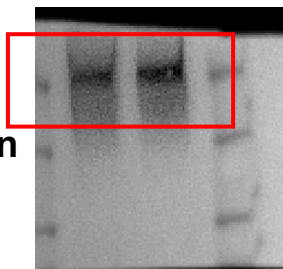

**Collagen 1**

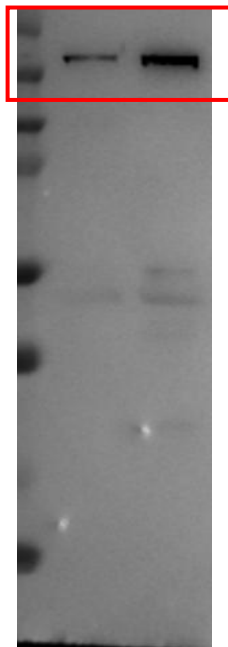

**$\alpha$ -SMA**

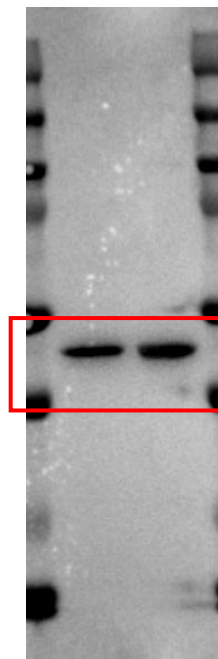

**CTGF**

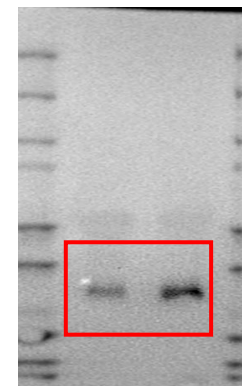

**$\beta$ -actin**

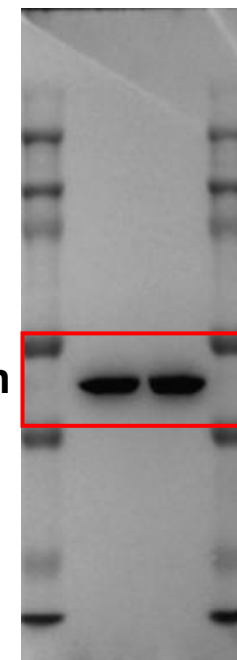

Supplement: Supplementary file 3 — Source Data for Figure 2 [file EMMM-15-e17601-s003.zip › Figure 2-2/2G/2G blot.pdf]

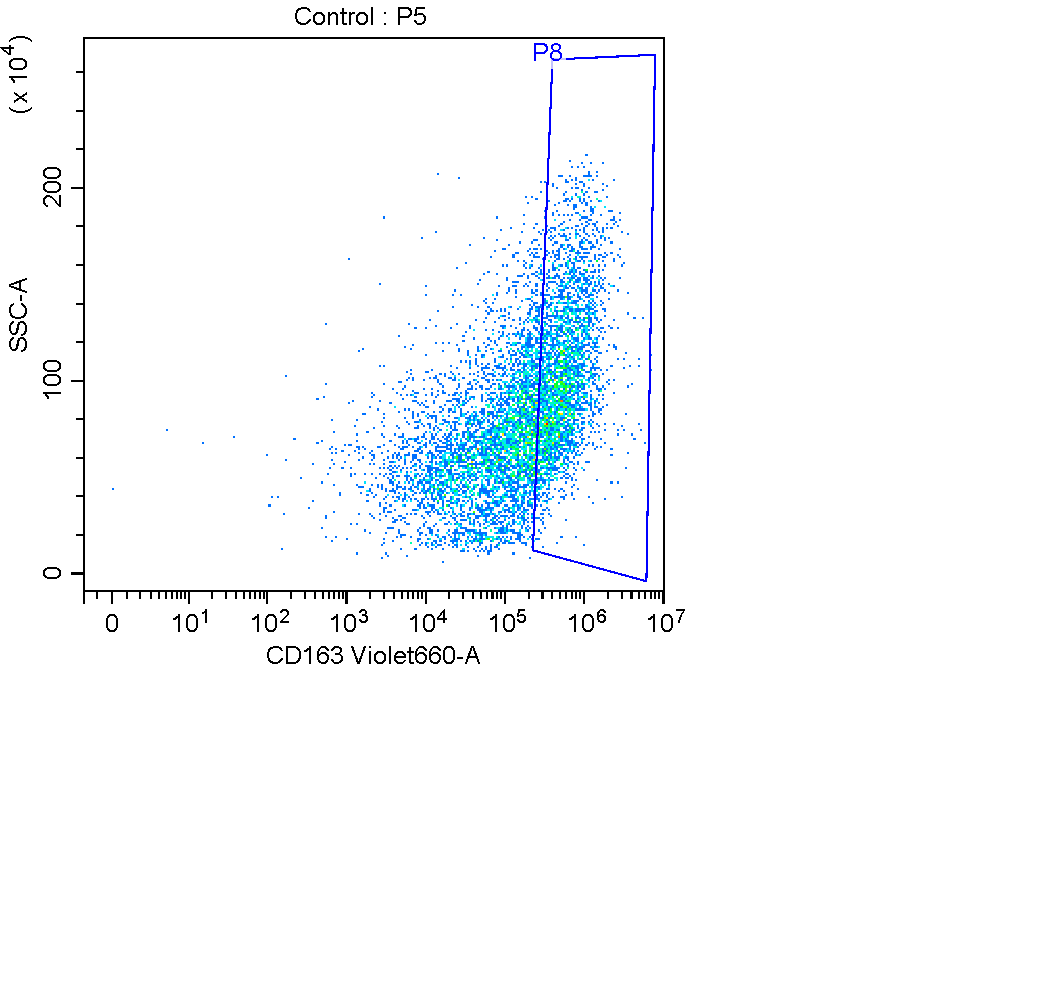

Supplement: Supplementary file 3 — Source Data for Figure 2 [file EMMM-15-e17601-s003.zip › Figure 2-2/2A/2A-Control-4.bmp]

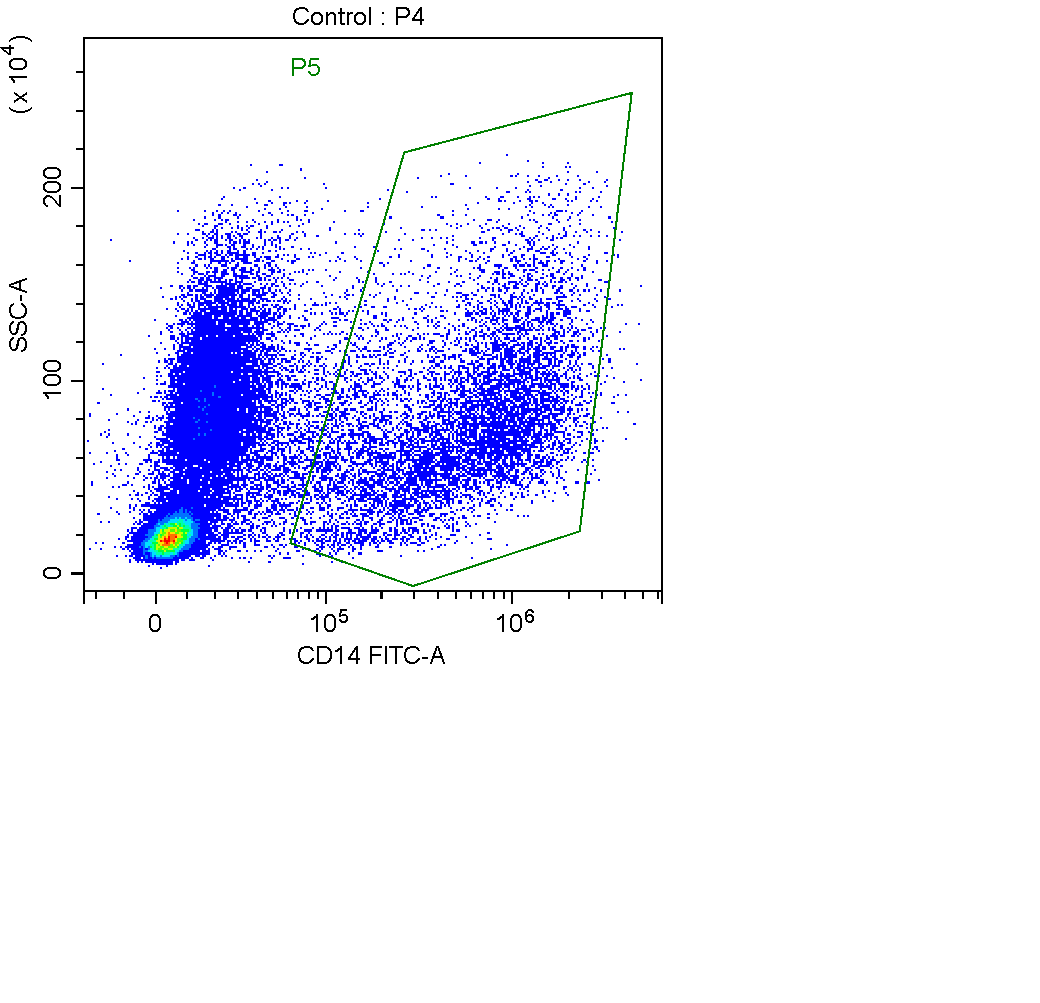

Supplement: Supplementary file 3 — Source Data for Figure 2 [file EMMM-15-e17601-s003.zip › Figure 2-2/2A/2A-Control-1.bmp]

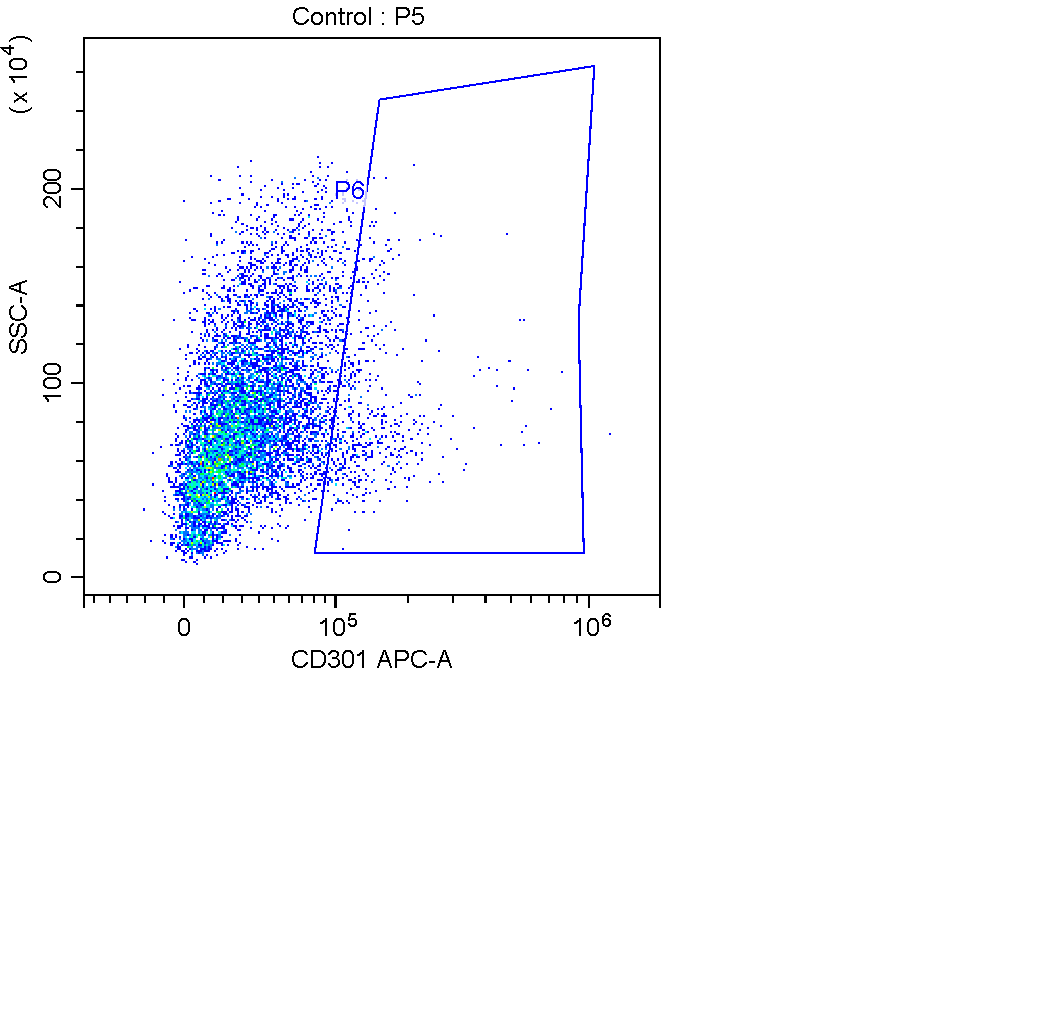

Supplement: Supplementary file 3 — Source Data for Figure 2 [file EMMM-15-e17601-s003.zip › Figure 2-2/2A/2A-Control-2.bmp]

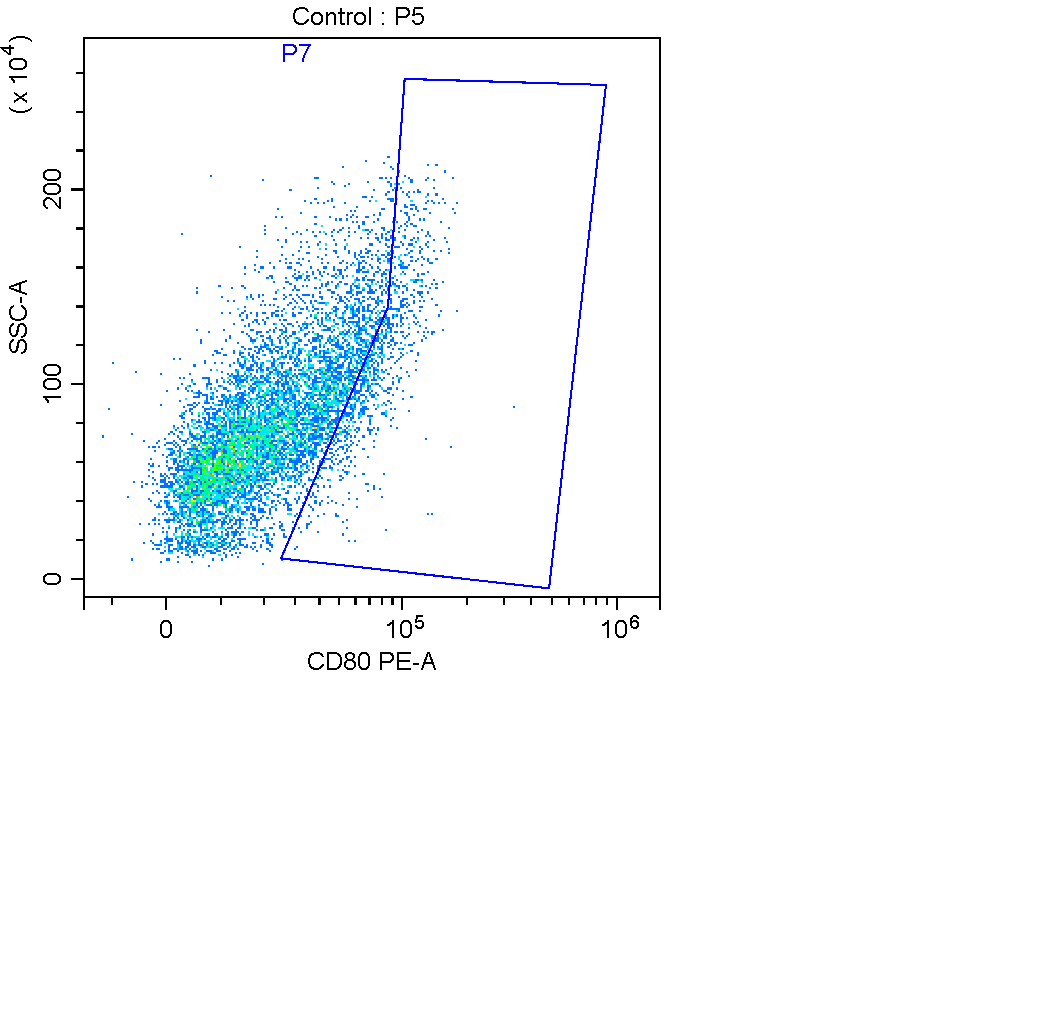

Supplement: Supplementary file 3 — Source Data for Figure 2 [file EMMM-15-e17601-s003.zip › Figure 2-2/2A/2A-Control-3.bmp]

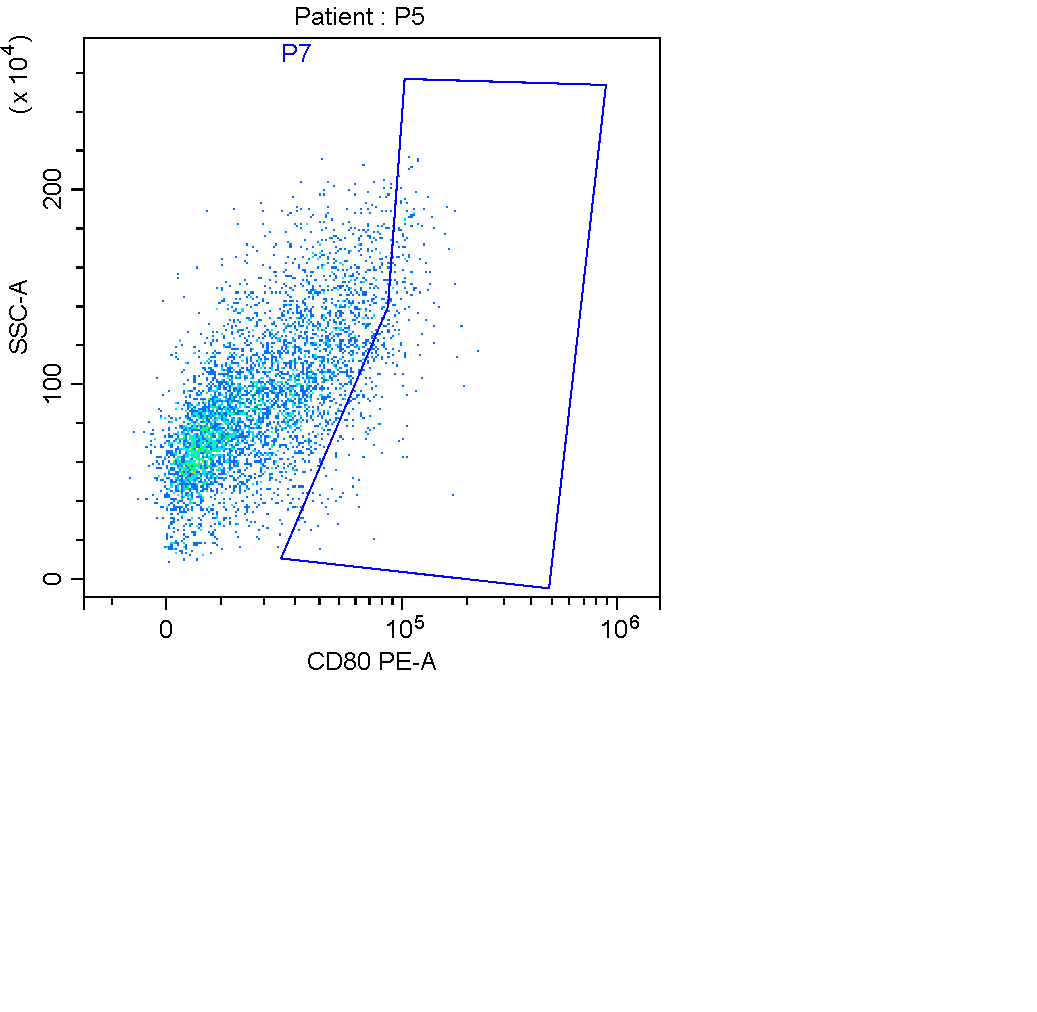

Supplement: Supplementary file 3 — Source Data for Figure 2 [file EMMM-15-e17601-s003.zip › Figure 2-2/2A/2A-Patient-3.bmp]

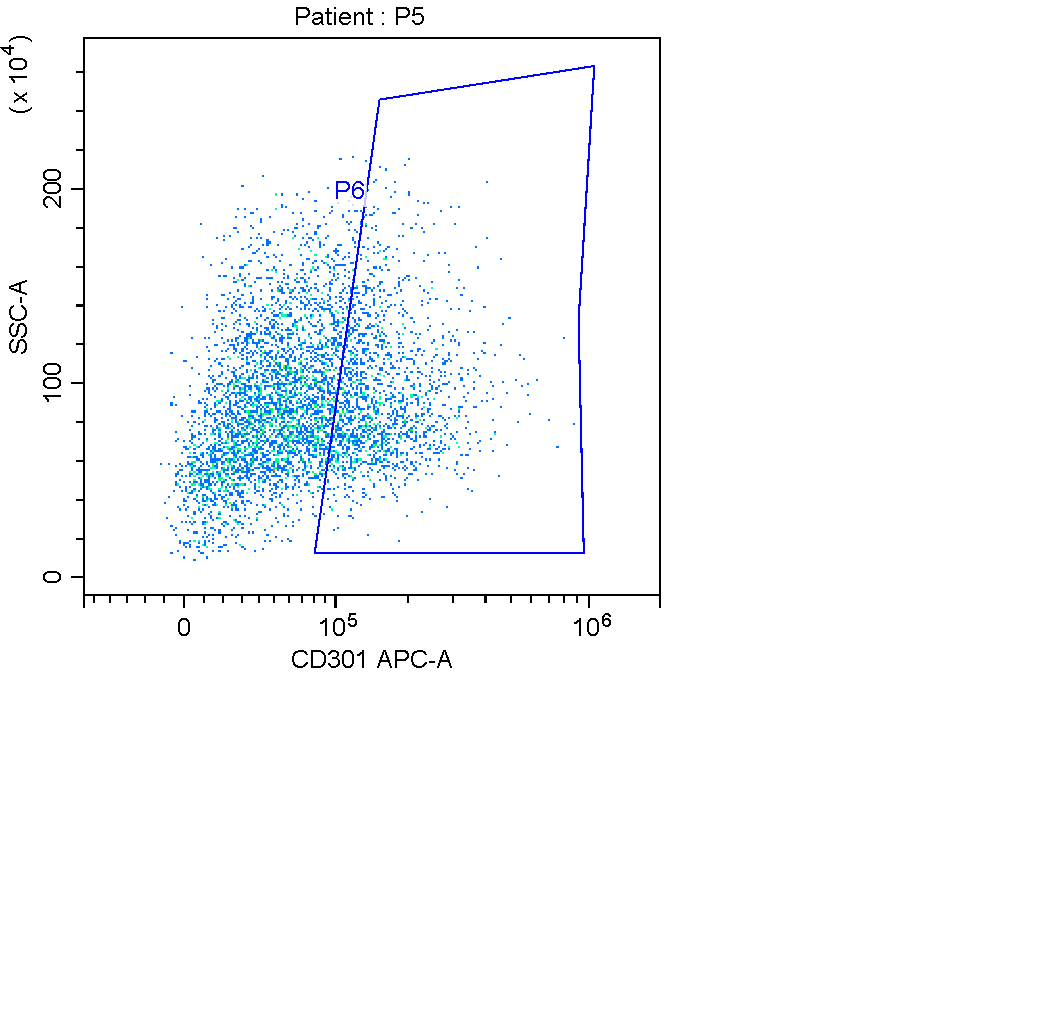

Supplement: Supplementary file 3 — Source Data for Figure 2 [file EMMM-15-e17601-s003.zip › Figure 2-2/2A/2A-Patient-2.bmp]

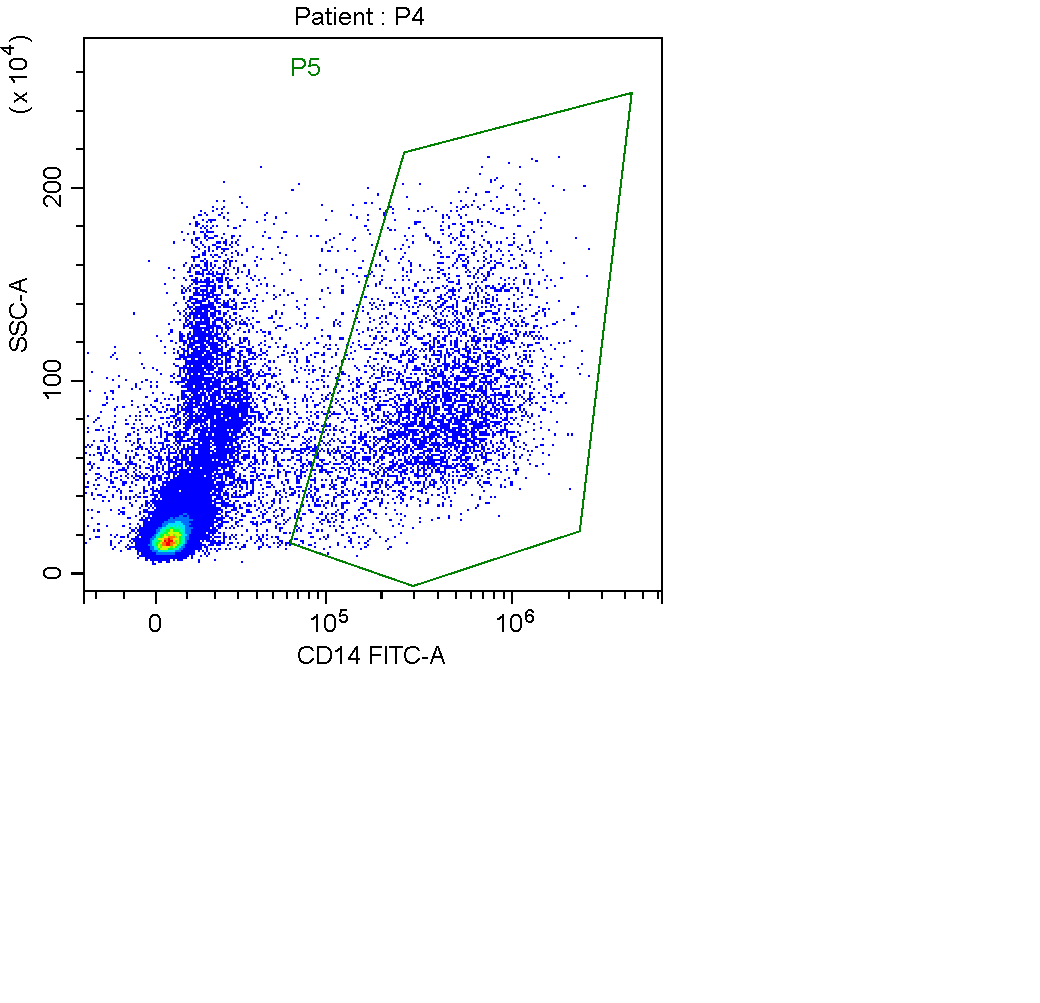

Supplement: Supplementary file 3 — Source Data for Figure 2 [file EMMM-15-e17601-s003.zip › Figure 2-2/2A/2A-Patient-1.bmp]

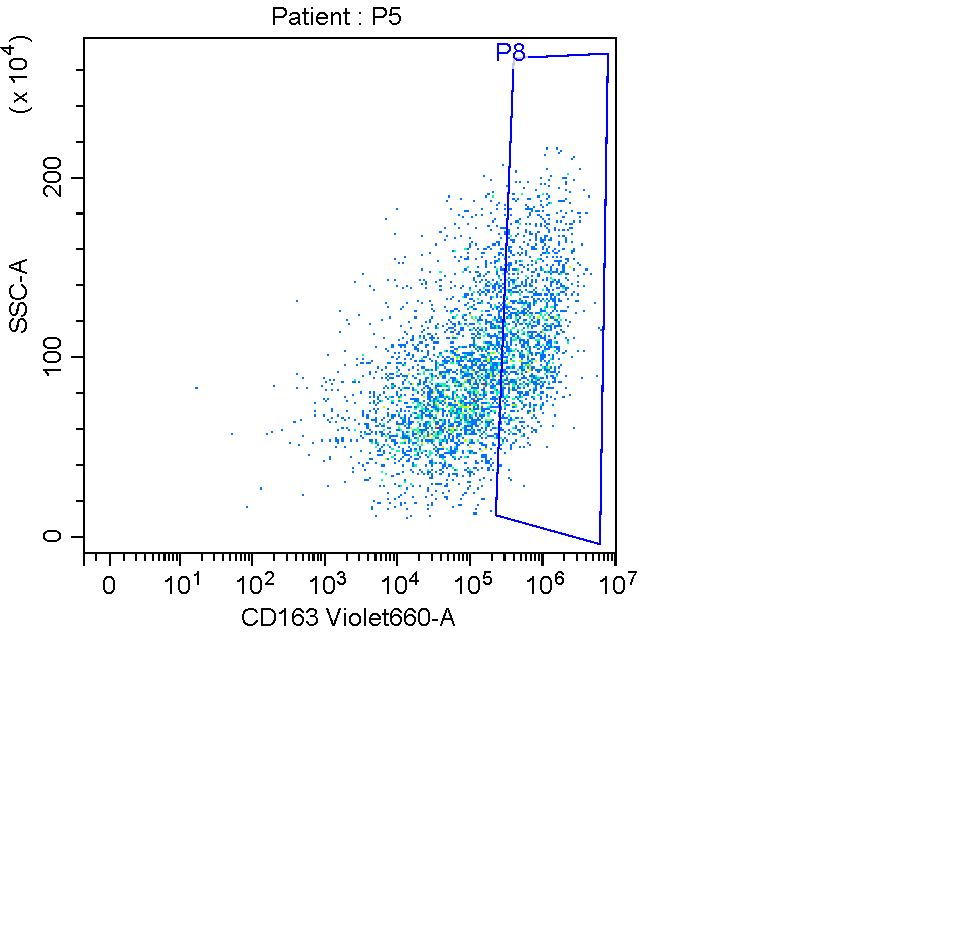

Supplement: Supplementary file 3 — Source Data for Figure 2 [file EMMM-15-e17601-s003.zip › Figure 2-2/2A/2A-Patient-4.bmp]

**CD301**

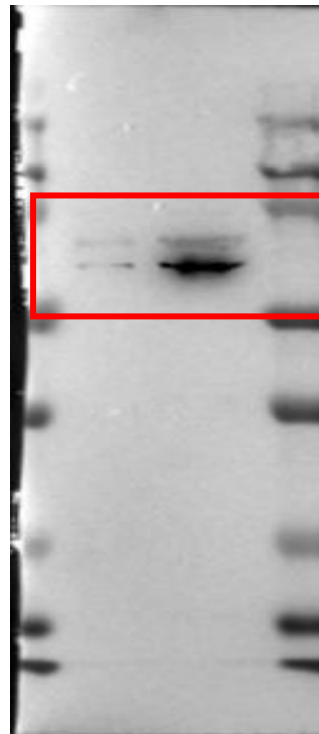

**CD14**

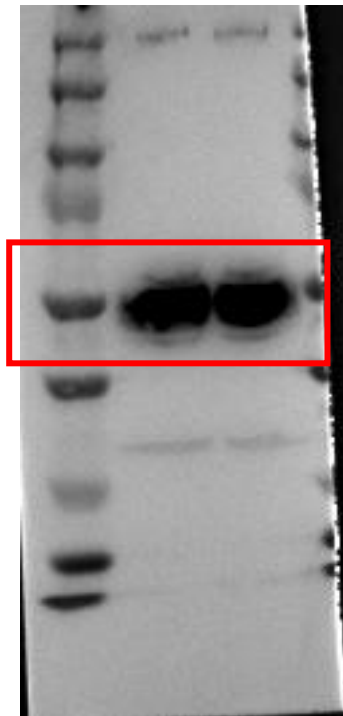

**$\beta$ -actin**

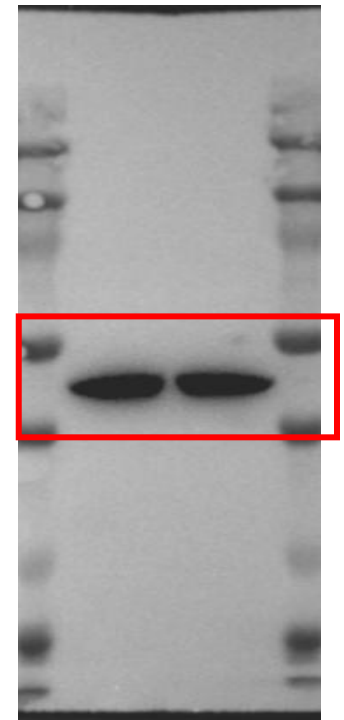

Supplement: Supplementary file 3 — Source Data for Figure 2 [file EMMM-15-e17601-s003.zip › Figure 2-2/2F/2F blot.pdf]

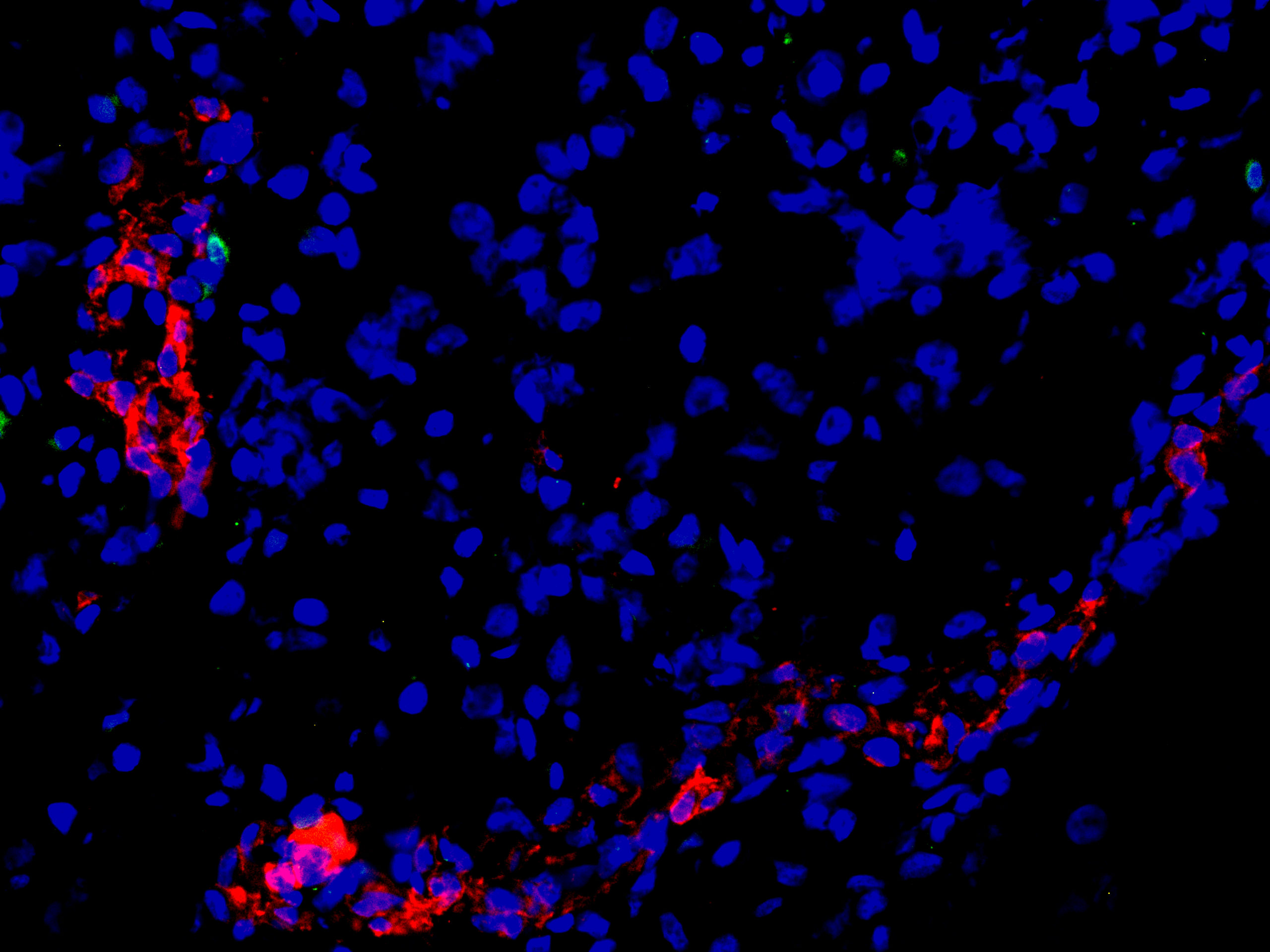

Supplement: Supplementary file 3 — Source Data for Figure 2 [file EMMM-15-e17601-s003.zip › Figure 2-2/2C/2C normal.tif]

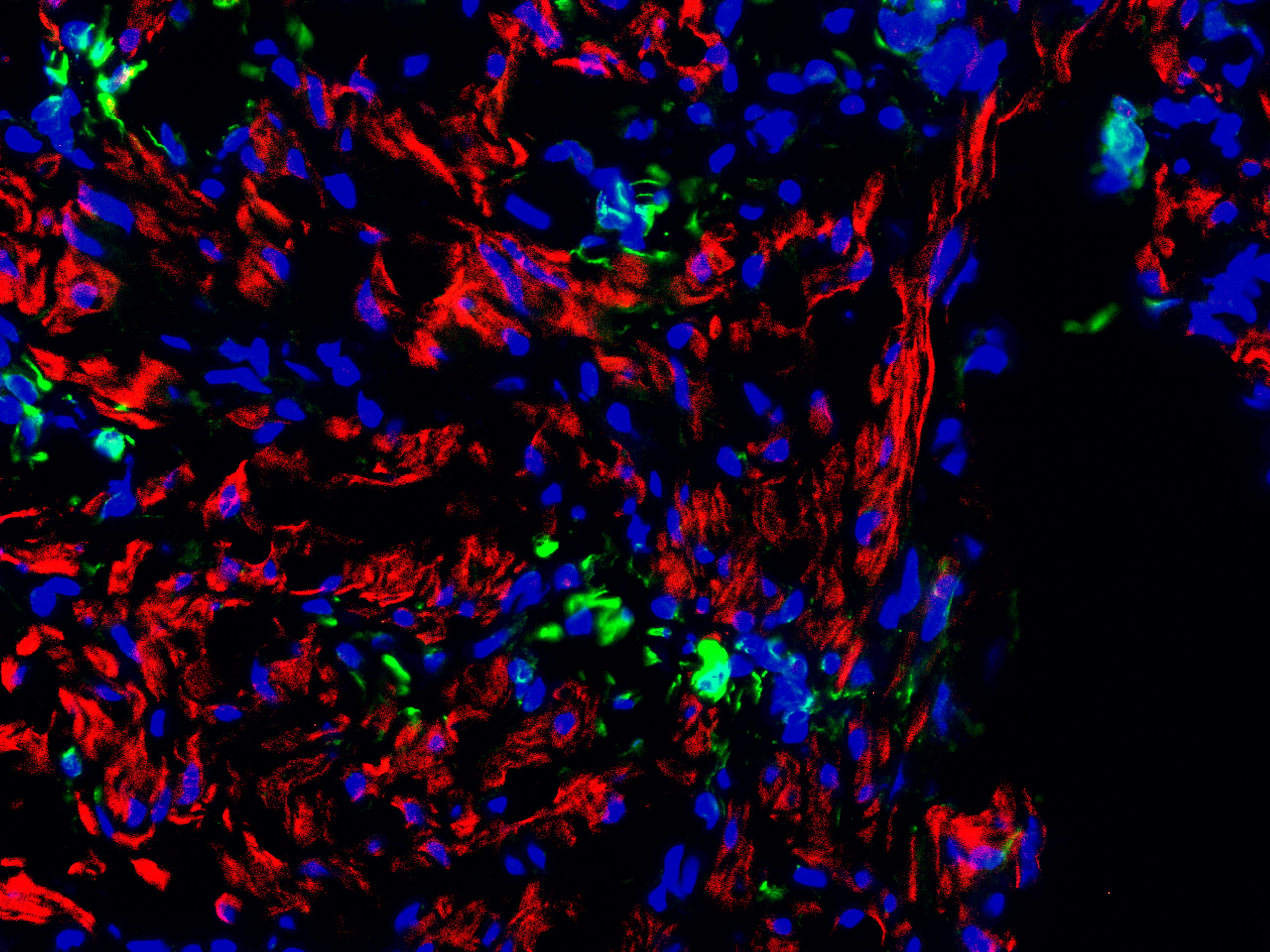

Supplement: Supplementary file 3 — Source Data for Figure 2 [file EMMM-15-e17601-s003.zip › Figure 2-2/2C/2C patient.tif]

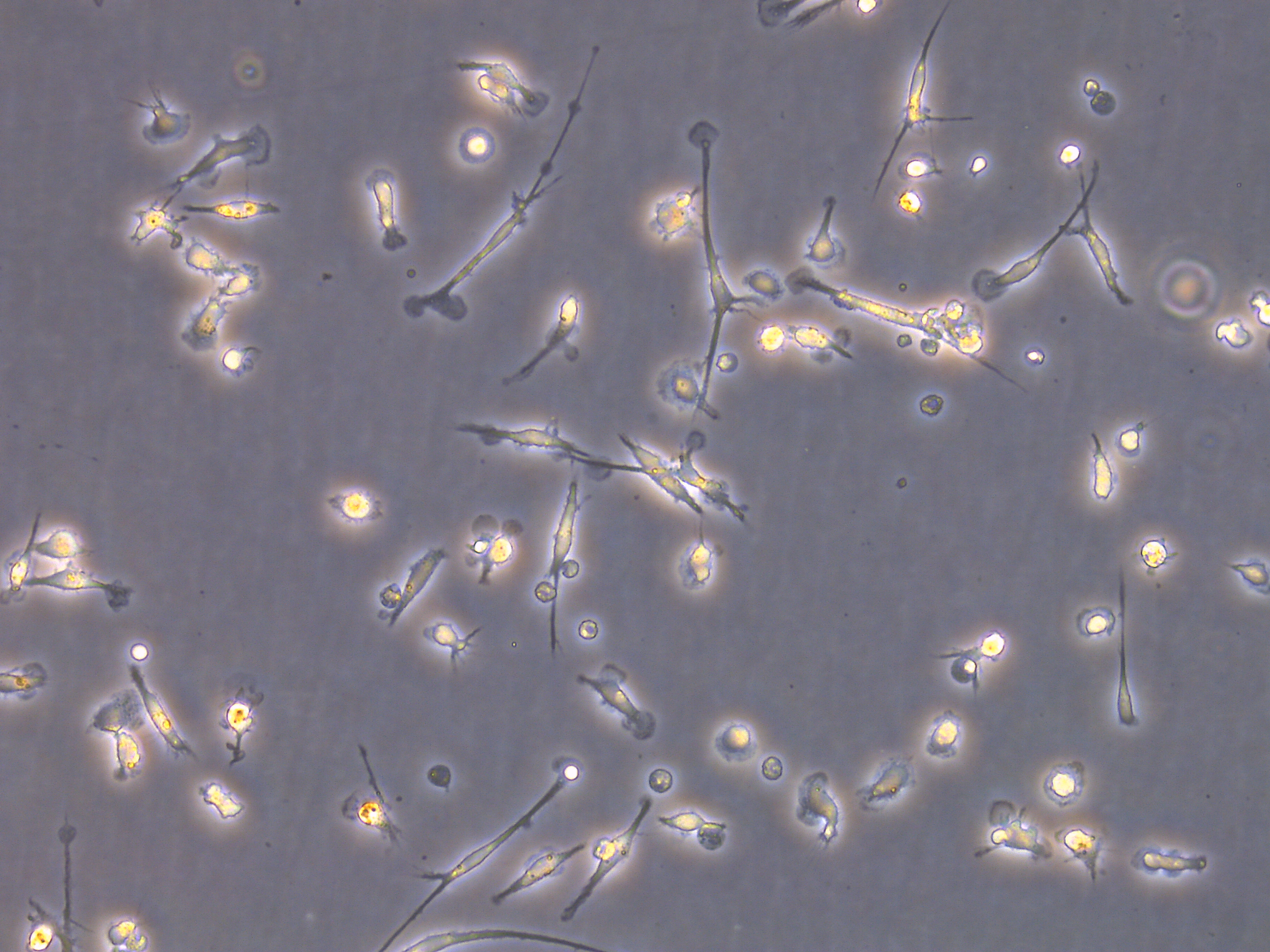

Supplement: Supplementary file 3 — Source Data for Figure 2 [file EMMM-15-e17601-s003.zip › Figure 2-2/2E/2E CD301+.jpg]

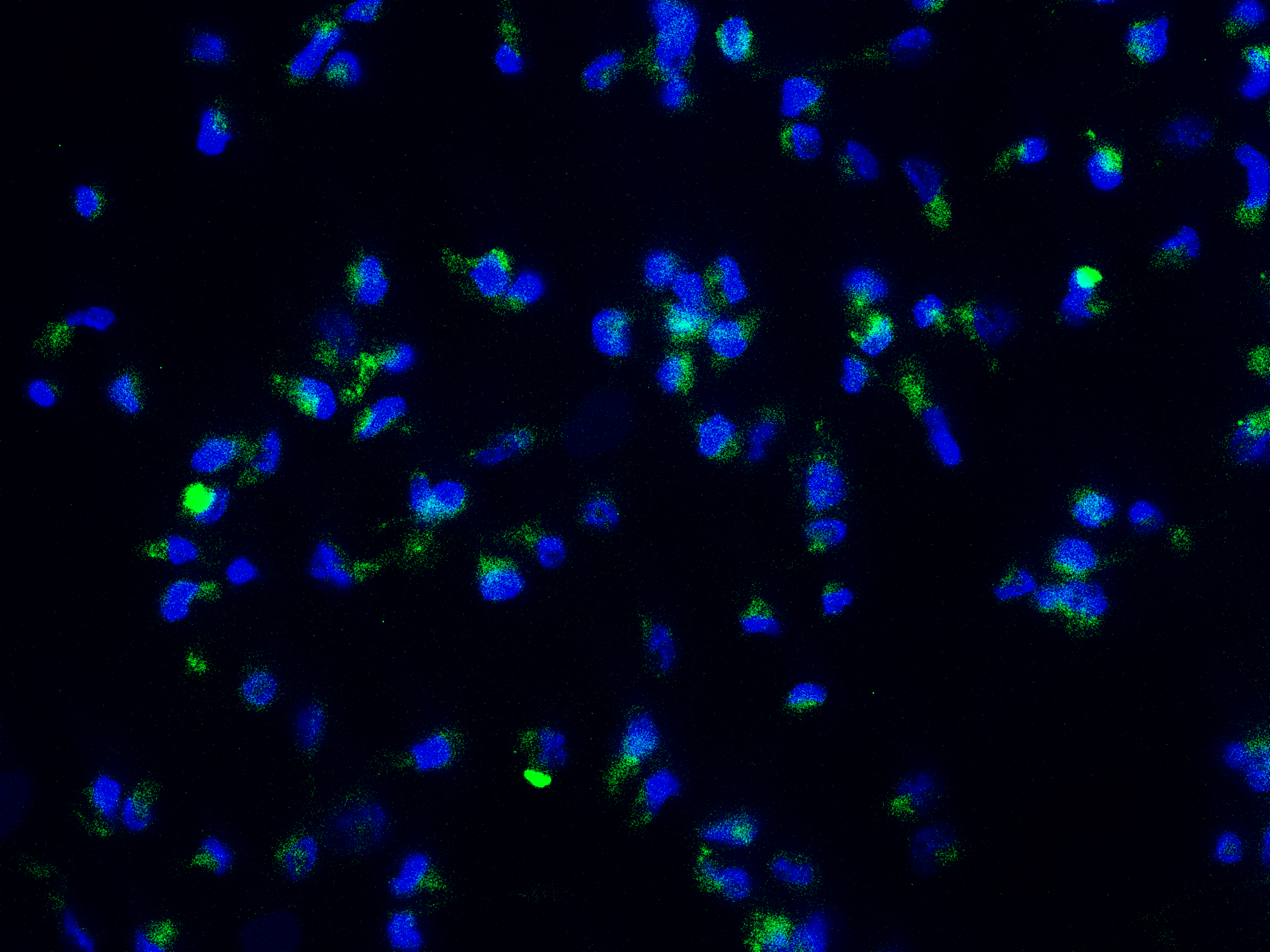

Supplement: Supplementary file 3 — Source Data for Figure 2 [file EMMM-15-e17601-s003.zip › Figure 2-2/2E/2E IF CD301-.tif]

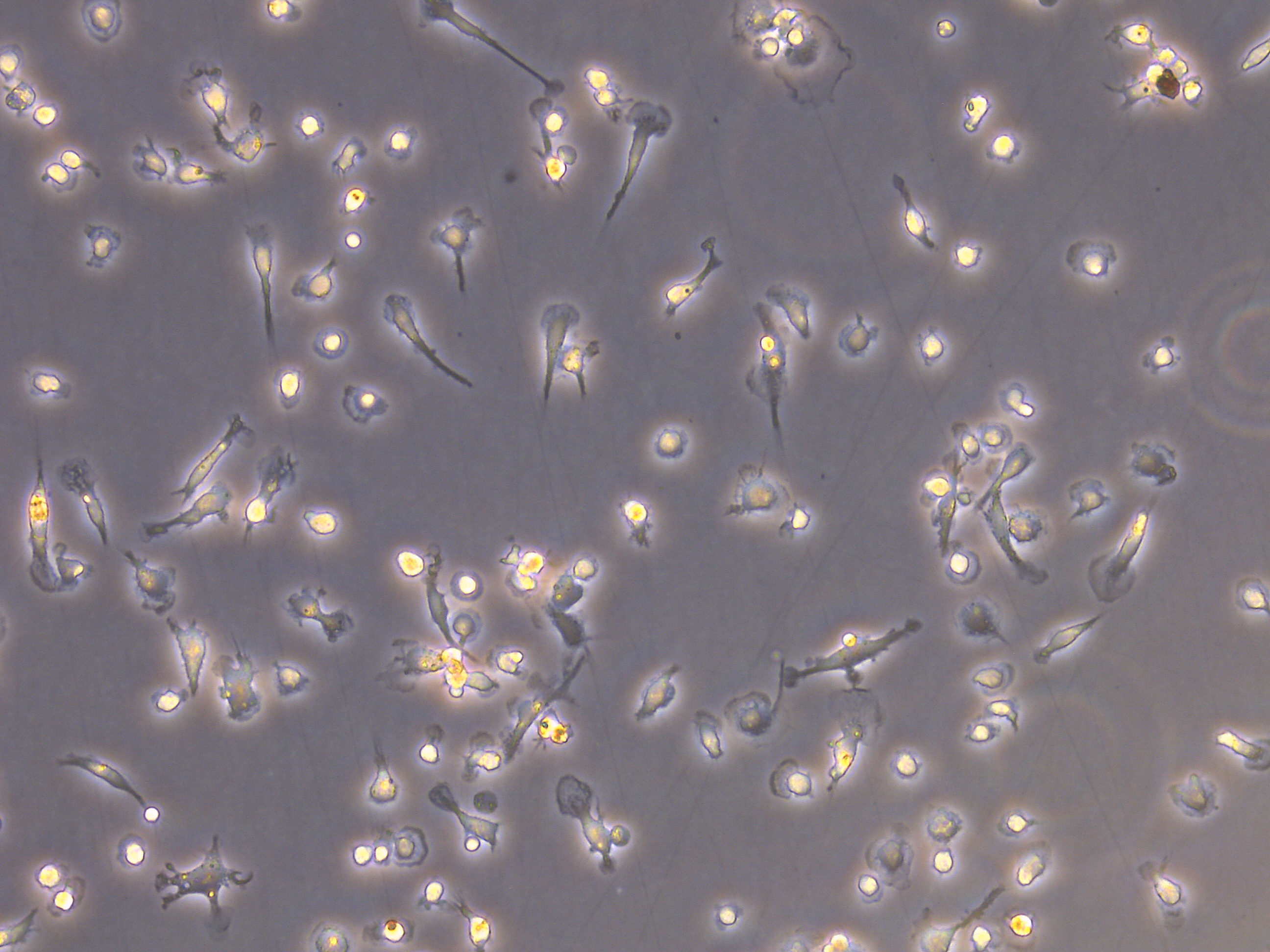

Supplement: Supplementary file 3 — Source Data for Figure 2 [file EMMM-15-e17601-s003.zip › Figure 2-2/2E/2E CD301-.jpg]

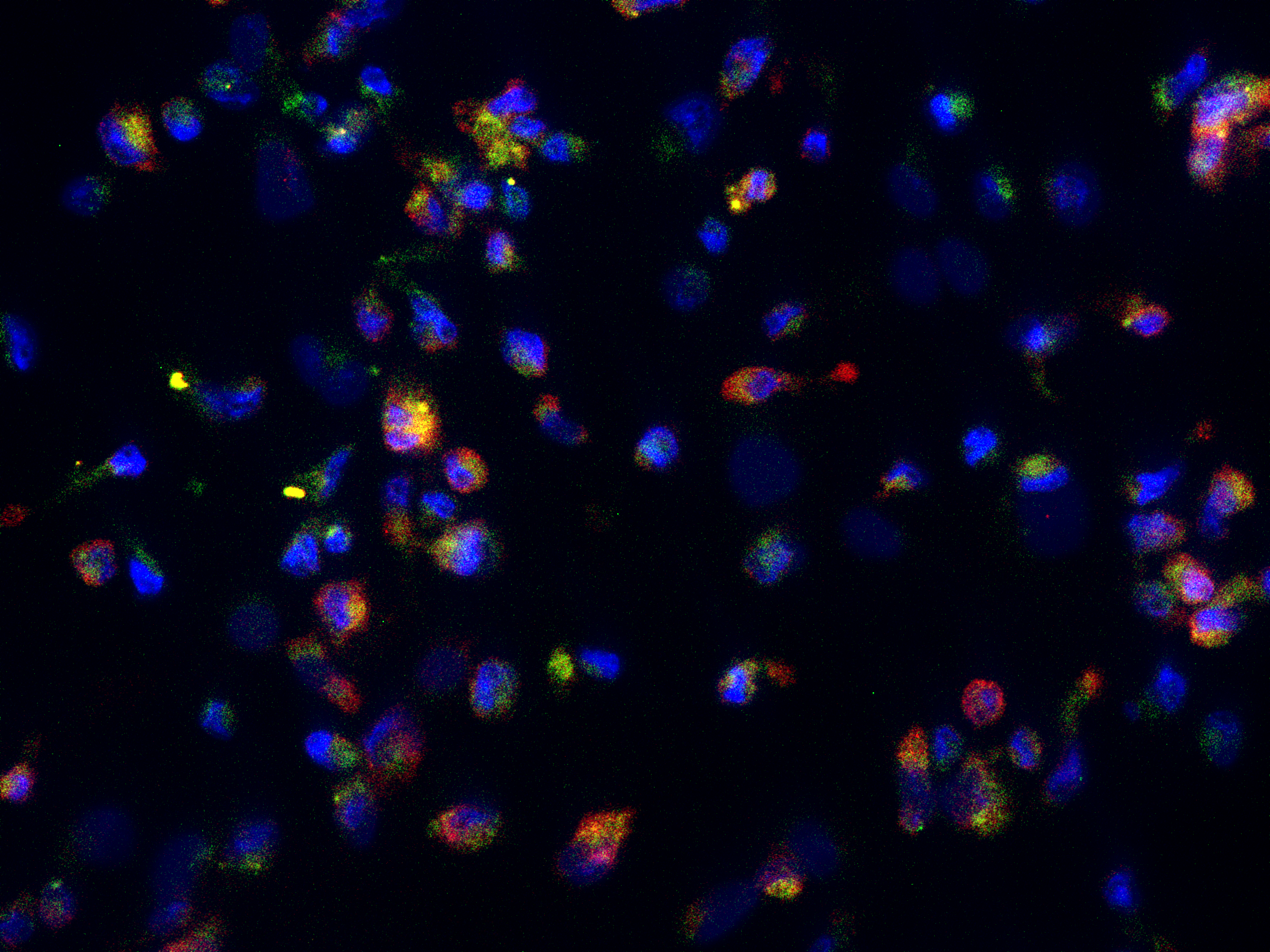

Supplement: Supplementary file 3 — Source Data for Figure 2 [file EMMM-15-e17601-s003.zip › Figure 2-2/2E/2E IF CD301+.tif]

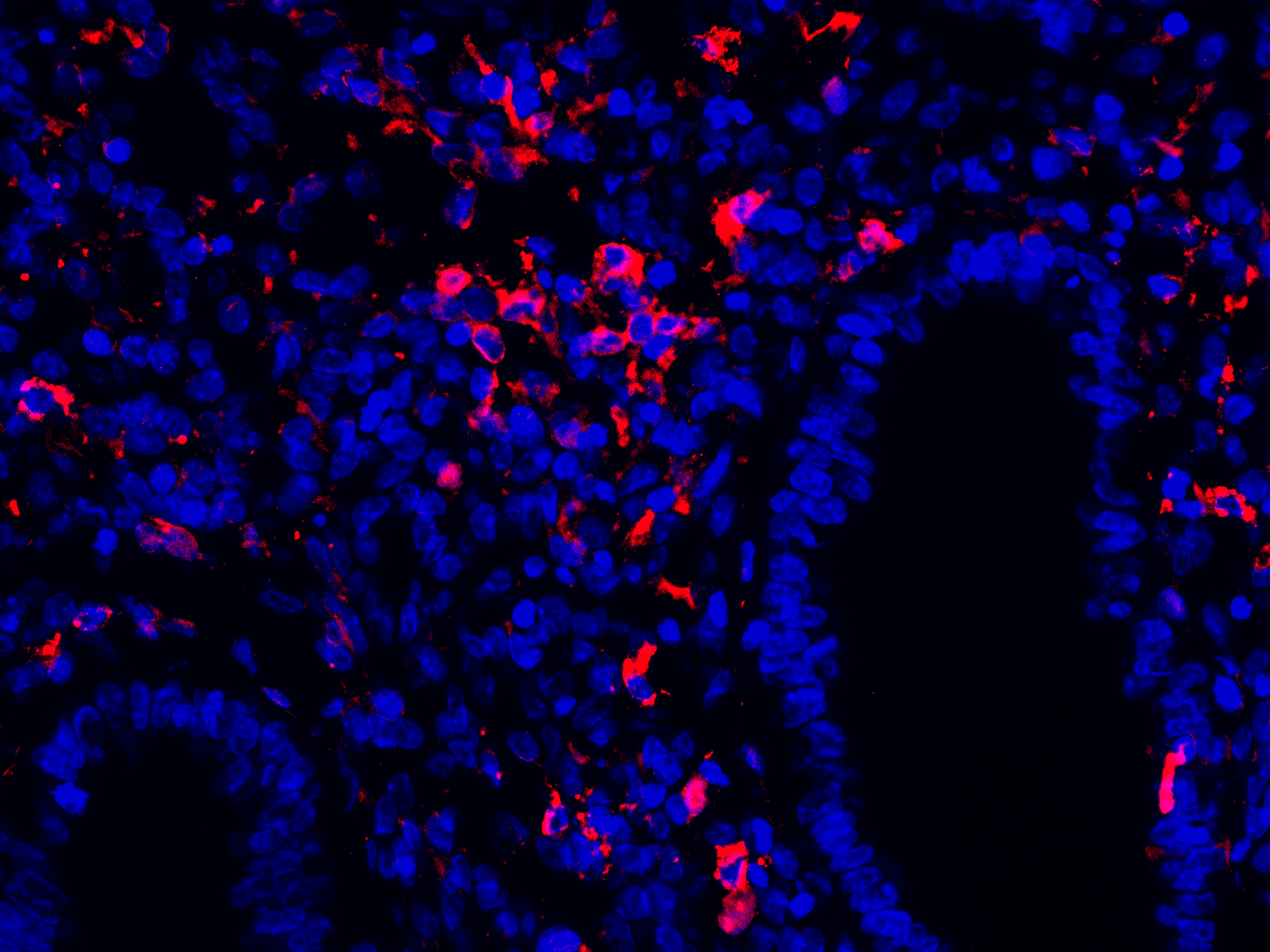

Supplement: Supplementary file 3 — Source Data for Figure 2 [file EMMM-15-e17601-s003.zip › Figure 2-2/2B/2B patient CD68.tif]

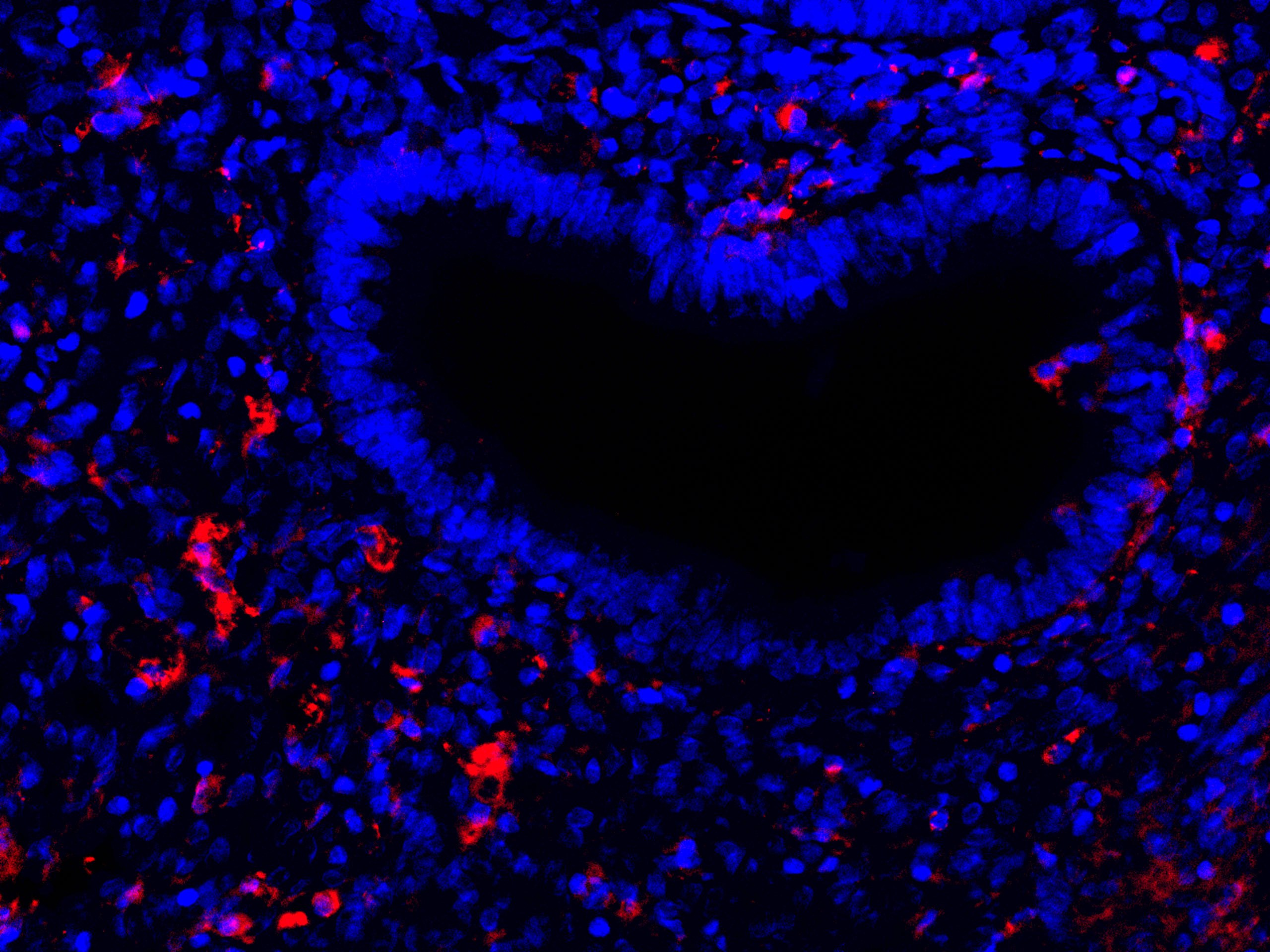

Supplement: Supplementary file 3 — Source Data for Figure 2 [file EMMM-15-e17601-s003.zip › Figure 2-2/2B/2B control CD68.tif]

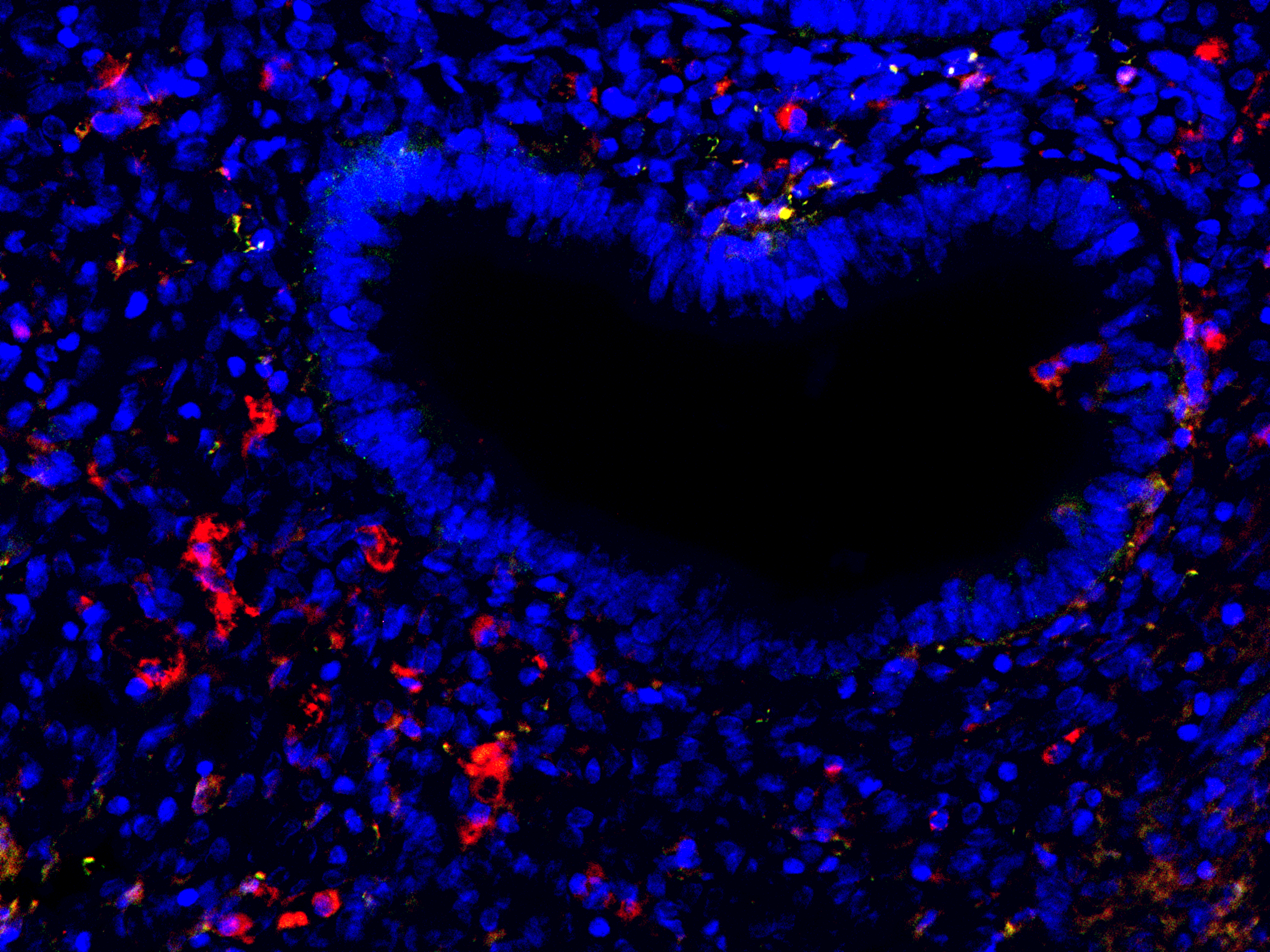

Supplement: Supplementary file 3 — Source Data for Figure 2 [file EMMM-15-e17601-s003.zip › Figure 2-2/2B/2B control CD68-CD301.tif]

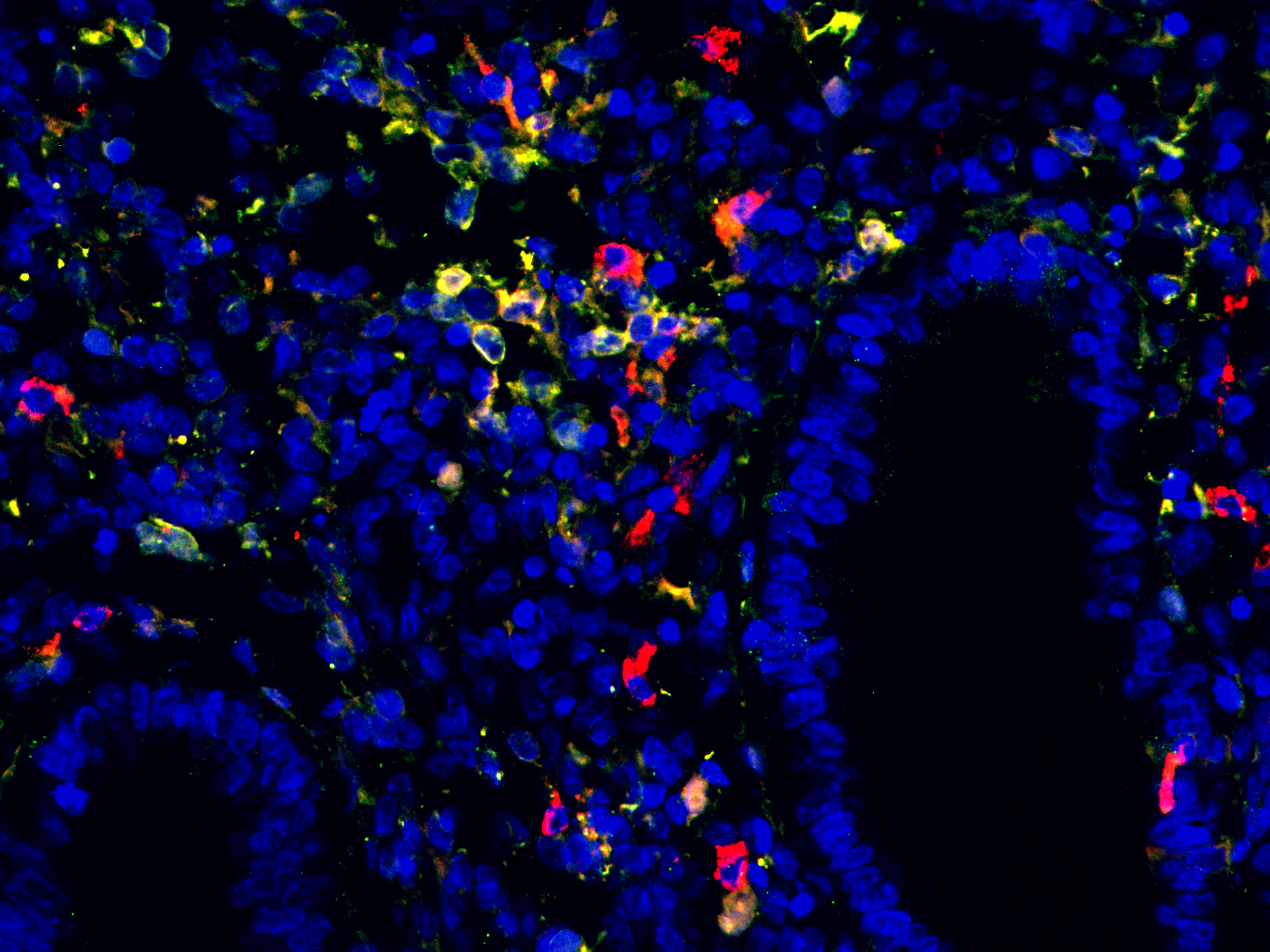

Supplement: Supplementary file 3 — Source Data for Figure 2 [file EMMM-15-e17601-s003.zip › Figure 2-2/2B/2B patient CD68-CD301.tif]

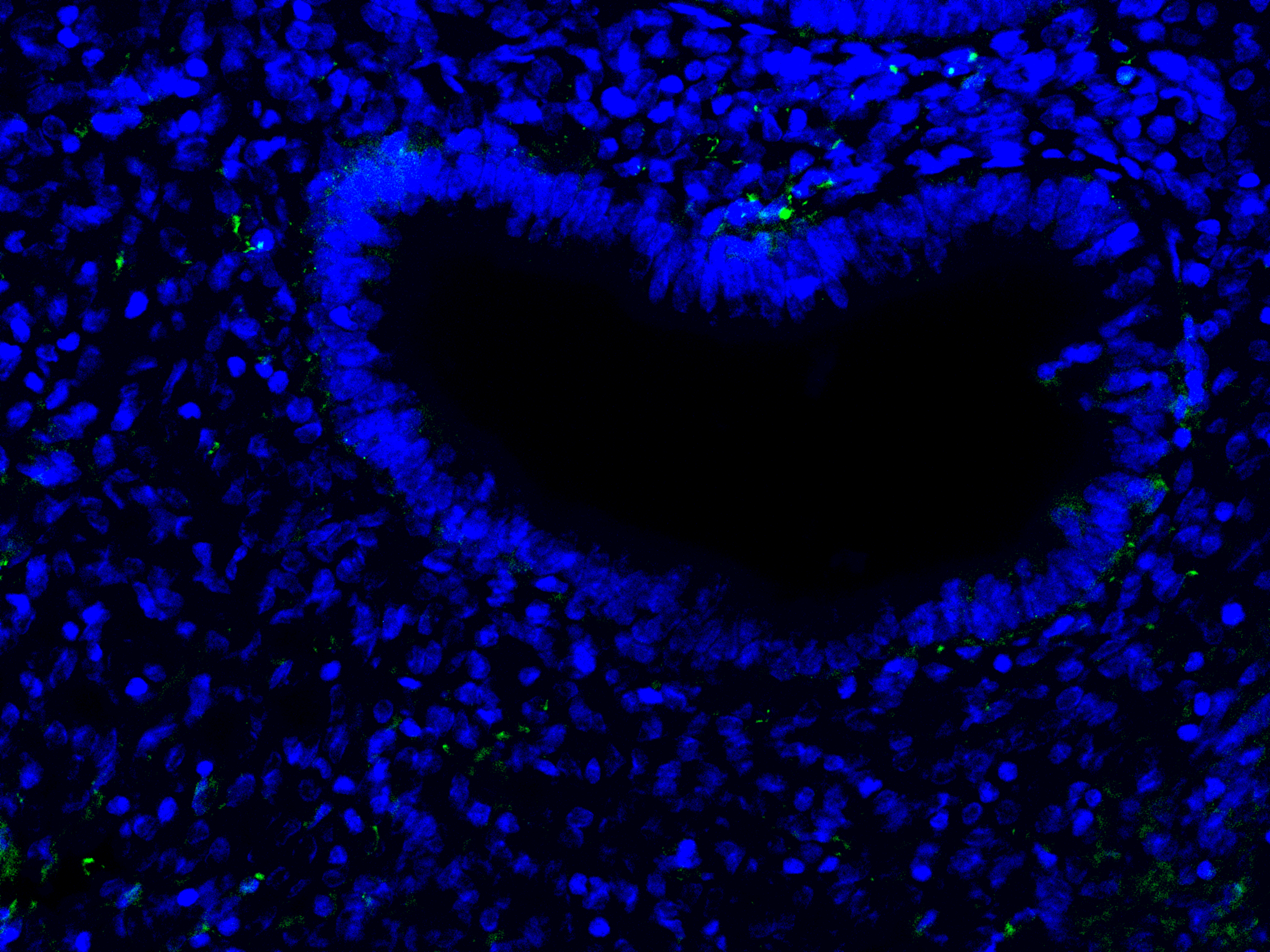

Supplement: Supplementary file 3 — Source Data for Figure 2 [file EMMM-15-e17601-s003.zip › Figure 2-2/2B/2B control CD301.tif]

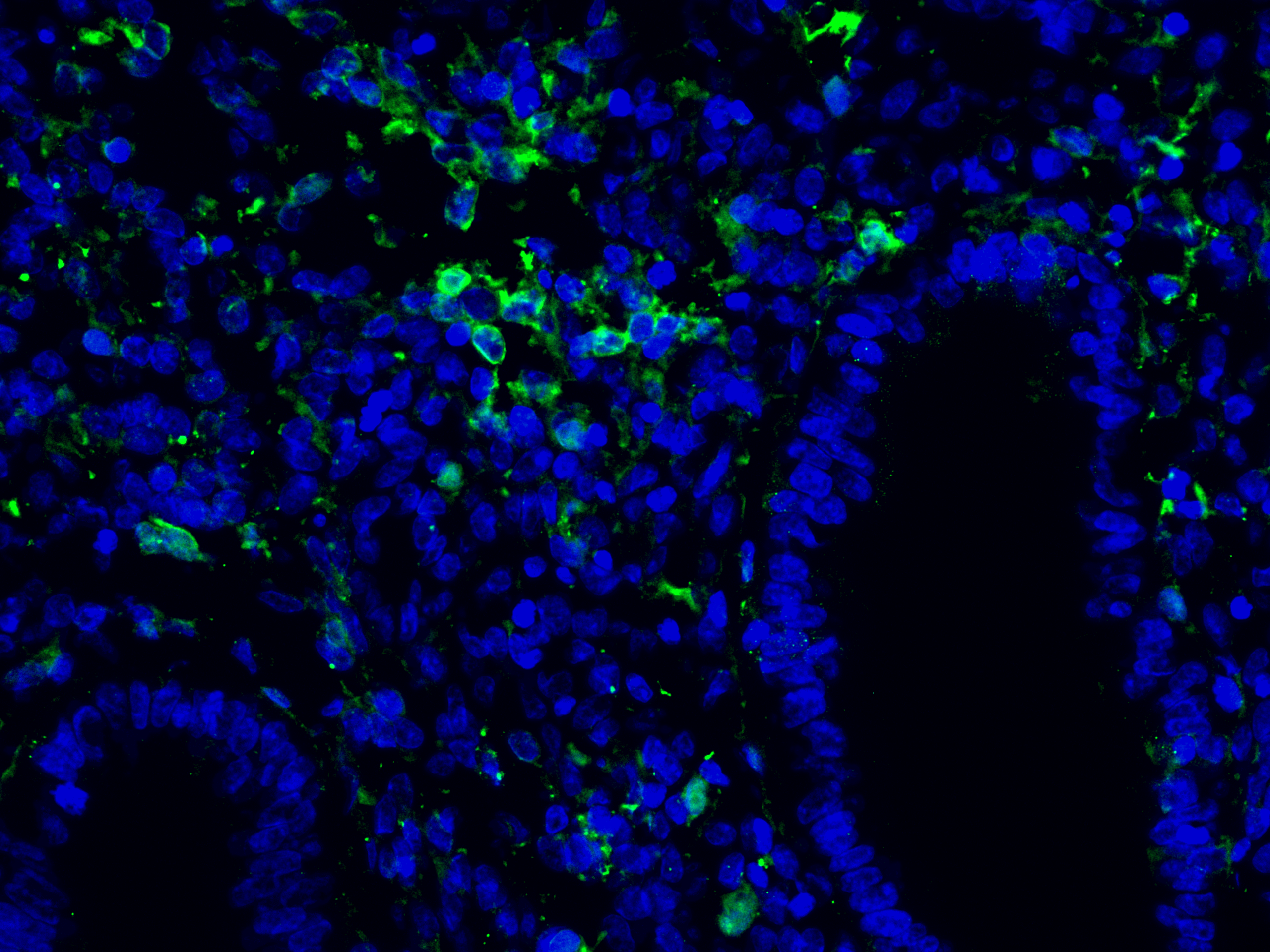

Supplement: Supplementary file 3 — Source Data for Figure 2 [file EMMM-15-e17601-s003.zip › Figure 2-2/2B/2B patient CD301.tif]

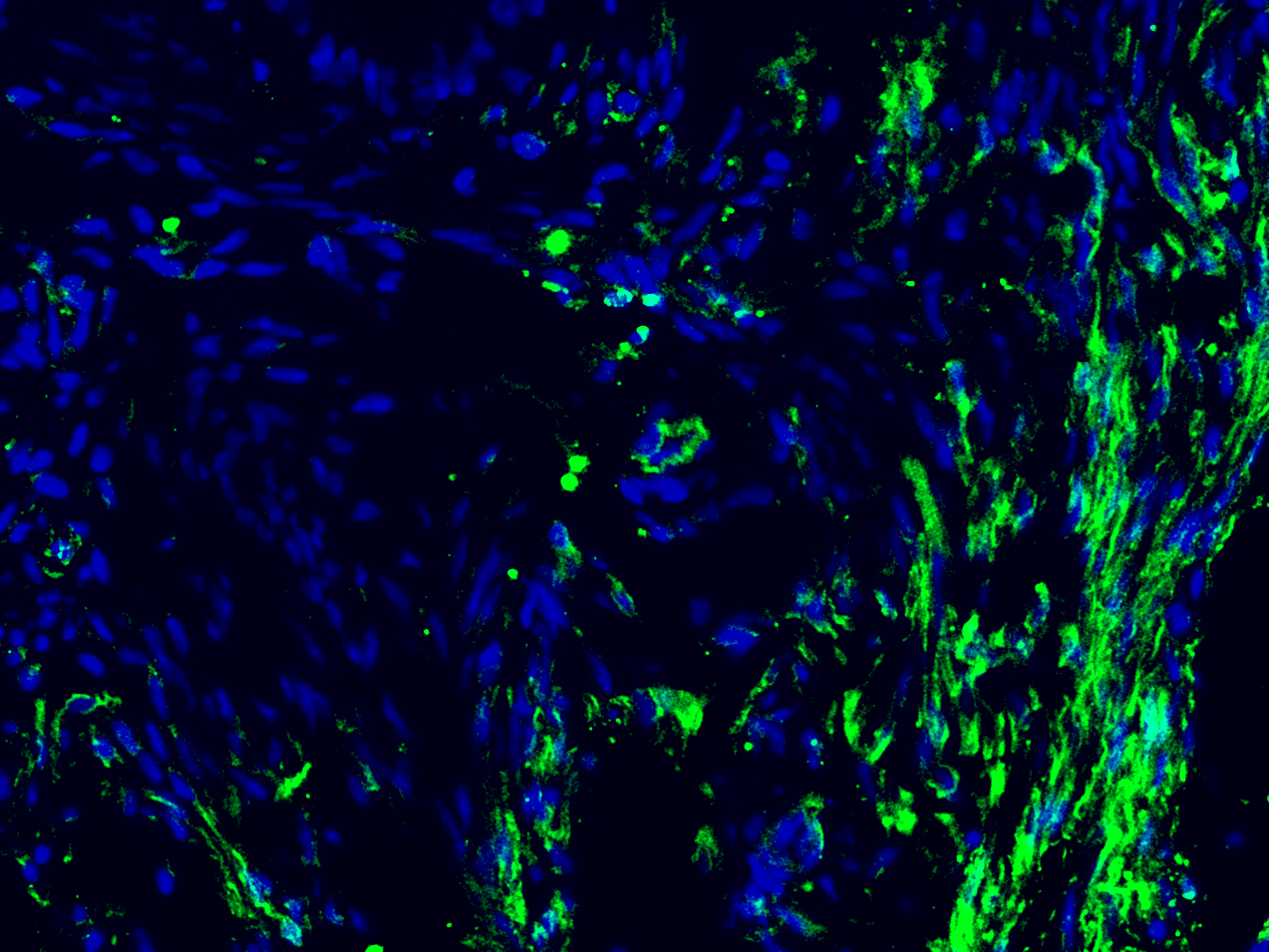

Supplement: Supplementary file 4 — Source Data for Figure 3 [file EMMM-15-e17601-s007.zip › Figure 3-2/3E/Patient AXL.tif]

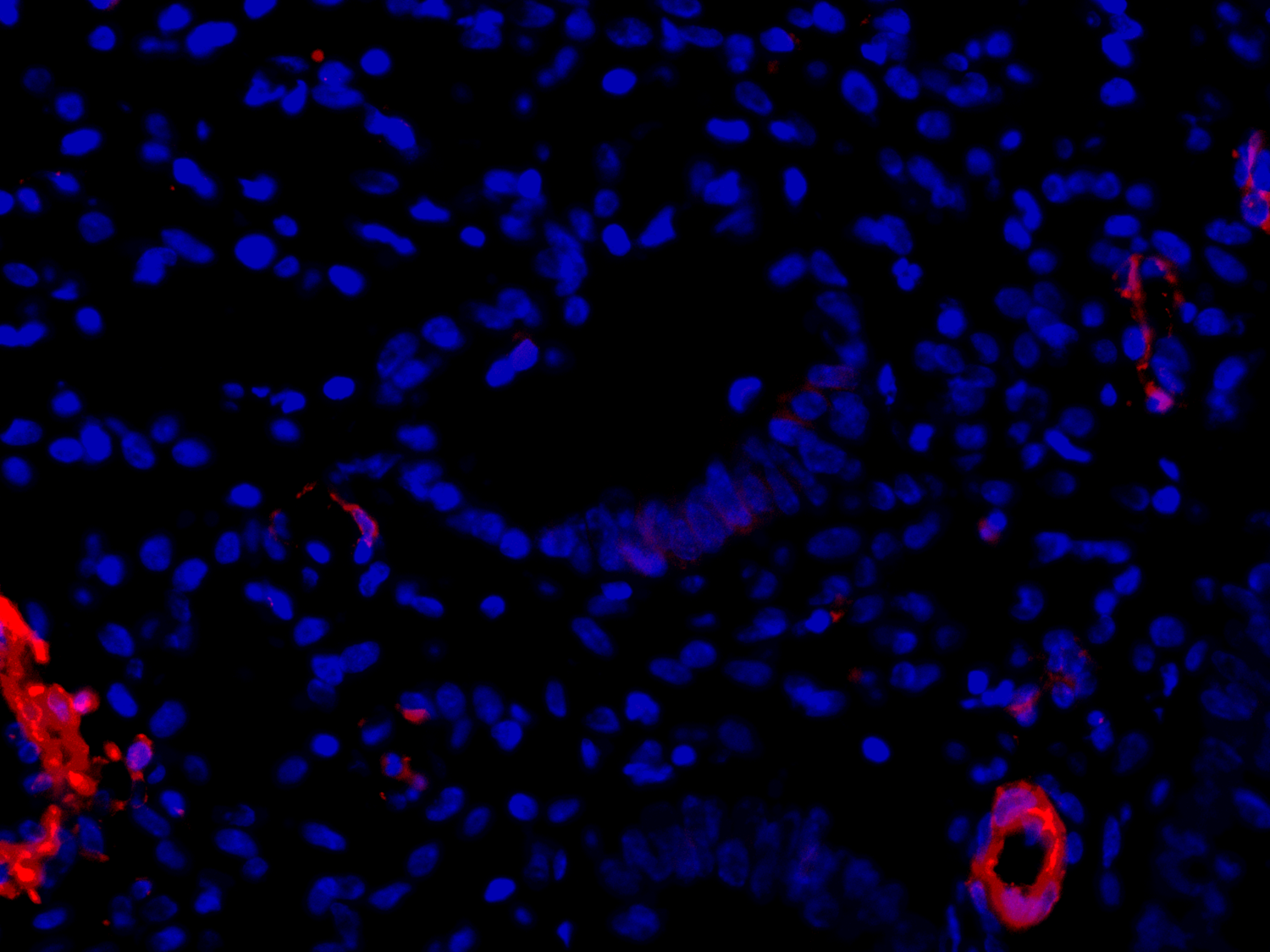

Supplement: Supplementary file 4 — Source Data for Figure 3 [file EMMM-15-e17601-s007.zip › Figure 3-2/3E/Control a-SMA.tif]

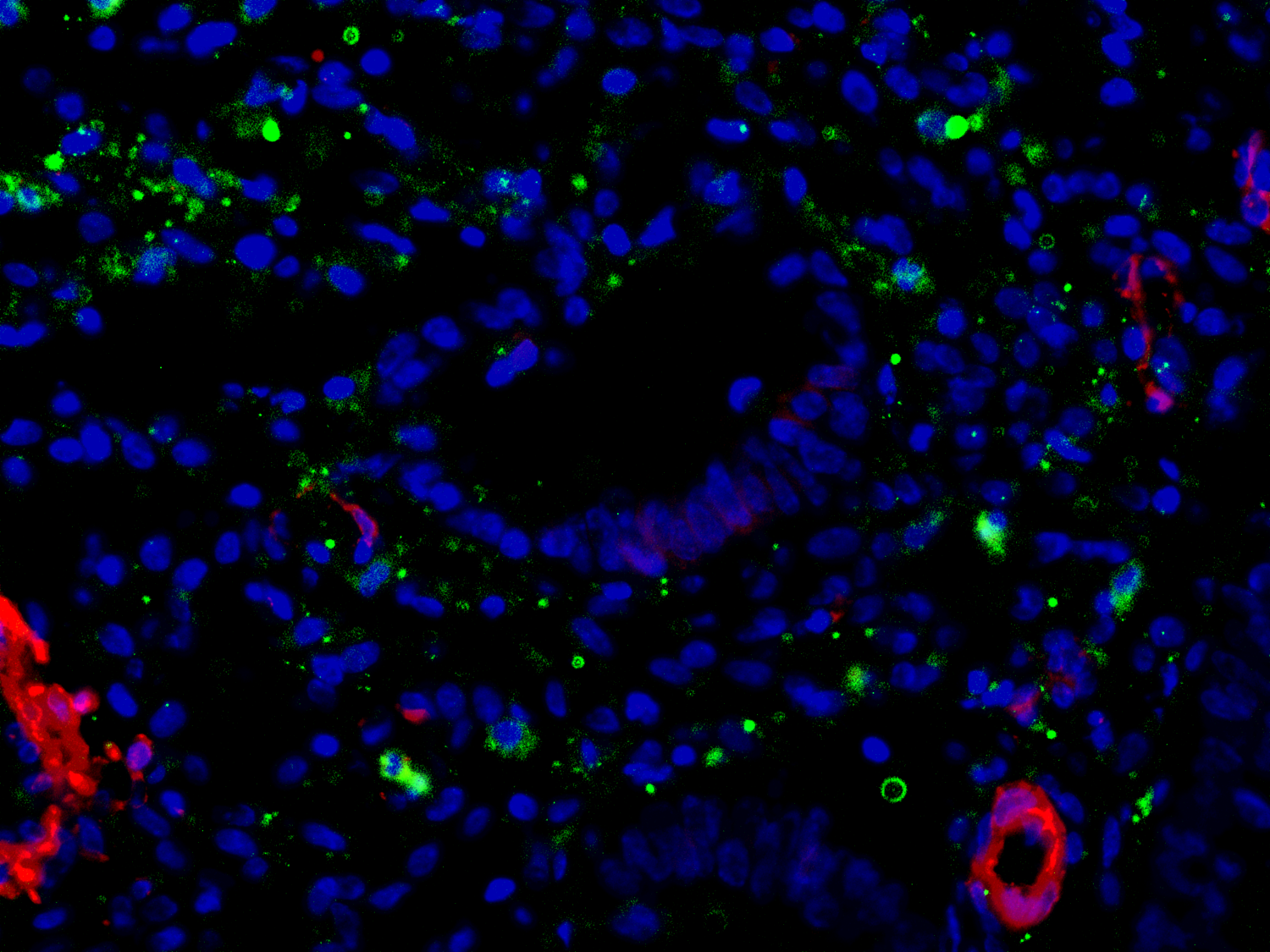

Supplement: Supplementary file 4 — Source Data for Figure 3 [file EMMM-15-e17601-s007.zip › Figure 3-2/3E/Control AXL-a-SMA.tif]

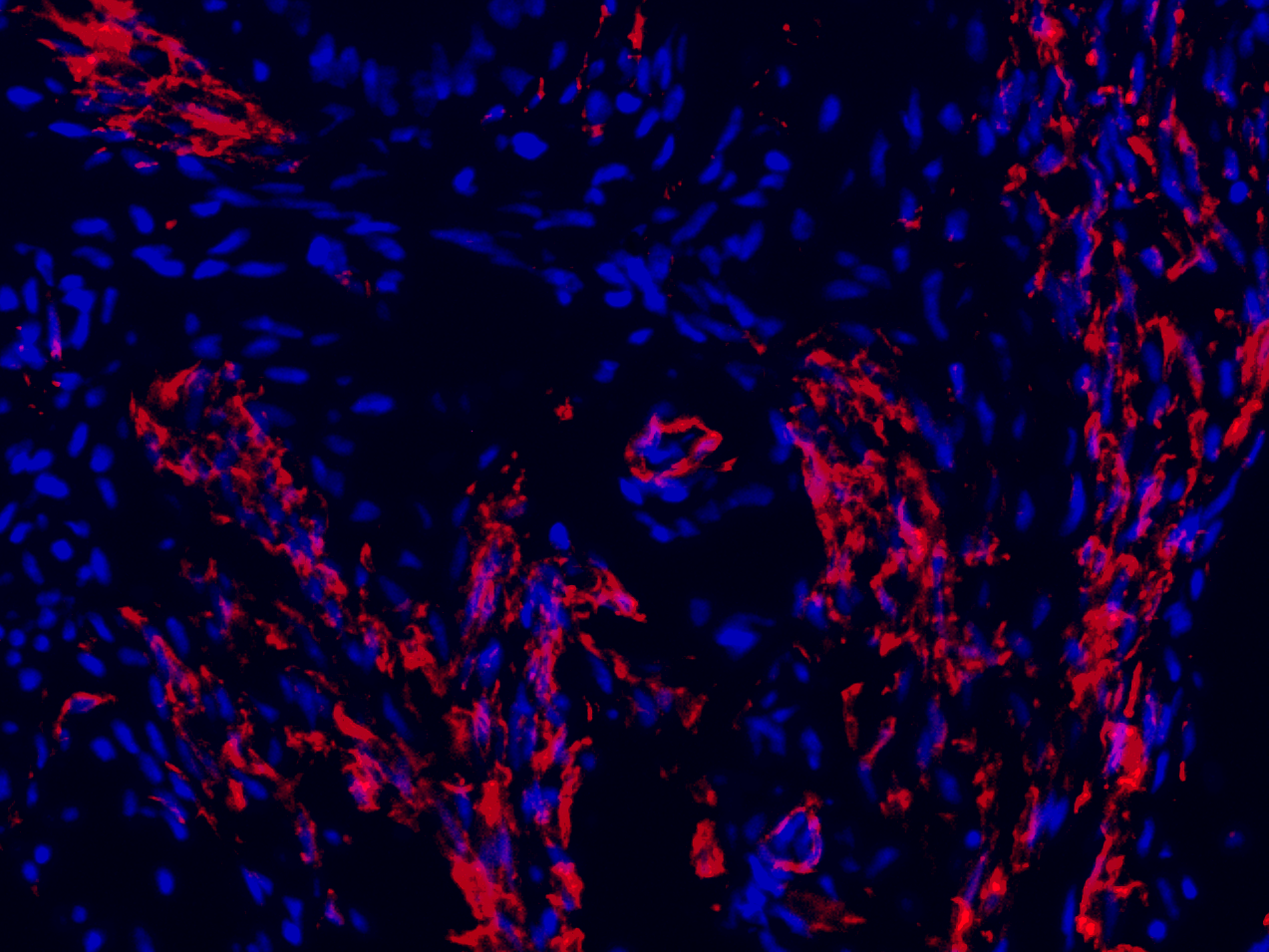

Supplement: Supplementary file 4 — Source Data for Figure 3 [file EMMM-15-e17601-s007.zip › Figure 3-2/3E/Patient a-SMA.tif]

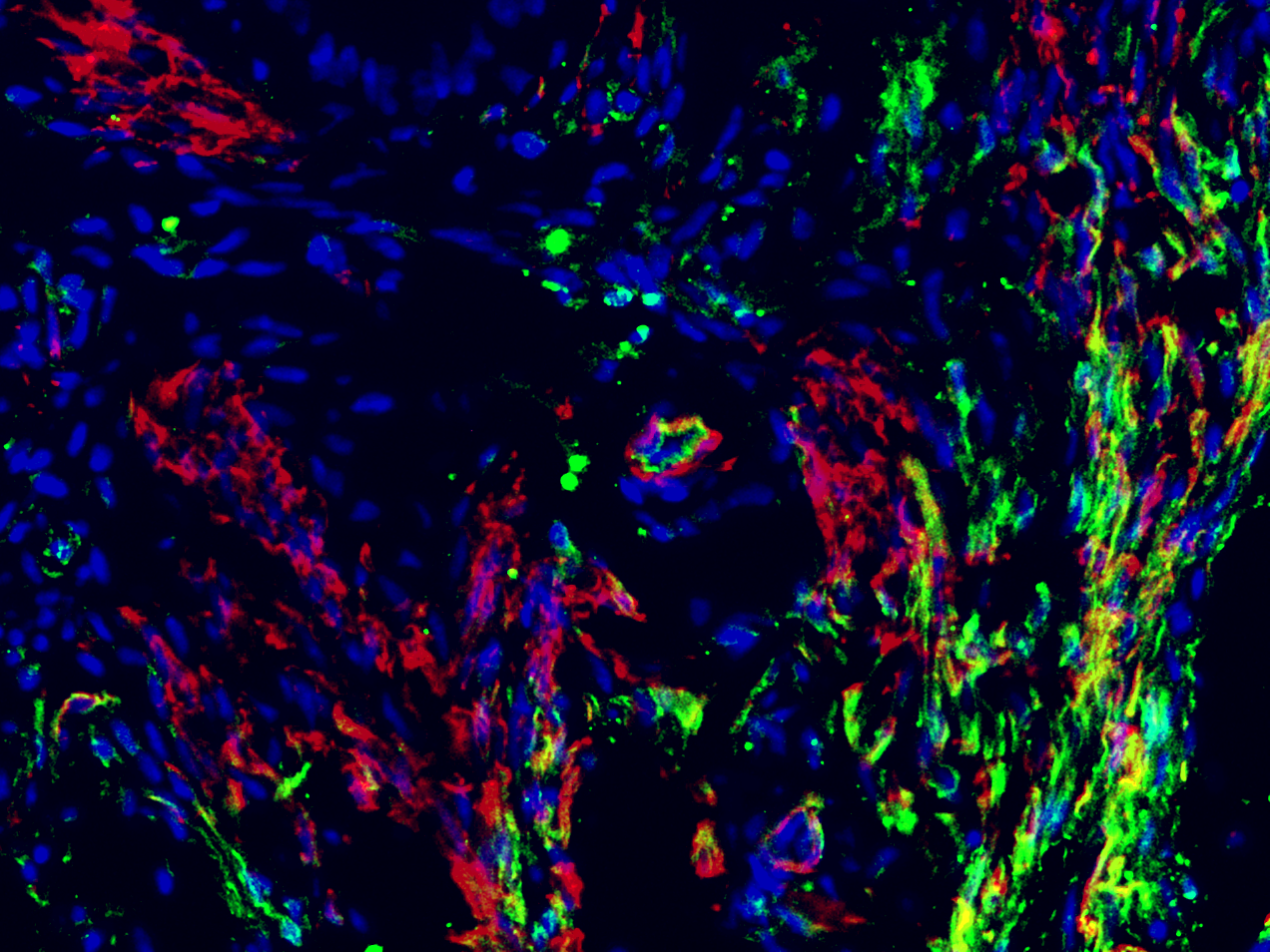

Supplement: Supplementary file 4 — Source Data for Figure 3 [file EMMM-15-e17601-s007.zip › Figure 3-2/3E/Patient AXL-a-SMA.tif]

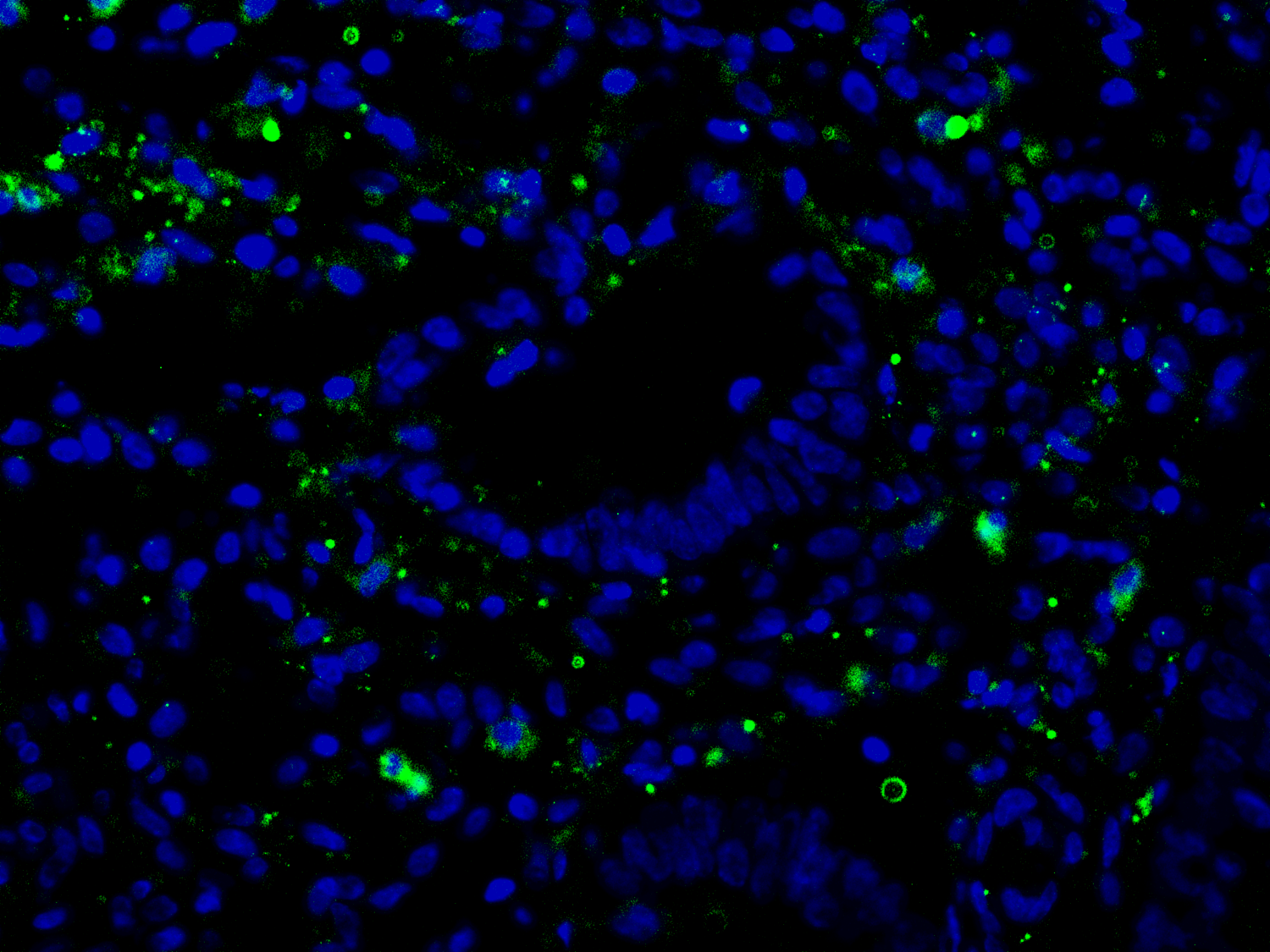

Supplement: Supplementary file 4 — Source Data for Figure 3 [file EMMM-15-e17601-s007.zip › Figure 3-2/3E/Control AXL.tif]

**Gas6**

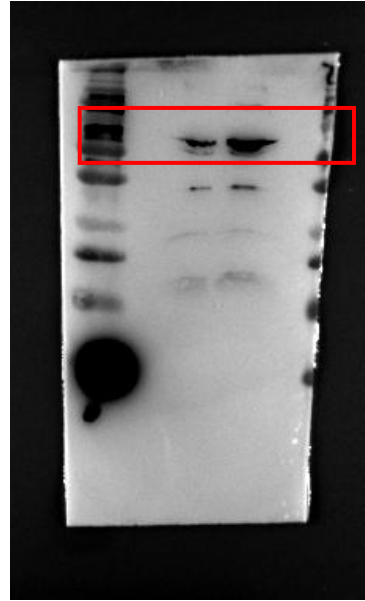

**CCL8**

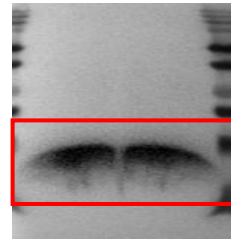

**$\beta$ -actin**

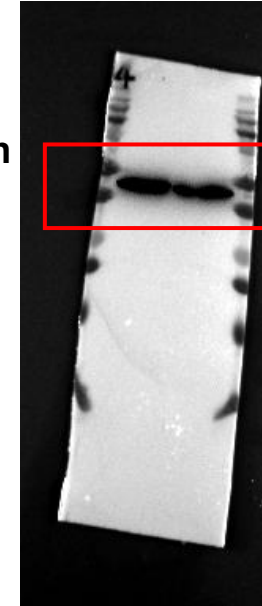

Supplement: Supplementary file 4 — Source Data for Figure 3 [file EMMM-15-e17601-s007.zip › Figure 3-2/3C/3C blot.pdf]

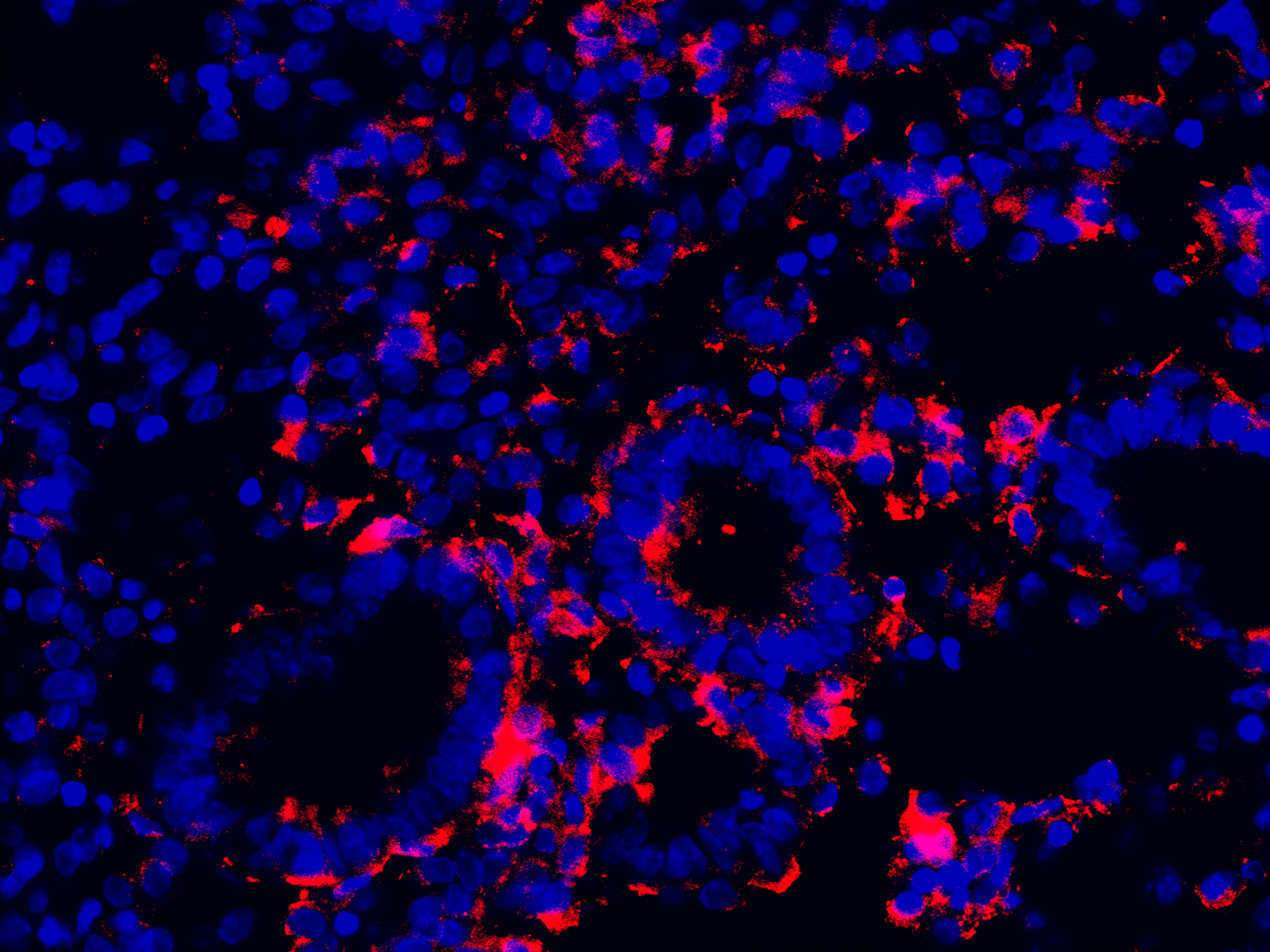

Supplement: Supplementary file 4 — Source Data for Figure 3 [file EMMM-15-e17601-s007.zip › Figure 3-2/3D/3D IF patient GAS6.tif]

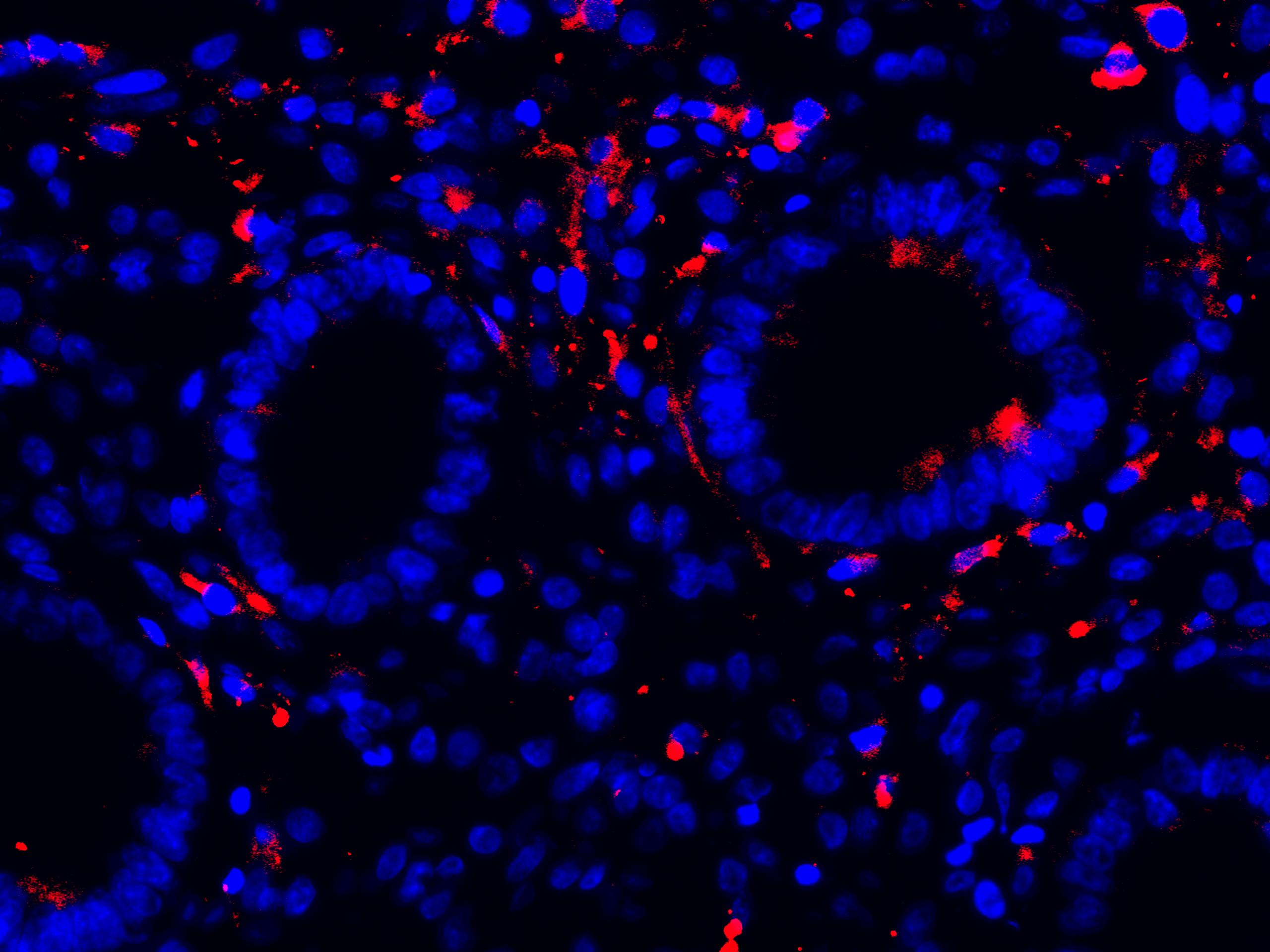

Supplement: Supplementary file 4 — Source Data for Figure 3 [file EMMM-15-e17601-s007.zip › Figure 3-2/3D/3D IF control GAS6.tif]

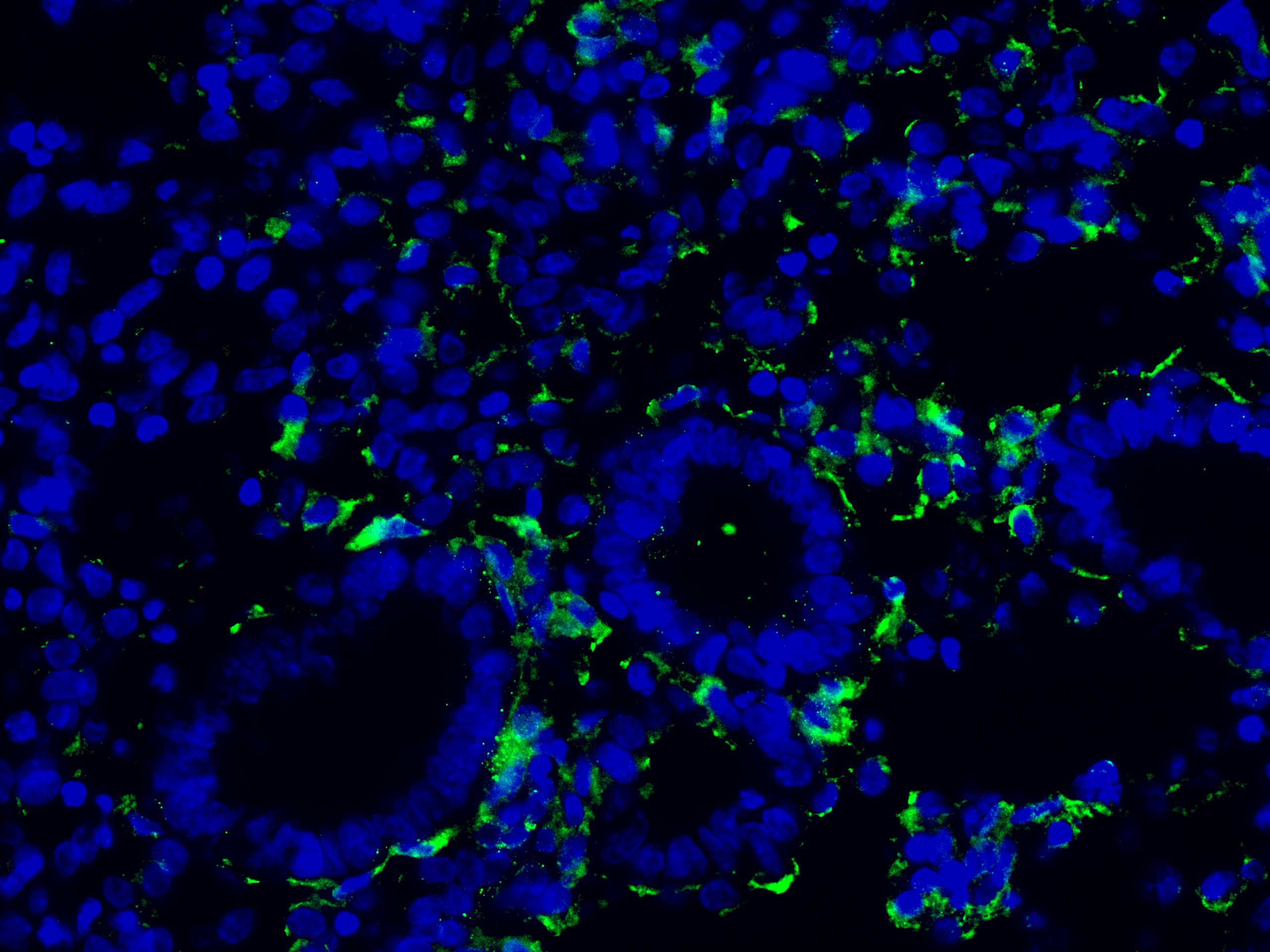

Supplement: Supplementary file 4 — Source Data for Figure 3 [file EMMM-15-e17601-s007.zip › Figure 3-2/3D/3D IF patient CD301.tif]

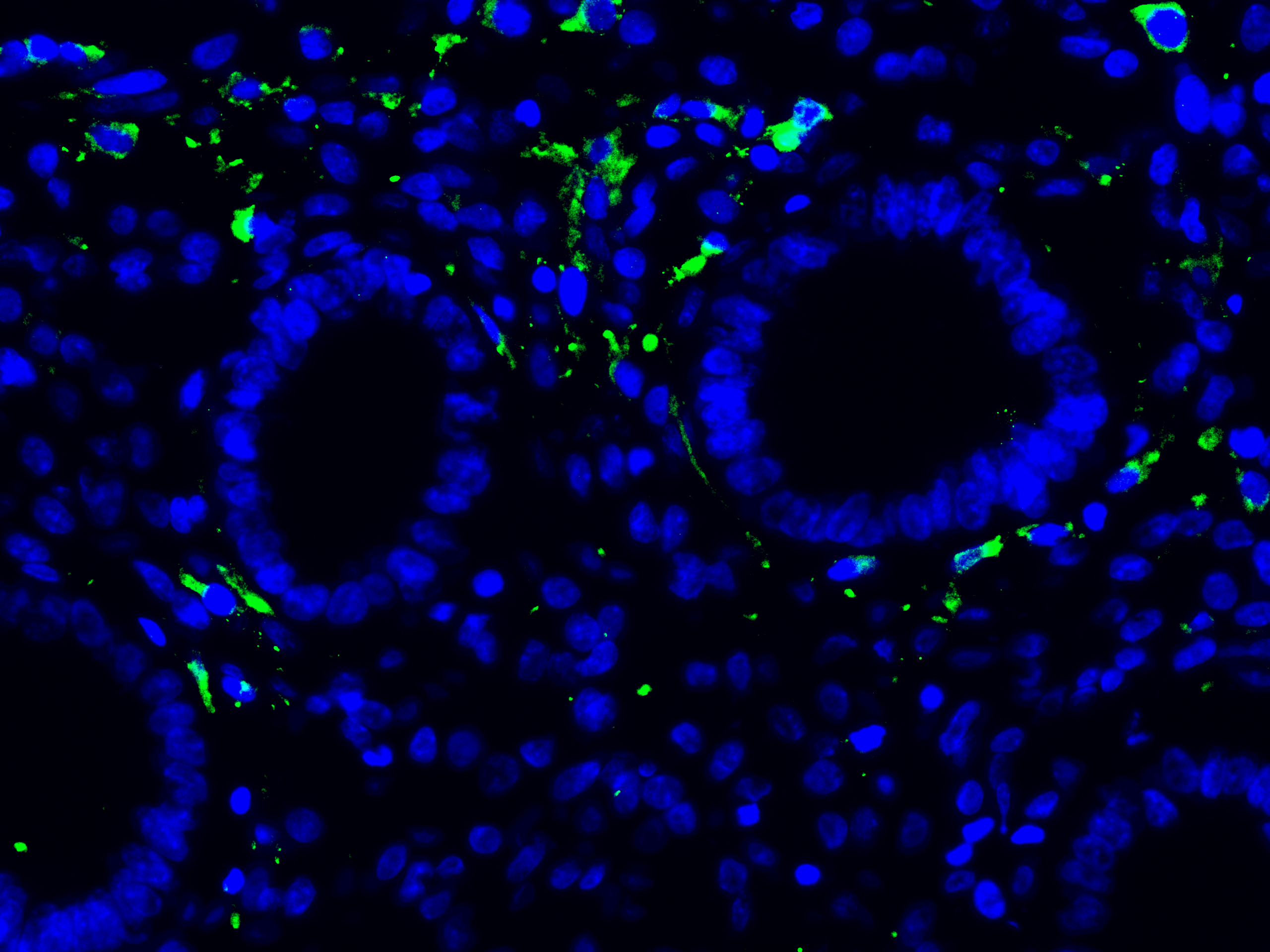

Supplement: Supplementary file 4 — Source Data for Figure 3 [file EMMM-15-e17601-s007.zip › Figure 3-2/3D/3D IF control CD301.tif]

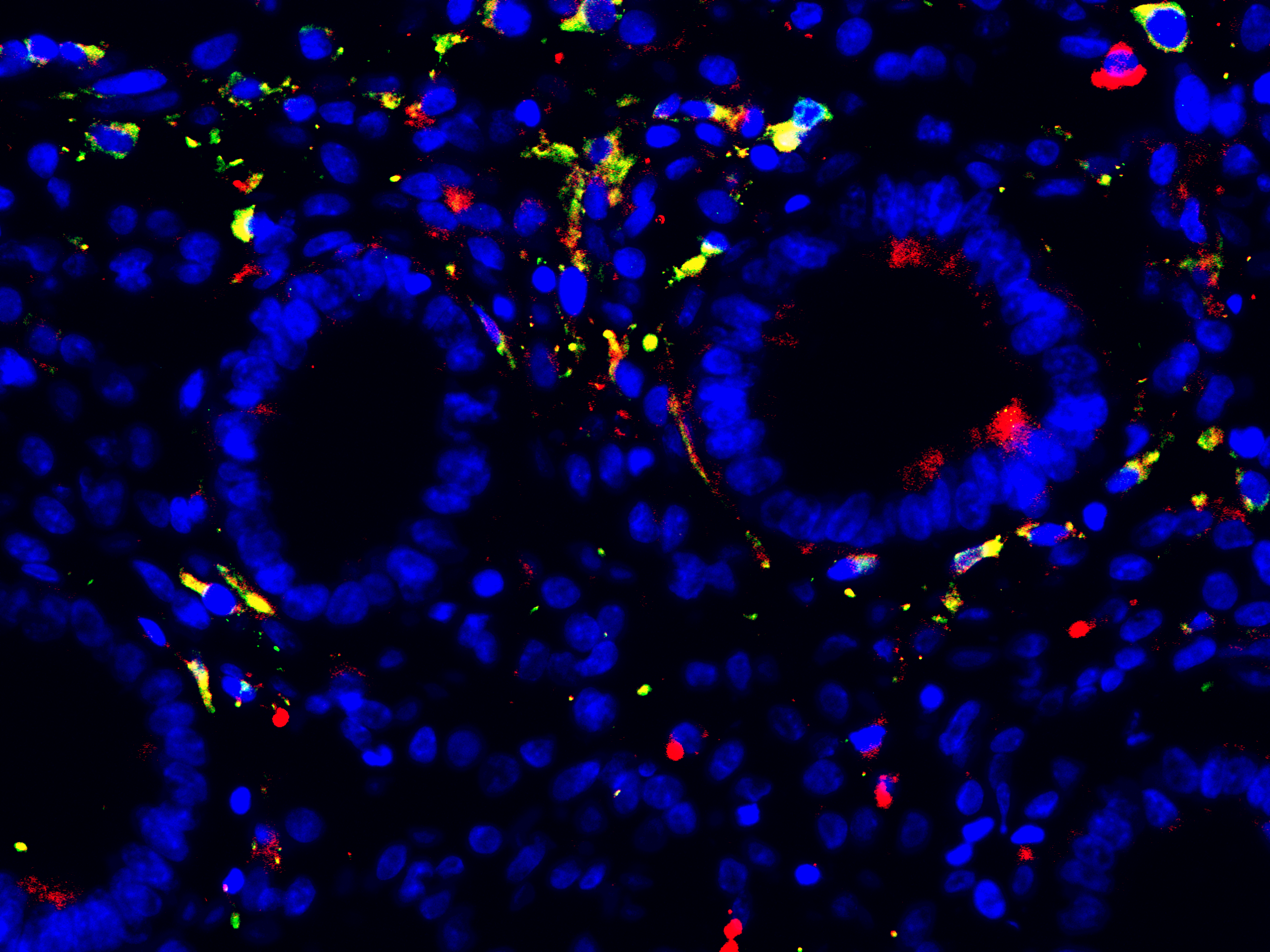

Supplement: Supplementary file 4 — Source Data for Figure 3 [file EMMM-15-e17601-s007.zip › Figure 3-2/3D/3D IF control GAS6-CD301.tif]

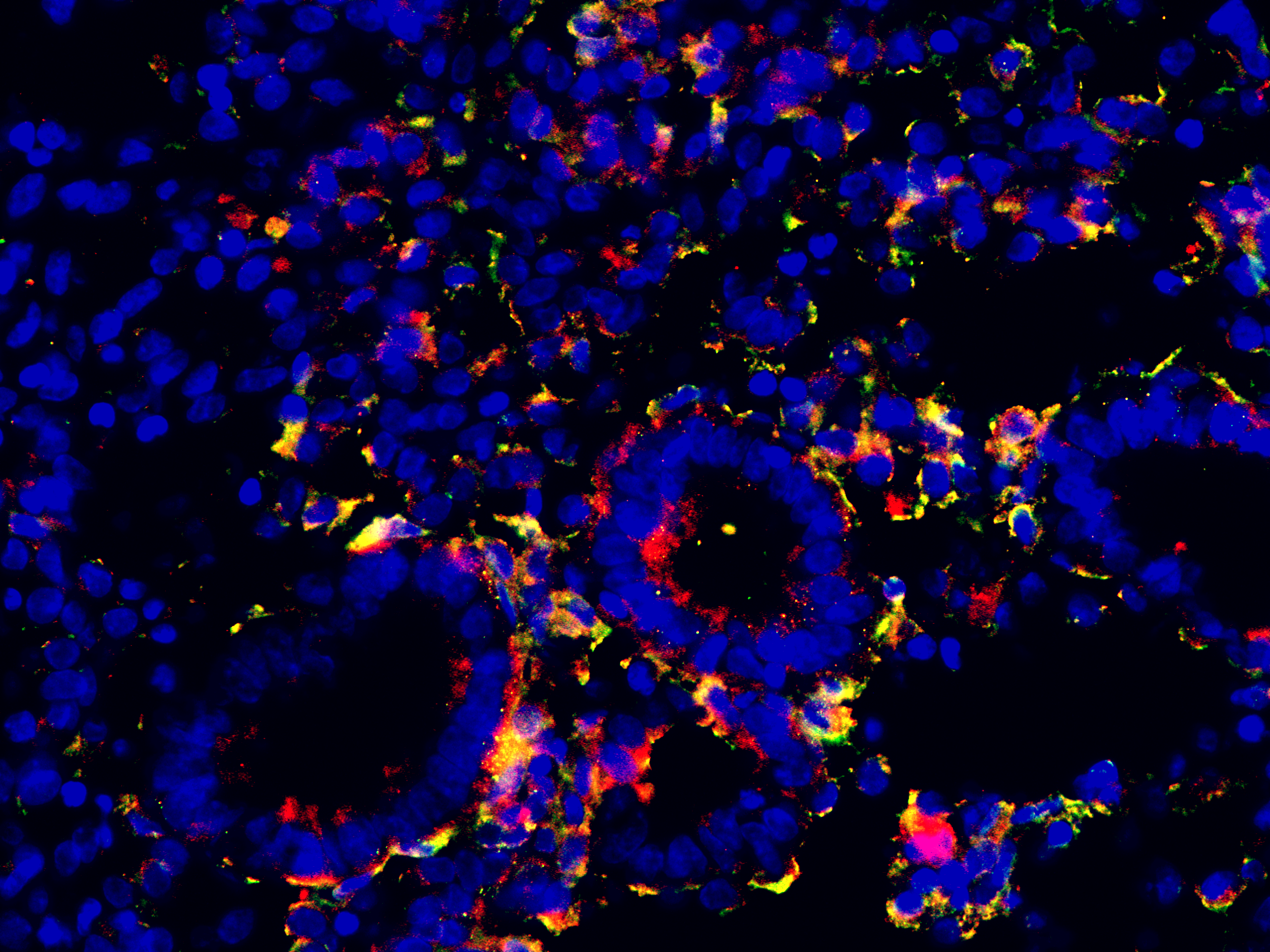

Supplement: Supplementary file 4 — Source Data for Figure 3 [file EMMM-15-e17601-s007.zip › Figure 3-2/3D/3D IF patient GAS6-CD301.tif]

**Collagen1**

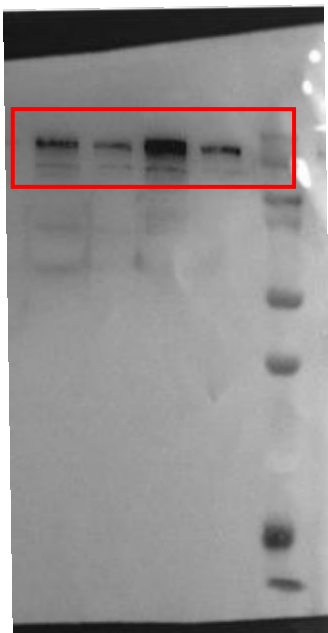

**$\alpha$ -SMA**

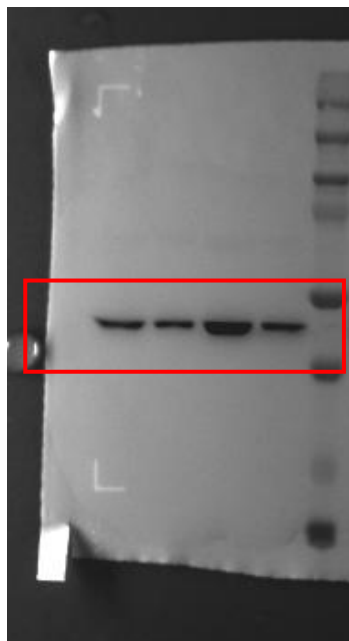

**$\beta$ -actin**

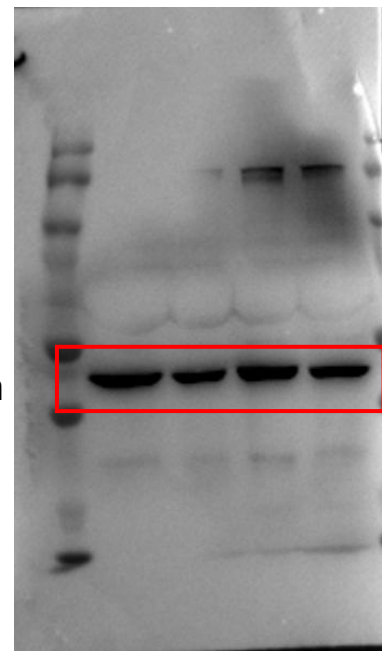

Supplement: Supplementary file 4 — Source Data for Figure 3 [file EMMM-15-e17601-s007.zip › Figure 3-2/3H/3H blot.pdf]

p-Axl

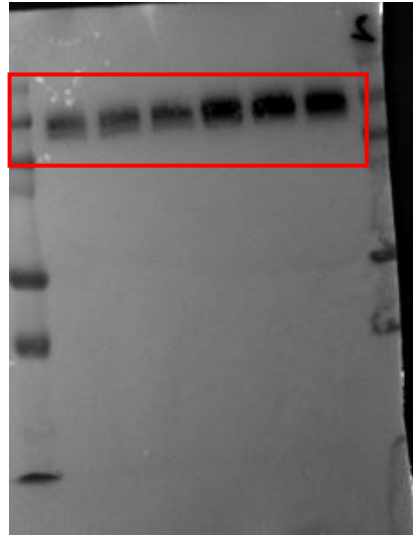

Axl

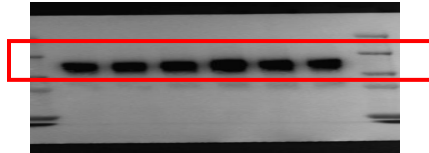

$\beta$ -actin

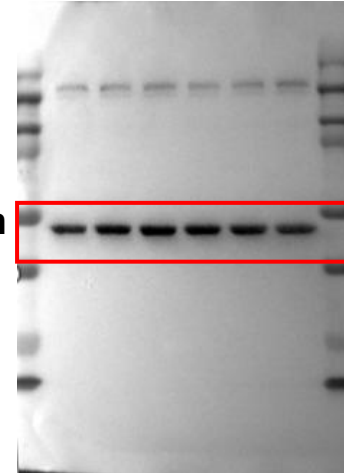

Supplement: Supplementary file 4 — Source Data for Figure 3 [file EMMM-15-e17601-s007.zip › Figure 3-2/3F/3F blot.pdf]

**Collagen1**

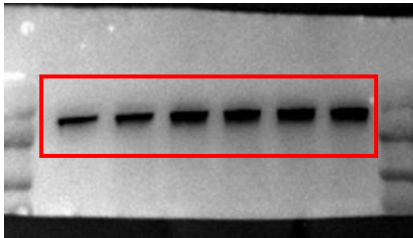

**$\alpha$ -SMA**

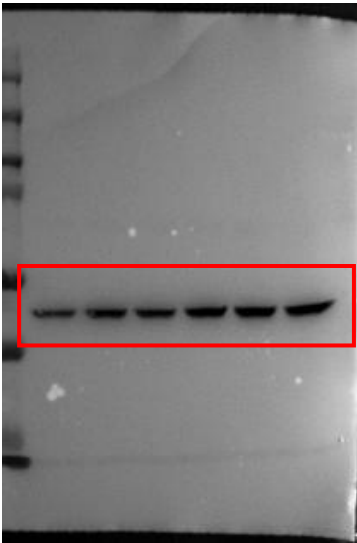

**$\beta$ -actin**

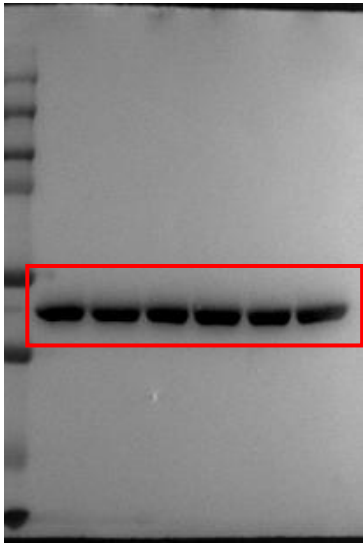

Supplement: Supplementary file 4 — Source Data for Figure 3 [file EMMM-15-e17601-s007.zip › Figure 3-2/3G/3G blot.pdf]

Collagen1

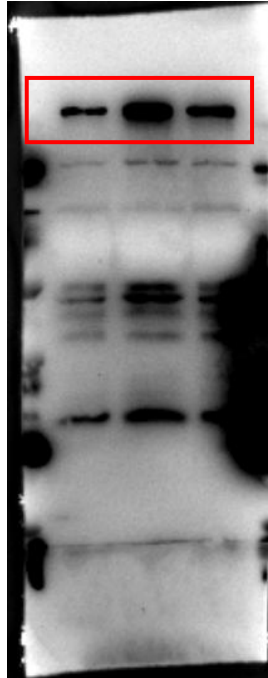

$\alpha$ -SMA

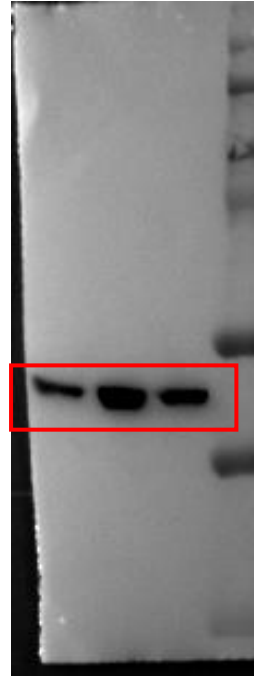

$\beta$ -actin

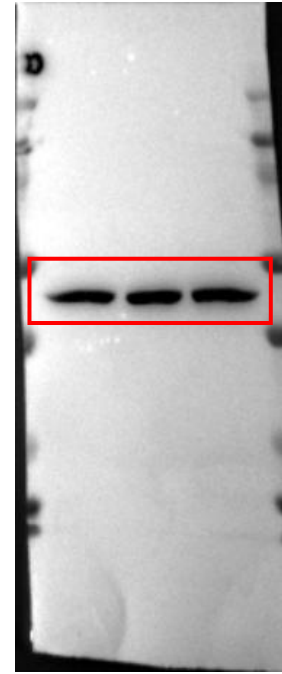

Supplement: Supplementary file 4 — Source Data for Figure 3 [file EMMM-15-e17601-s007.zip › Figure 3-2/3I/3I blot.pdf]

p-p65

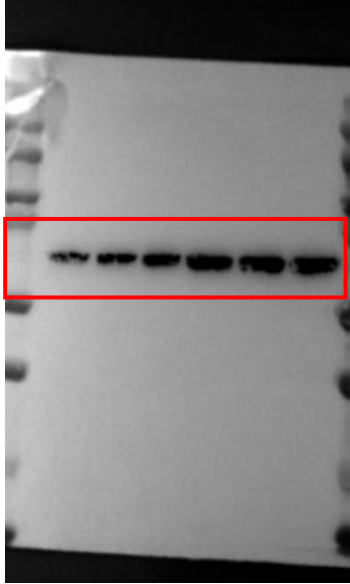

p65

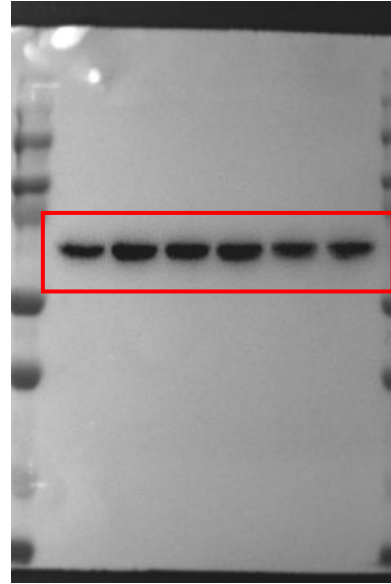

$\beta$ -actin

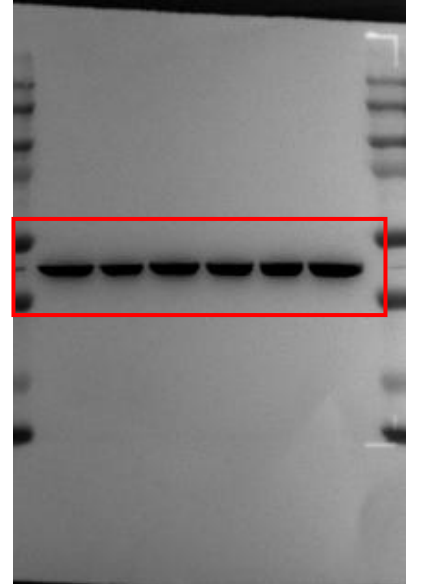

Supplement: Supplementary file 5 — Source Data for Figure 4 [file EMMM-15-e17601-s008.zip › Figure 4-2/4B/4B blot.pdf]

p-p65

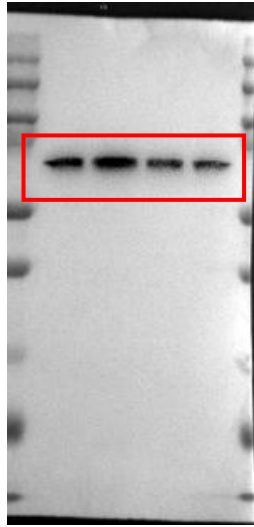

p65

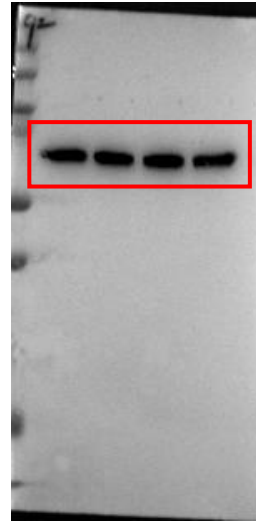

Collagen1

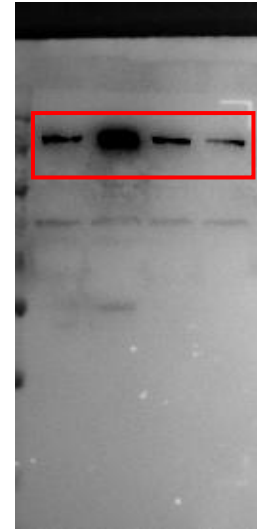

$\alpha$ -SMA

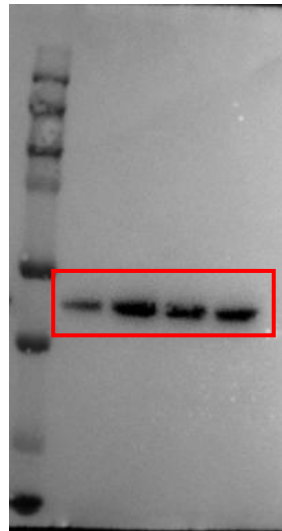

$\beta$ -actin

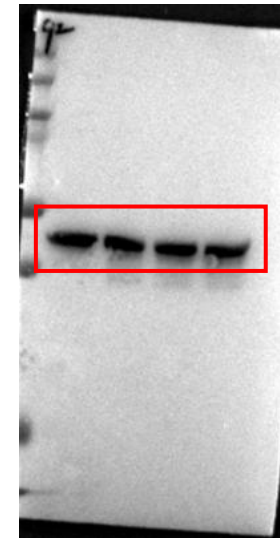

Supplement: Supplementary file 5 — Source Data for Figure 4 [file EMMM-15-e17601-s008.zip › Figure 4-2/4C/4C blot.pdf]

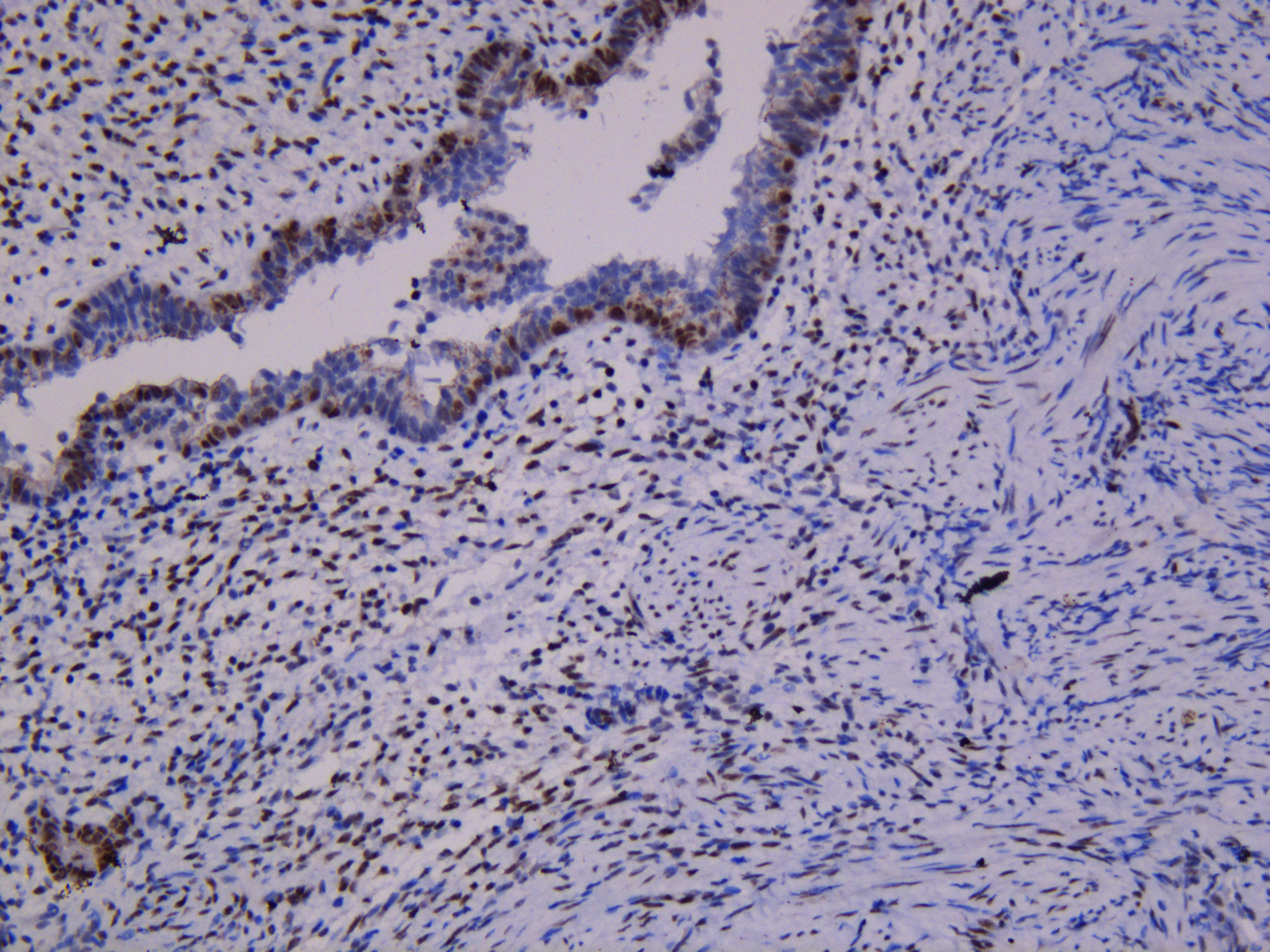

Supplement: Supplementary file 5 — Source Data for Figure 4 [file EMMM-15-e17601-s008.zip › Figure 4-2/4D/4D Patient.tif]

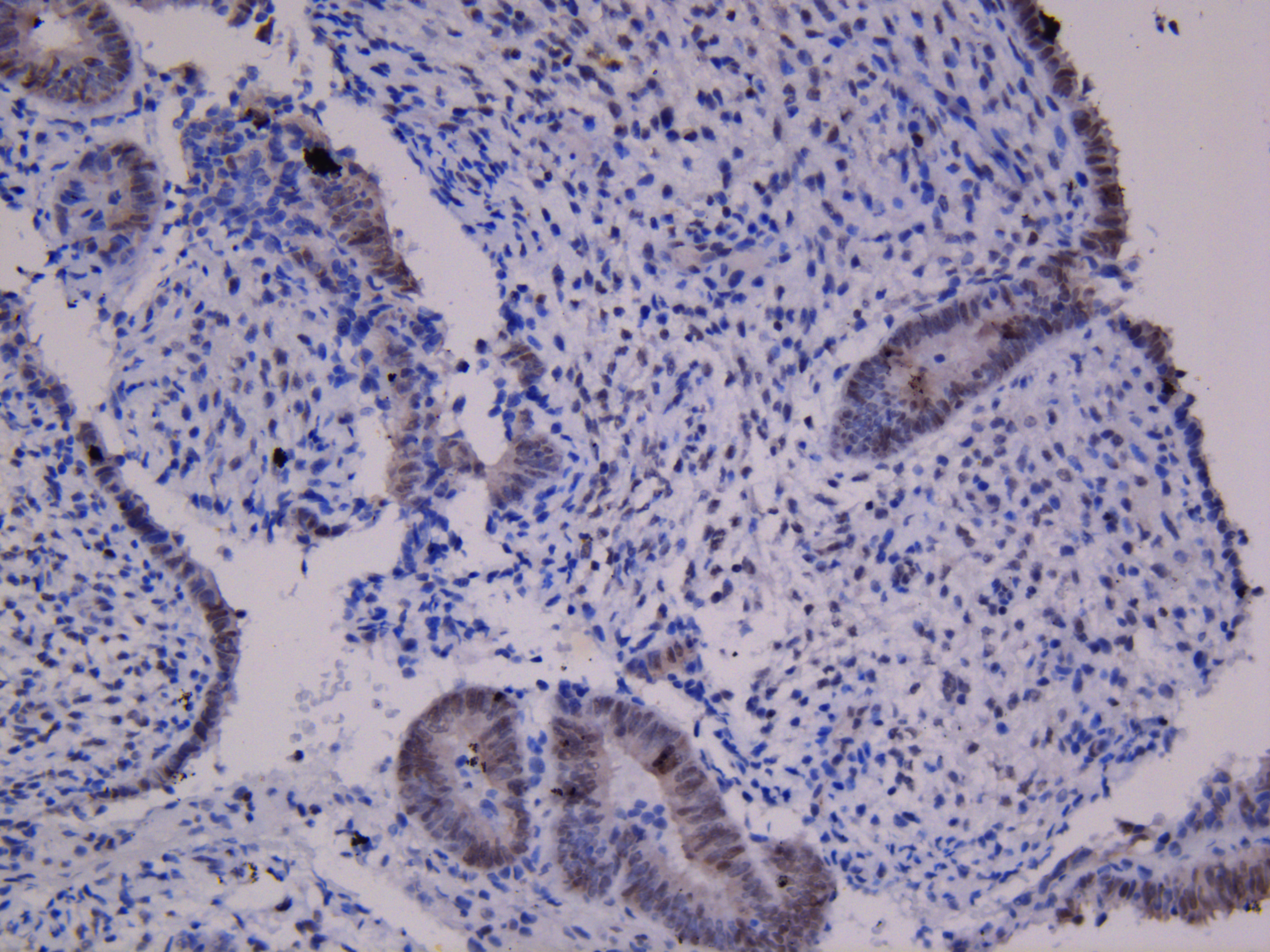

Supplement: Supplementary file 5 — Source Data for Figure 4 [file EMMM-15-e17601-s008.zip › Figure 4-2/4D/4D Control.tif]

**Collagen1**

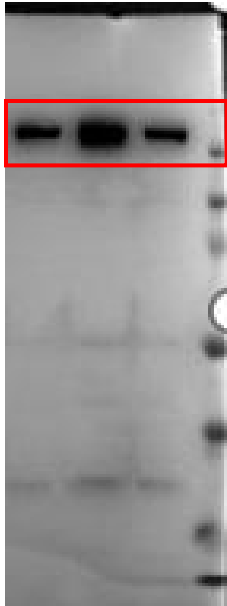

**$\alpha$ -SMA**

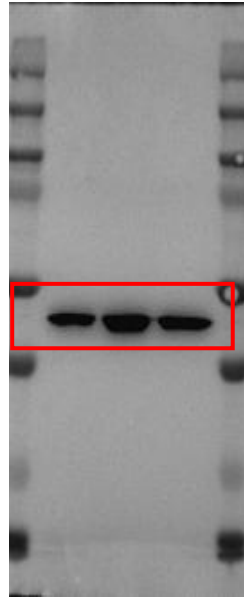

**$\beta$ -actin**

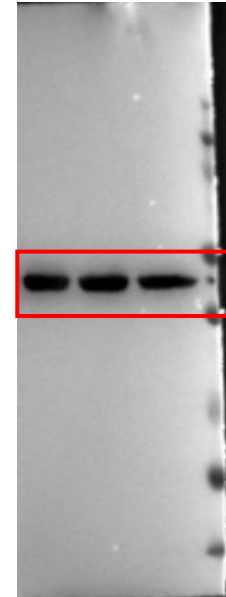

Supplement: Supplementary file 5 — Source Data for Figure 4 [file EMMM-15-e17601-s008.zip › Figure 4-2/4F/4F blot.pdf]

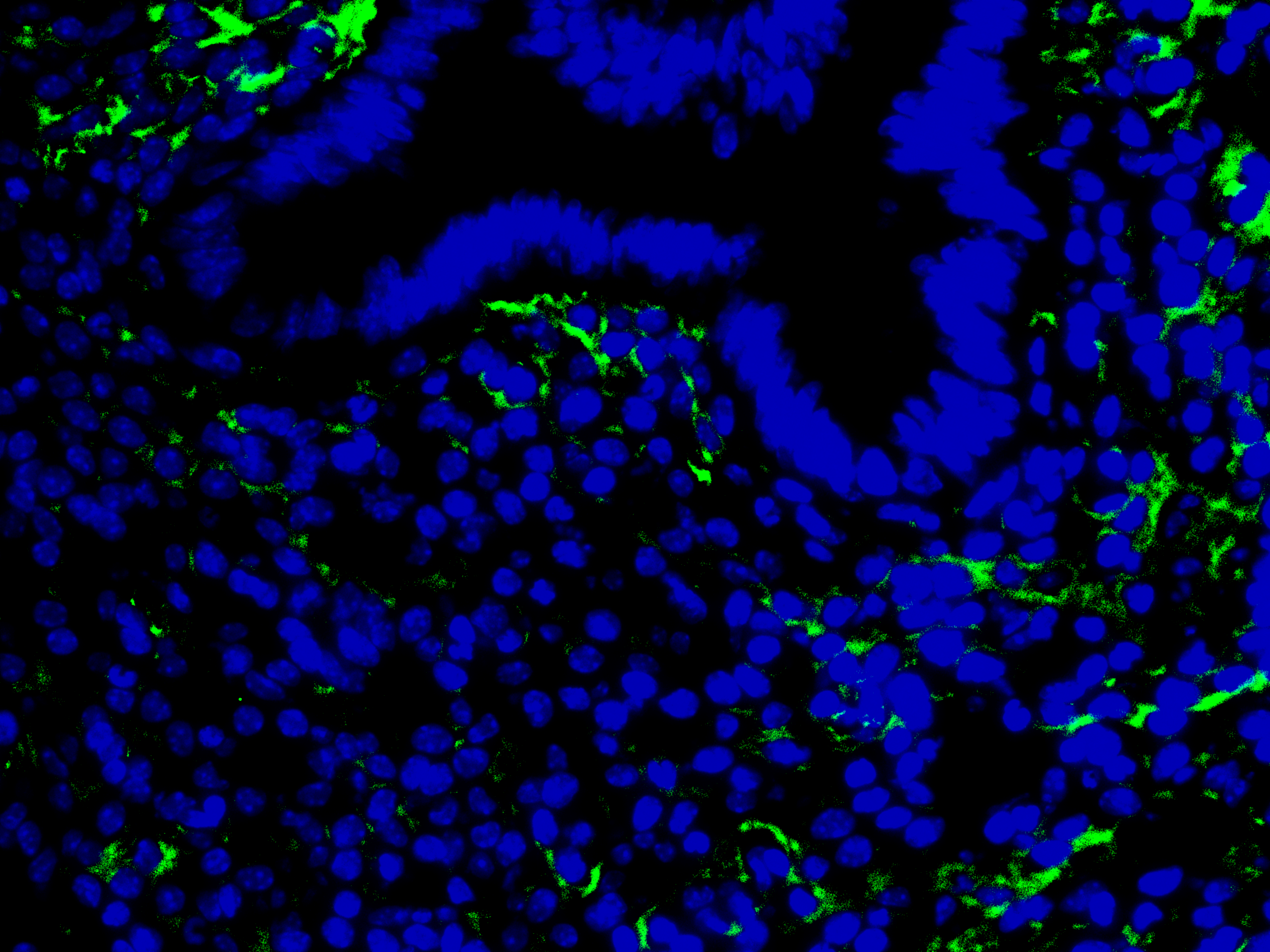

Supplement: Supplementary file 6 — Source Data for Figure 5 [file EMMM-15-e17601-s005.zip › Figure 5-2/5F/5F Sham AXL.tif]

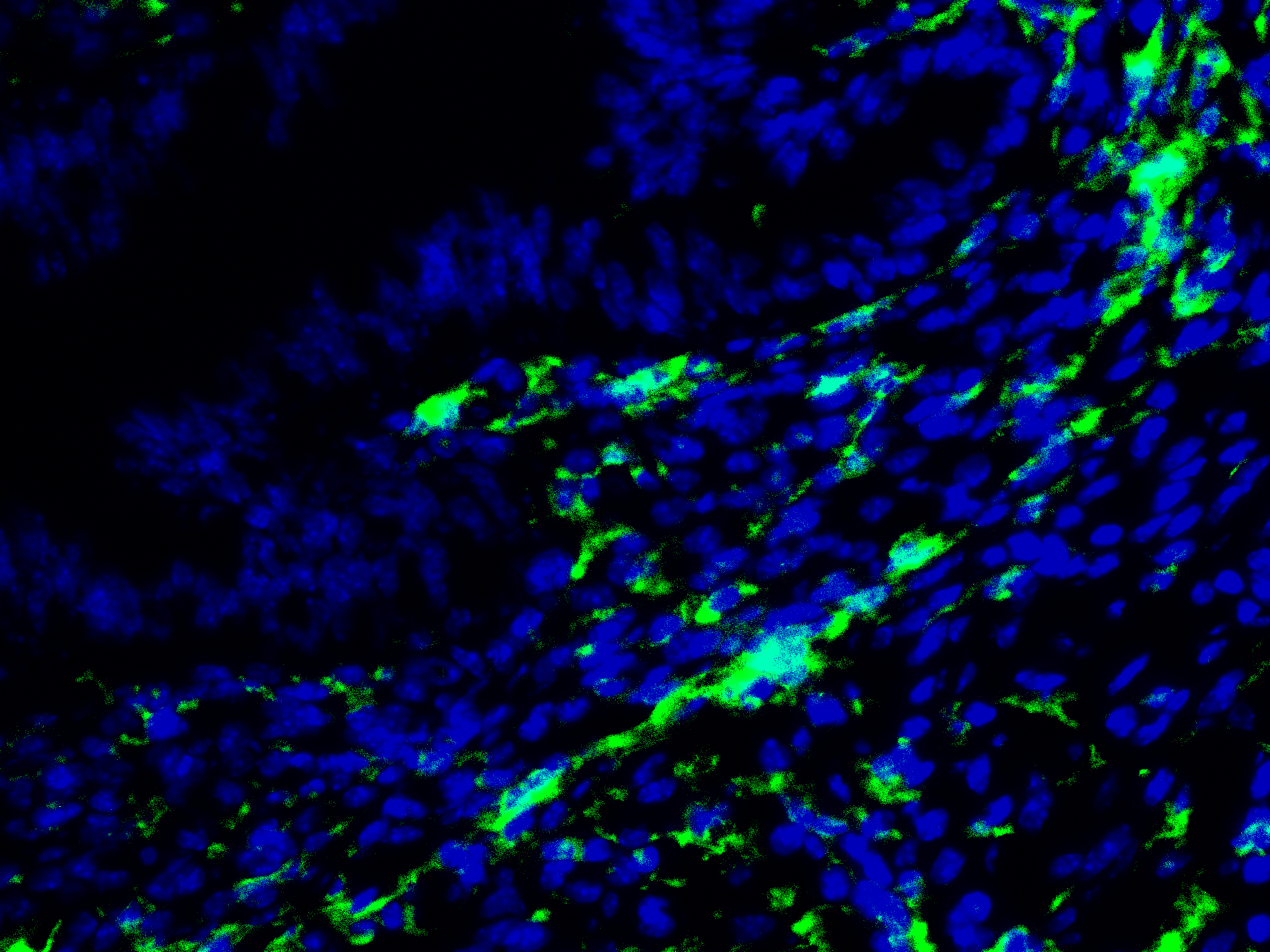

Supplement: Supplementary file 6 — Source Data for Figure 5 [file EMMM-15-e17601-s005.zip › Figure 5-2/5F/5F IUA AXL.tif]

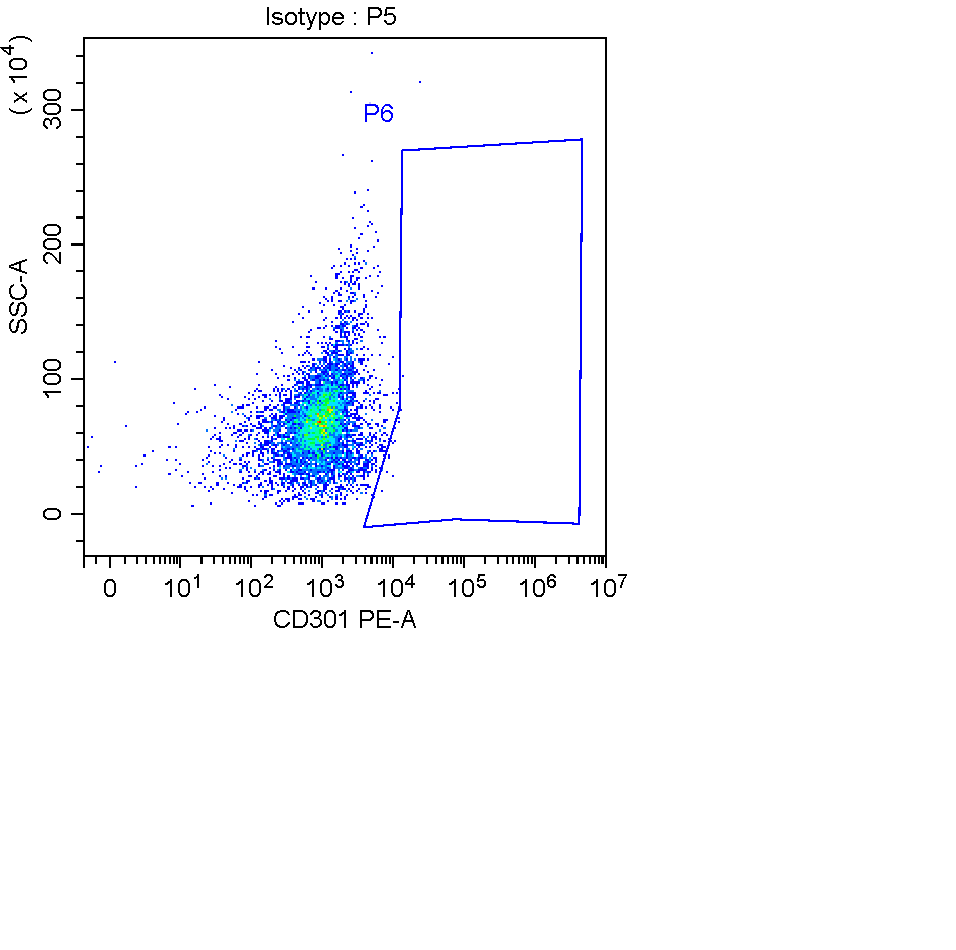

Supplement: Supplementary file 6 — Source Data for Figure 5 [file EMMM-15-e17601-s005.zip › Figure 5-2/5D/Isotype-2.bmp]

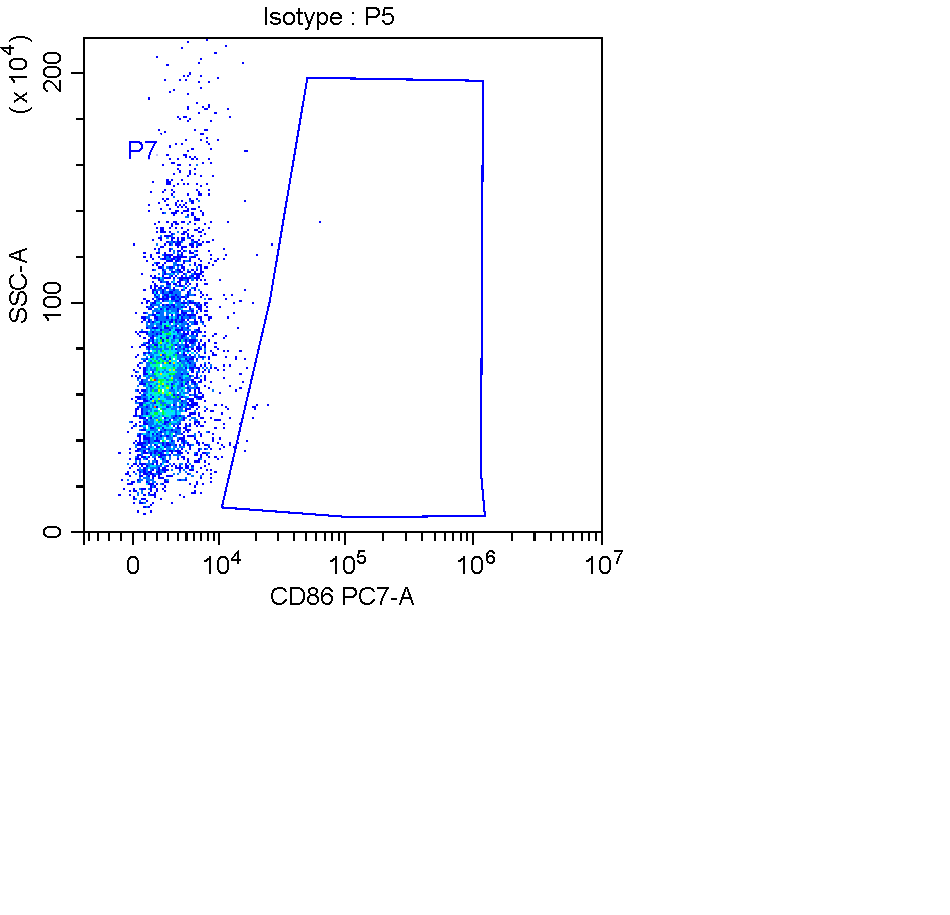

Supplement: Supplementary file 6 — Source Data for Figure 5 [file EMMM-15-e17601-s005.zip › Figure 5-2/5D/Isotype-3.bmp]

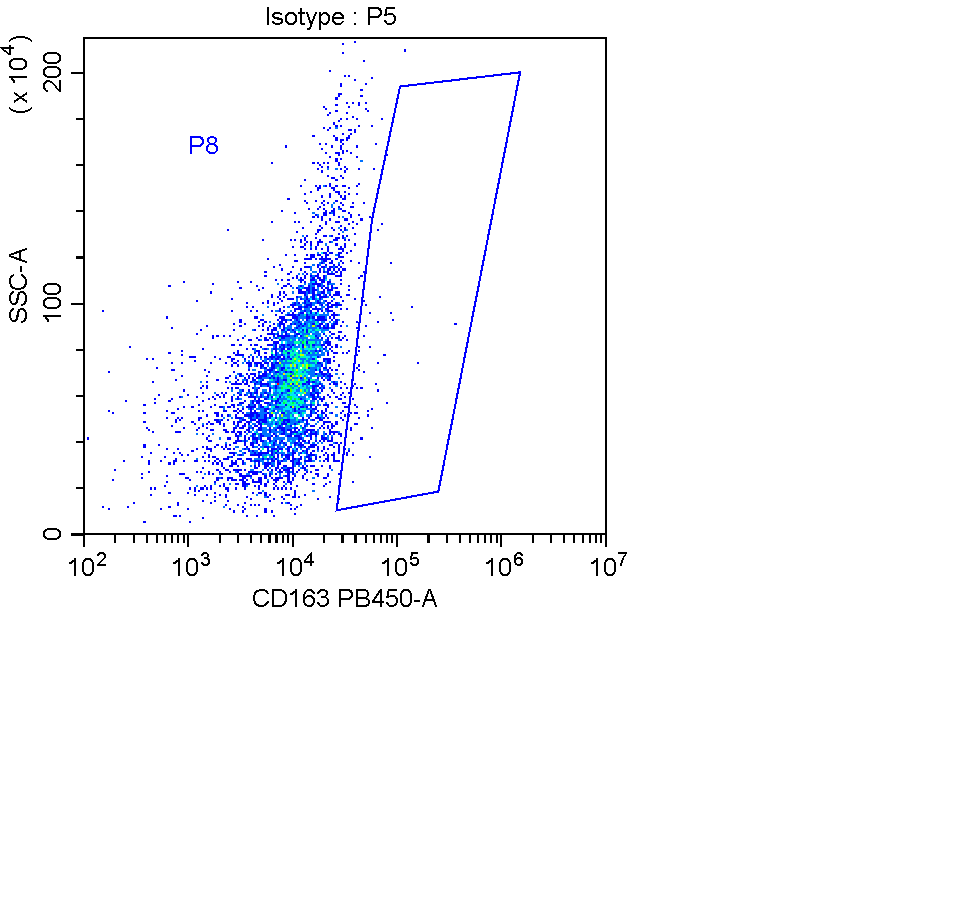

Supplement: Supplementary file 6 — Source Data for Figure 5 [file EMMM-15-e17601-s005.zip › Figure 5-2/5D/Isotype-4.bmp]

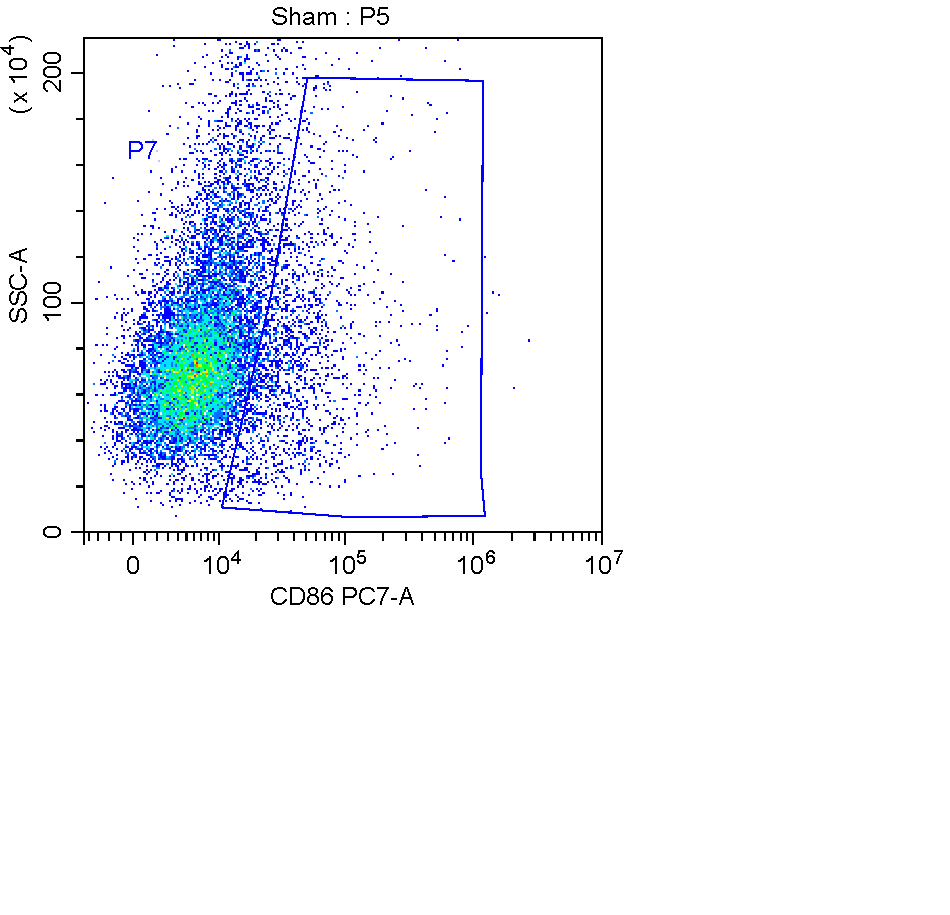

Supplement: Supplementary file 6 — Source Data for Figure 5 [file EMMM-15-e17601-s005.zip › Figure 5-2/5D/Sham-3.bmp]

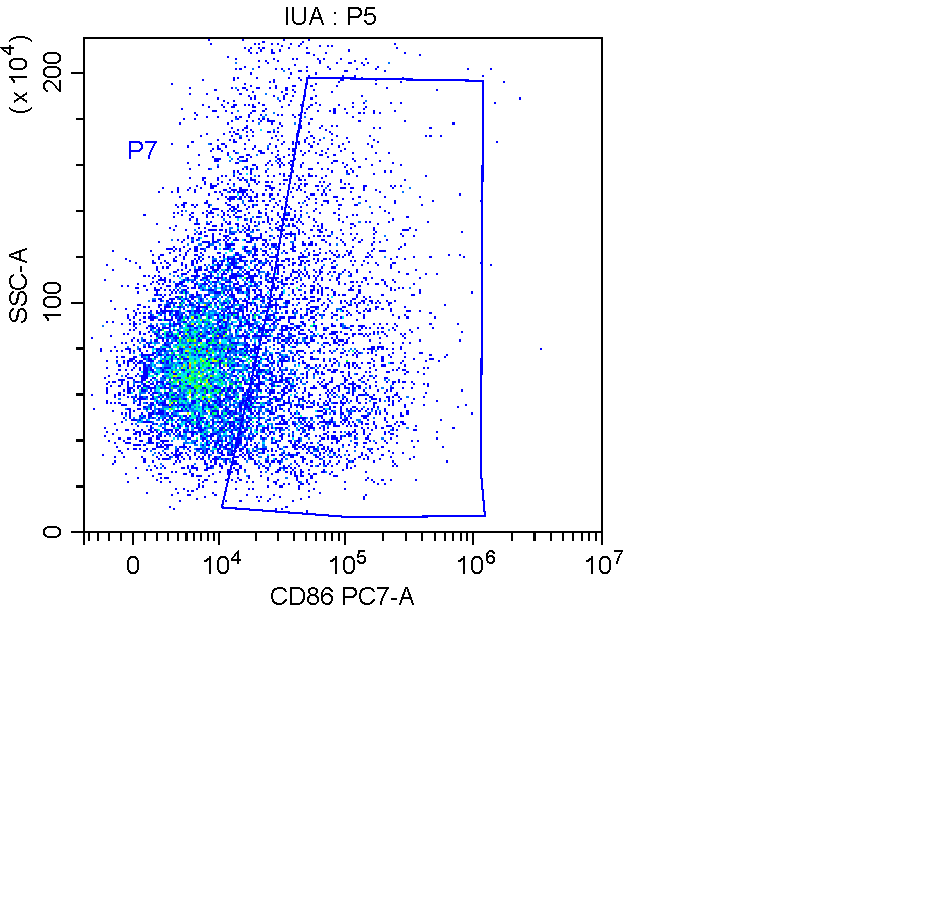

Supplement: Supplementary file 6 — Source Data for Figure 5 [file EMMM-15-e17601-s005.zip › Figure 5-2/5D/IUA-3.bmp]

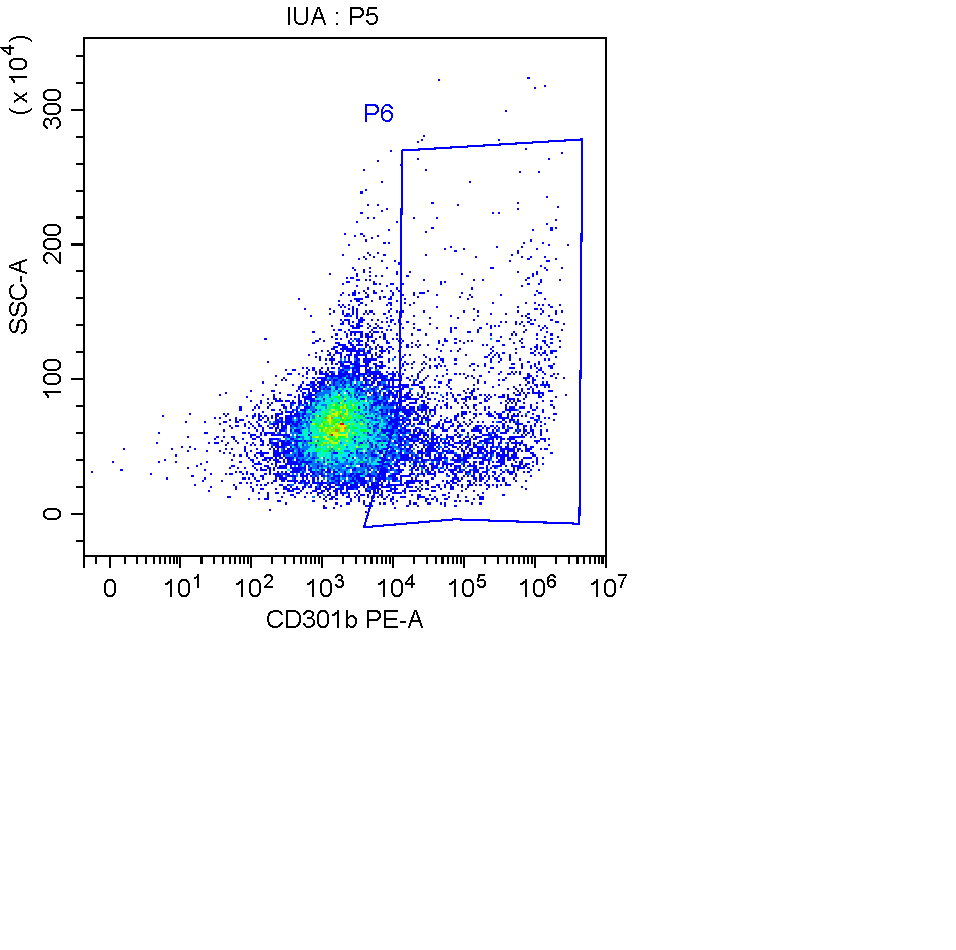

Supplement: Supplementary file 6 — Source Data for Figure 5 [file EMMM-15-e17601-s005.zip › Figure 5-2/5D/IUA-2.bmp]

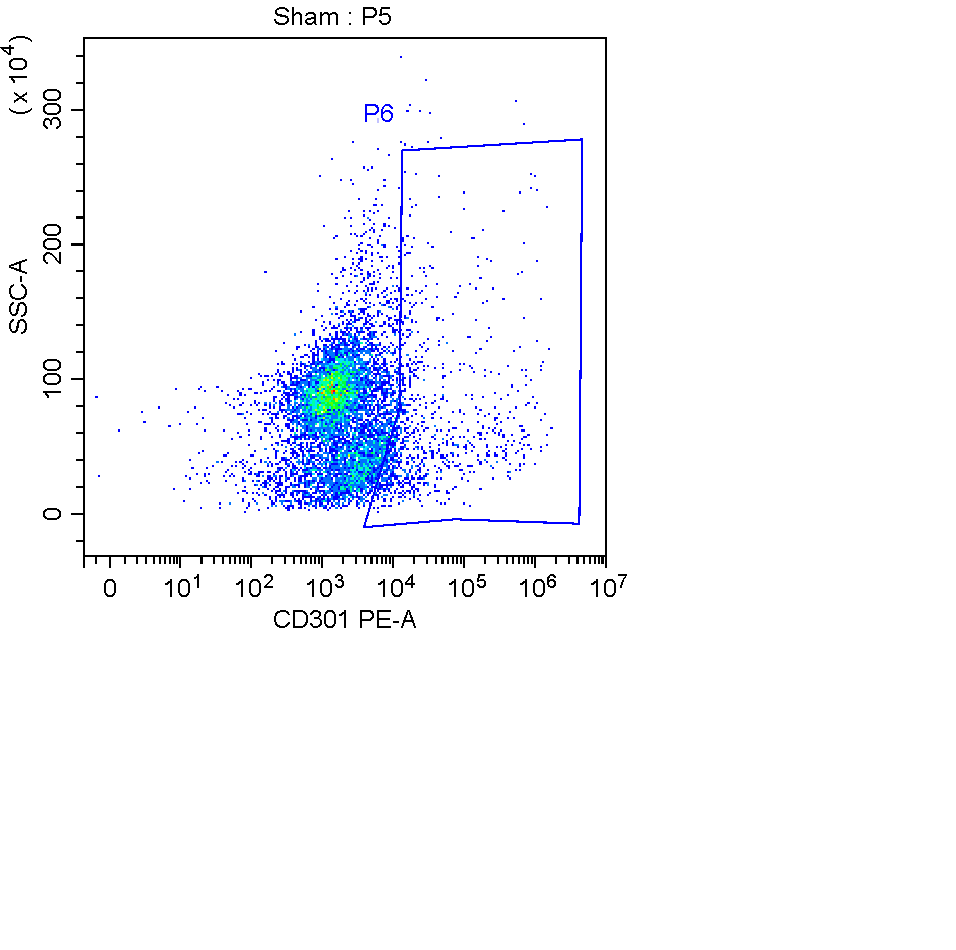

Supplement: Supplementary file 6 — Source Data for Figure 5 [file EMMM-15-e17601-s005.zip › Figure 5-2/5D/Sham-2.bmp]

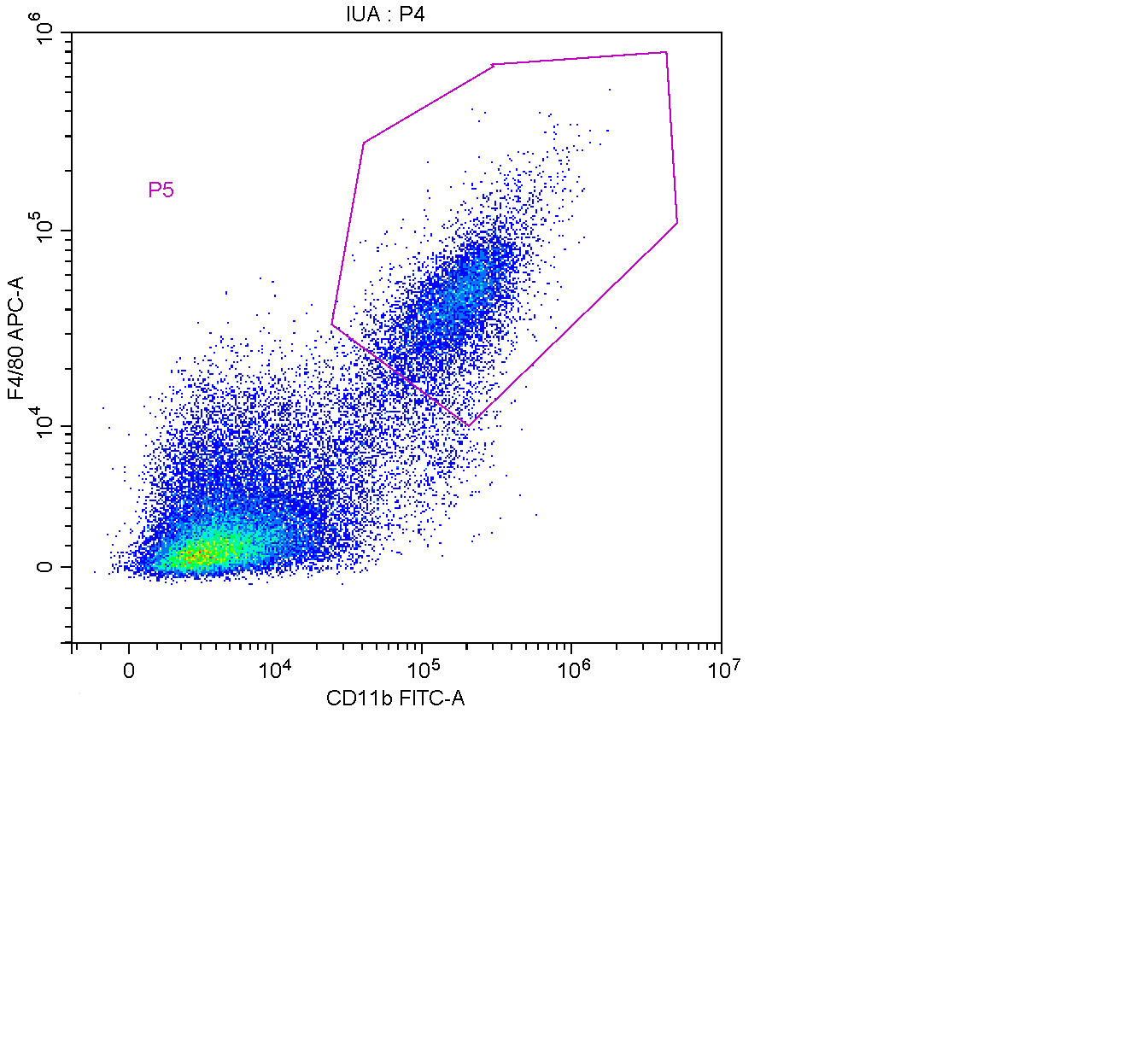

Supplement: Supplementary file 6 — Source Data for Figure 5 [file EMMM-15-e17601-s005.zip › Figure 5-2/5D/IUA-1.bmp]

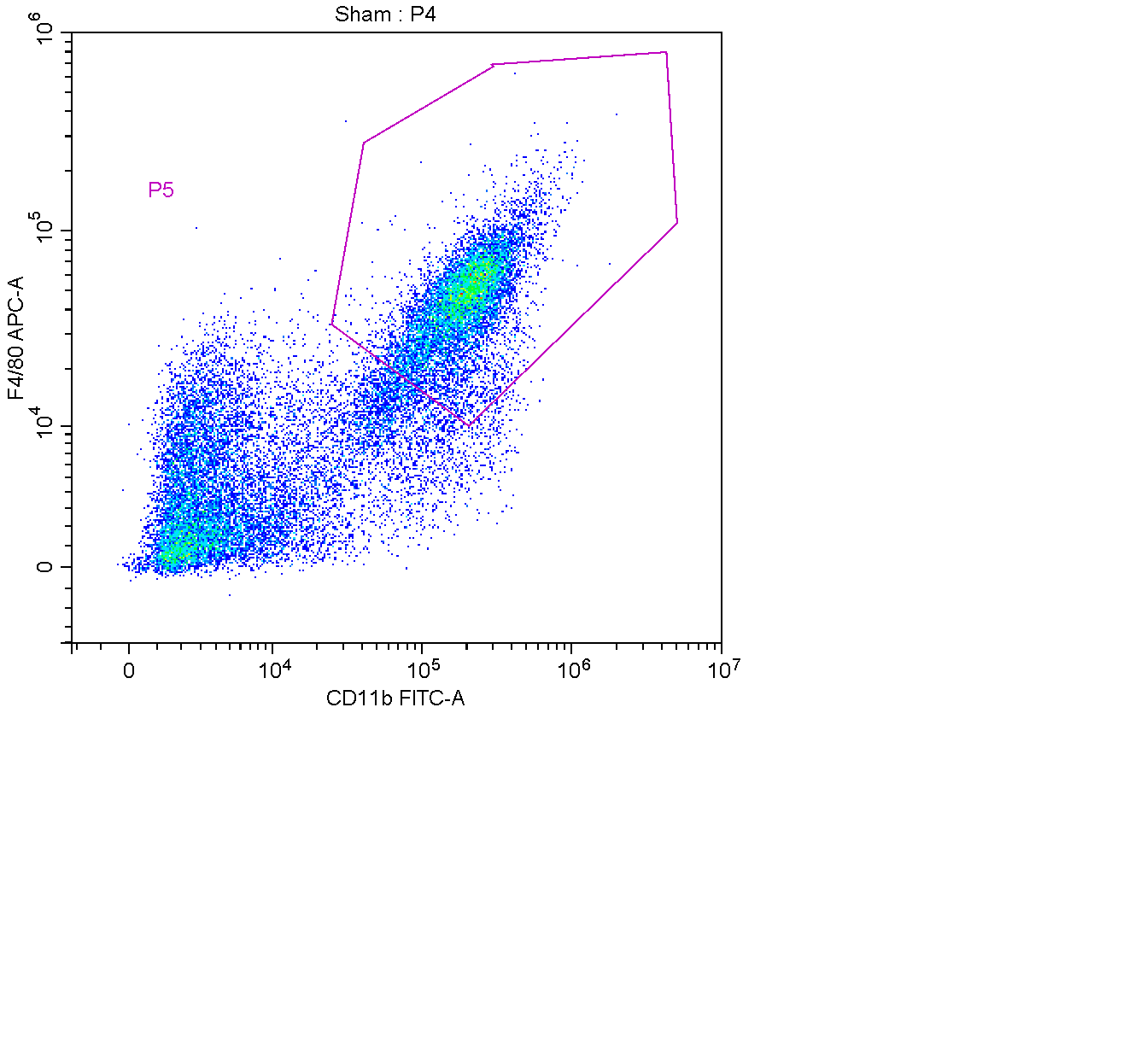

Supplement: Supplementary file 6 — Source Data for Figure 5 [file EMMM-15-e17601-s005.zip › Figure 5-2/5D/Sham-1.bmp]

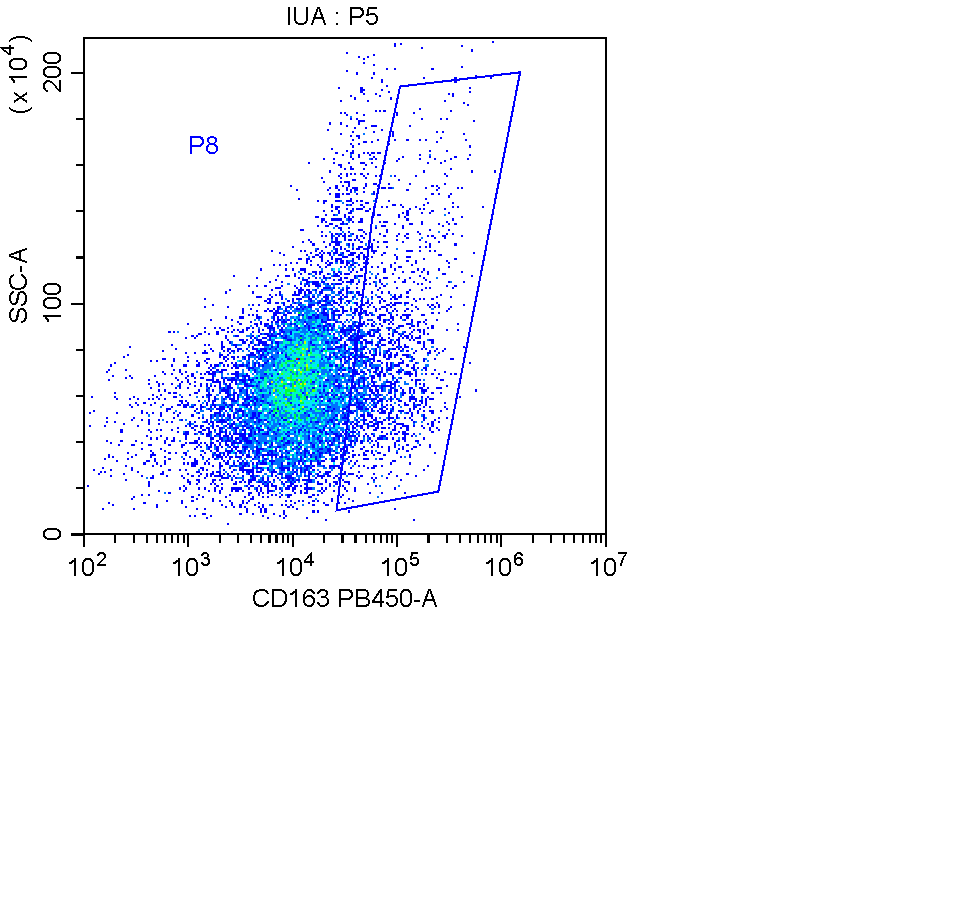

Supplement: Supplementary file 6 — Source Data for Figure 5 [file EMMM-15-e17601-s005.zip › Figure 5-2/5D/IUA-4.bmp]

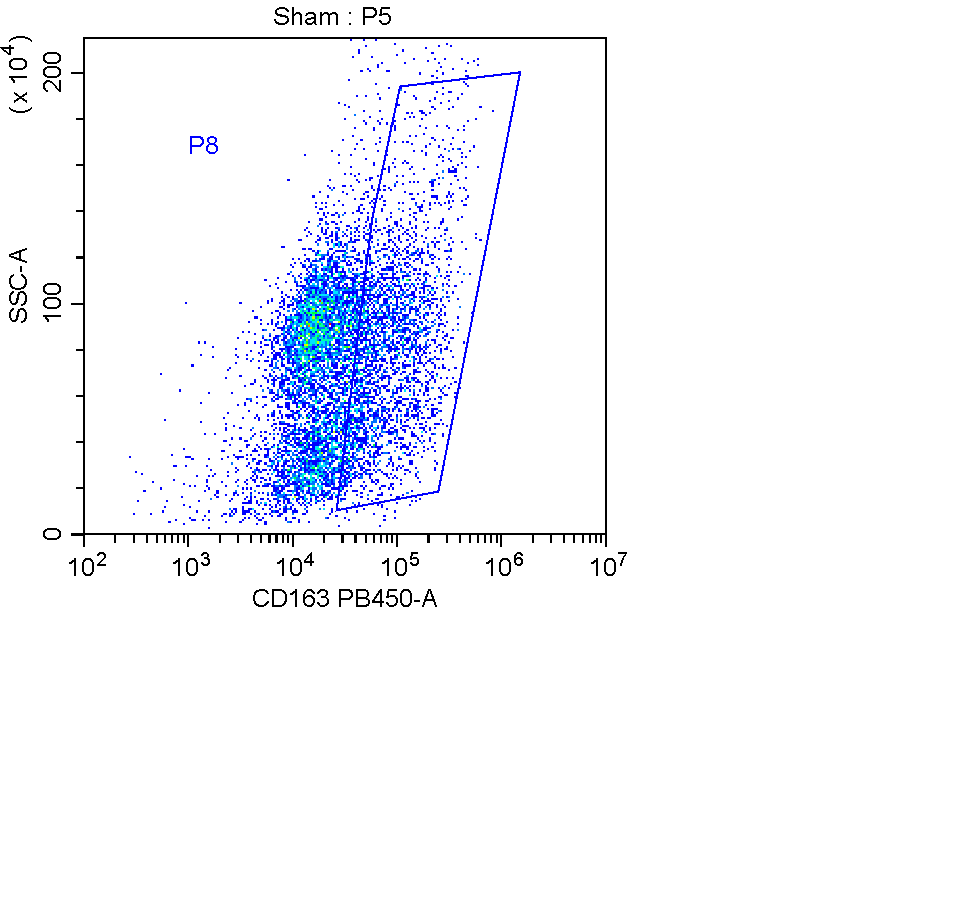

Supplement: Supplementary file 6 — Source Data for Figure 5 [file EMMM-15-e17601-s005.zip › Figure 5-2/5D/Sham-4.bmp]

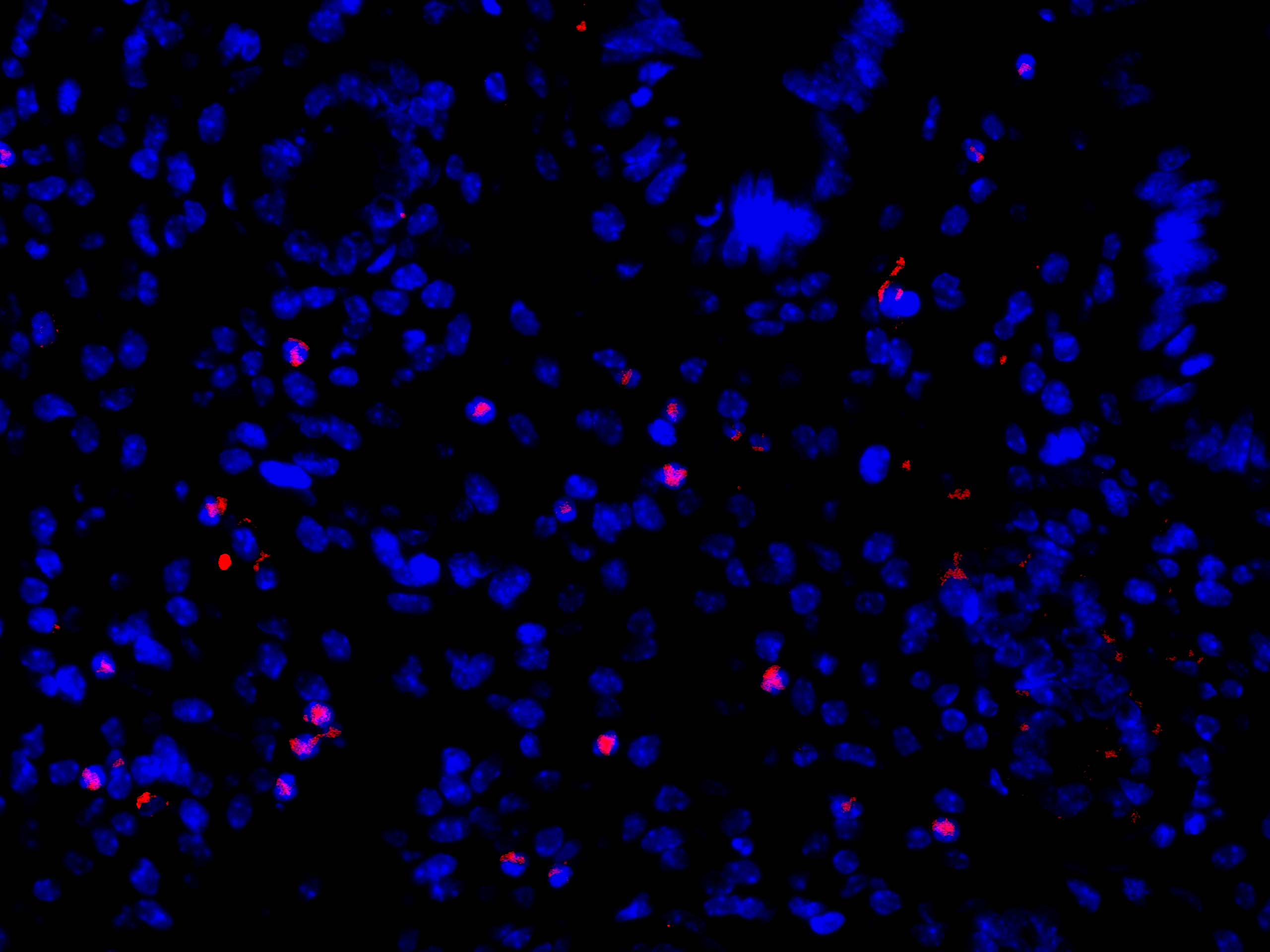

Supplement: Supplementary file 6 — Source Data for Figure 5 [file EMMM-15-e17601-s005.zip › Figure 5-2/5E/Sham GAS6.tif]

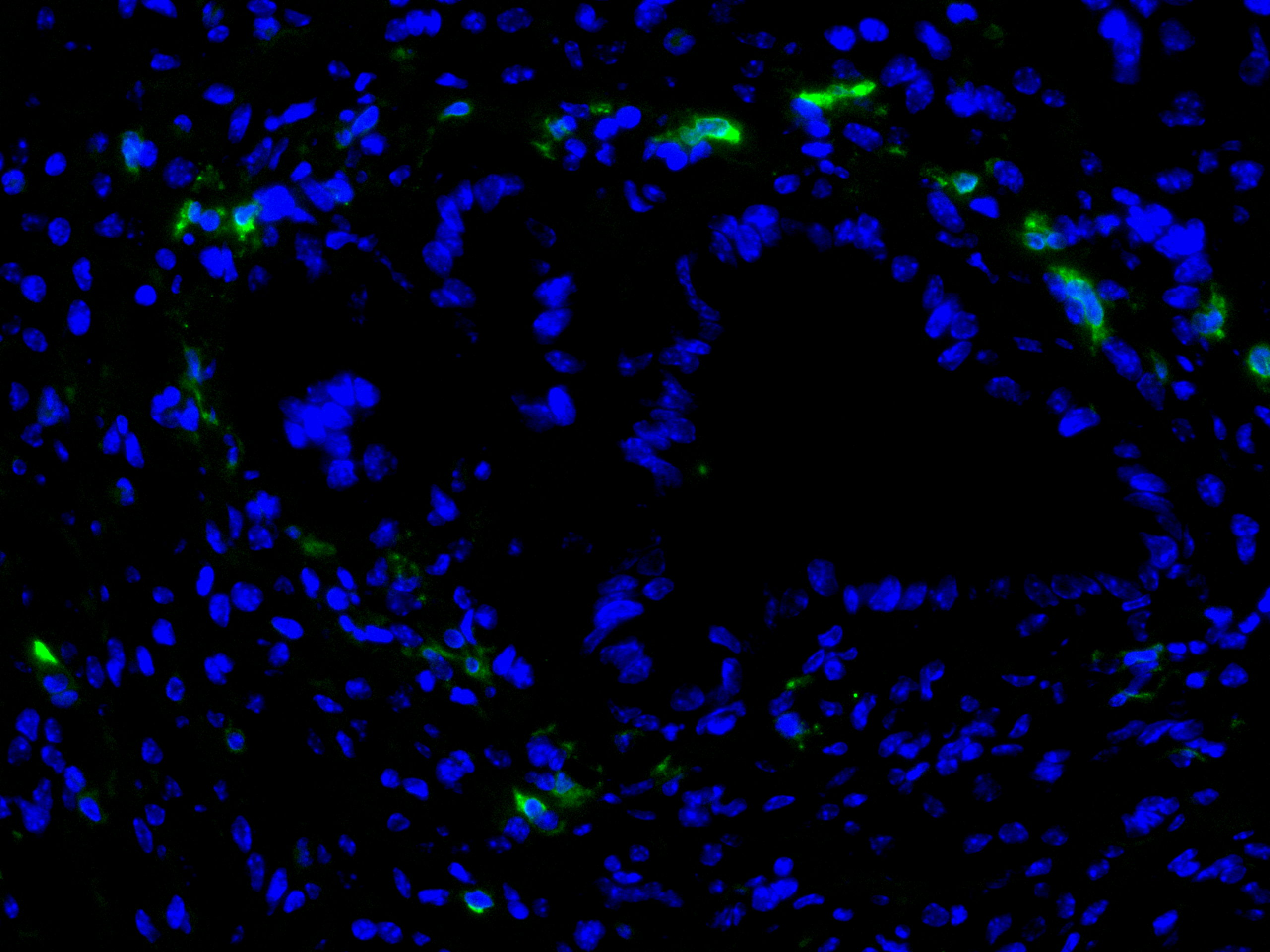

Supplement: Supplementary file 6 — Source Data for Figure 5 [file EMMM-15-e17601-s005.zip › Figure 5-2/5E/IUA CD301b.tif]

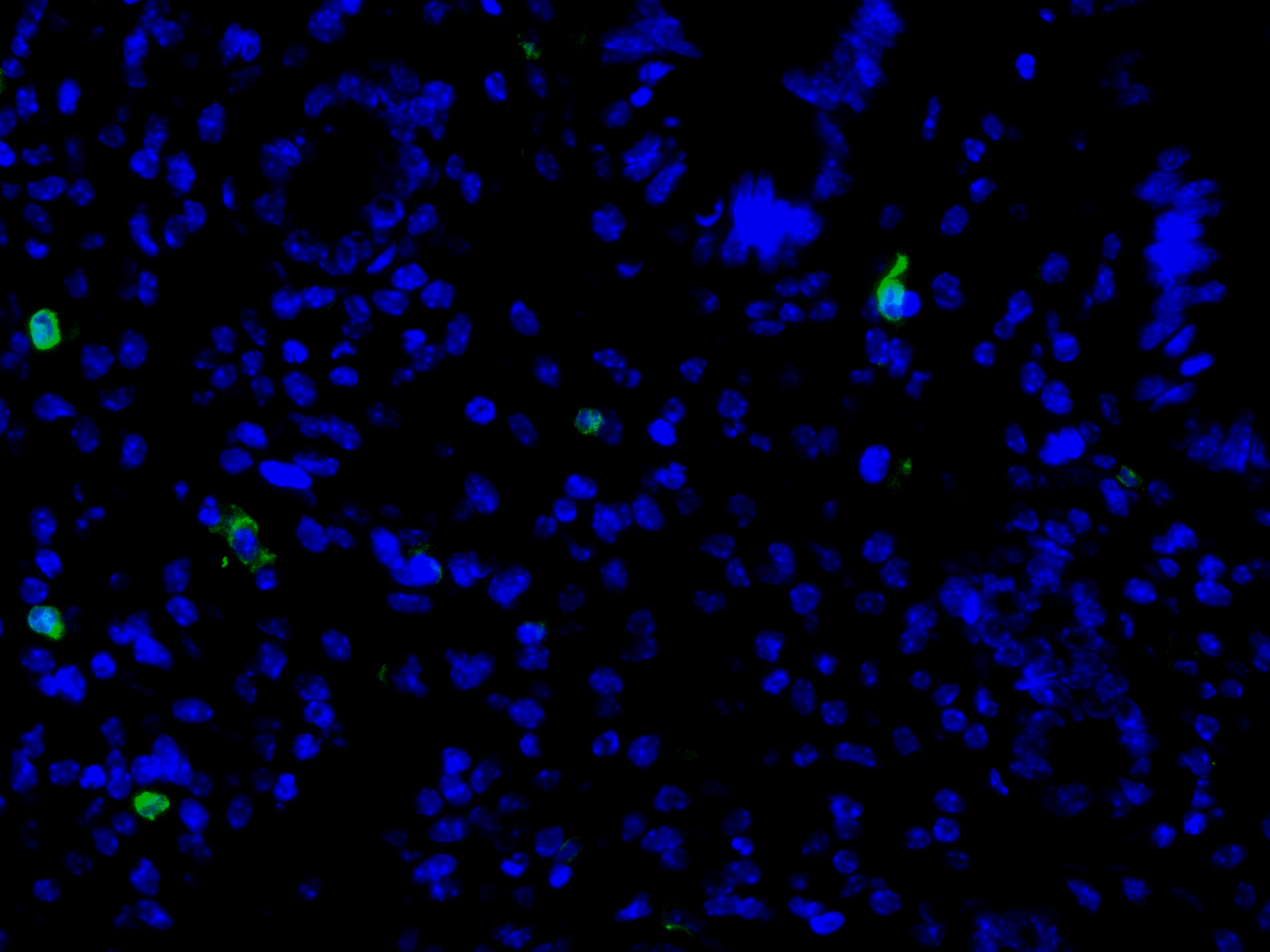

Supplement: Supplementary file 6 — Source Data for Figure 5 [file EMMM-15-e17601-s005.zip › Figure 5-2/5E/Sham CD301b.tif]

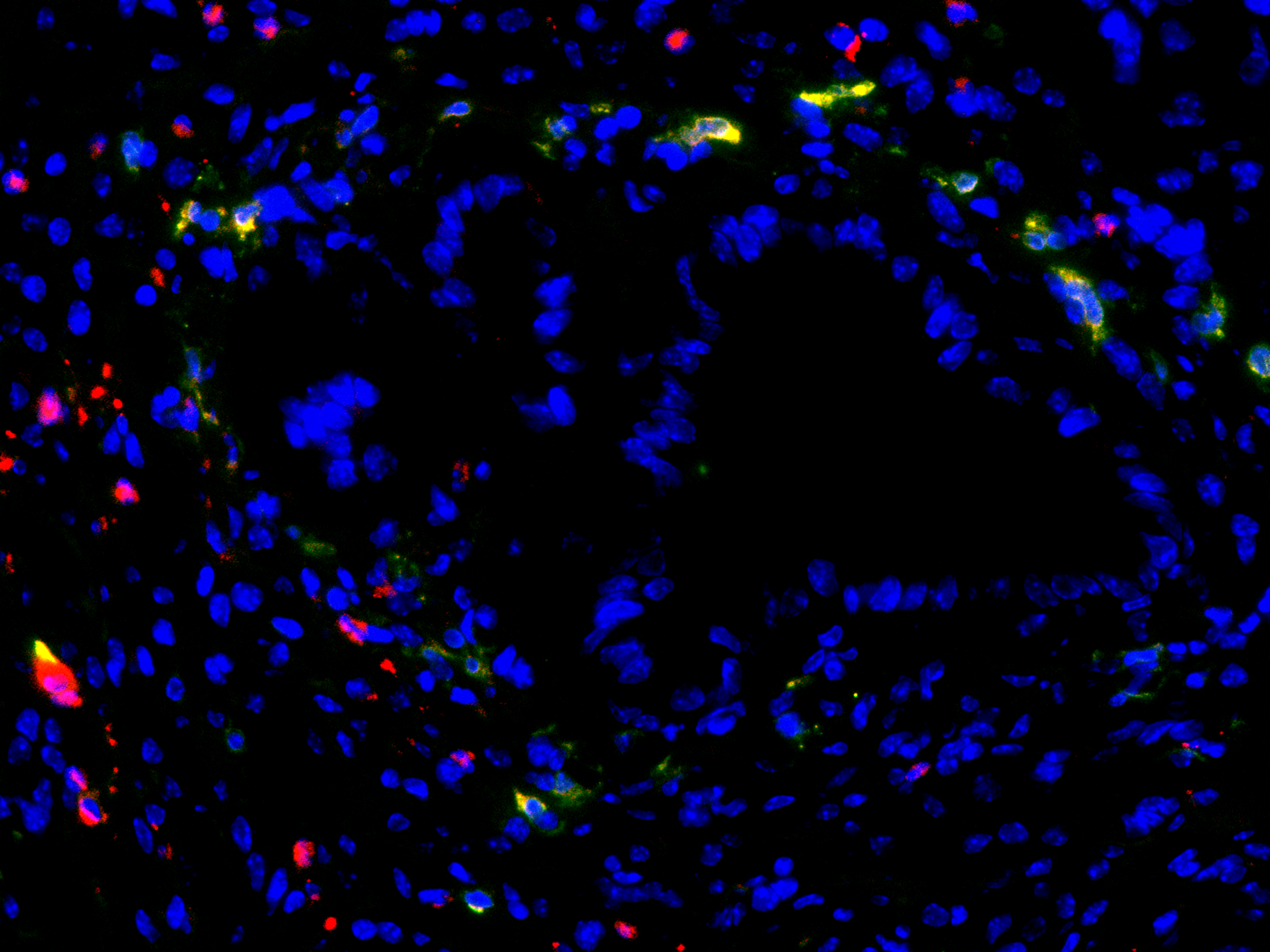

Supplement: Supplementary file 6 — Source Data for Figure 5 [file EMMM-15-e17601-s005.zip › Figure 5-2/5E/IUA GAS6-CD301b.tif]

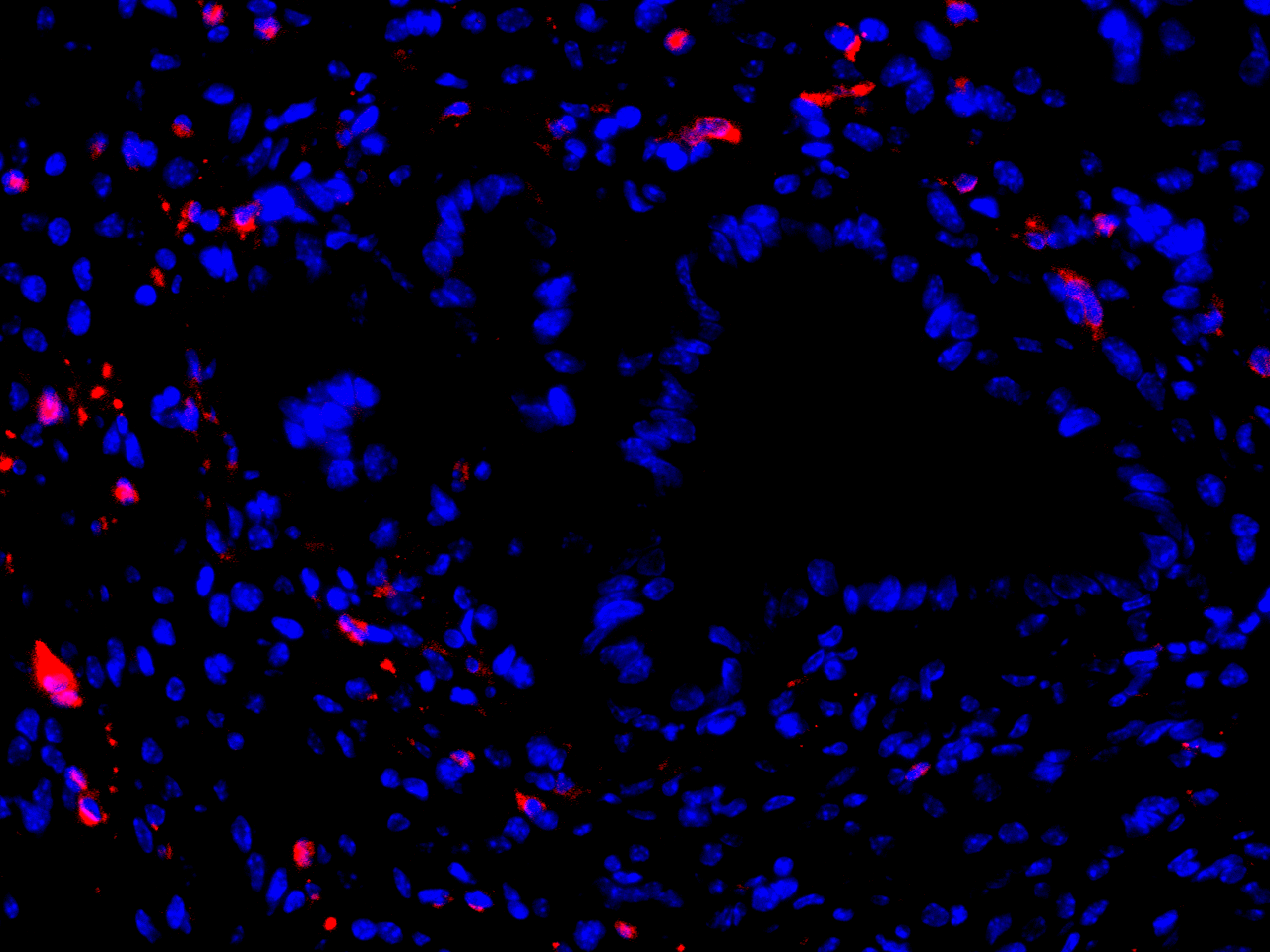

Supplement: Supplementary file 6 — Source Data for Figure 5 [file EMMM-15-e17601-s005.zip › Figure 5-2/5E/IUA GAS6.tif]

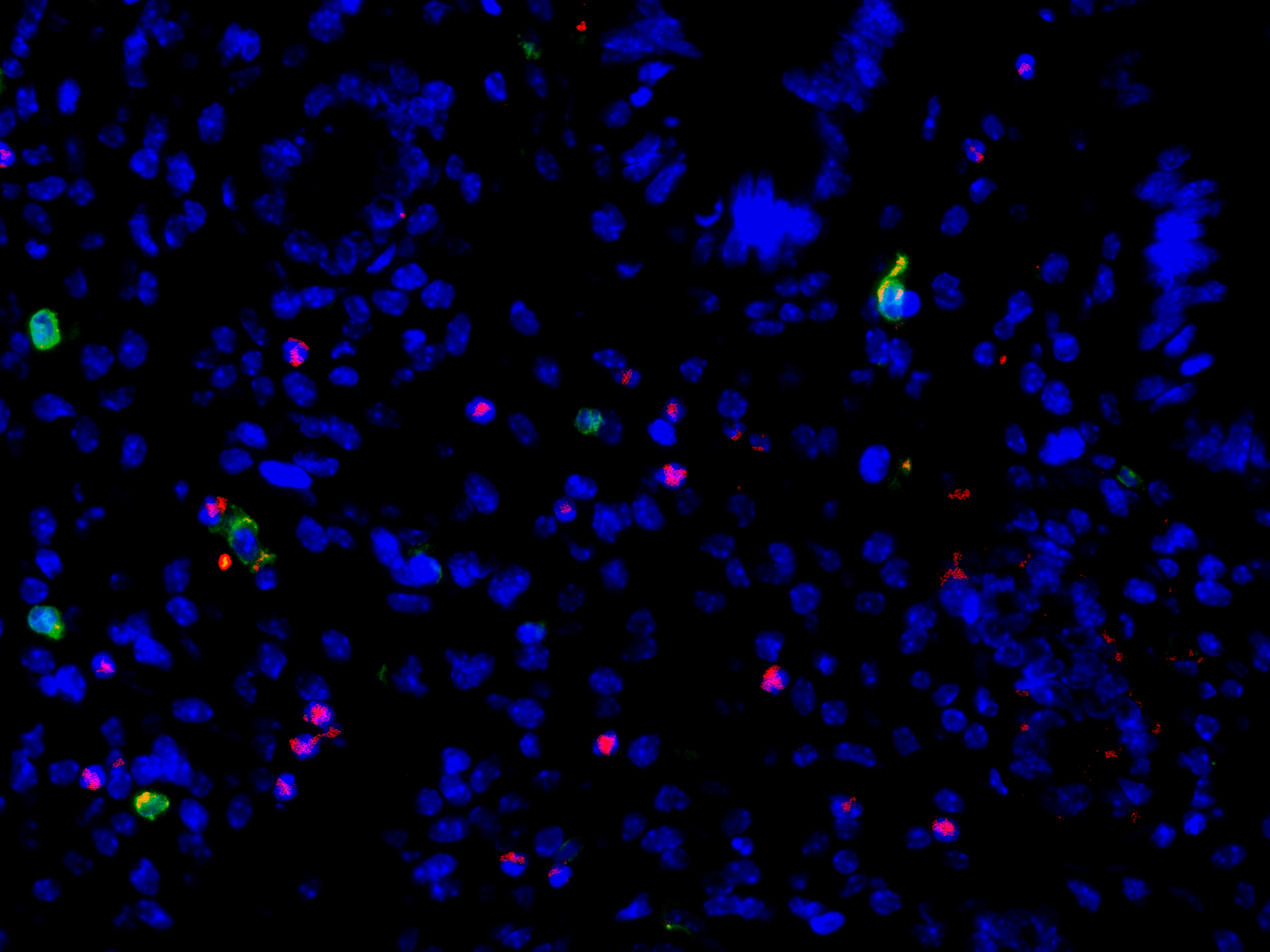

Supplement: Supplementary file 6 — Source Data for Figure 5 [file EMMM-15-e17601-s005.zip › Figure 5-2/5E/Sham GAS6-CD301b.tif]

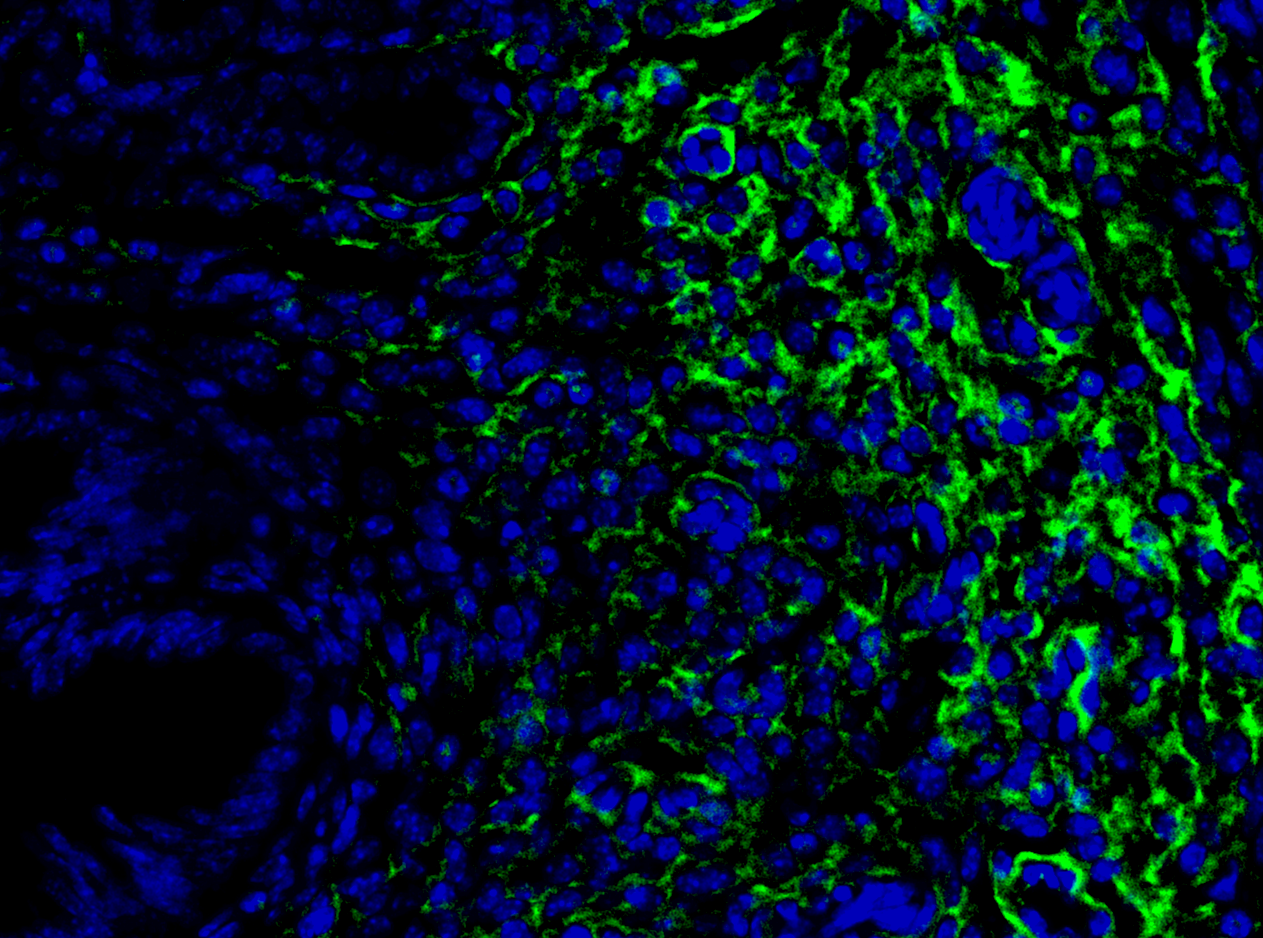

Supplement: Supplementary file 6 — Source Data for Figure 5 [file EMMM-15-e17601-s005.zip › Figure 5-2/5B/Collagen1 IUA.tif]

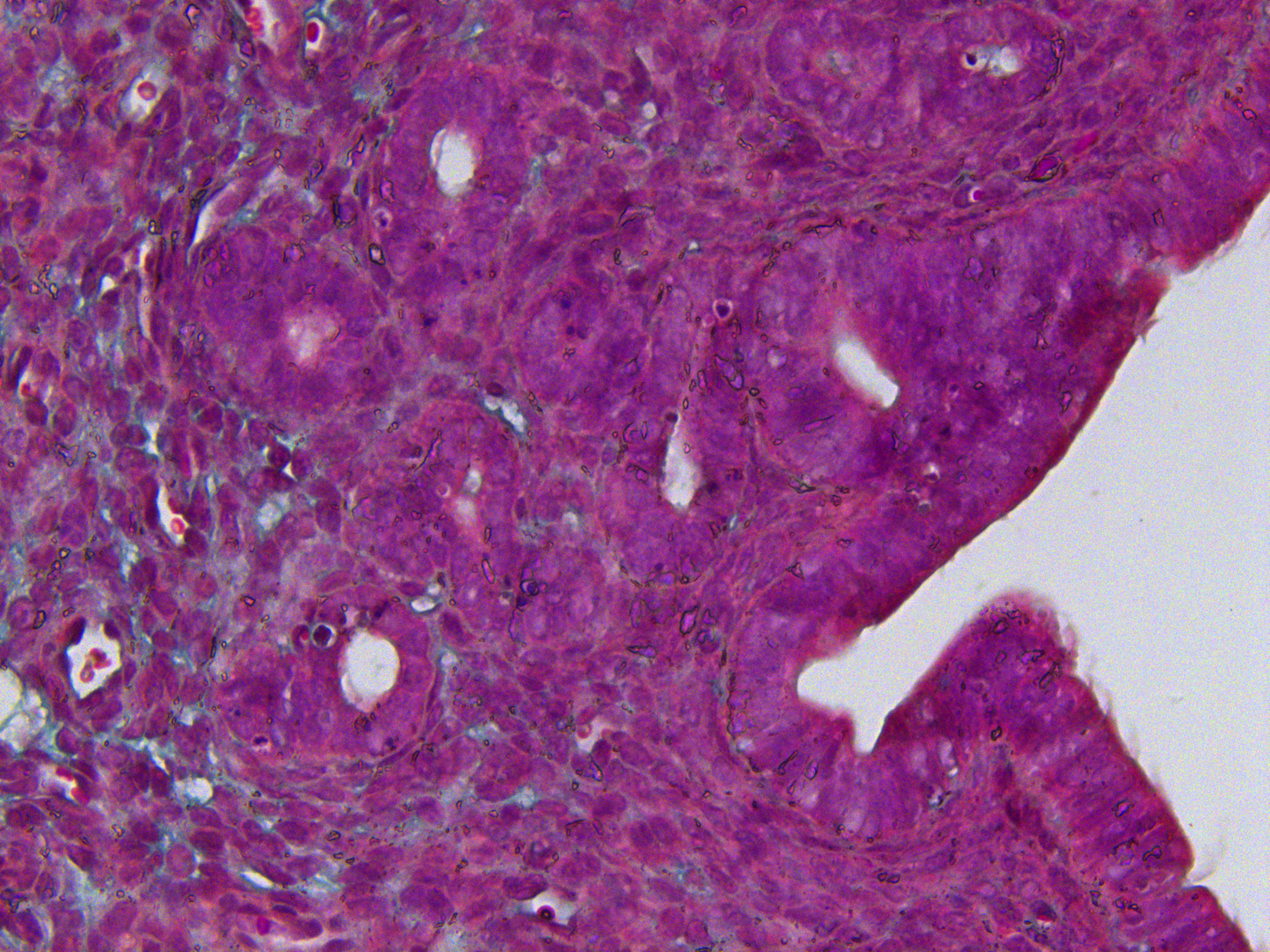

Supplement: Supplementary file 6 — Source Data for Figure 5 [file EMMM-15-e17601-s005.zip › Figure 5-2/5B/Masson Sham.tif]

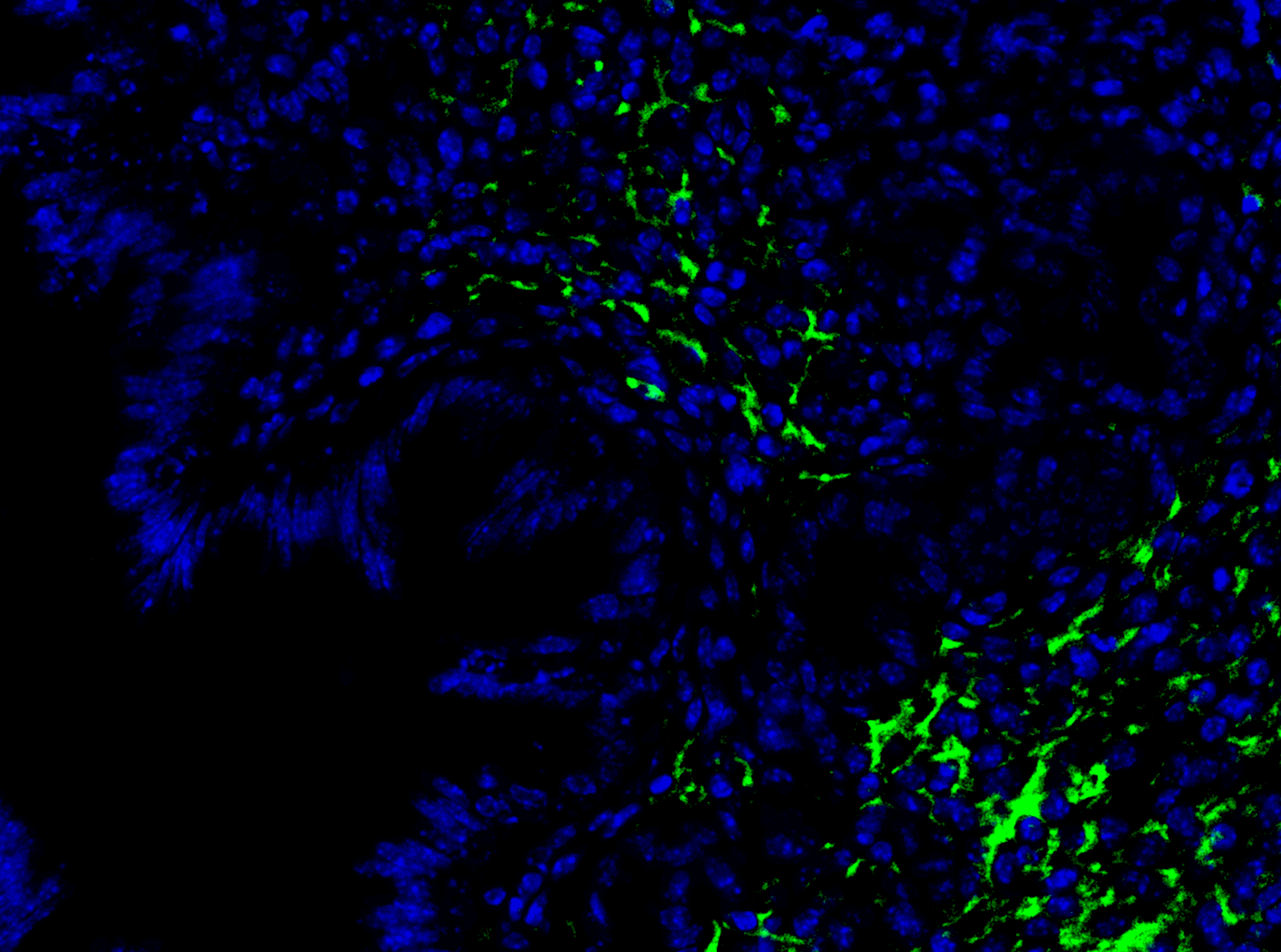

Supplement: Supplementary file 6 — Source Data for Figure 5 [file EMMM-15-e17601-s005.zip › Figure 5-2/5B/Collagen 1 Sham.tif]

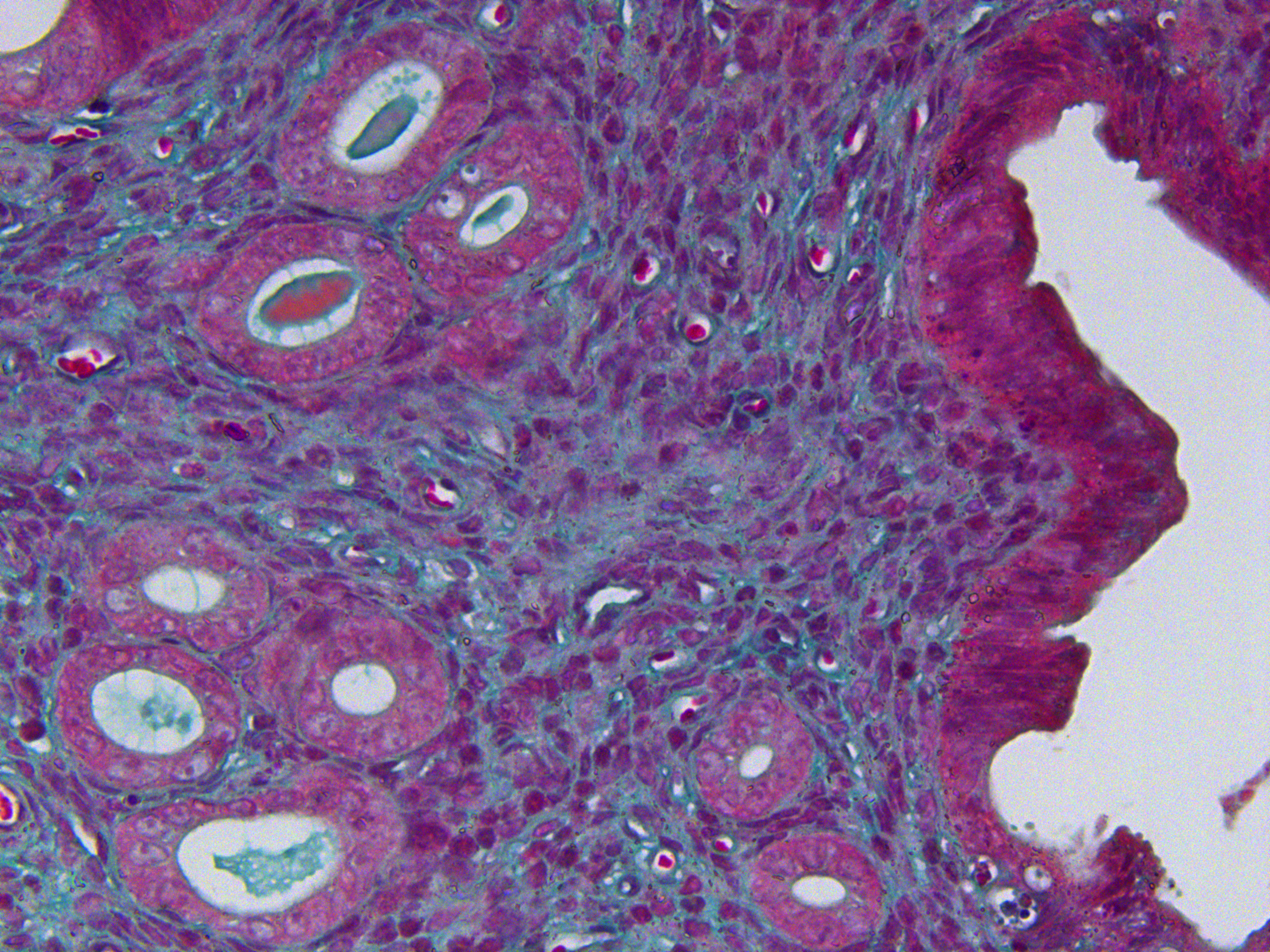

Supplement: Supplementary file 6 — Source Data for Figure 5 [file EMMM-15-e17601-s005.zip › Figure 5-2/5B/Masson IUA.tif]

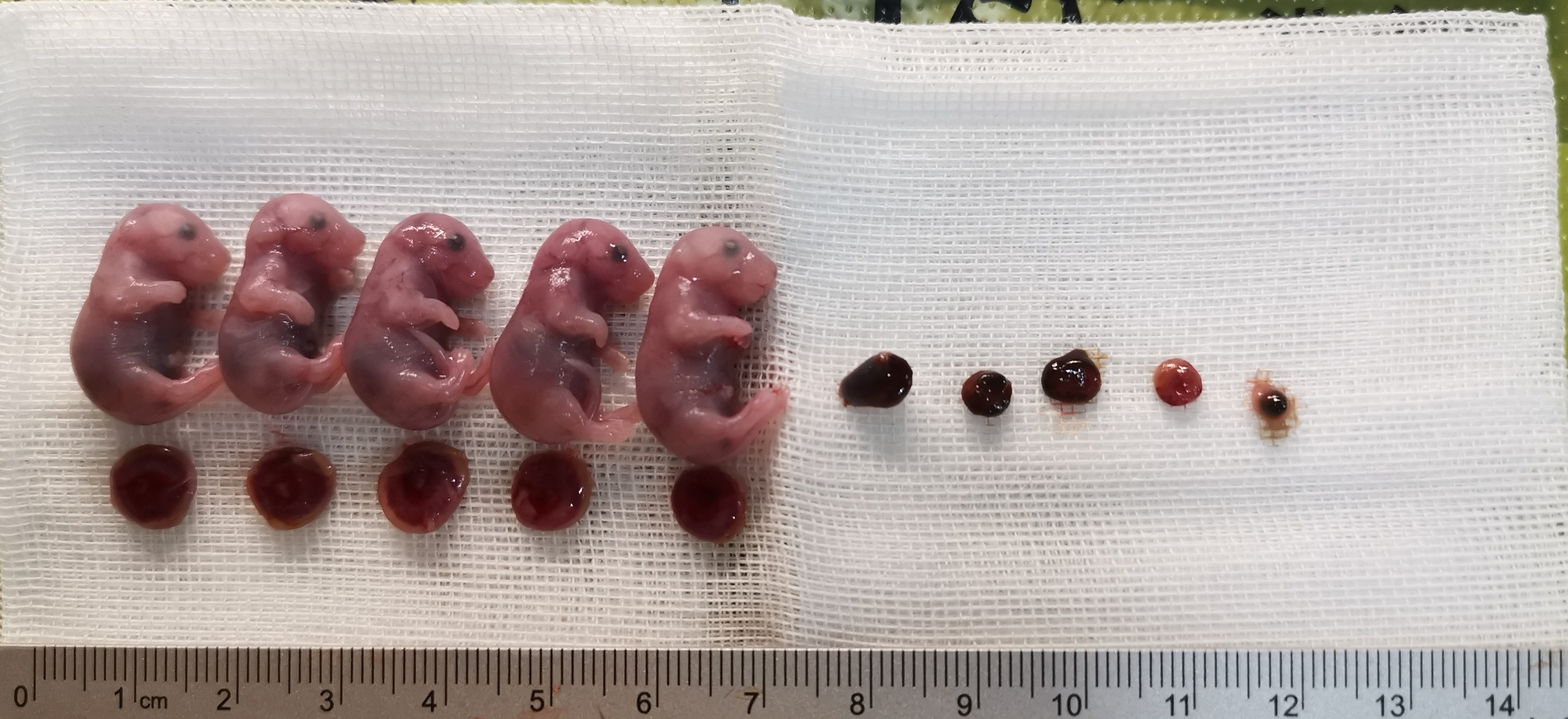

Supplement: Supplementary file 7 — Source Data for Figure 6 [file EMMM-15-e17601-s006.zip › Figure 6-1/6F/IUA-2.jpg]

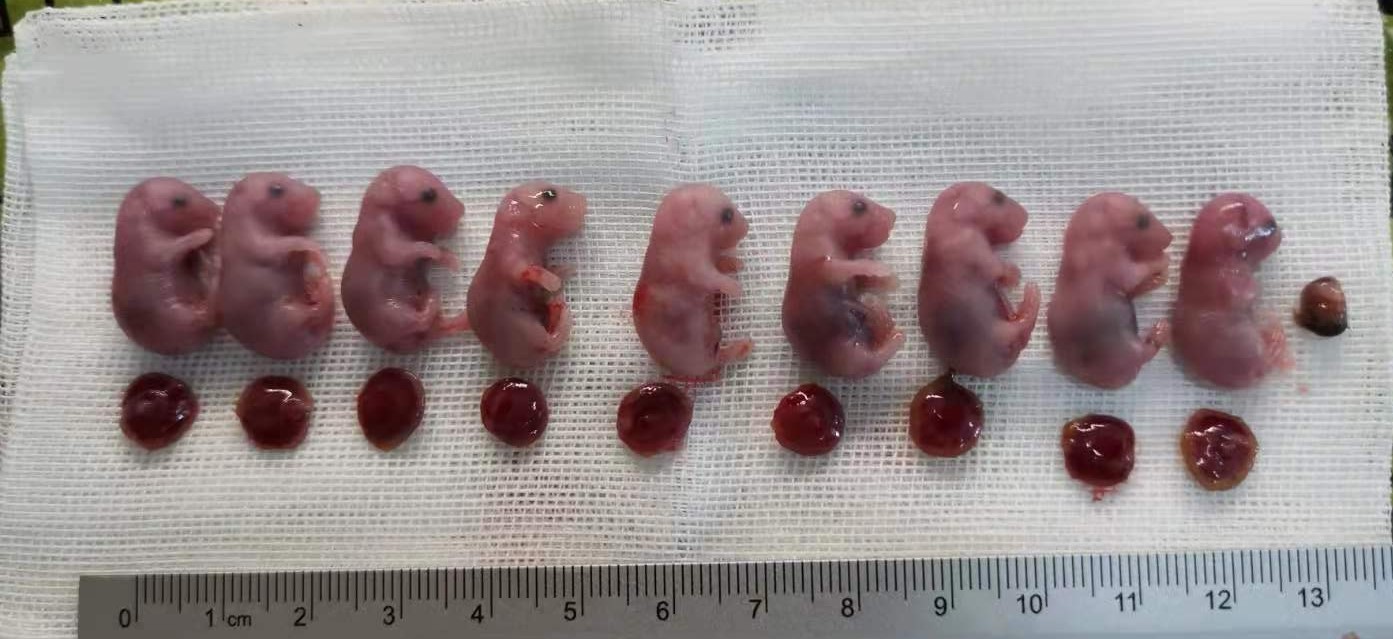

Supplement: Supplementary file 7 — Source Data for Figure 6 [file EMMM-15-e17601-s006.zip › Figure 6-1/6F/Sham-2.jpg]

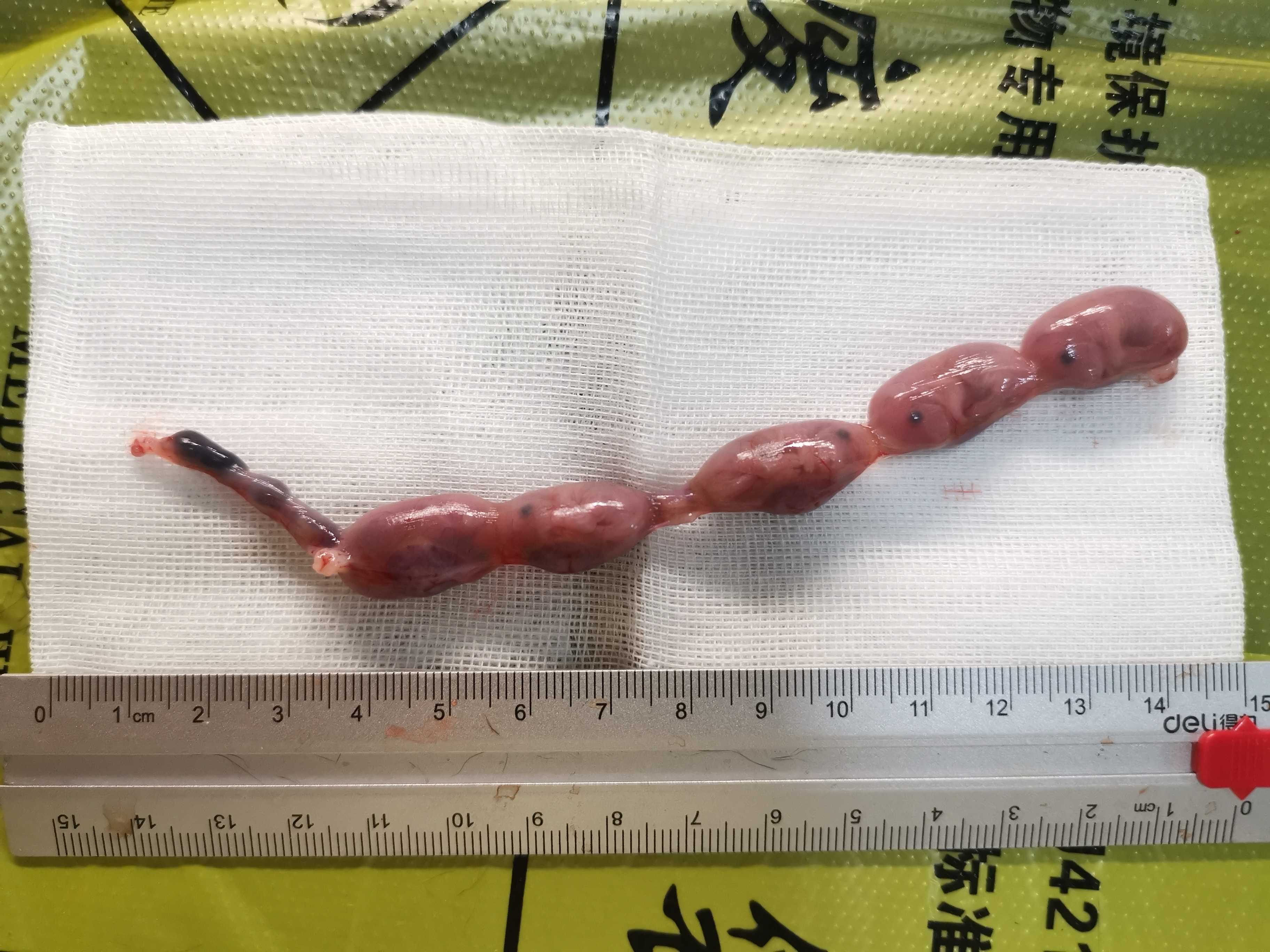

Supplement: Supplementary file 7 — Source Data for Figure 6 [file EMMM-15-e17601-s006.zip › Figure 6-1/6F/IUA-1.jpg]

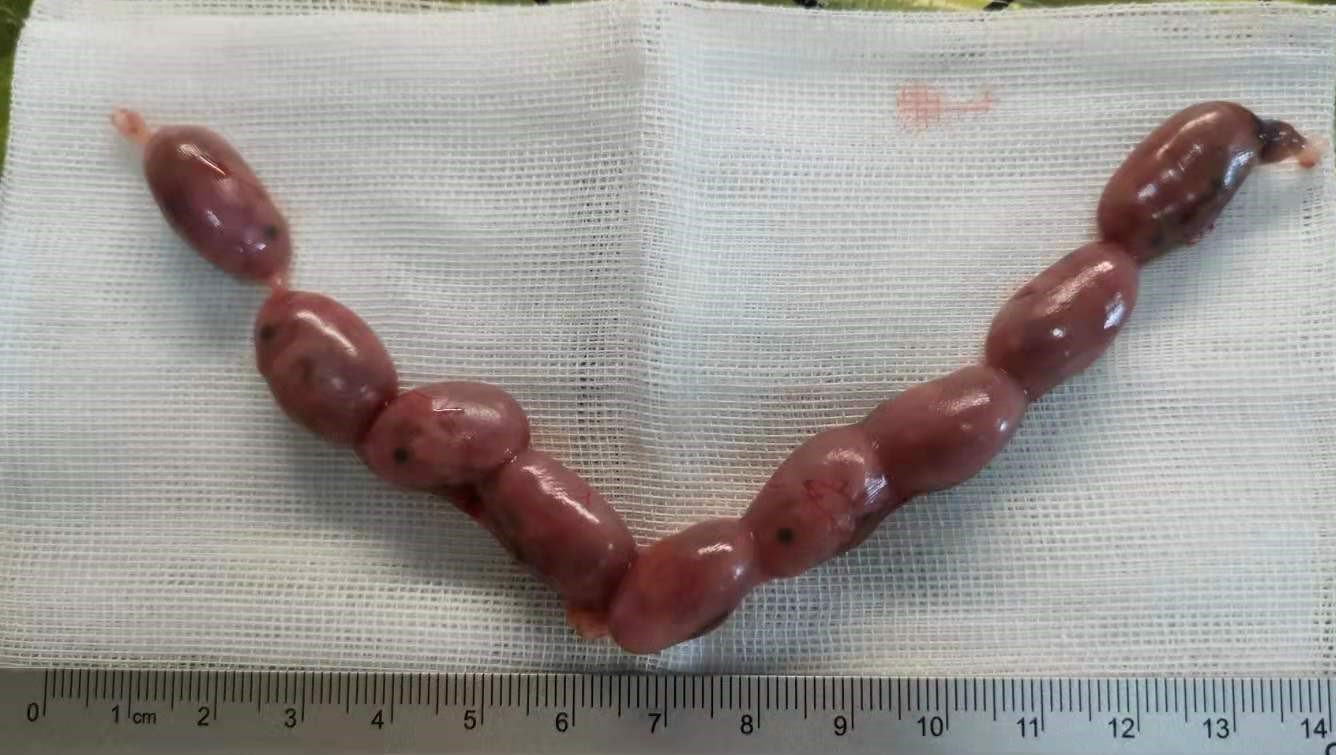

Supplement: Supplementary file 7 — Source Data for Figure 6 [file EMMM-15-e17601-s006.zip › Figure 6-1/6F/Sham-1.jpg]

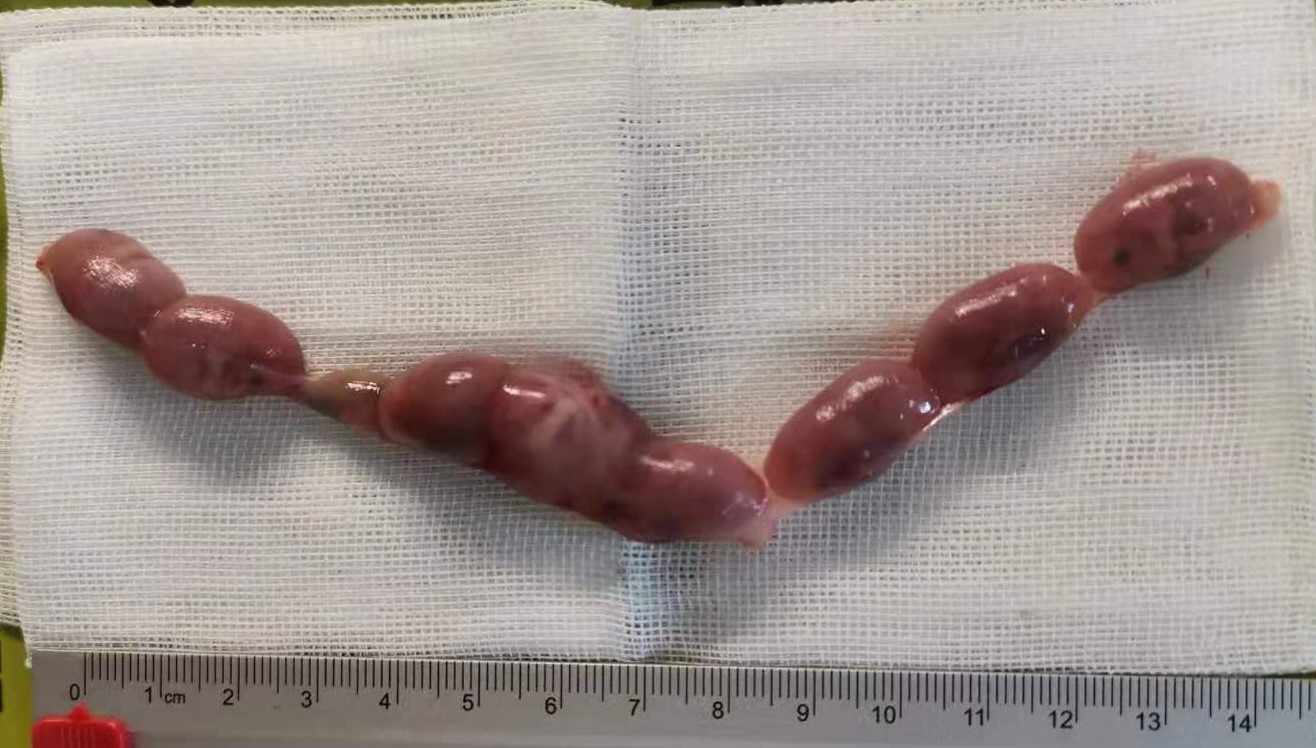

Supplement: Supplementary file 7 — Source Data for Figure 6 [file EMMM-15-e17601-s006.zip › Figure 6-1/6F/IUA+DT-1.jpg]

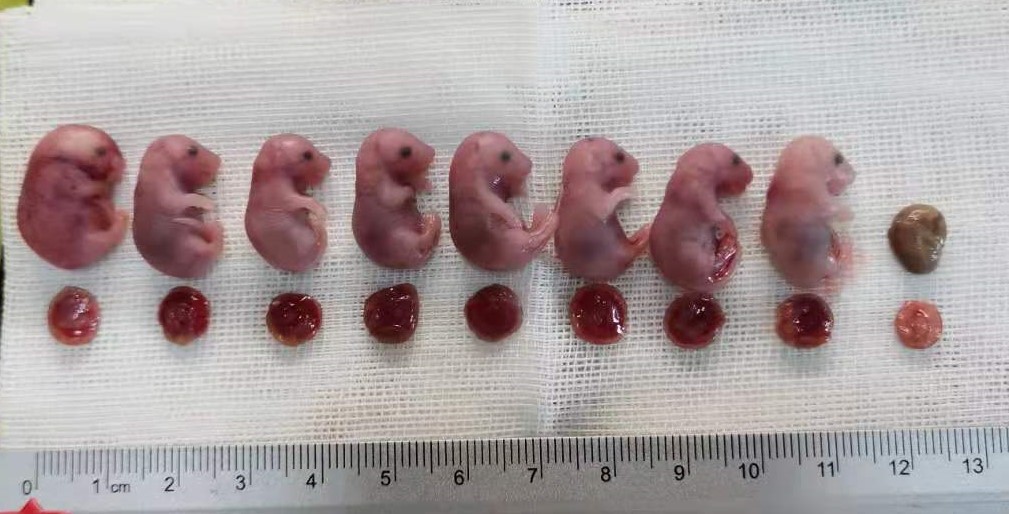

Supplement: Supplementary file 7 — Source Data for Figure 6 [file EMMM-15-e17601-s006.zip › Figure 6-1/6F/IUA+DT-2.jpg]

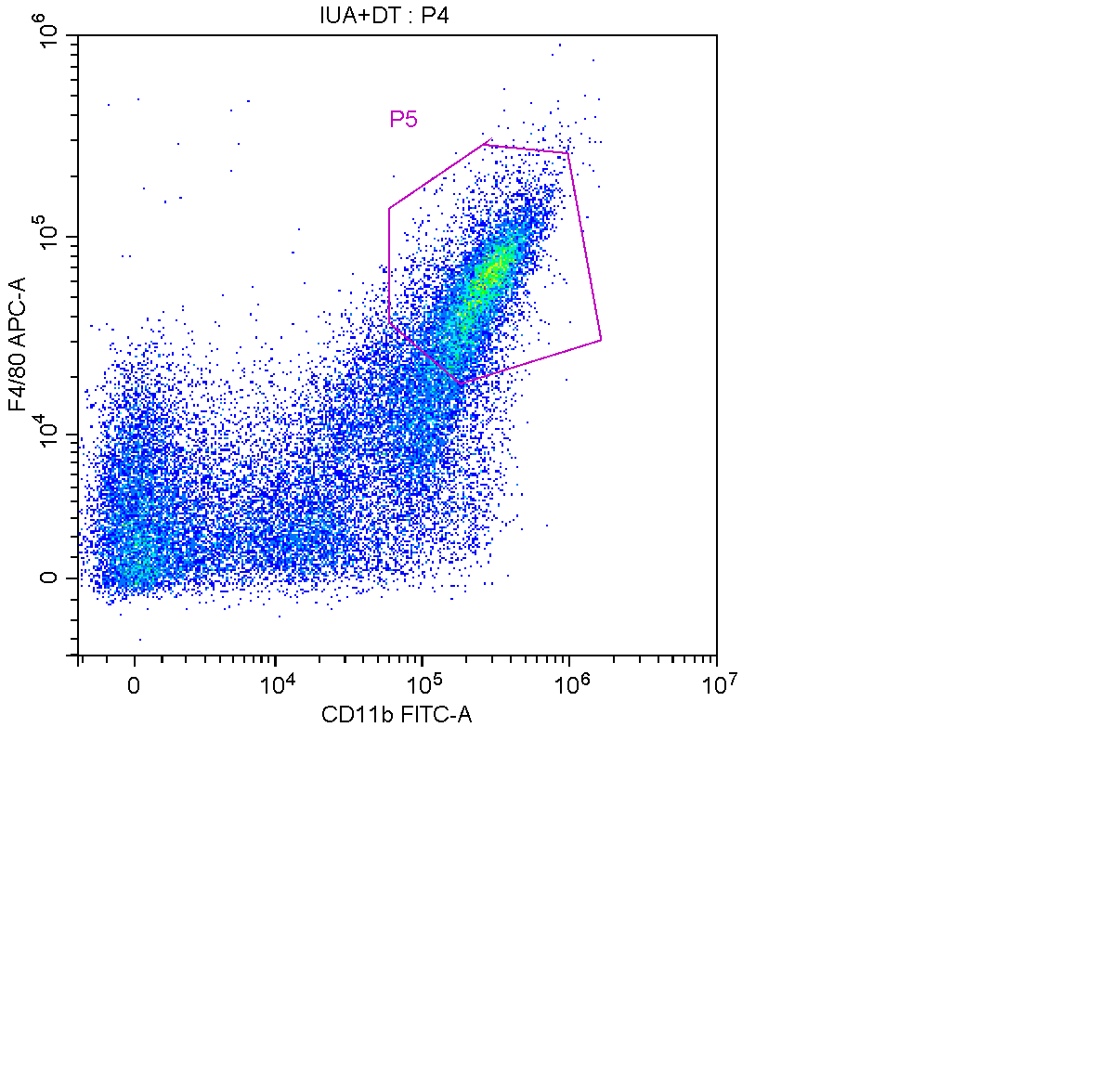

Supplement: Supplementary file 7 — Source Data for Figure 6 [file EMMM-15-e17601-s006.zip › Figure 6-1/6B/IUA+DT-1.bmp]

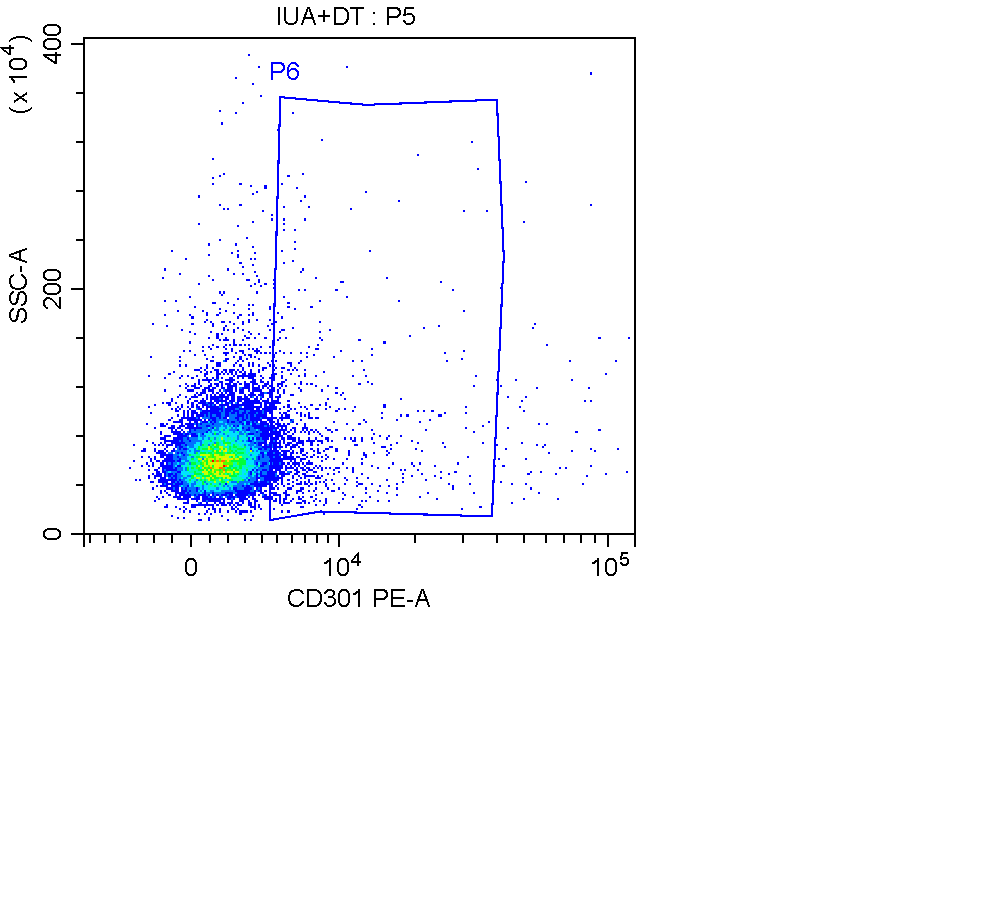

Supplement: Supplementary file 7 — Source Data for Figure 6 [file EMMM-15-e17601-s006.zip › Figure 6-1/6B/IUA+DT-2.bmp]

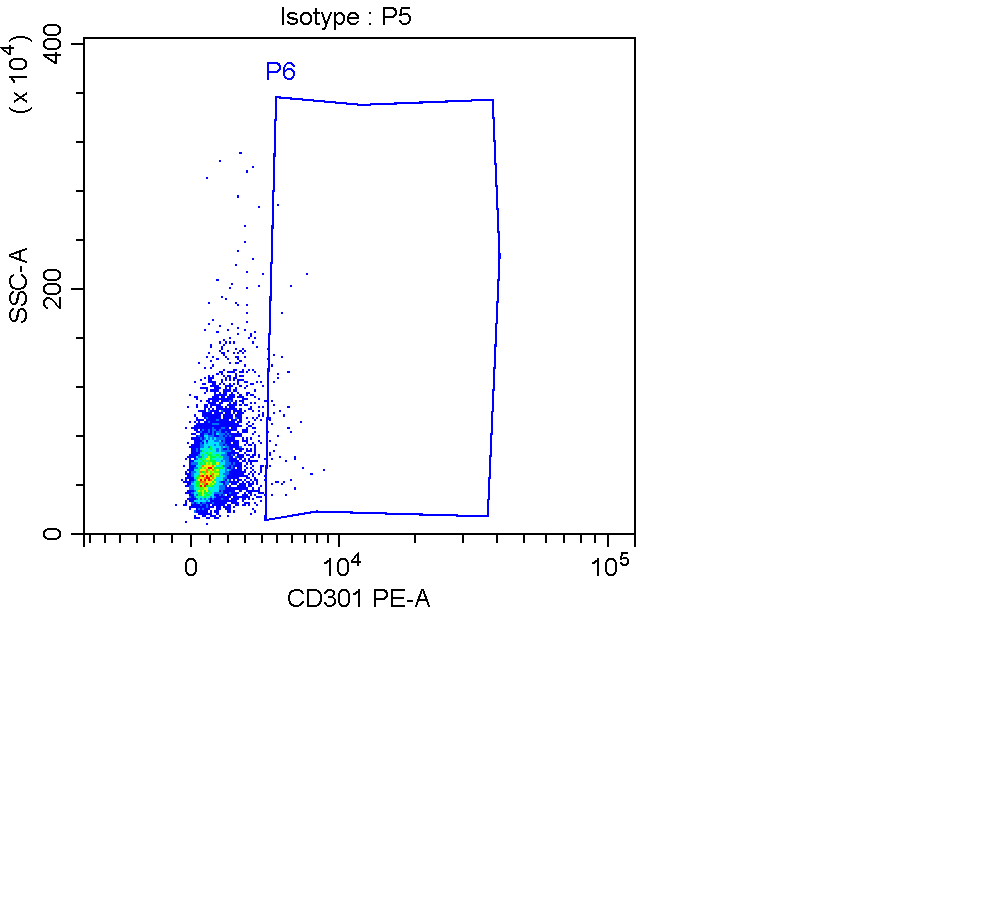

Supplement: Supplementary file 7 — Source Data for Figure 6 [file EMMM-15-e17601-s006.zip › Figure 6-1/6B/Isotype-1.bmp]

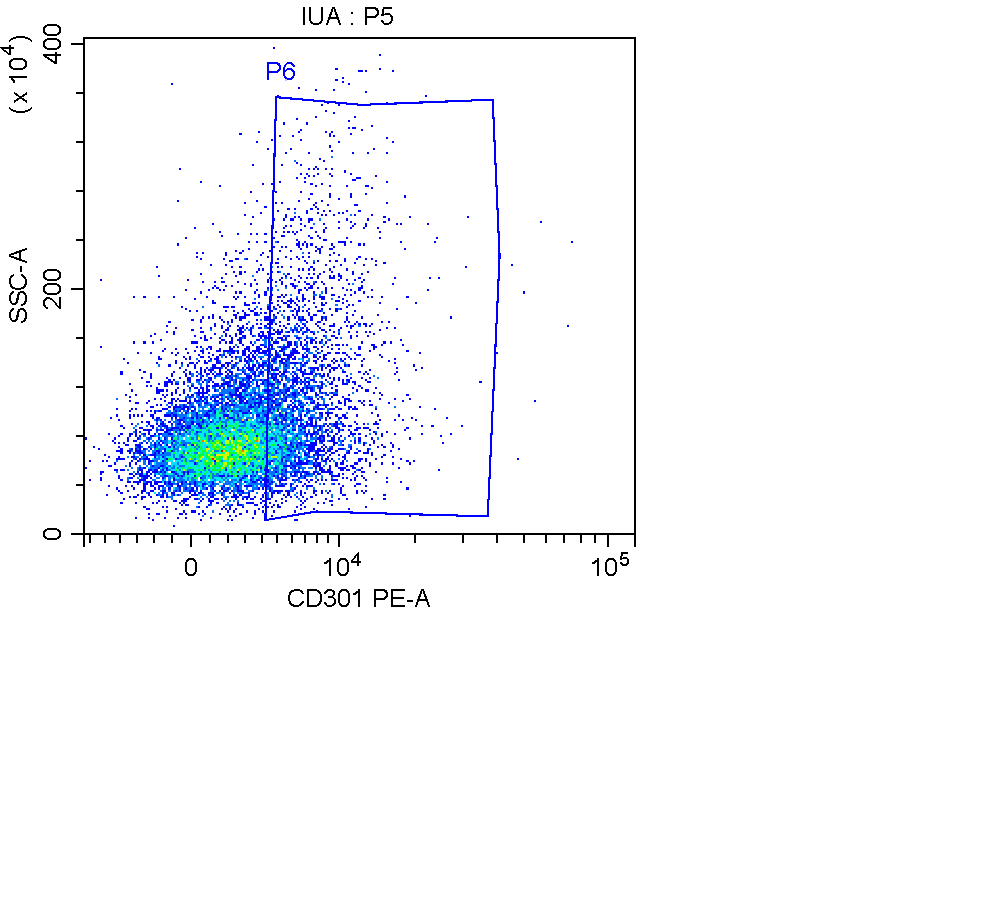

Supplement: Supplementary file 7 — Source Data for Figure 6 [file EMMM-15-e17601-s006.zip › Figure 6-1/6B/IUA-2.bmp]

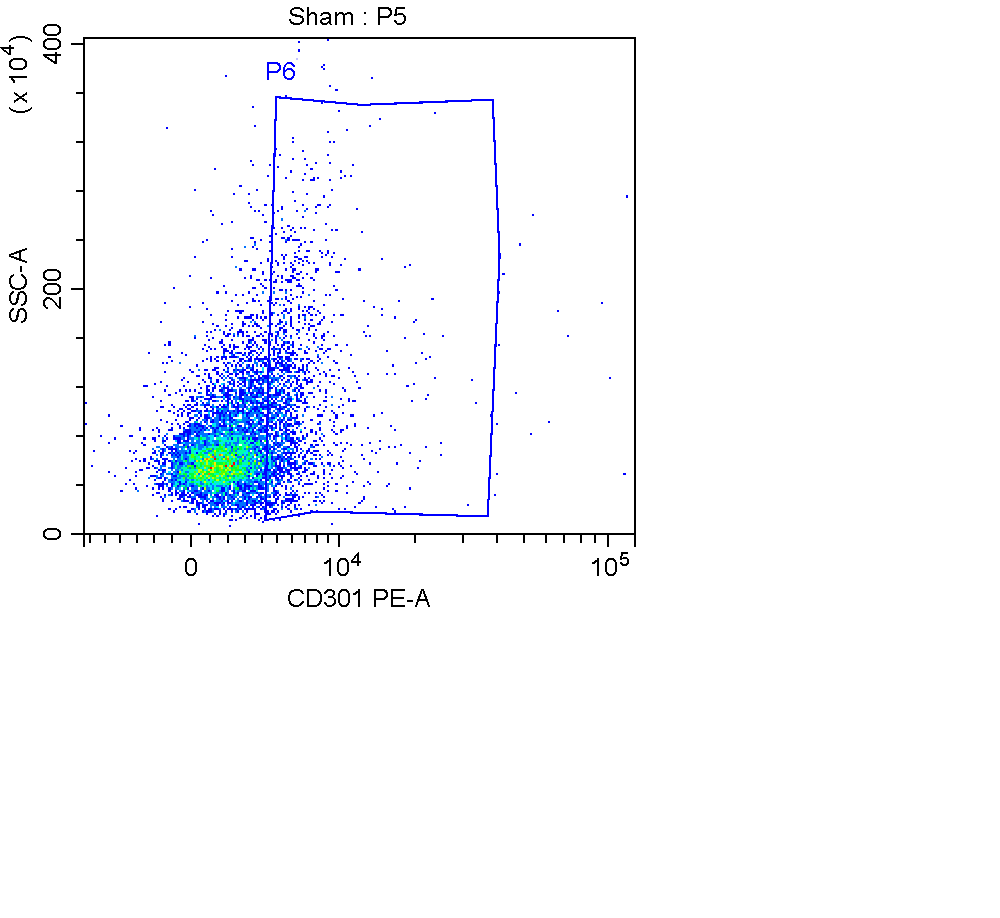

Supplement: Supplementary file 7 — Source Data for Figure 6 [file EMMM-15-e17601-s006.zip › Figure 6-1/6B/Sham-2.bmp]

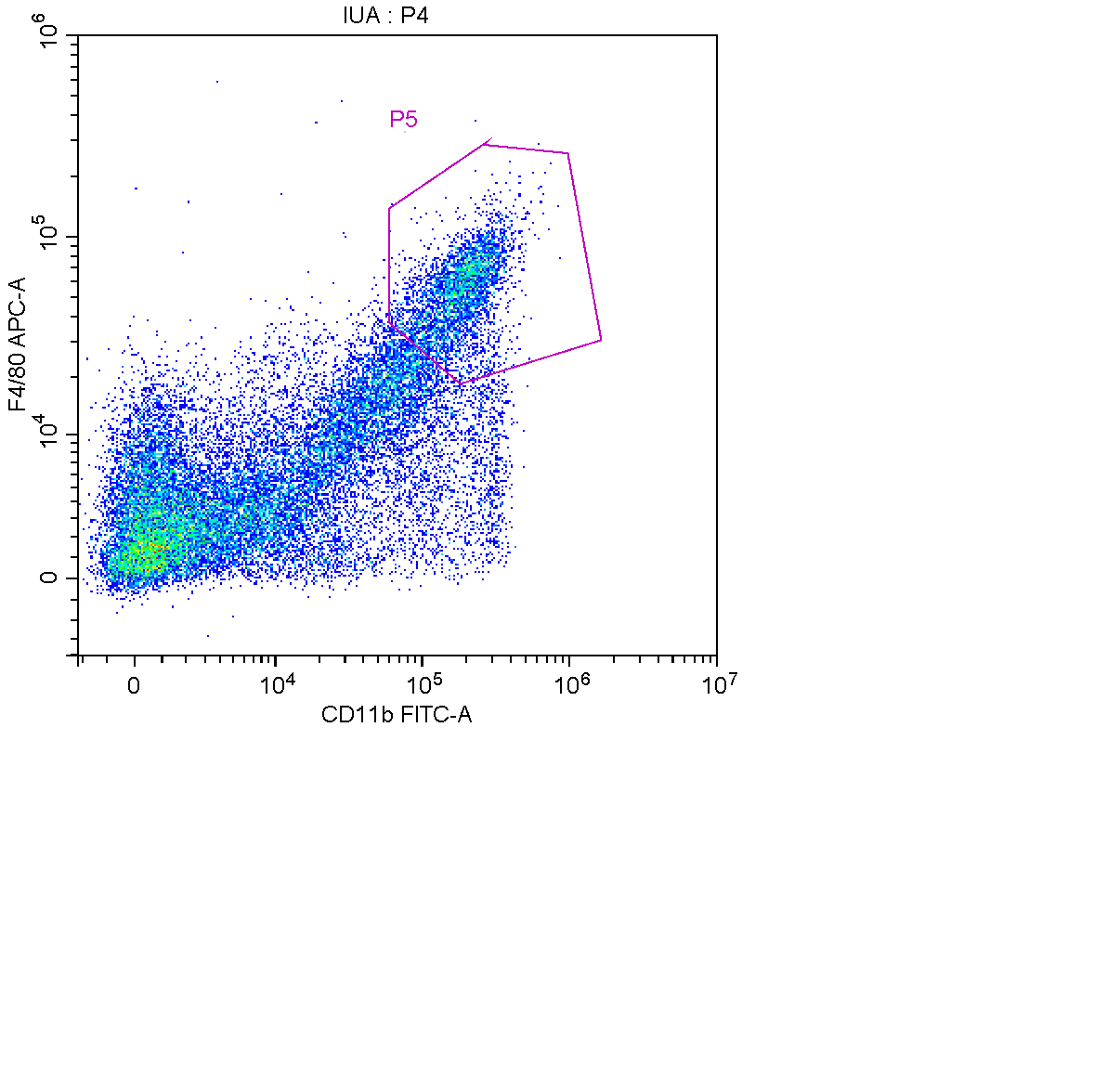

Supplement: Supplementary file 7 — Source Data for Figure 6 [file EMMM-15-e17601-s006.zip › Figure 6-1/6B/IUA-1.bmp]

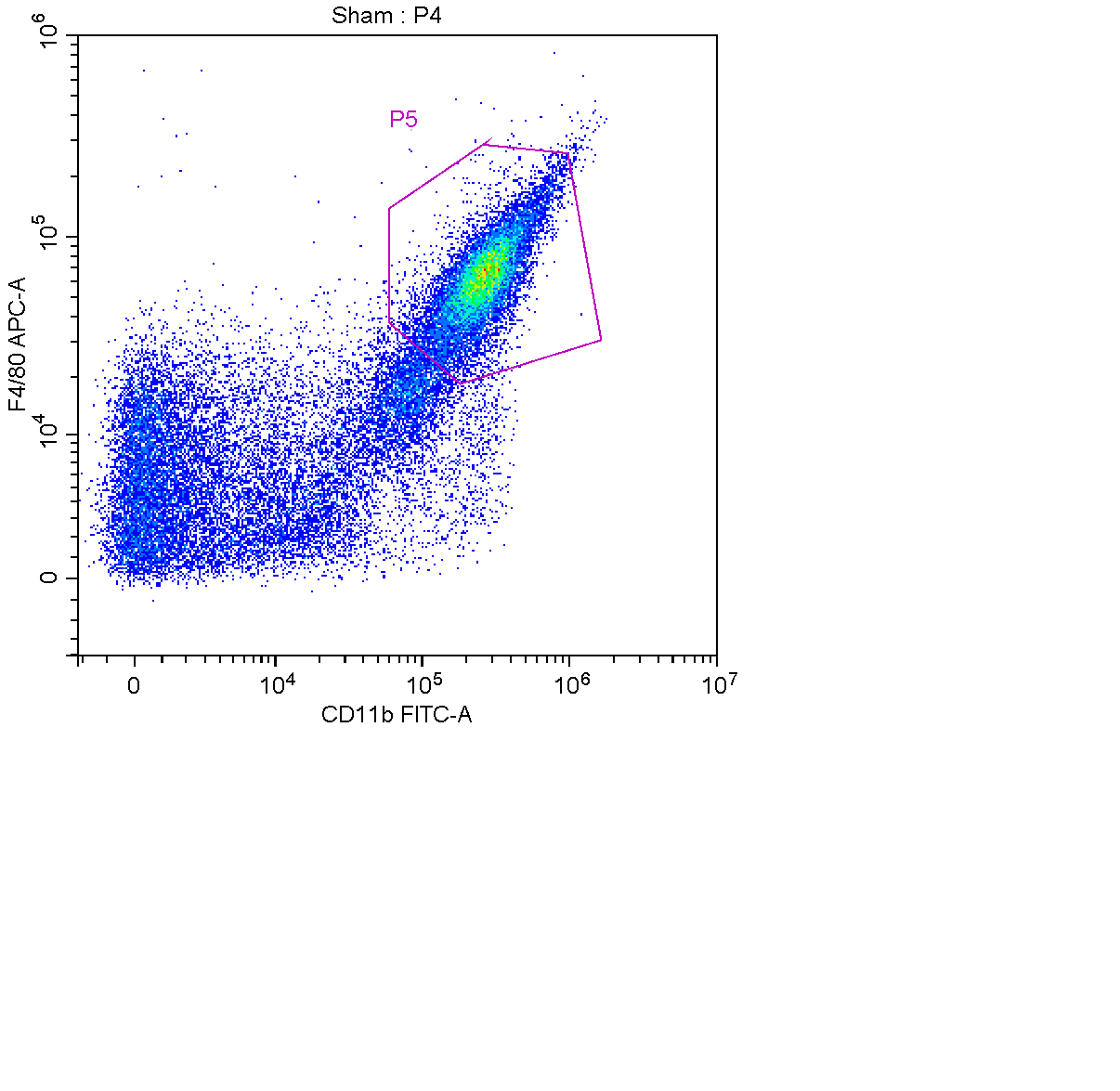

Supplement: Supplementary file 7 — Source Data for Figure 6 [file EMMM-15-e17601-s006.zip › Figure 6-1/6B/Sham-1.bmp]

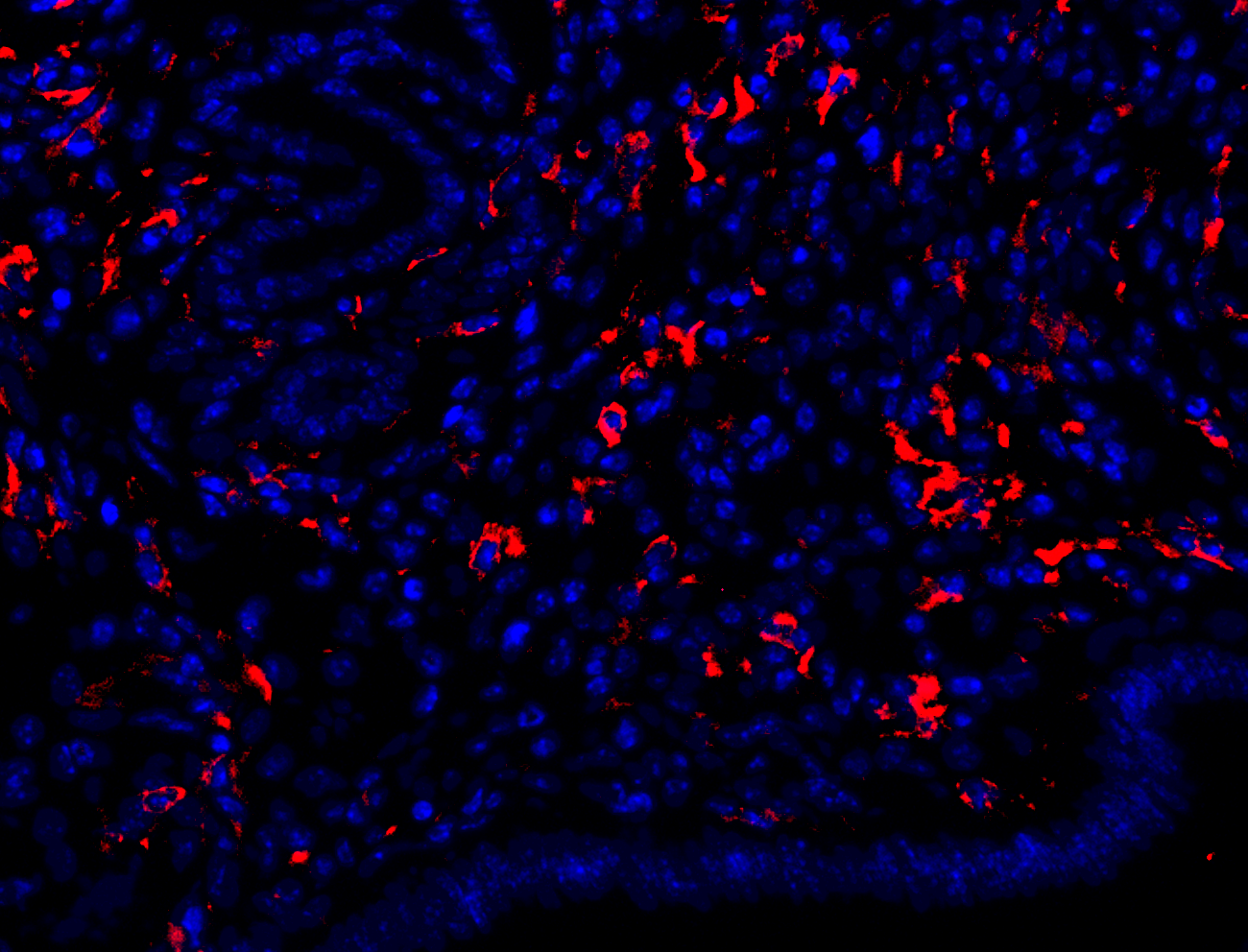

Supplement: Supplementary file 7 — Source Data for Figure 6 [file EMMM-15-e17601-s006.zip › Figure 6-1/6E/AXL IUA.tif]

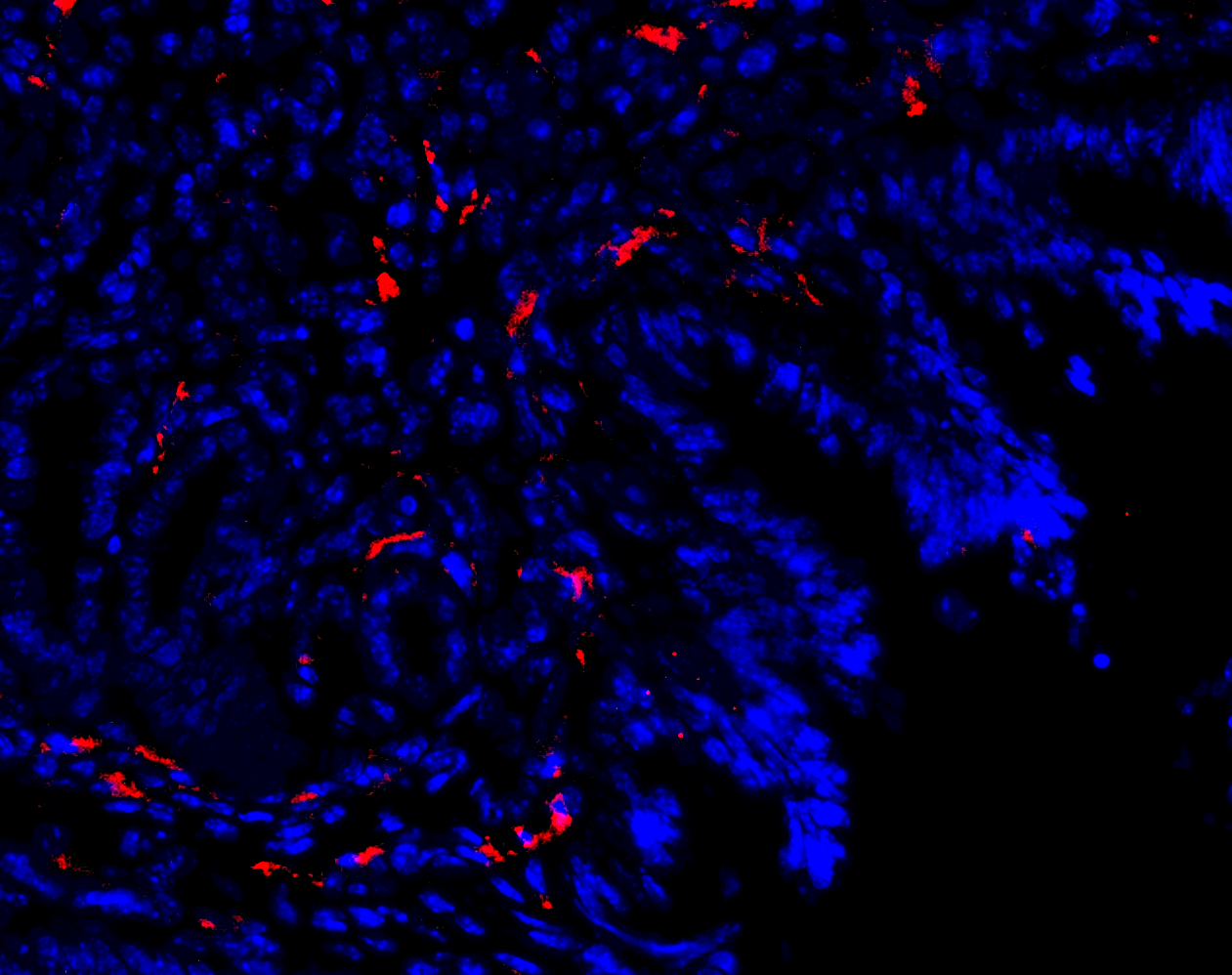

Supplement: Supplementary file 7 — Source Data for Figure 6 [file EMMM-15-e17601-s006.zip › Figure 6-1/6E/AXL Sham.tif]

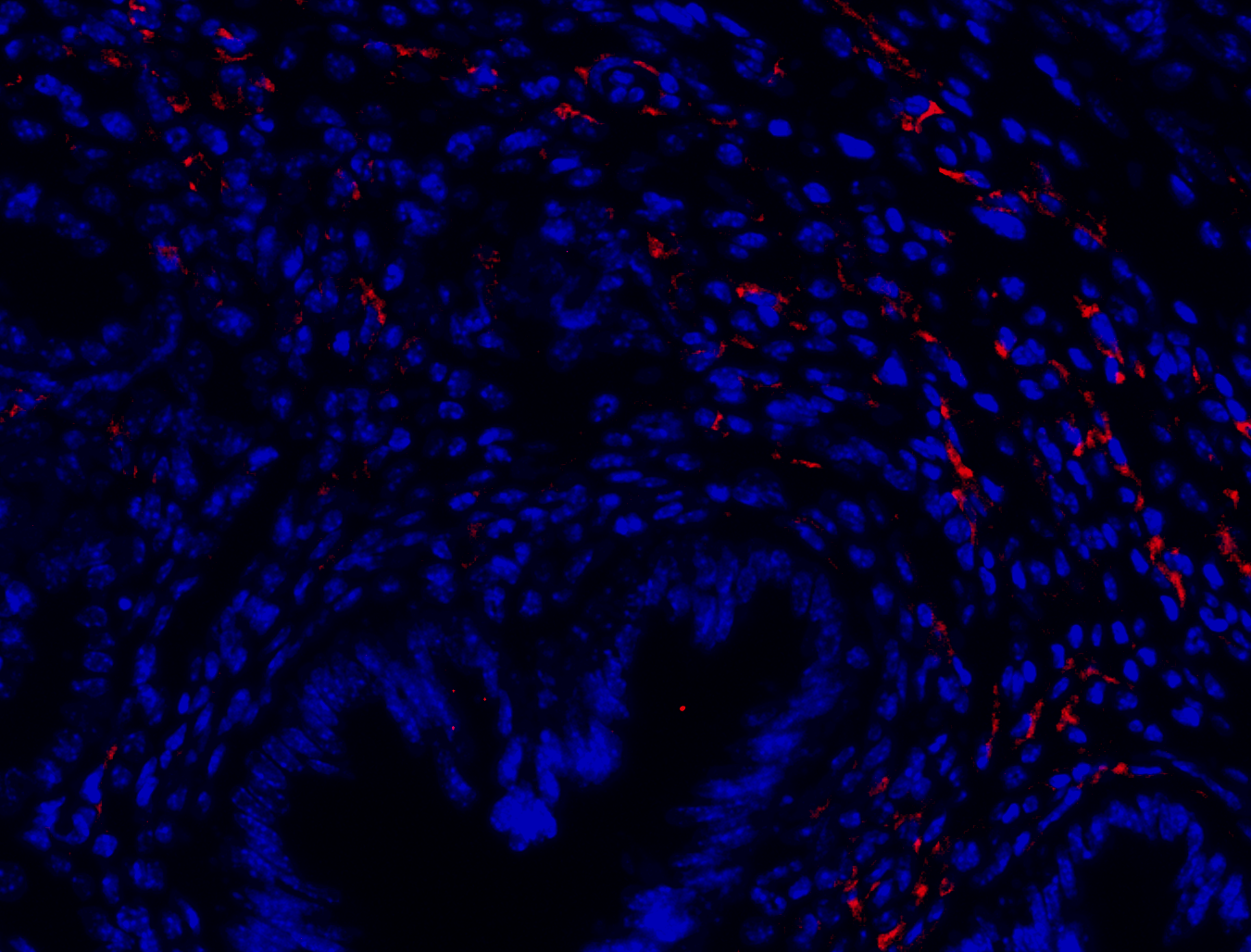

Supplement: Supplementary file 7 — Source Data for Figure 6 [file EMMM-15-e17601-s006.zip › Figure 6-1/6E/AXL IUA+DT.tif]

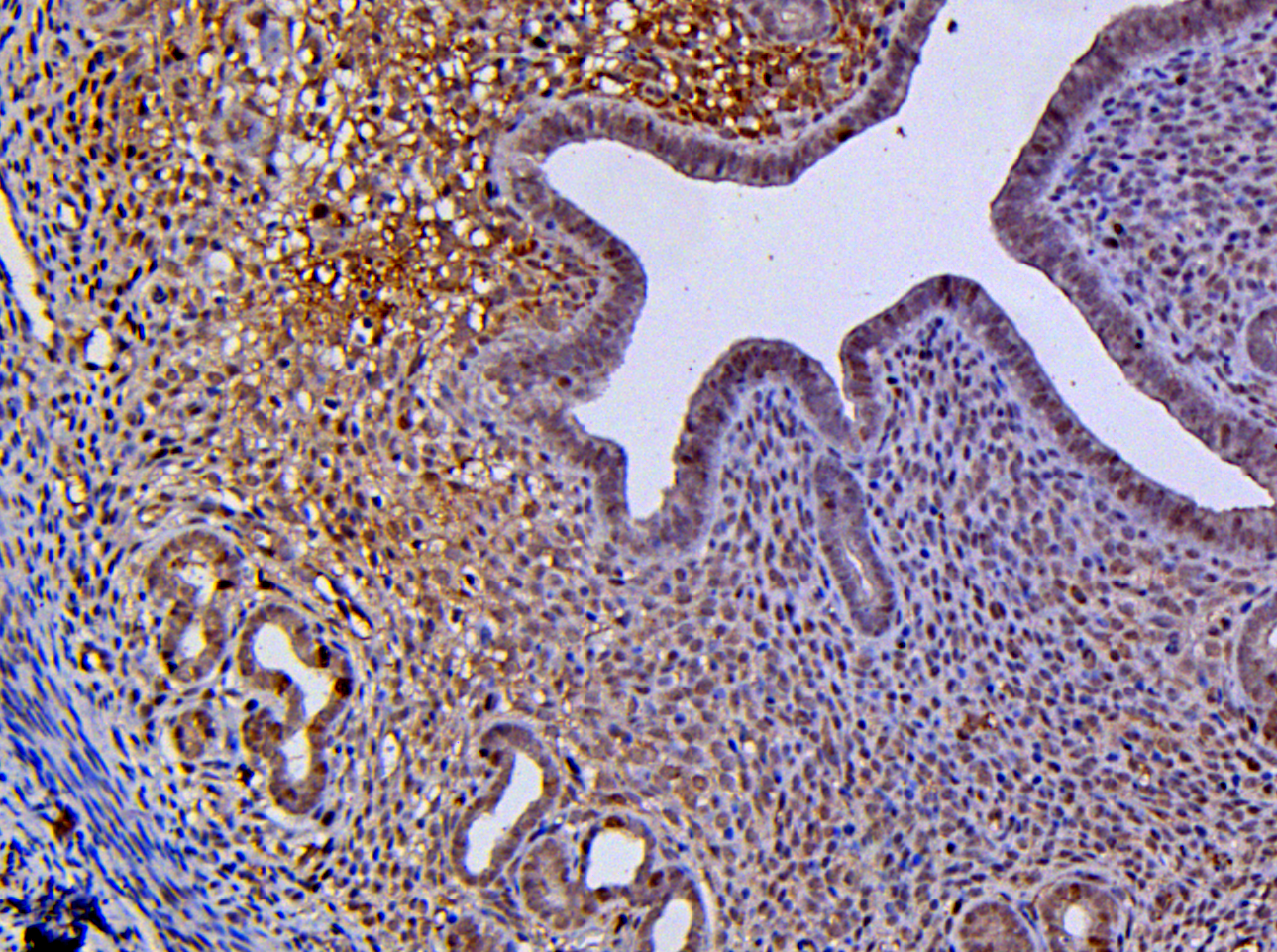

Supplement: Supplementary file 7 — Source Data for Figure 6 [file EMMM-15-e17601-s006.zip › Figure 6-1/6E/p-p65 IUA.tif]

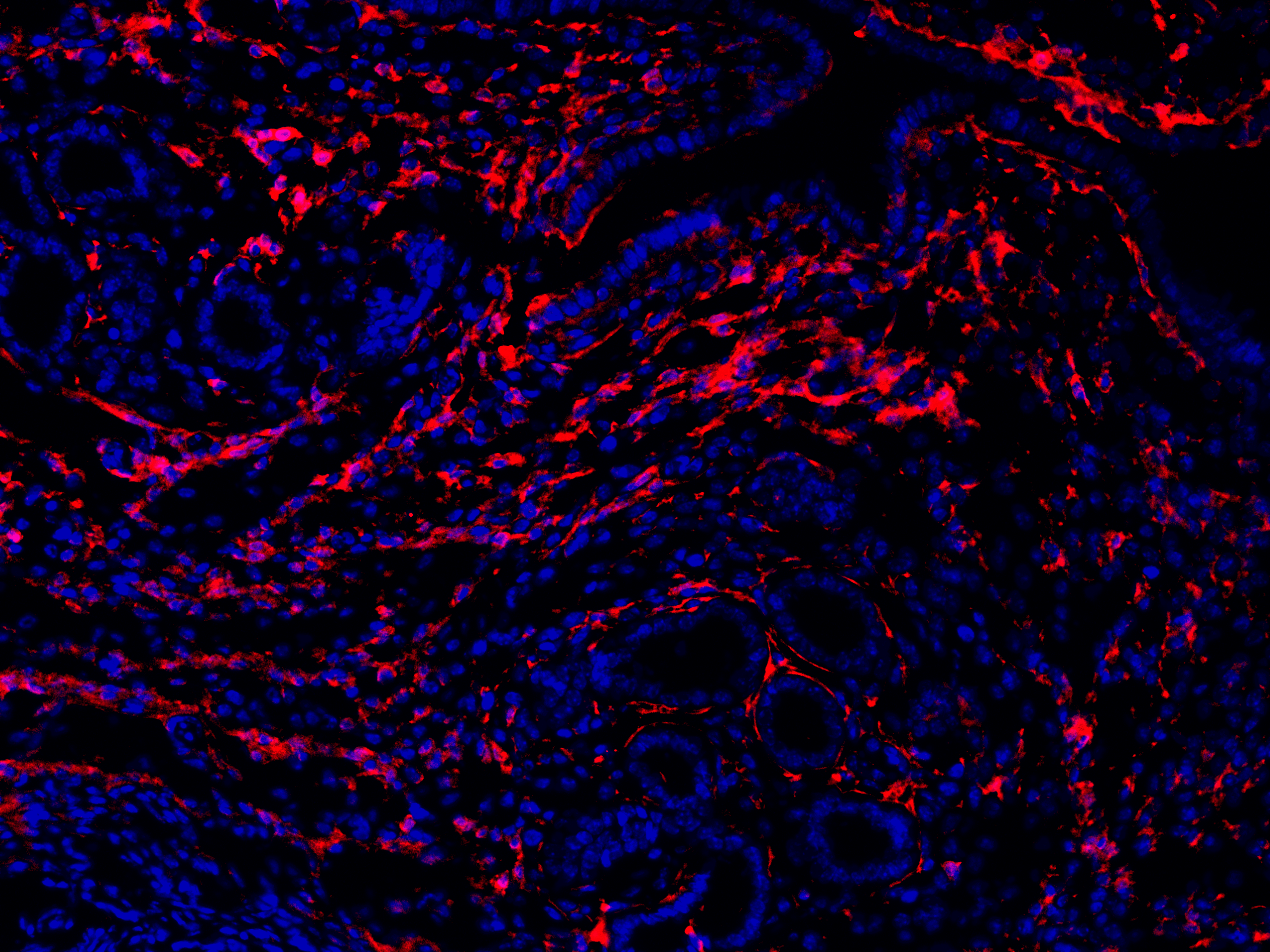

Supplement: Supplementary file 7 — Source Data for Figure 6 [file EMMM-15-e17601-s006.zip › Figure 6-1/6E/Collagen1 IUA.tif]

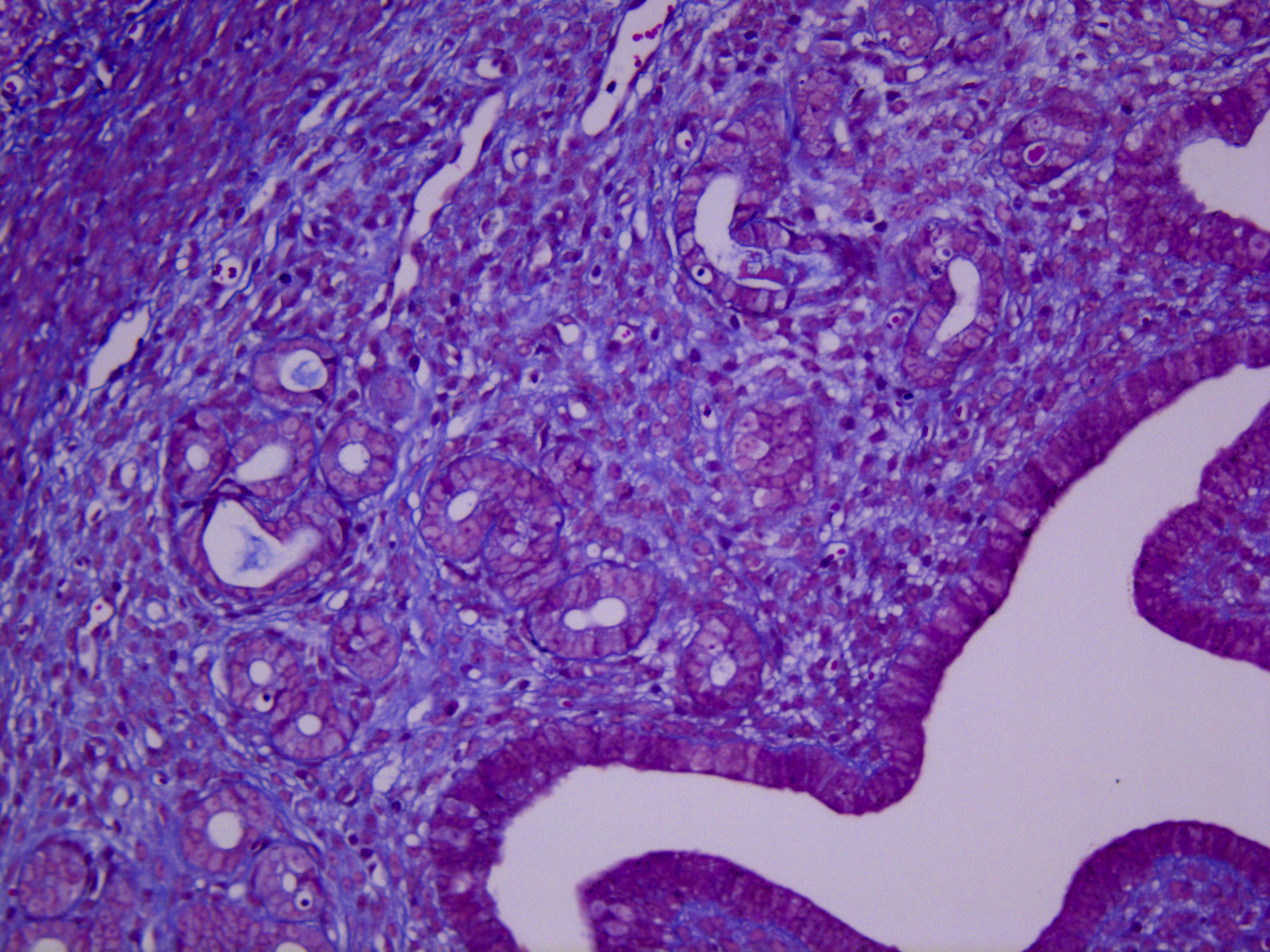

Supplement: Supplementary file 7 — Source Data for Figure 6 [file EMMM-15-e17601-s006.zip › Figure 6-1/6E/6E Masson Sham.tif]

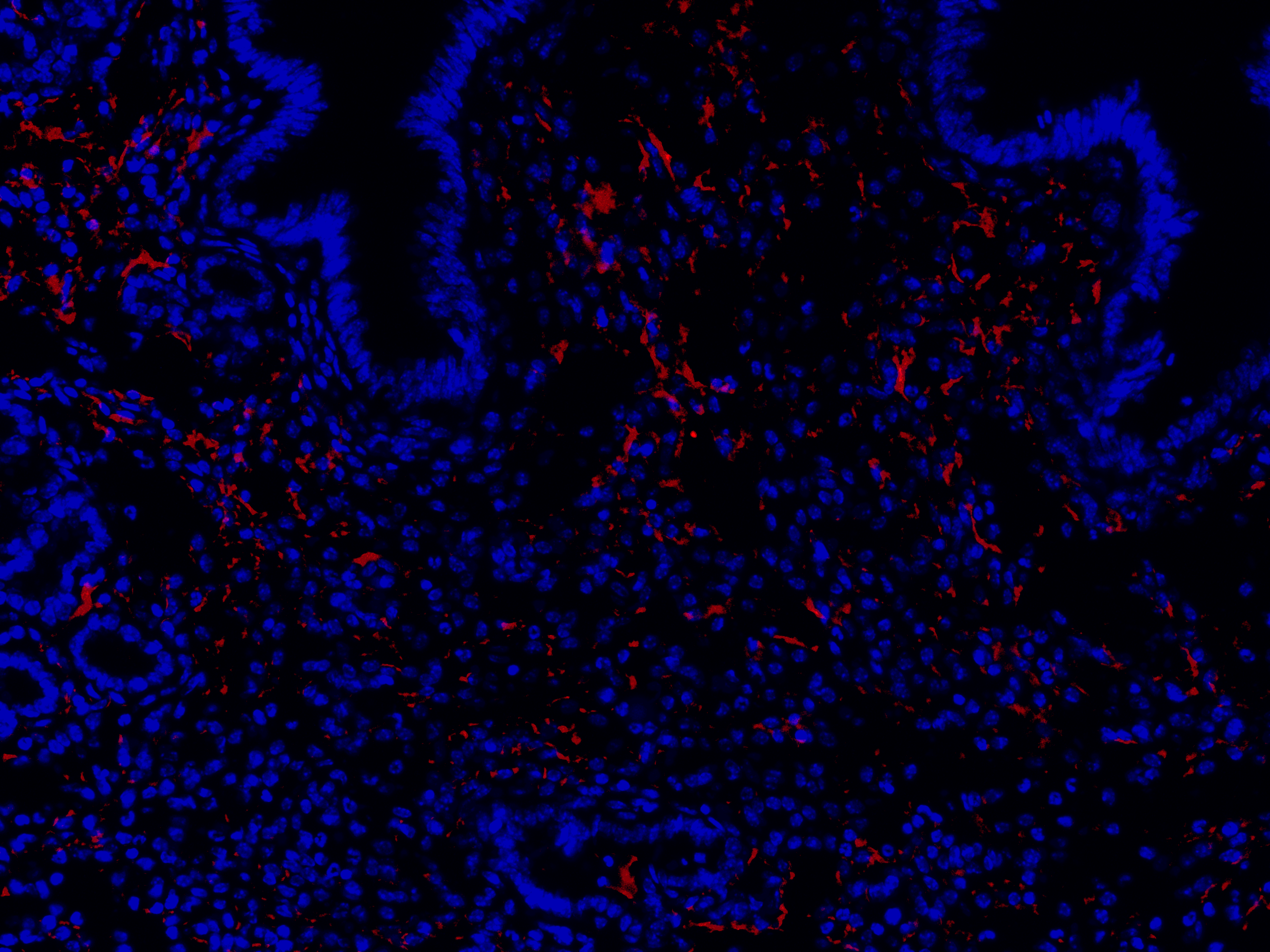

Supplement: Supplementary file 7 — Source Data for Figure 6 [file EMMM-15-e17601-s006.zip › Figure 6-1/6E/Collagen1 Sham.tif]

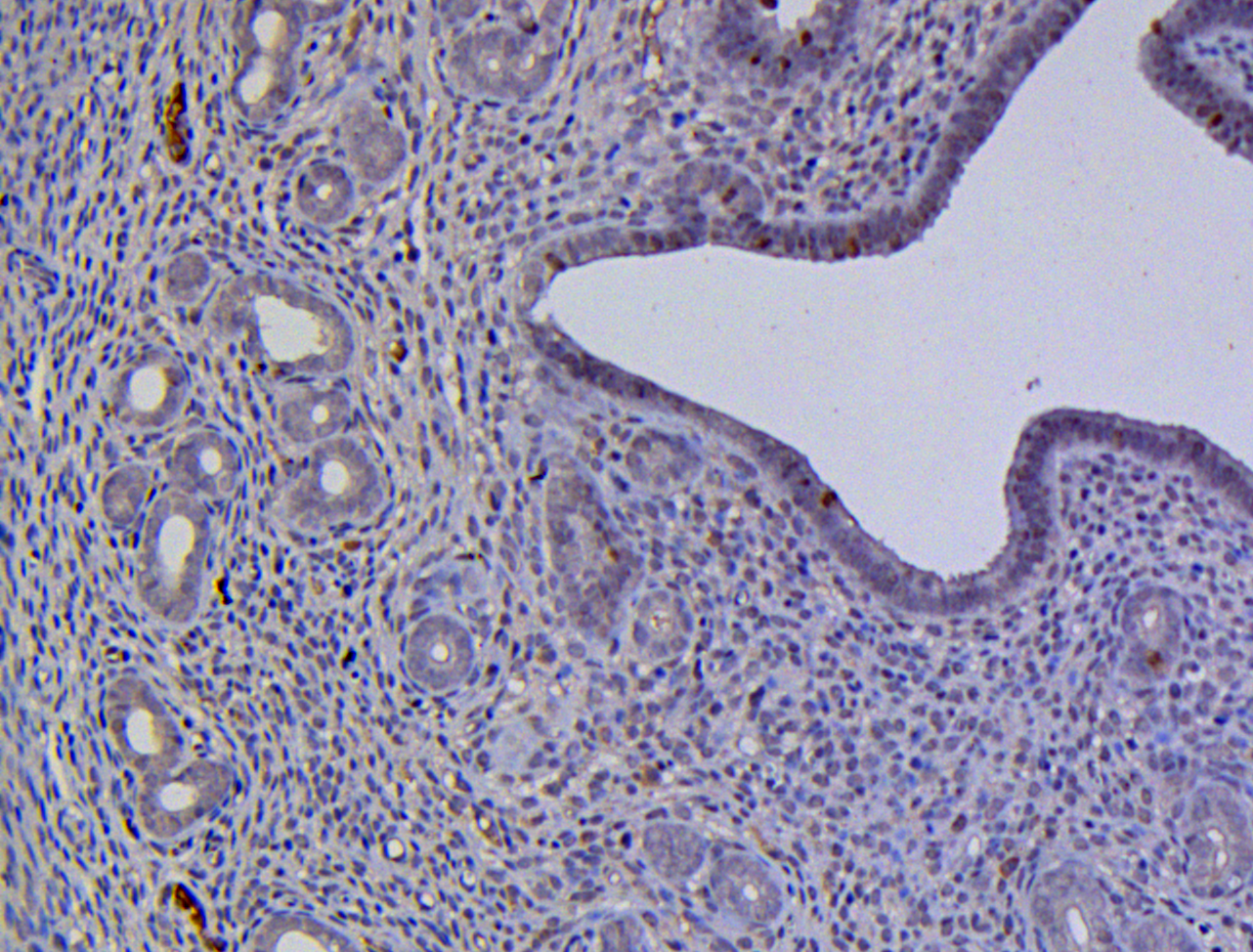

Supplement: Supplementary file 7 — Source Data for Figure 6 [file EMMM-15-e17601-s006.zip › Figure 6-1/6E/p-p65 Sham.tif]

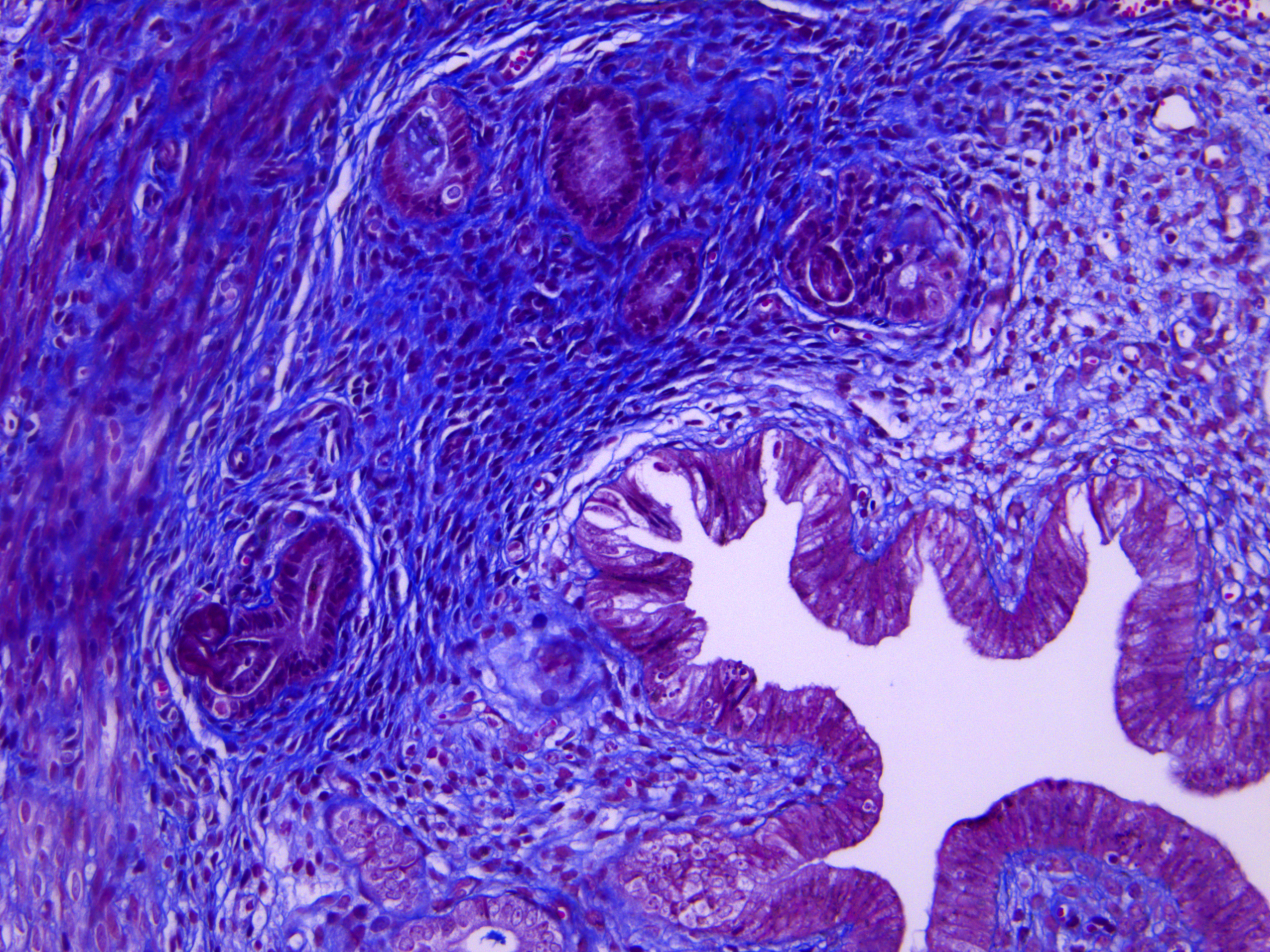

Supplement: Supplementary file 7 — Source Data for Figure 6 [file EMMM-15-e17601-s006.zip › Figure 6-1/6E/6E Masson IUA.tif]

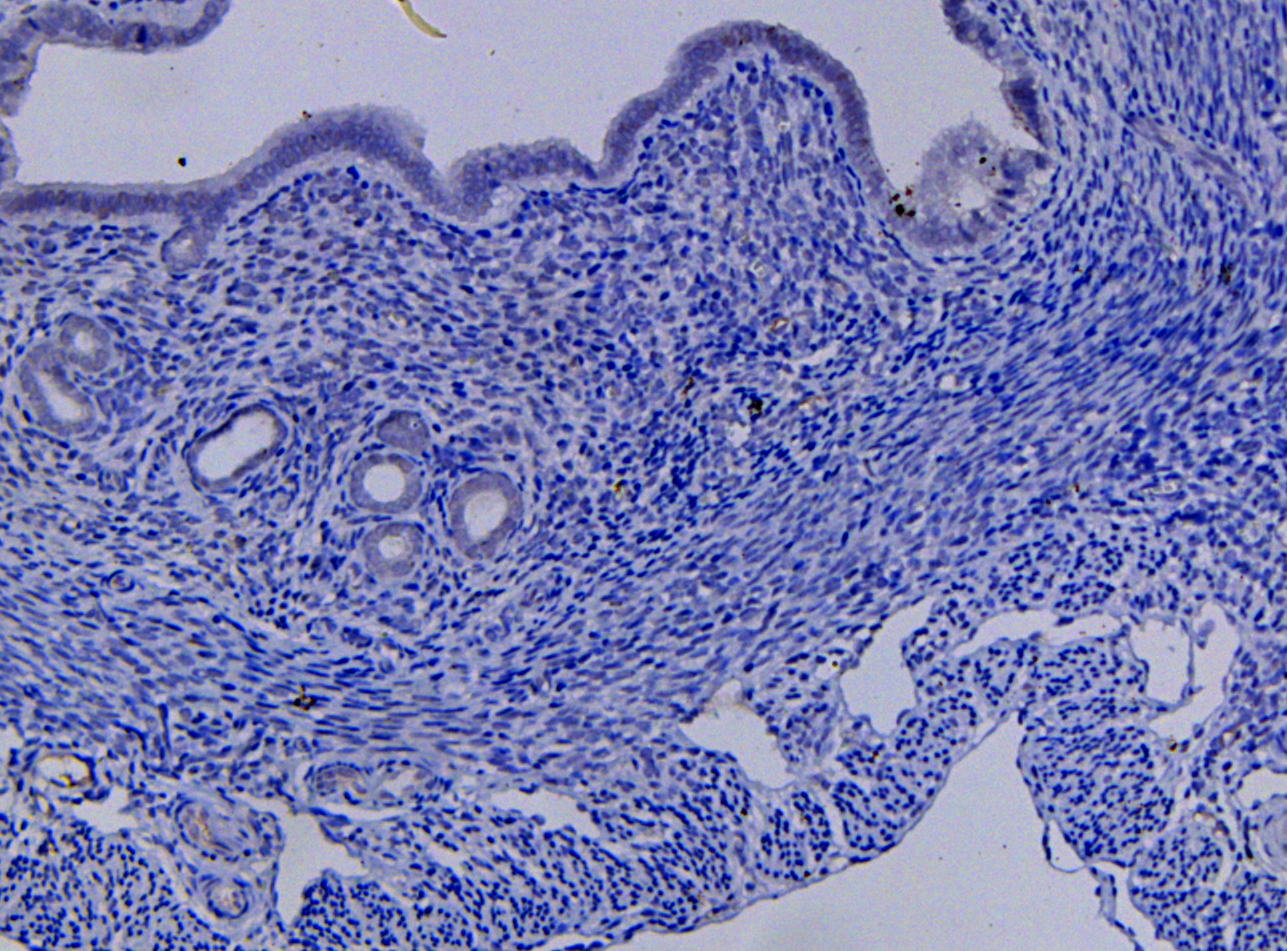

Supplement: Supplementary file 7 — Source Data for Figure 6 [file EMMM-15-e17601-s006.zip › Figure 6-1/6E/p-p65 IUA+DT.tif]

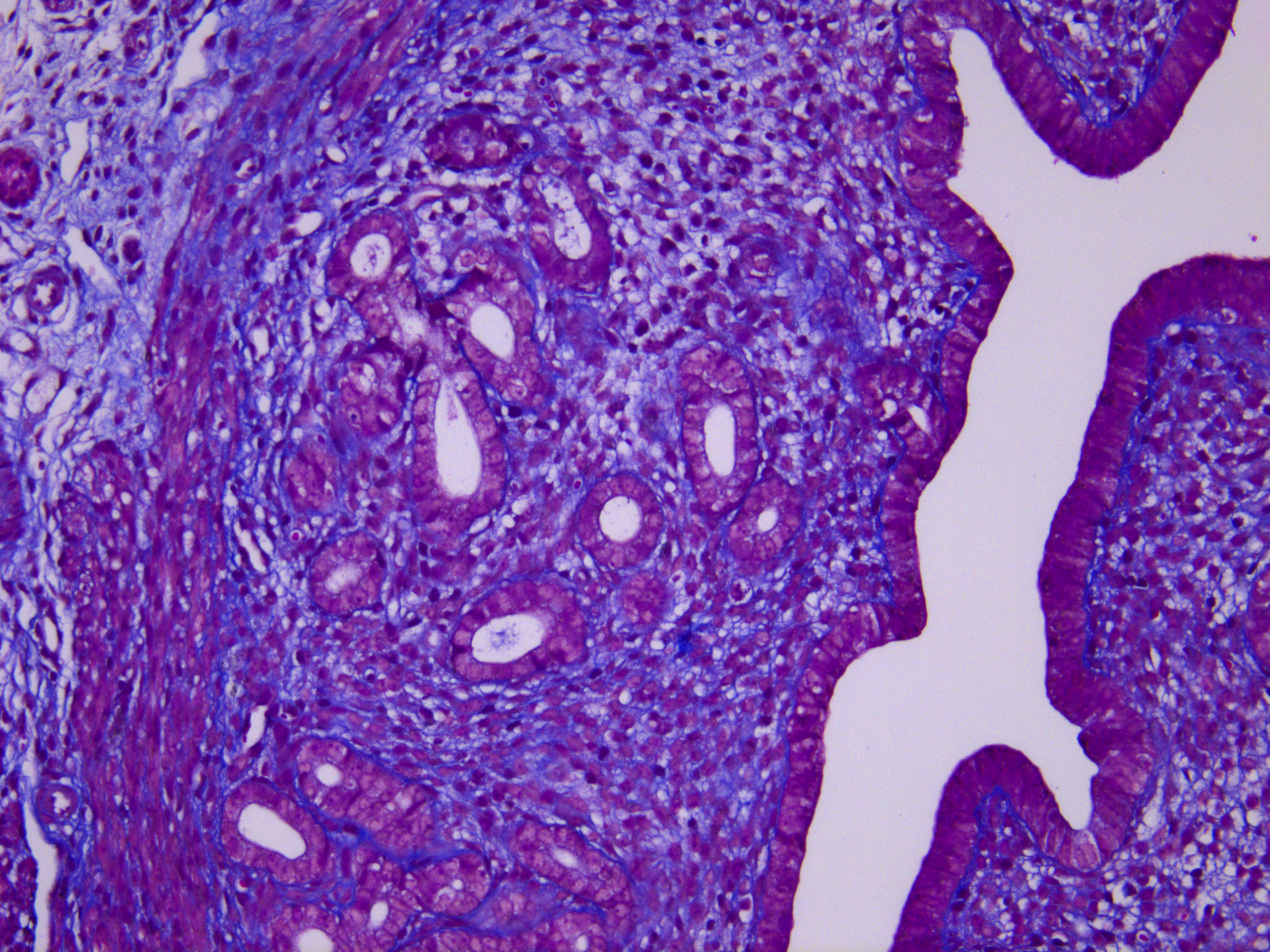

Supplement: Supplementary file 7 — Source Data for Figure 6 [file EMMM-15-e17601-s006.zip › Figure 6-1/6E/6E Masson IUA+DT.tif]

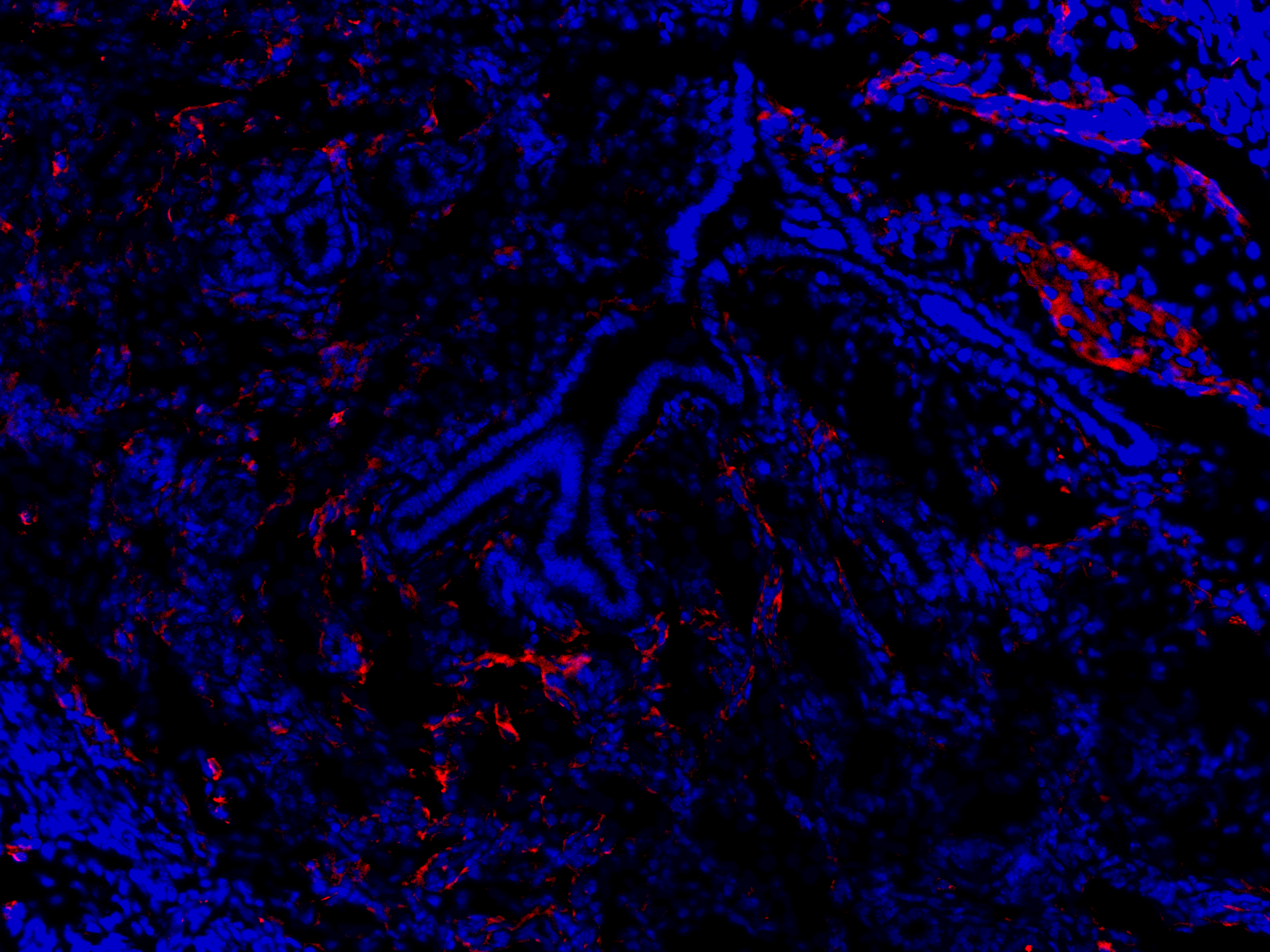

Supplement: Supplementary file 7 — Source Data for Figure 6 [file EMMM-15-e17601-s006.zip › Figure 6-1/6E/Collagen1 IUA+DT.tif]

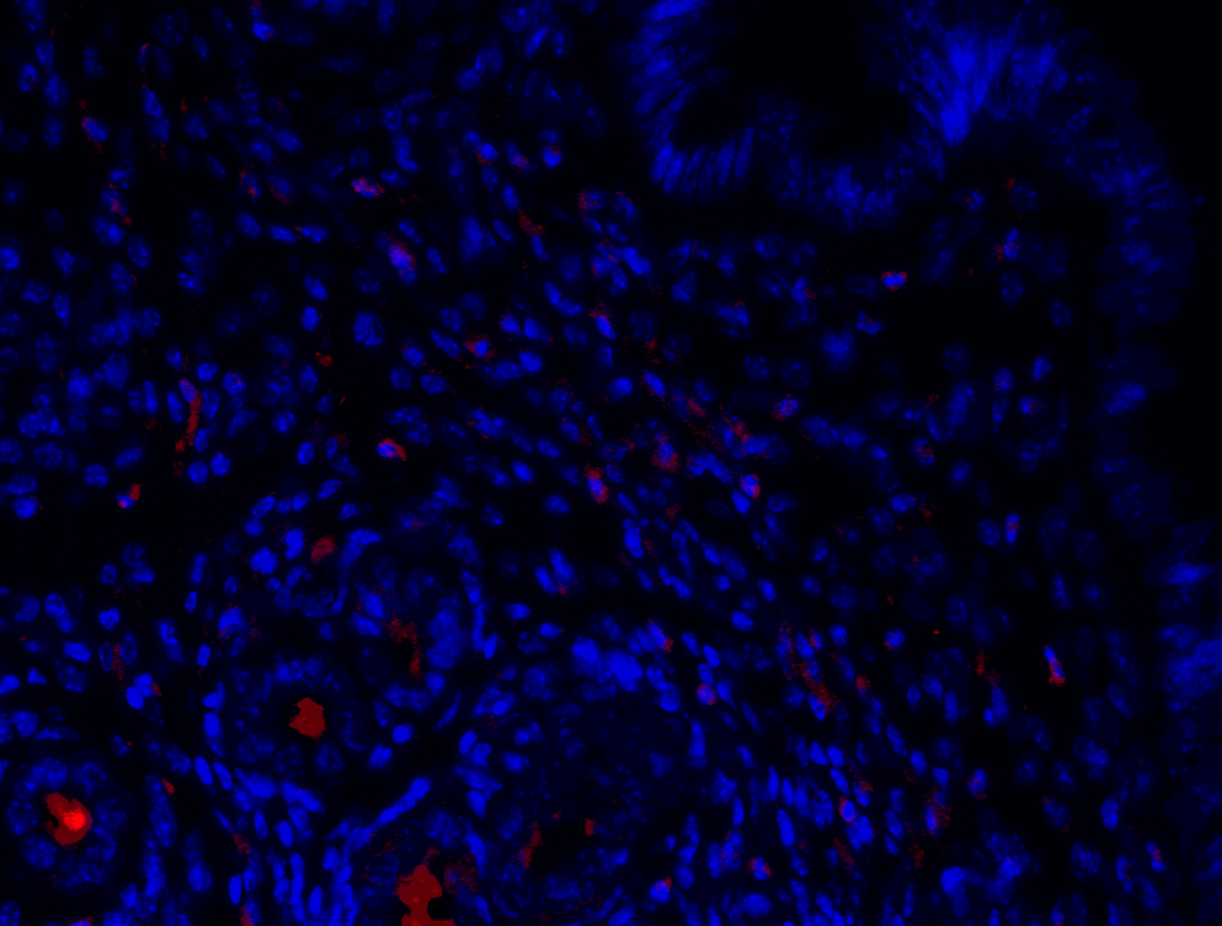

Supplement: Supplementary file 7 — Source Data for Figure 6 [file EMMM-15-e17601-s006.zip › Figure 6-1/6C/Sham GAS6.tif]

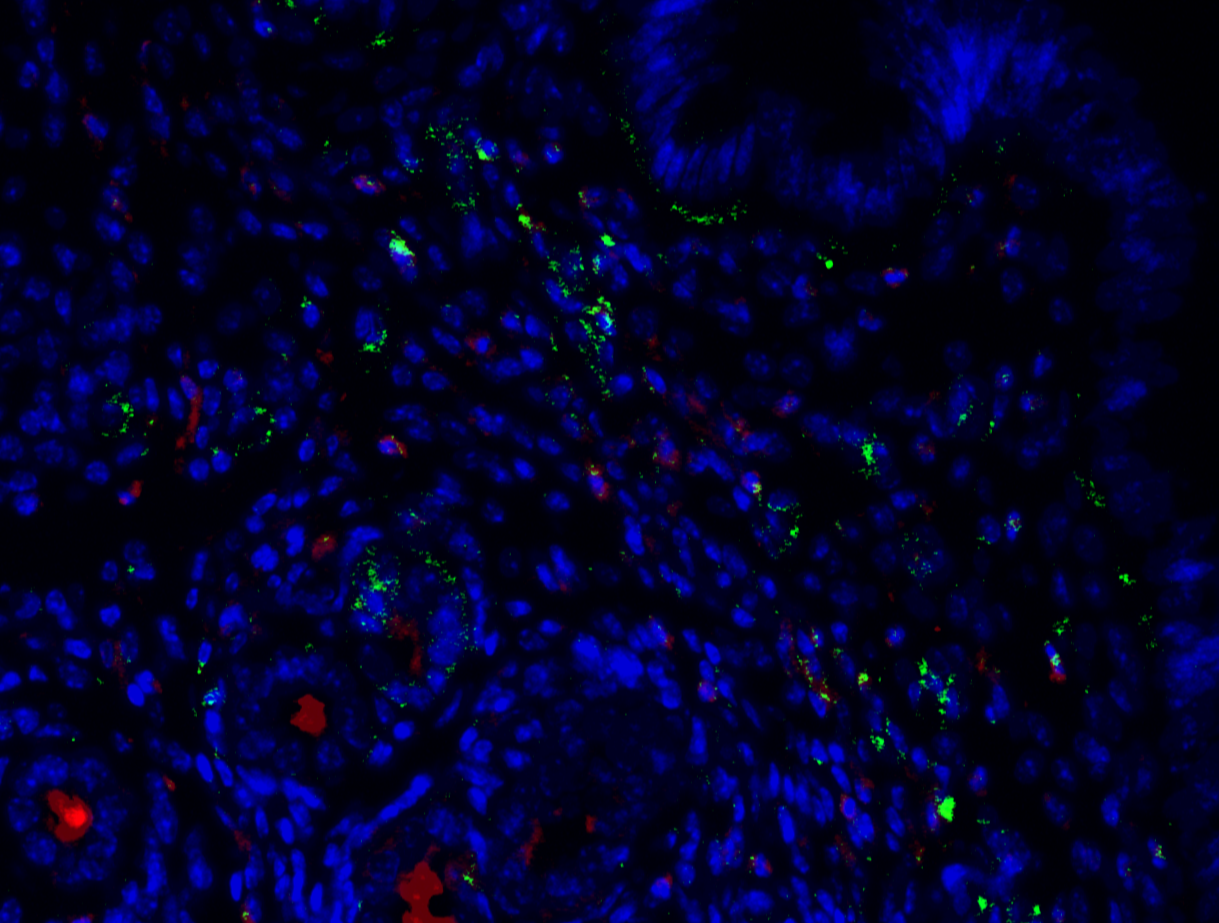

Supplement: Supplementary file 7 — Source Data for Figure 6 [file EMMM-15-e17601-s006.zip › Figure 6-1/6C/Sham GAS6-GFP.tif]
