# Supplementary figures and images for: Targeting CD301 + macrophages inhibits endometrial fibrosis and improves pregnancy outcome (part 2 of 2)
Source: EMBO Mol Med. 2023 Jul 31;15(9):e17601. doi: 10.15252/emmm.202317601 (PMC10493587; doi:10.15252/emmm.202317601)

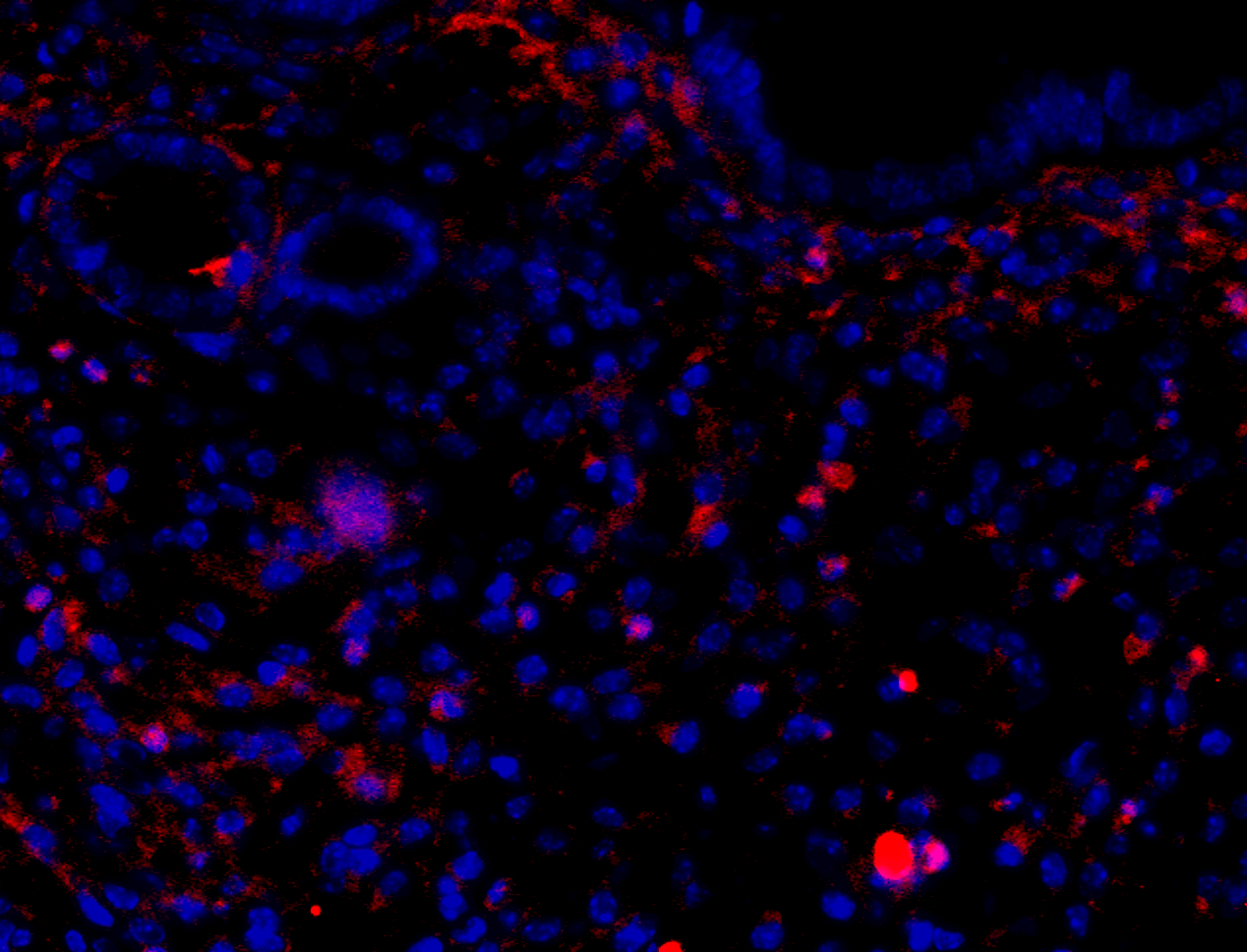

Supplement: Supplementary file 7 — Source Data for Figure 6 [file EMMM-15-e17601-s006.zip › Figure 6-1/6C/IUA GAS6.tif]

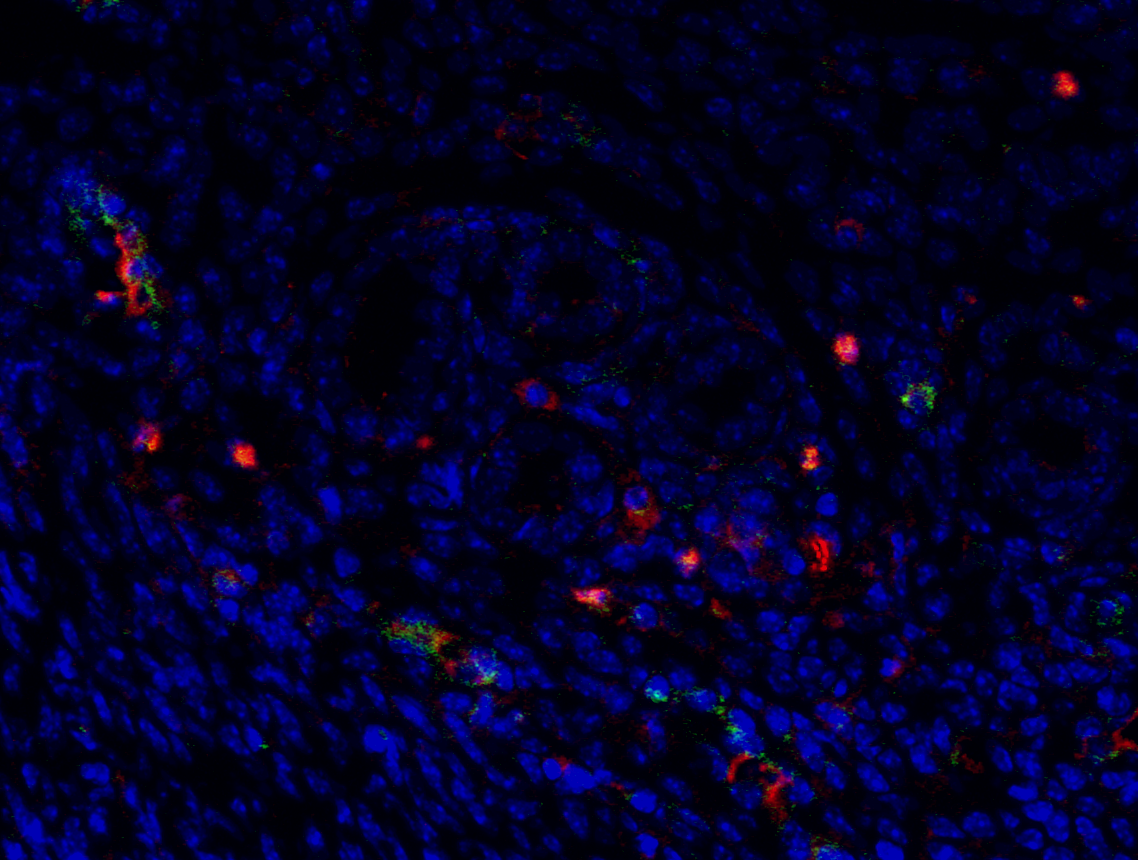

Supplement: Supplementary file 7 — Source Data for Figure 6 [file EMMM-15-e17601-s006.zip › Figure 6-1/6C/IUA+DT GAS6-GFP.tif]

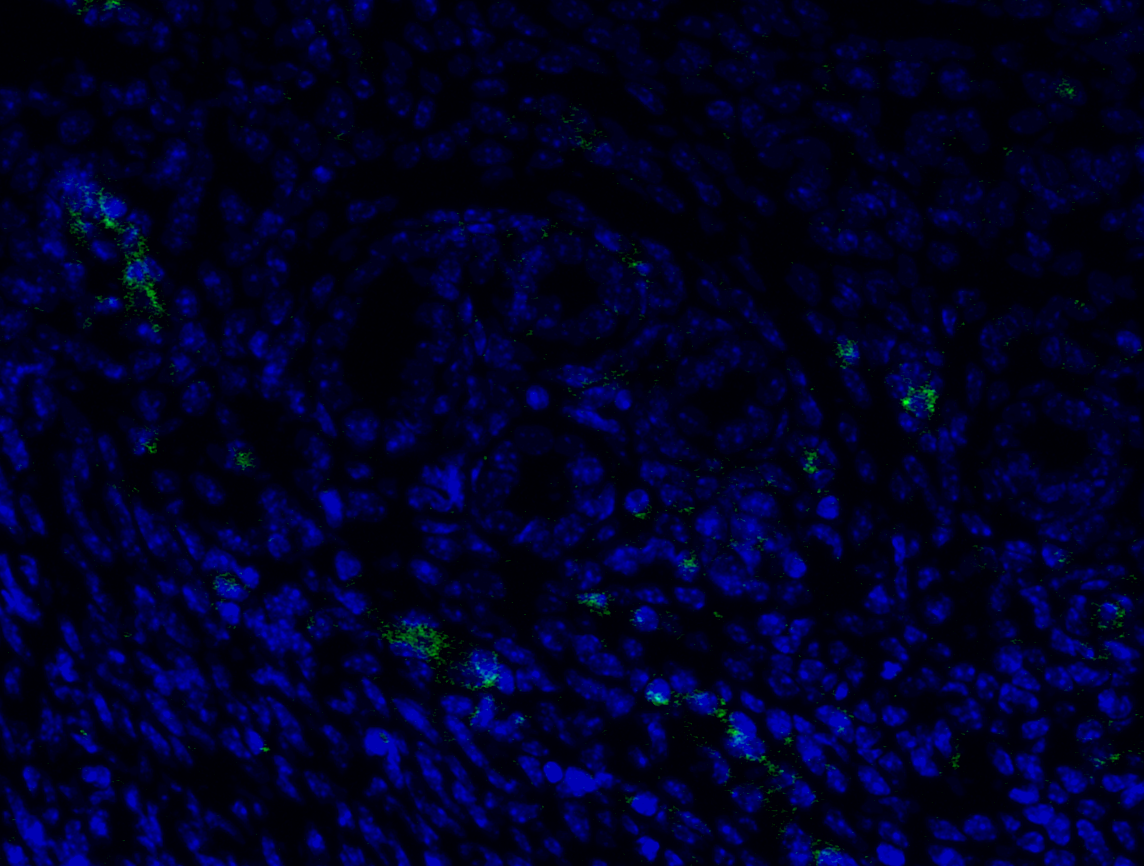

Supplement: Supplementary file 7 — Source Data for Figure 6 [file EMMM-15-e17601-s006.zip › Figure 6-1/6C/IUA+DT GFP.tif]

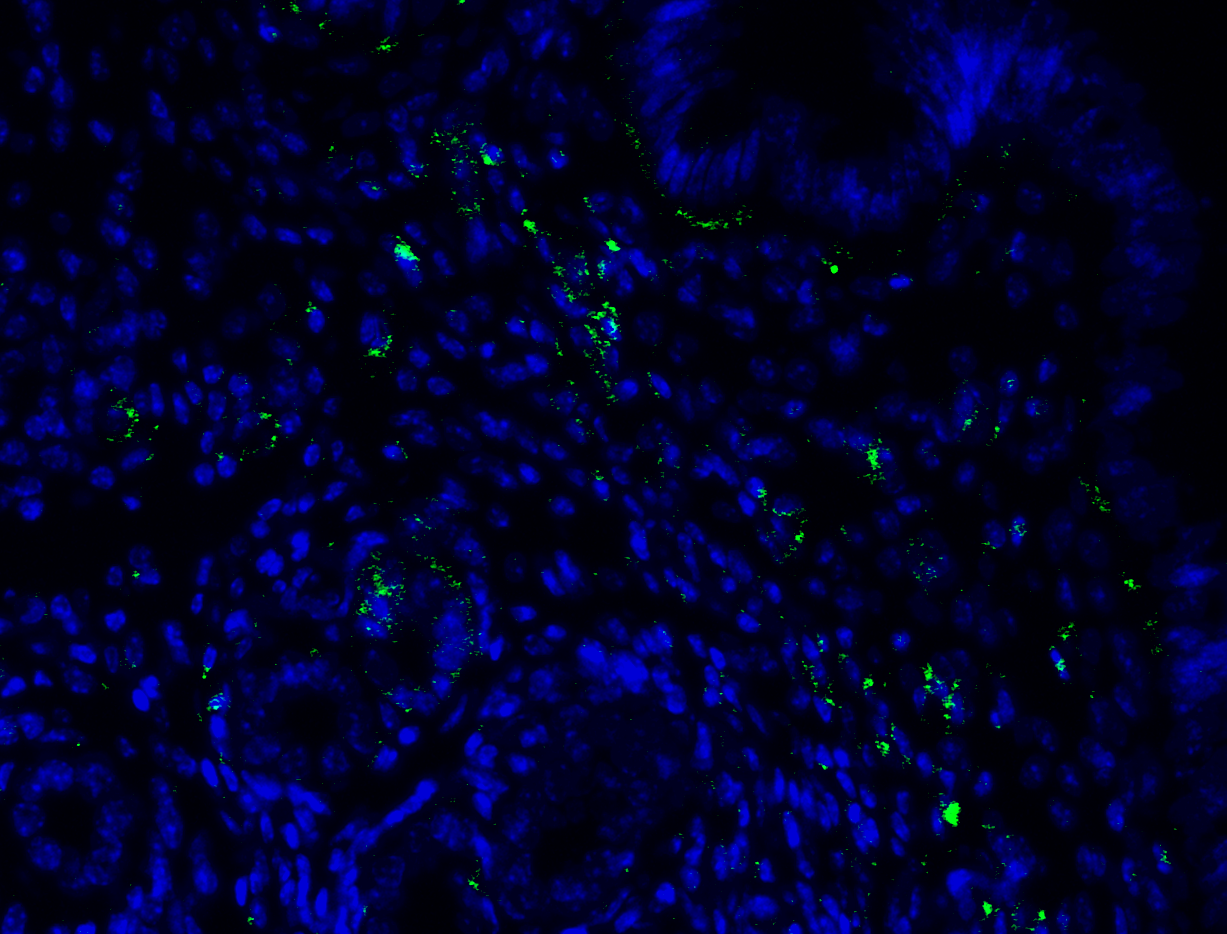

Supplement: Supplementary file 7 — Source Data for Figure 6 [file EMMM-15-e17601-s006.zip › Figure 6-1/6C/Sham GFP.tif]

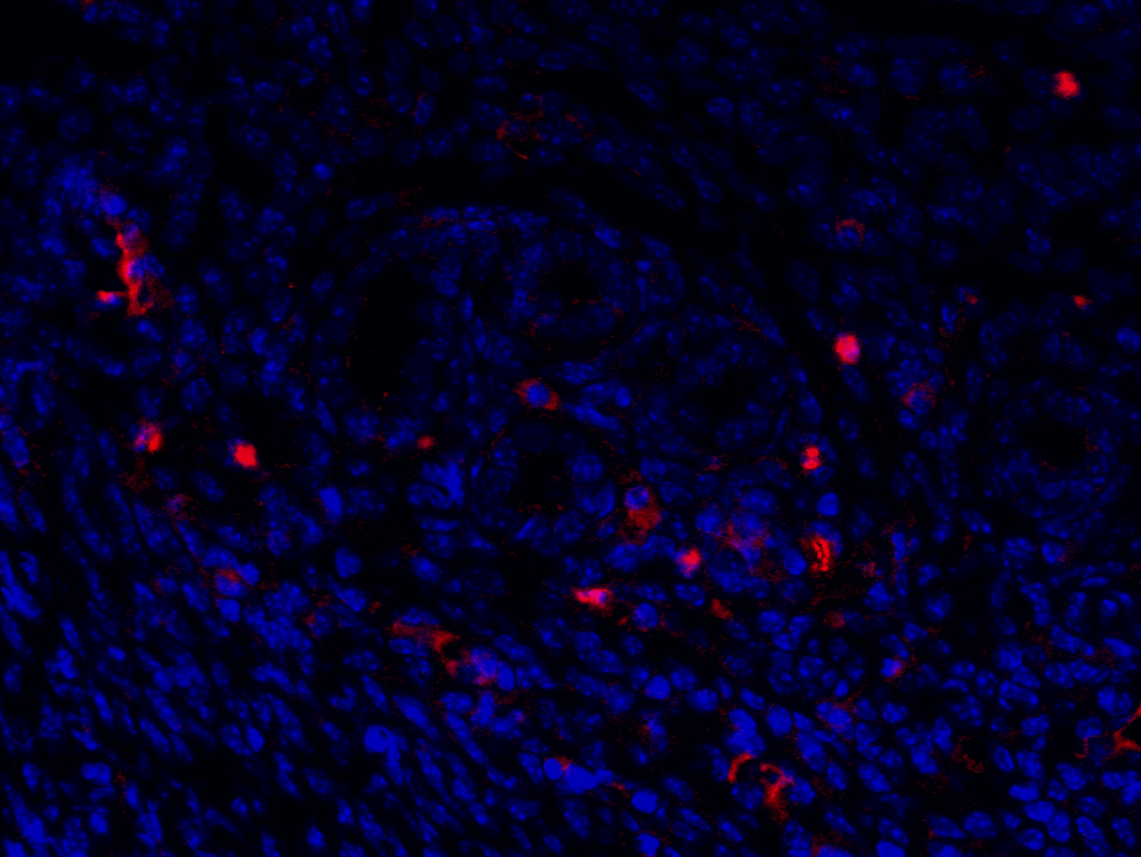

Supplement: Supplementary file 7 — Source Data for Figure 6 [file EMMM-15-e17601-s006.zip › Figure 6-1/6C/IUA+DT GAS6.tif]

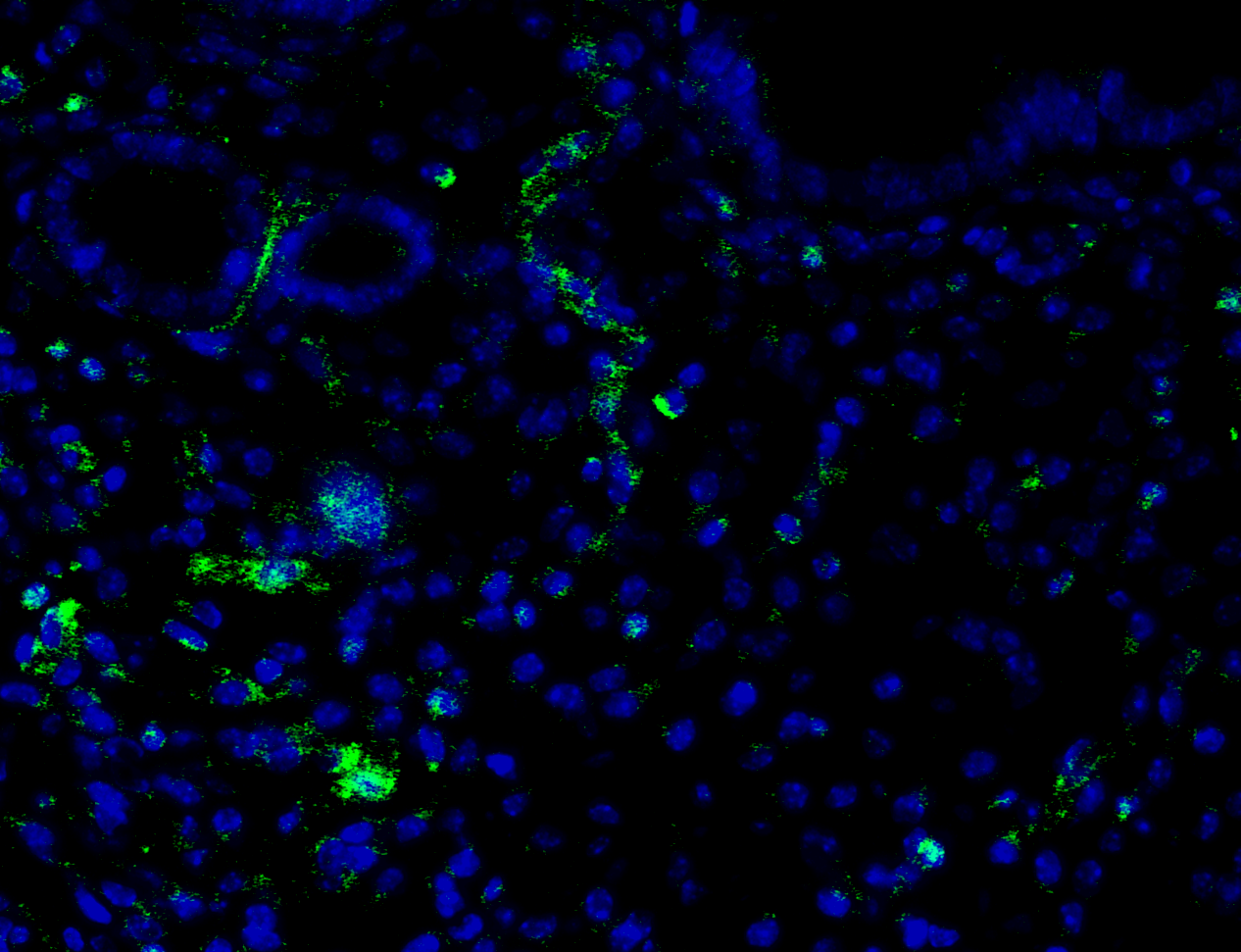

Supplement: Supplementary file 7 — Source Data for Figure 6 [file EMMM-15-e17601-s006.zip › Figure 6-1/6C/IUA GFP.tif]

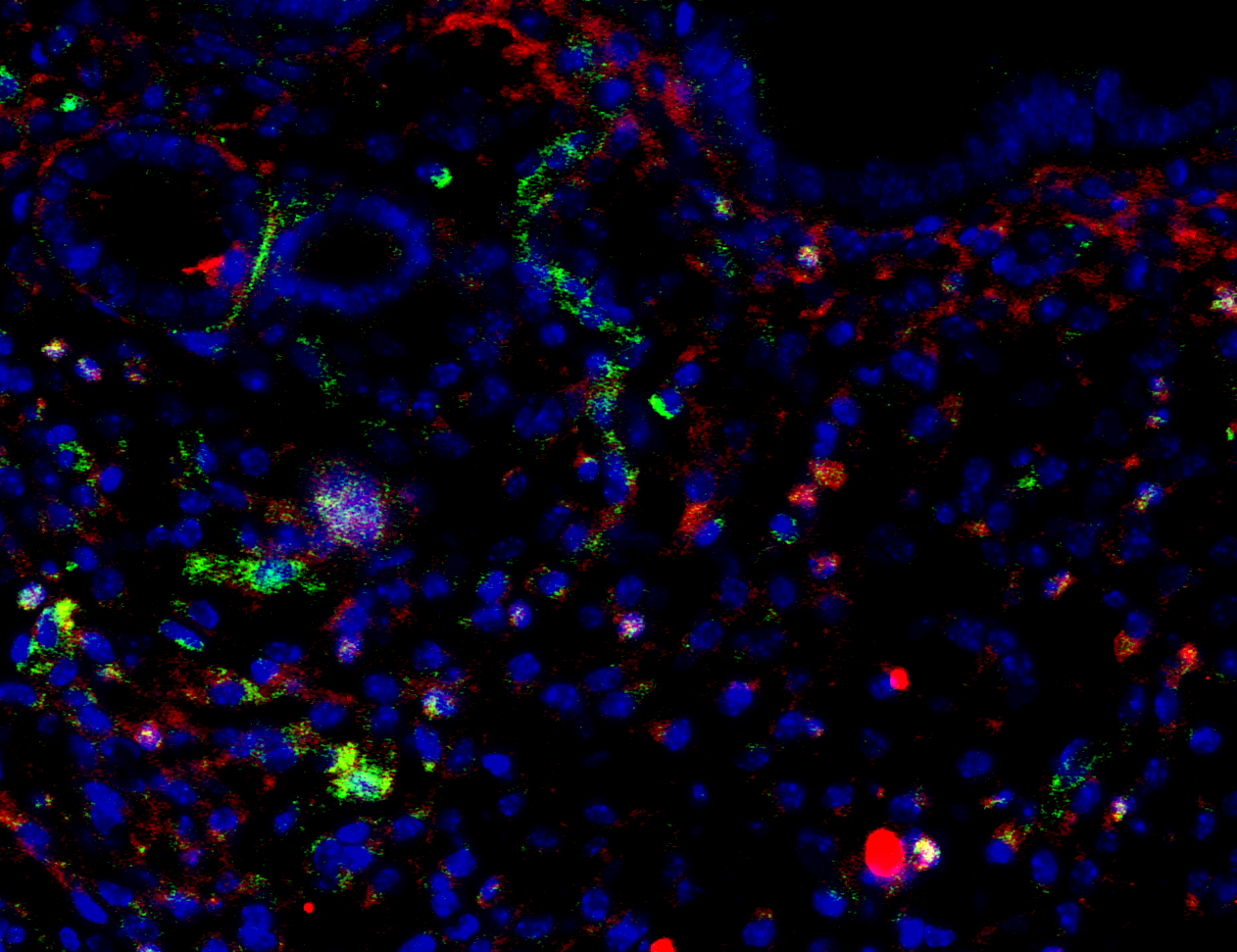

Supplement: Supplementary file 7 — Source Data for Figure 6 [file EMMM-15-e17601-s006.zip › Figure 6-1/6C/IUA GAS6-GFP.tif]

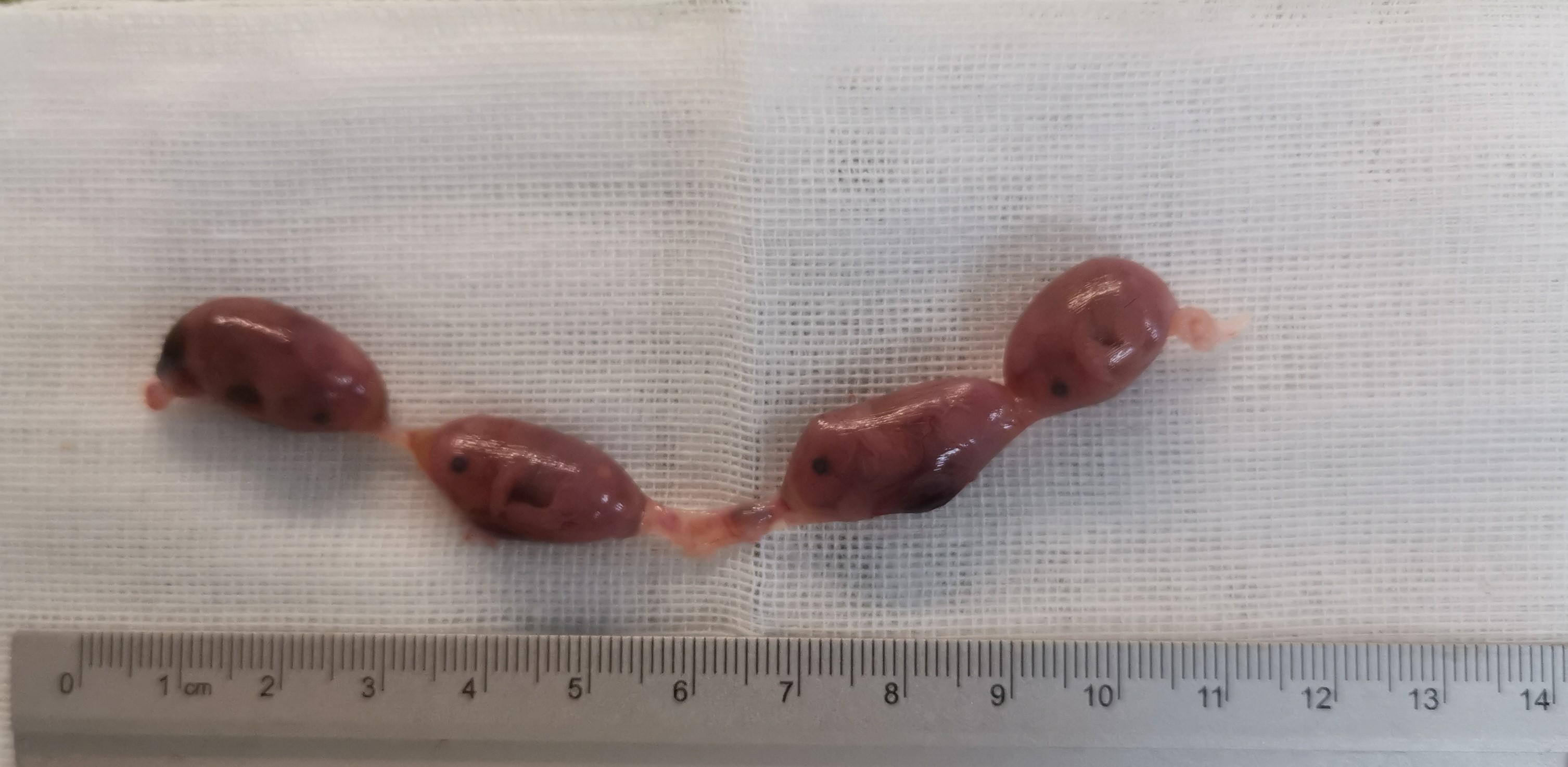

Supplement: Supplementary file 8 — Source Data for Figure 7 [file EMMM-15-e17601-s001.zip › Figure 7-1/7D/IUA+PBS-1.jpg]

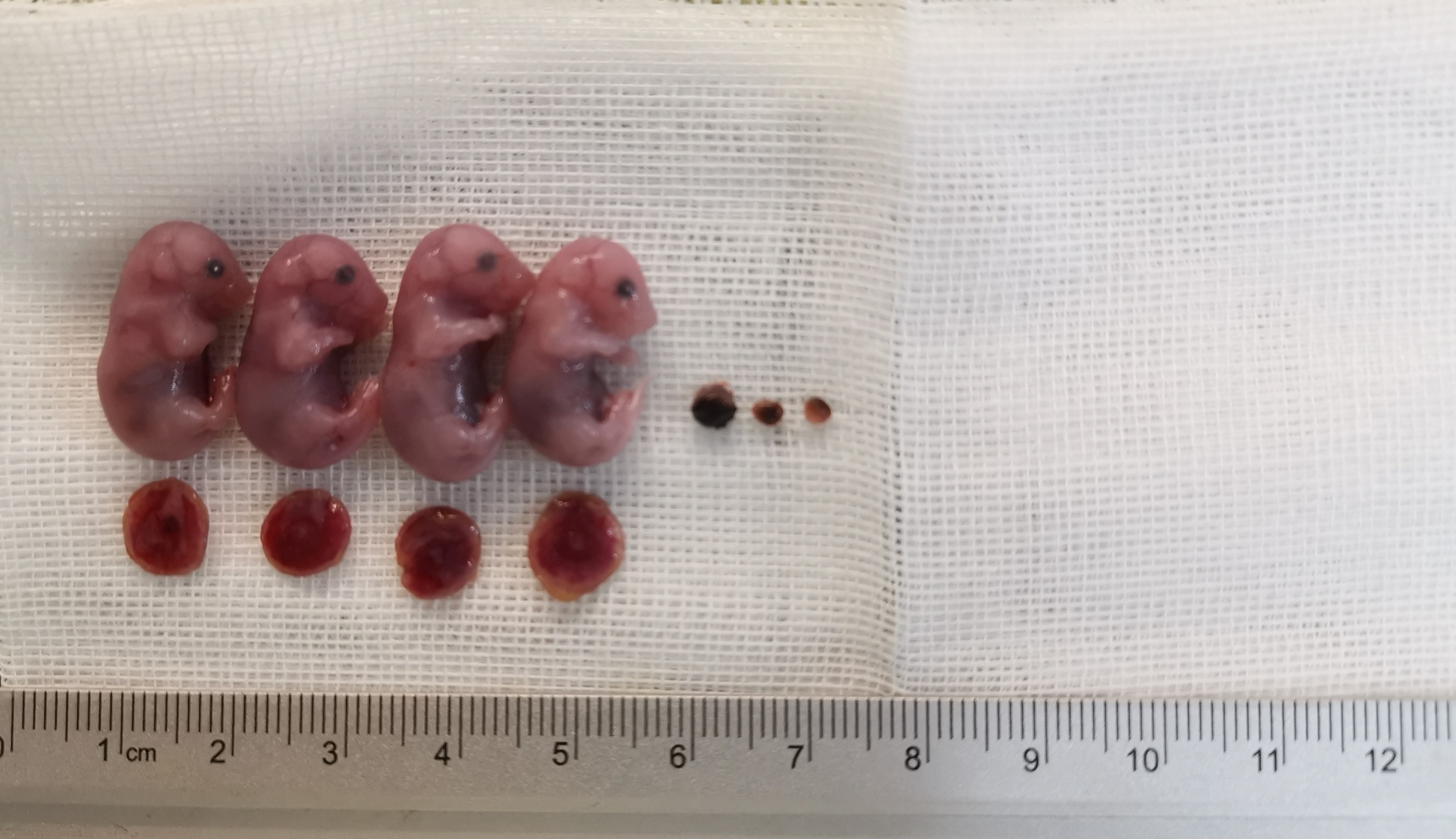

Supplement: Supplementary file 8 — Source Data for Figure 7 [file EMMM-15-e17601-s001.zip › Figure 7-1/7D/IUA+PBS-2.jpg]

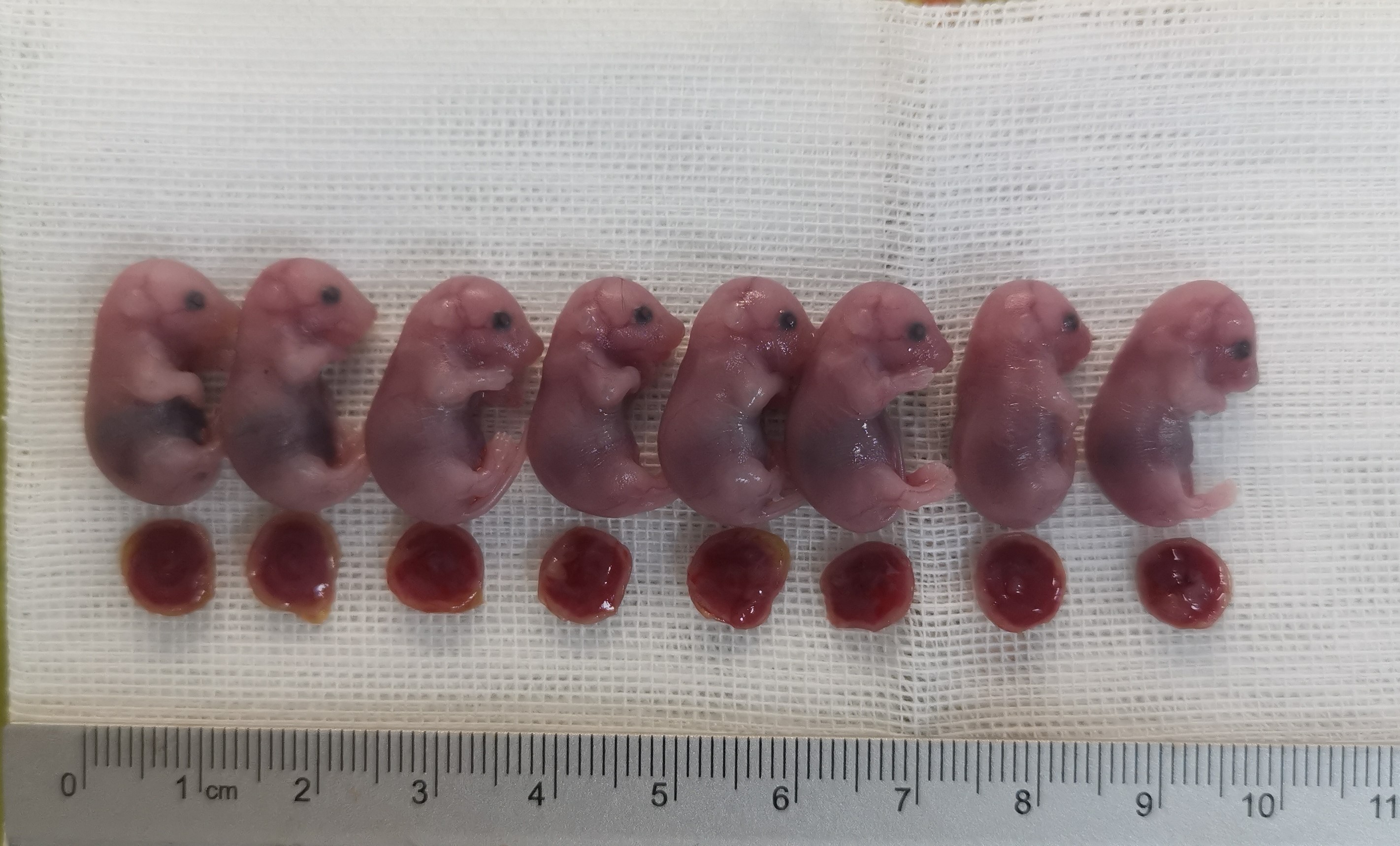

Supplement: Supplementary file 8 — Source Data for Figure 7 [file EMMM-15-e17601-s001.zip › Figure 7-1/7D/IUA+BEM-2.jpg]

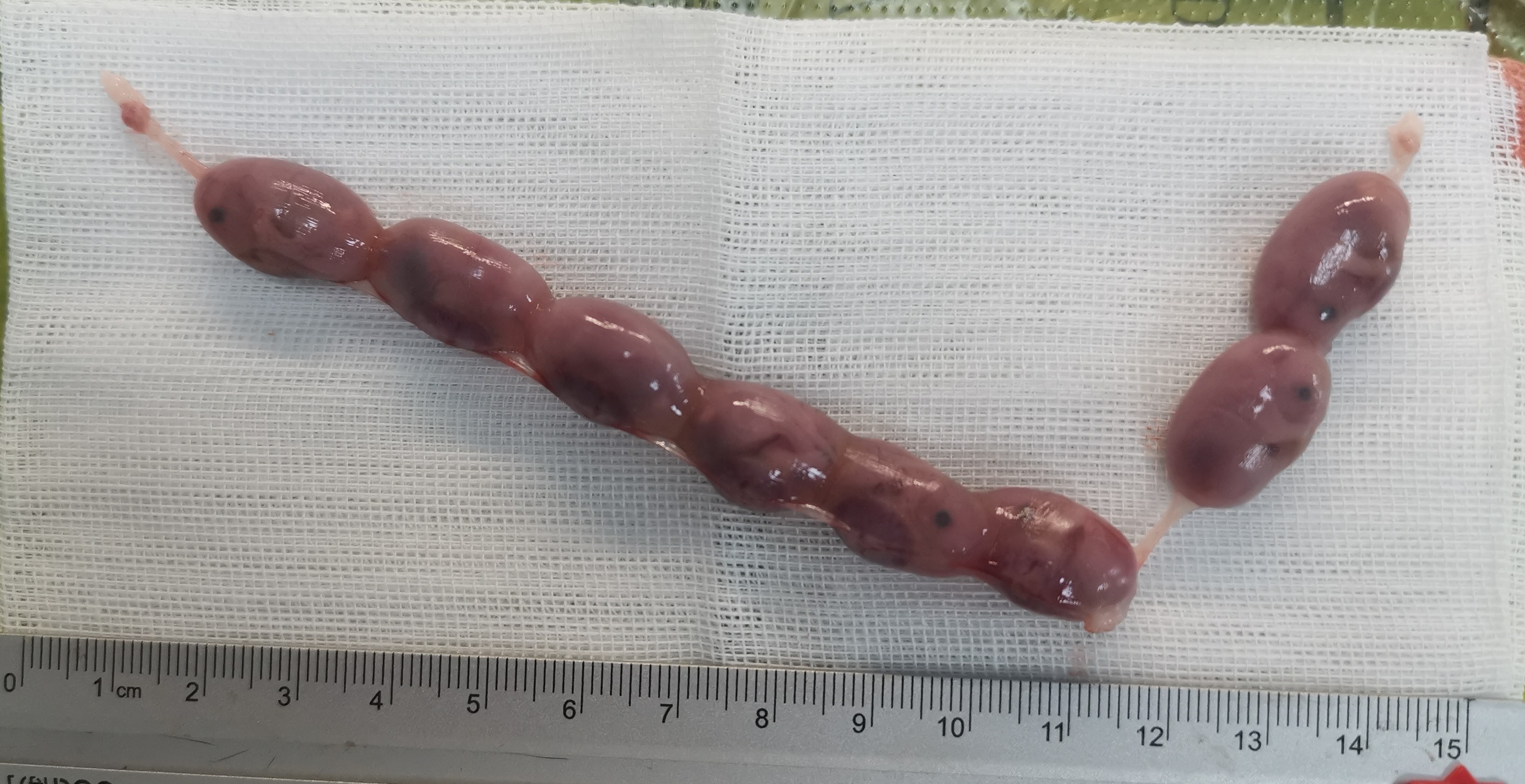

Supplement: Supplementary file 8 — Source Data for Figure 7 [file EMMM-15-e17601-s001.zip › Figure 7-1/7D/IUA+BEM-1.jpg]

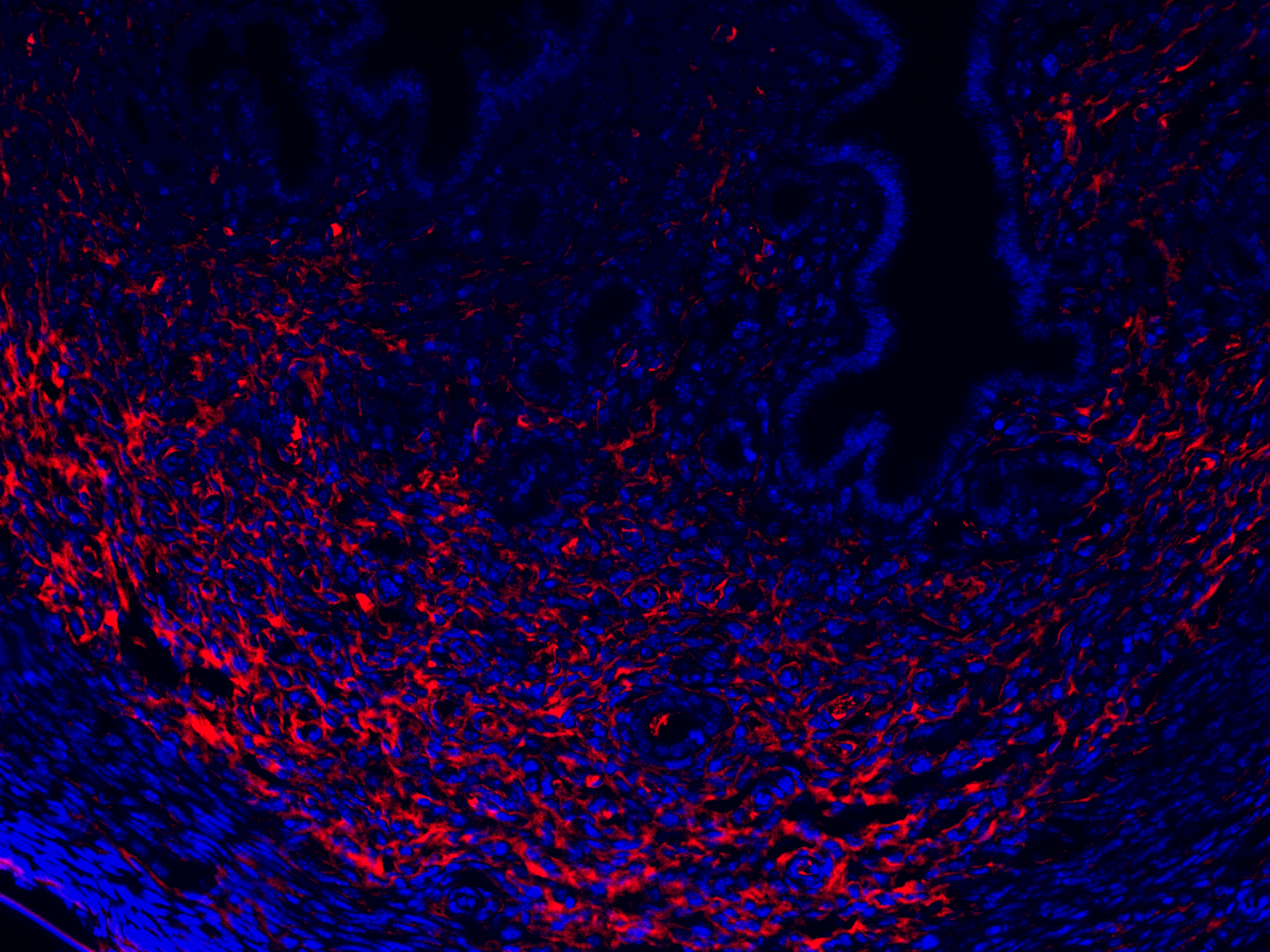

Supplement: Supplementary file 8 — Source Data for Figure 7 [file EMMM-15-e17601-s001.zip › Figure 7-1/7C/Collagen 1 IUA+PBS.tif]

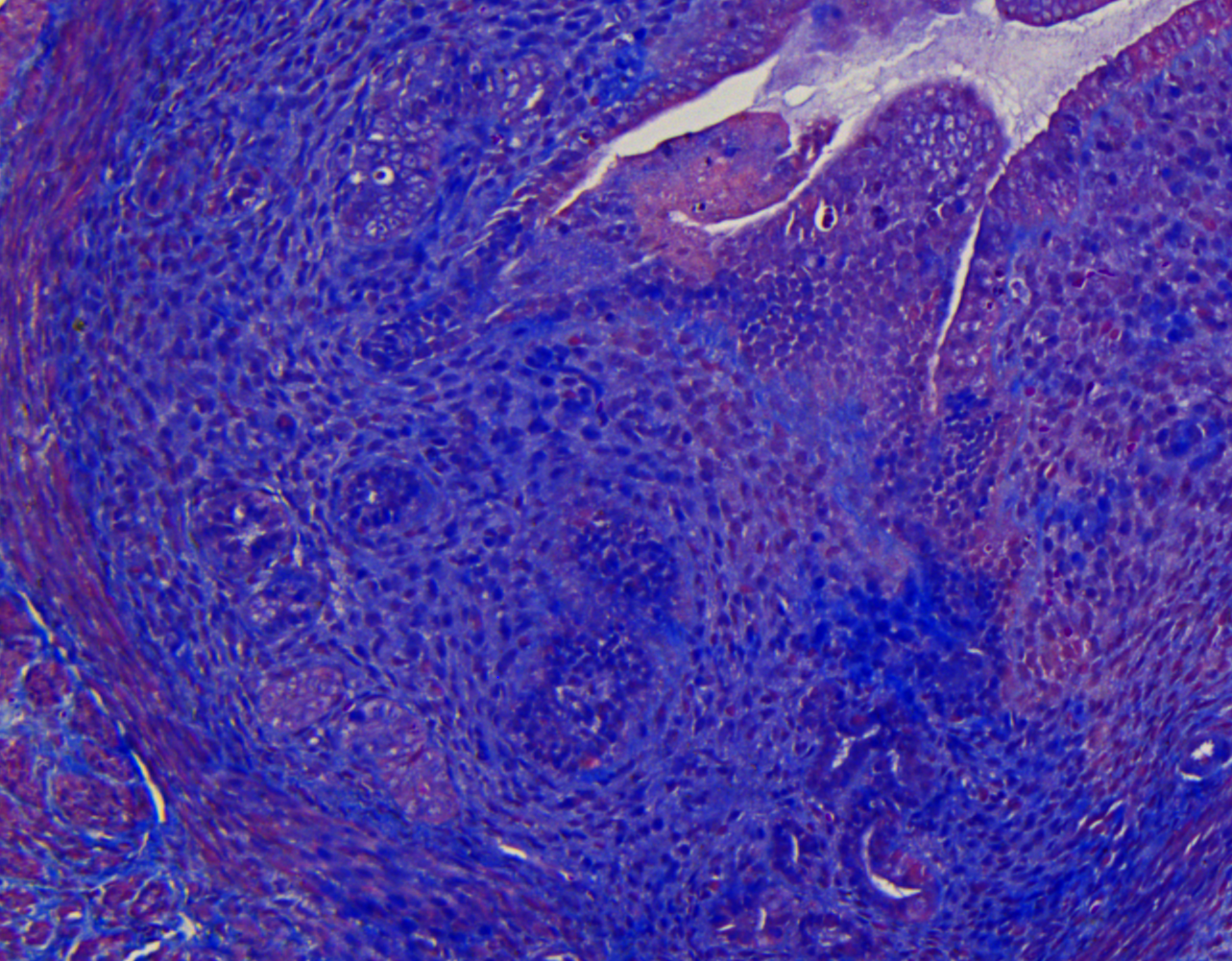

Supplement: Supplementary file 8 — Source Data for Figure 7 [file EMMM-15-e17601-s001.zip › Figure 7-1/7C/Masson IUA+PBS.tif]

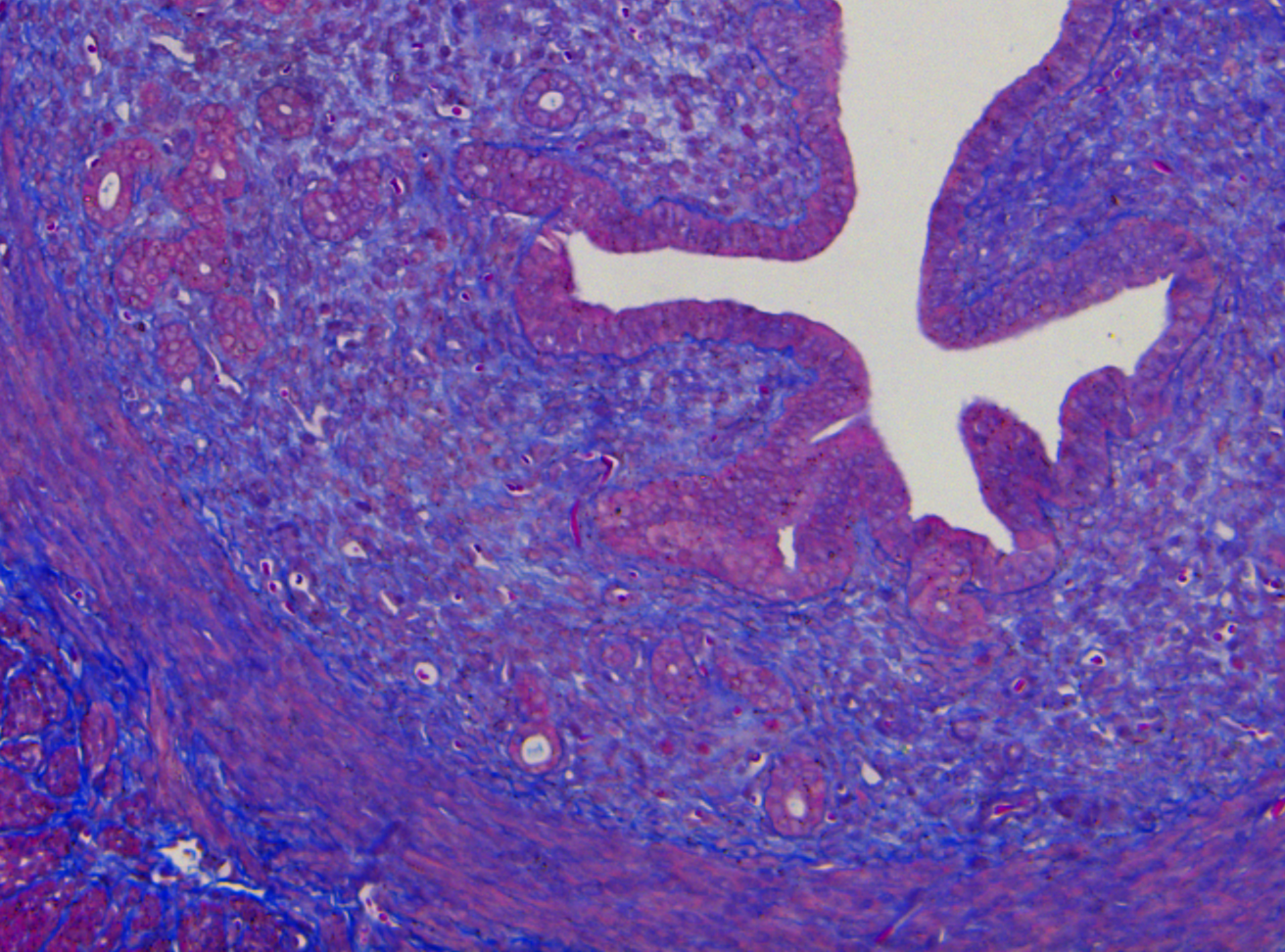

Supplement: Supplementary file 8 — Source Data for Figure 7 [file EMMM-15-e17601-s001.zip › Figure 7-1/7C/Masson IUA+BEM.tif]

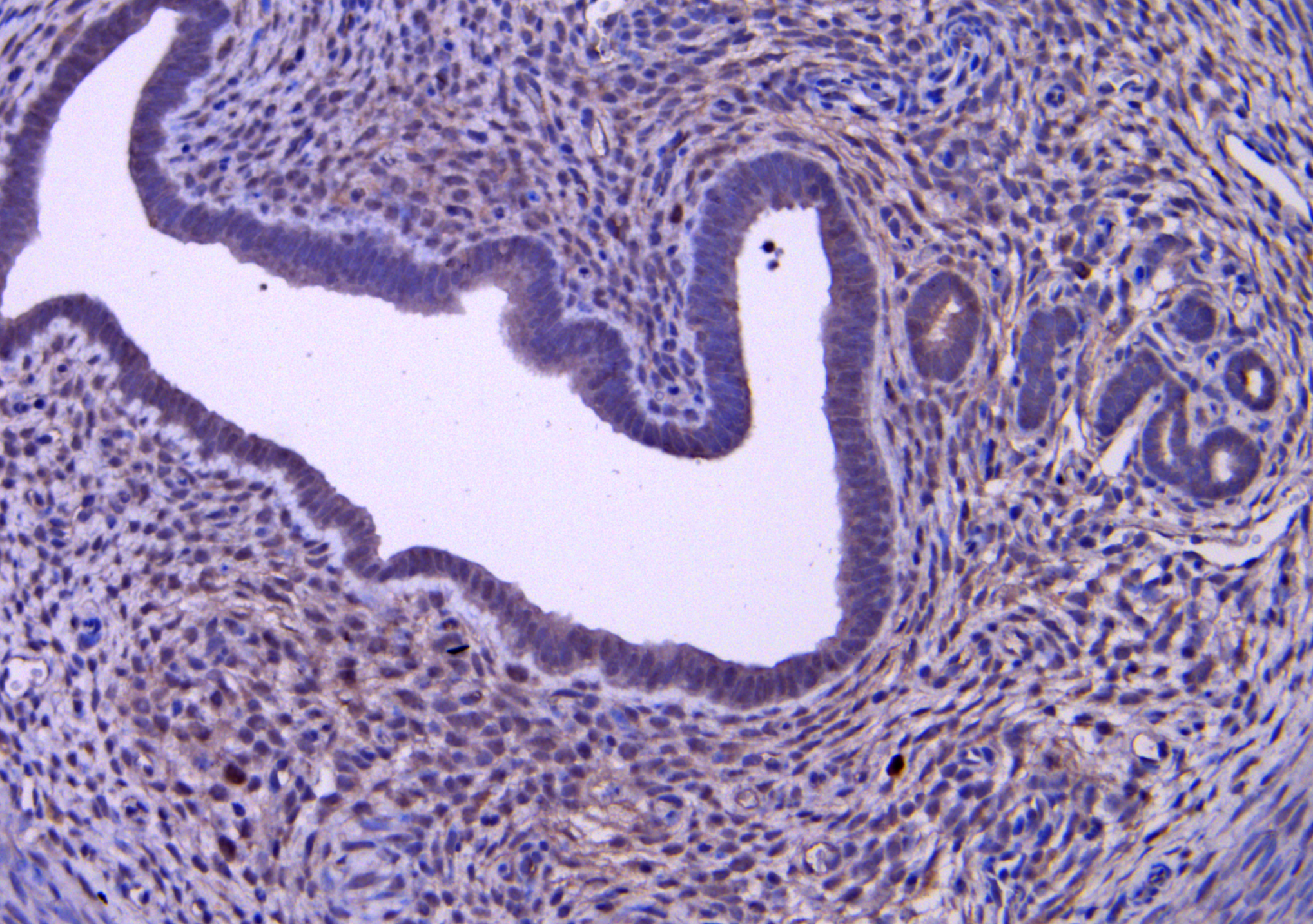

Supplement: Supplementary file 8 — Source Data for Figure 7 [file EMMM-15-e17601-s001.zip › Figure 7-1/7C/p-p65 IUA+PBS.tif]

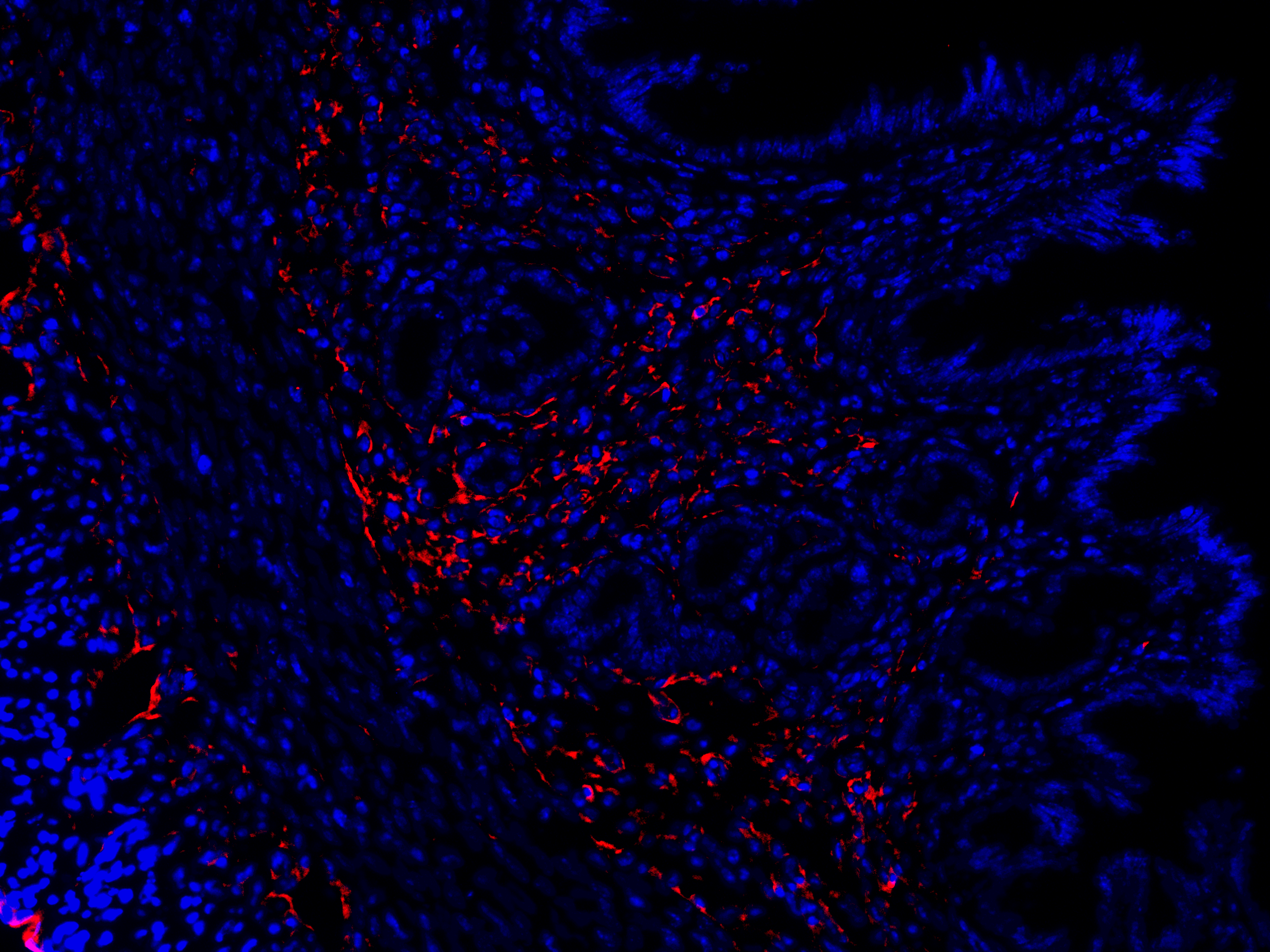

Supplement: Supplementary file 8 — Source Data for Figure 7 [file EMMM-15-e17601-s001.zip › Figure 7-1/7C/Collagen1 IUA+BEM.tif]

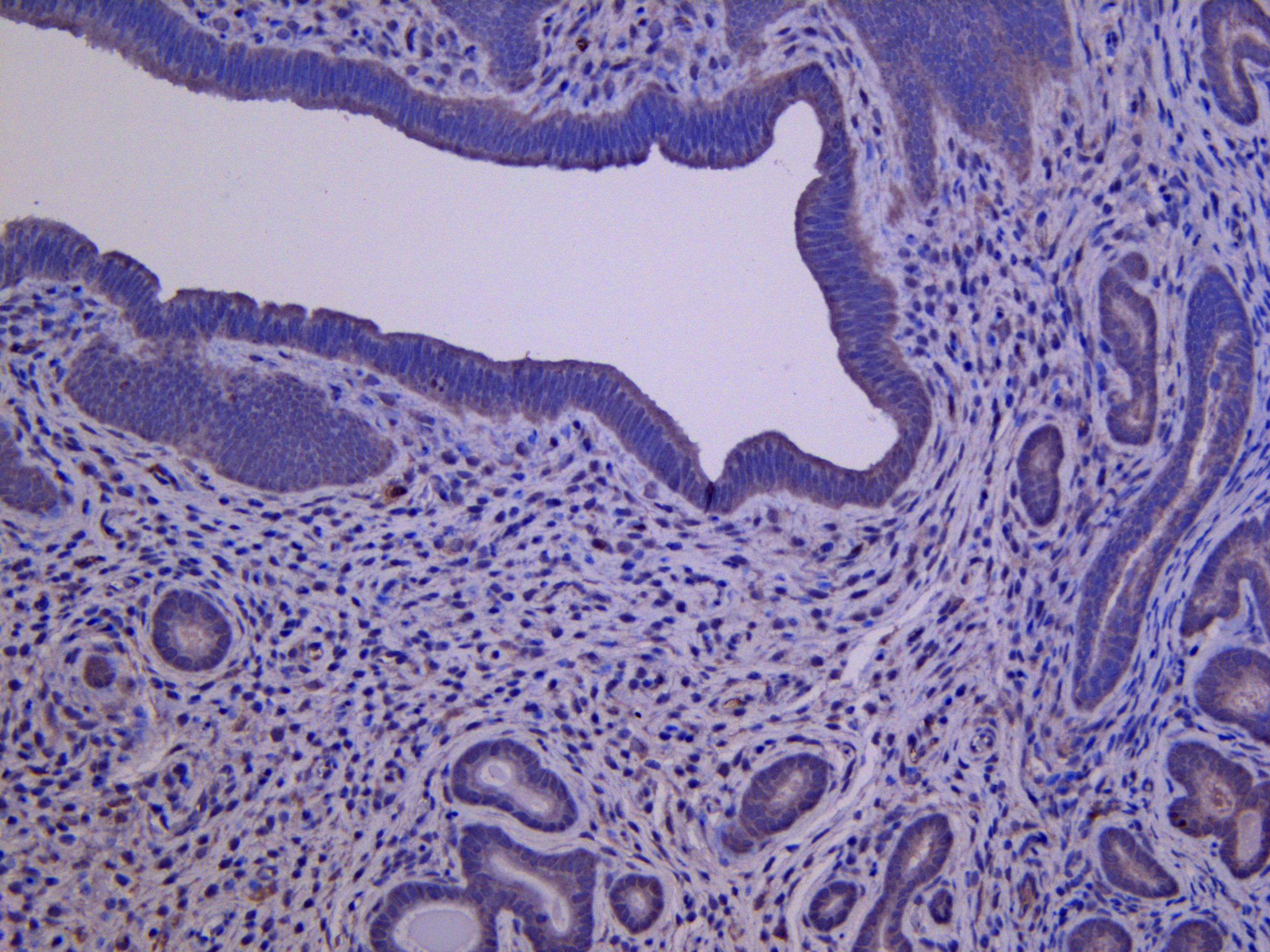

Supplement: Supplementary file 8 — Source Data for Figure 7 [file EMMM-15-e17601-s001.zip › Figure 7-1/7C/p-p65 IUA+BEM.tif]

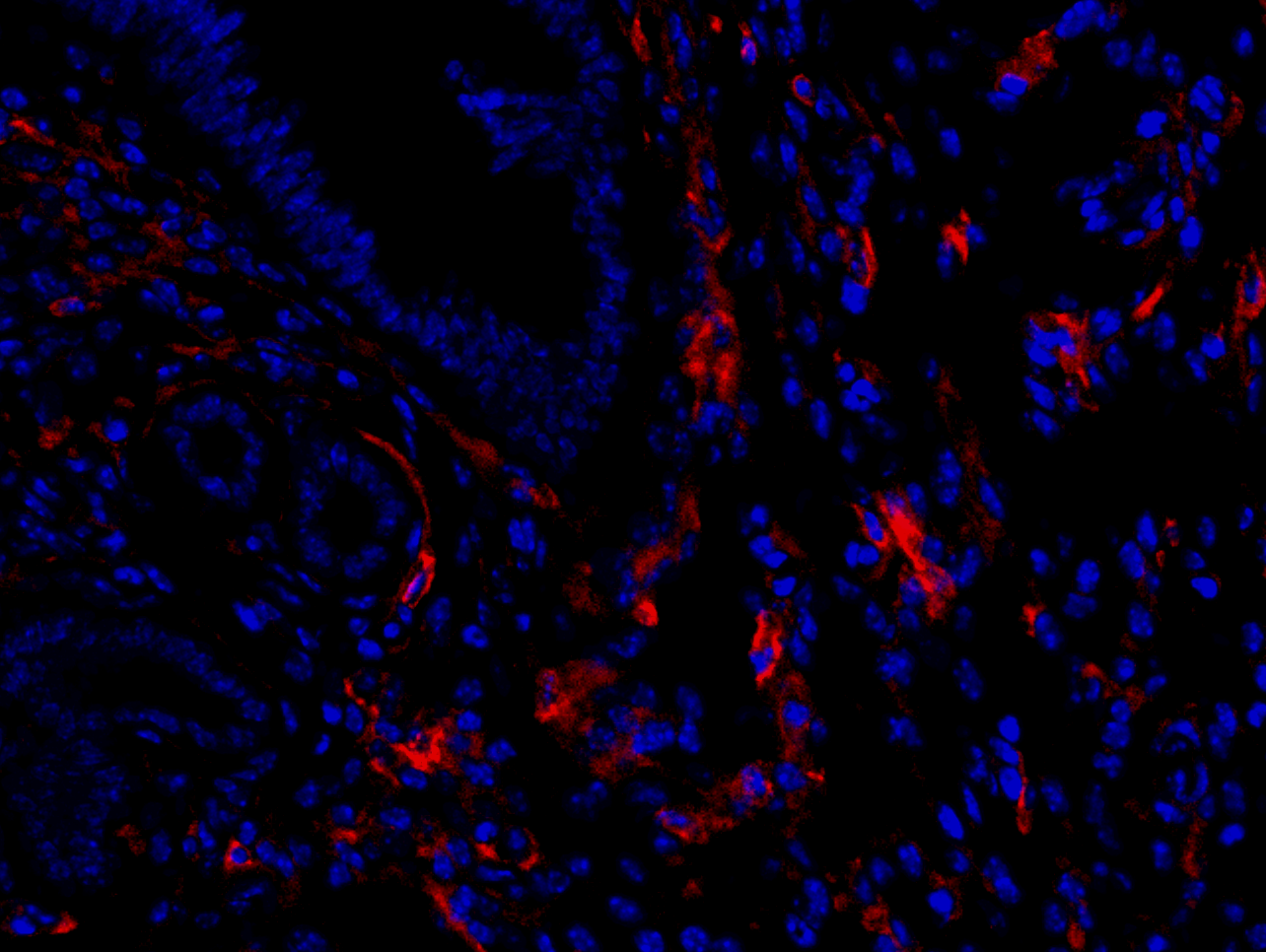

Supplement: Supplementary file 8 — Source Data for Figure 7 [file EMMM-15-e17601-s001.zip › Figure 7-1/7C/AXL IUA+PBS.tif]

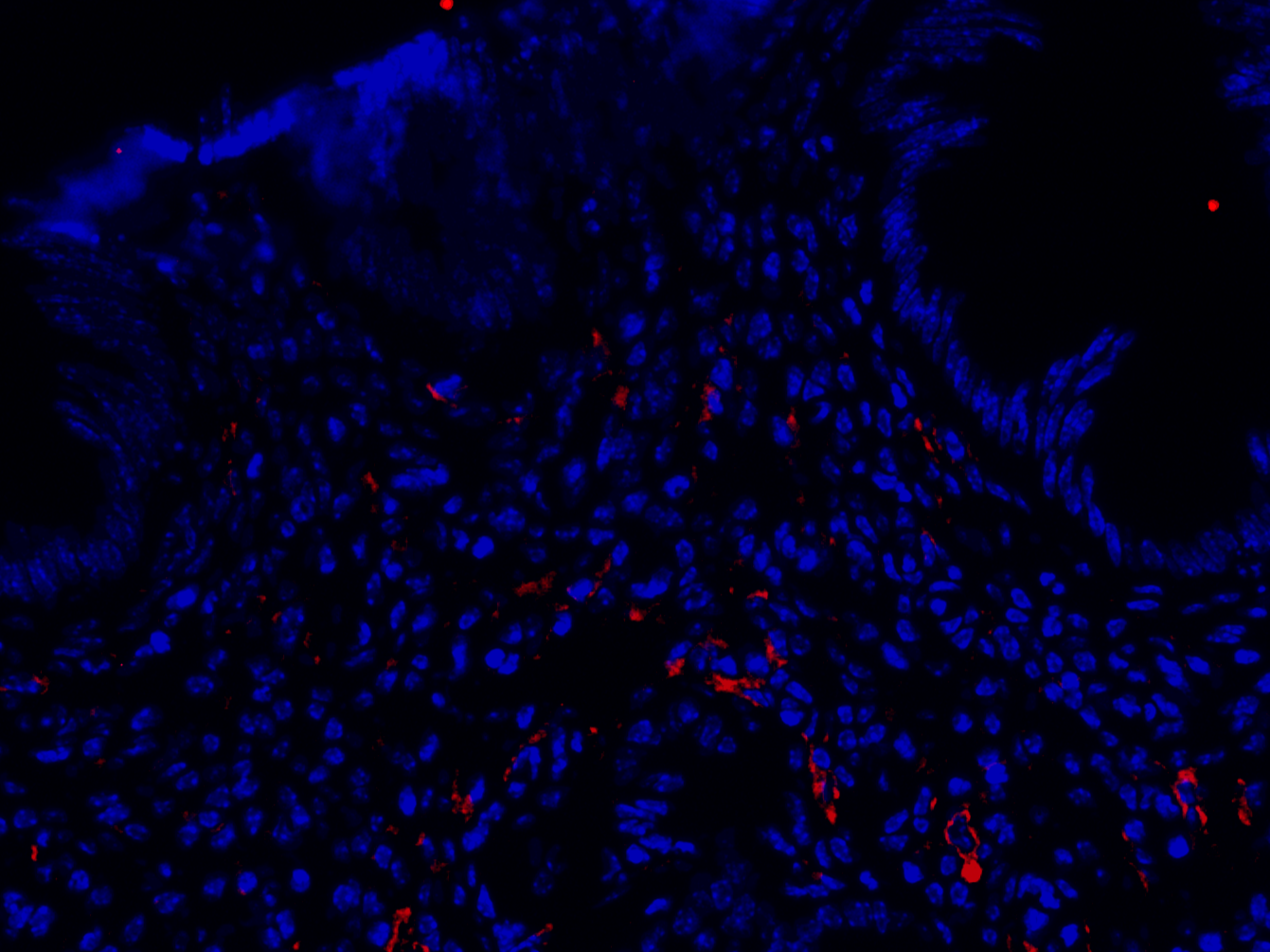

Supplement: Supplementary file 8 — Source Data for Figure 7 [file EMMM-15-e17601-s001.zip › Figure 7-1/7C/AXL IUA+BEM.tif]
