# Supplementary material for: Targeting CD301 + macrophages inhibits endometrial fibrosis and improves pregnancy outcome
Source: EMBO Mol Med. 2023 Jul 31;15(9):e17601. doi: 10.15252/emmm.202317601 (PMC10493587; doi:10.15252/emmm.202317601)
Supplement: Supplementary file 9 [file EMMM-15-e17601-s002.pdf]

EMBO Molecular Medicine

Targeting CD301+ macrophages inhibits endometrial fibrosis and improves pregnancy outcome

Haining Lv, Haixiang Sun, Limin Wang, Simin Yao, Dan Liu, Xiwen Zhang, Zhongrui Pei, Jianjun Zhou, Huiyan Wang, Jianwu Dai, Guijun Yan, Lijun Ding, Zhiyin Wang, Chenrui Cao, Guangfeng Zhao, and Yali Hu
DOI: 10.15252/emmm.202317601

Corresponding authors: Yali Hu (yalihu@nju.edu.cn) , Guangfeng Zhao (zhaoguangfeng@nju.edu.cn)

Review Timeline:

Submission Date:	20th Feb 23
Editorial Decision:	30th Mar 23
Revision Received:	2nd Jun 23
Editorial Decision:	6th Jul 23
Revision Received:	13th Jul 23
Accepted:	18th Jul 23

Editor: Zeljko Durdevic

Transaction Report:

(Note: With the exception of the correction of typographical or spelling errors that could be a source of ambiguity, letters and reports are not edited. Depending on transfer agreements, referee reports obtained elsewhere may or may not be included in this compilation. Referee reports are anonymous unless the Referee chooses to sign their reports.)

30th Mar 2023

Dear Dr. Hu,

Thank you for the submission of your manuscript to EMBO Molecular Medicine. We have now received feedback from the three reviewers who agreed to evaluate your manuscript. As you will see from the reports below, all referees recognize potential interest of the study, but also raise important concerns that should be addressed in a major revision. If you would like to discuss further the points raised by the referees, I am available to do so via email or video. Let me know if you are interested in this option.

Further consideration of a revision that addresses reviewers' concerns in full will entail a second round of review. EMBO Molecular Medicine encourages a single round of revision only and therefore, acceptance or rejection of the manuscript will depend on the completeness of your responses included in the next, final version of the manuscript. For this reason, and to save you from any frustrations in the end, I would strongly advise against returning an incomplete revision.

We would welcome the submission of a revised version within three months for further consideration. Please let us know if you require longer to complete the revision.

I look forward to receiving your revised manuscript.

Yours sincerely,

Zeljko Durdevic

Zeljko Durdevic
Editor
EMBO Molecular Medicine

*****

When submitting your revised manuscript, please carefully review the instructions that follow below. We perform an initial quality control of all revised manuscripts before re-review; failure to include requested items will delay the evaluation of your revision.

We require:

1) A .docx formatted version of the manuscript text (including legends for main figures, EV figures and tables). Please make sure that the changes are highlighted to be clearly visible.

2) Individual production quality figure files as .eps, .tif, .jpg (one file per figure). For guidance, download the 'Figure Guide PDF': (https://www.embopress.org/page/journal/17574684/authorguide#figureformat).

3) A .docx formatted letter INCLUDING the reviewers' reports and your detailed point-by-point responses to their comments. As part of the EMBO Press transparent editorial process, the point-by-point response is part of the Review Process File (RPF), which will be published alongside your paper.

4) A complete author checklist, which you can download from our author guidelines (https://www.embopress.org/page/journal/17574684/authorguide#submissionofrevisions). Please insert information in the checklist that is also reflected in the manuscript. The completed author checklist will also be part of the RPF.

5) Please note that all corresponding authors are required to supply an ORCID ID for their name upon submission of a revised manuscript.

6) It is mandatory to include a 'Data Availability' section after the Materials and Methods. Before submitting your revision, primary

datasets produced in this study need to be deposited in an appropriate public database, and the accession numbers and database listed under 'Data Availability'. Please remember to provide a reviewer password if the datasets are not yet public (see https://www.embopress.org/page/journal/17574684/authorguide#dataavailability).

In case you have no data that requires deposition in a public database, please state so in this section. Note that the Data Availability Section is restricted to new primary data that are part of this study.

7) For data quantification: please specify the name of the statistical test used to generate error bars and P values, the number (n) of independent experiments (specify technical or biological replicates) underlying each data point and the test used to calculate p-values in each figure legend. The figure legends should contain a basic description of n, P and the test applied. Graphs must include a description of the bars and the error bars (s.d., s.e.m.). See also 'Figure Legend' guidelines: https://www.embopress.org/page/journal/17574684/authorguide#figureformat

8) At EMBO Press we ask authors to provide source data for the main manuscript figures. Our source data coordinator will contact you to discuss which figure panels we would need source data for and will also provide you with helpful tips on how to upload and organize the files.

9) Our journal encourages inclusion of *data citations in the reference list* to directly cite datasets that were re-used and obtained from public databases. Data citations in the article text are distinct from normal bibliographical citations and should directly link to the database records from which the data can be accessed. In the main text, data citations are formatted as follows: "Data ref: Smith et al, 2001" or "Data ref: NCBI Sequence Read Archive PRJNA342805, 2017". In the Reference list, data citations must be labeled with "[DATASET]". A data reference must provide the database name, accession number/identifiers and a resolvable link to the landing page from which the data can be accessed at the end of the reference. Further instructions are available at .

10) We replaced Supplementary Information with Expanded View (EV) Figures and Tables that are collapsible/expandable online. A maximum of 5 EV Figures can be typeset. EV Figures should be cited as 'Figure EV1, Figure EV2" etc... in the text and their respective legends should be included in the main text after the legends of regular figures.

- For the figures that you do NOT wish to display as Expanded View figures, they should be bundled together with their legends in a single PDF file called *Appendix*, which should start with a short Table of Content. Appendix figures should be referred to in the main text as: "Appendix Figure S1, Appendix Figure S2" etc.

- Additional Tables/Datasets should be labeled and referred to as Table EV1, Dataset EV1, etc. Legends have to be provided in a separate tab in case of .xls files. Alternatively, the legend can be supplied as a separate text file (README) and zipped together with the Table/Dataset file.

See detailed instructions here:

.

11) The paper explained: EMBO Molecular Medicine articles are accompanied by a summary of the articles to emphasize the major findings in the paper and their medical implications for the non-specialist reader. Please provide a draft summary of your article highlighting

- the medical issue you are addressing,

- the results obtained and

- their clinical impact.

This may be edited to ensure that readers understand the significance and context of the research. Please refer to any of our published articles for an example.

12) For more information: There is space at the end of each article to list relevant web links for further consultation by our readers. Could you identify some relevant ones and provide such information as well? Some examples are patient associations, relevant databases, OMIM/proteins/genes links, author's websites, etc...

13) Author contributions: You will be asked to provide CRediT (Contributor Role Taxonomy) terms in the submission system. These replace a narrative author contribution section in the manuscript.

14) A Conflict of Interest statement should be provided in the main text.

15) Every published paper now includes a 'Synopsis' to further enhance discoverability. Synopses are displayed on the journal webpage and are freely accessible to all readers. They include a short stand first (maximum of 300 characters, including space) as well as 2-5 one-sentences bullet points that summarizes the paper. Please write the bullet points to summarize the key NEW findings. They should be designed to be complementary to the abstract - i.e. not repeat the same text. We encourage inclusion of key acronyms and quantitative information (maximum of 30 words / bullet point). Please use the passive voice. Please attach these in a separate file or send them by email, we will incorporate them accordingly.

Please also suggest a striking image or visual abstract to illustrate your article as a PNG file 550 px wide x 300-600 px high.

EMBO Molecular Medicine has a "scooping protection" policy, whereby similar findings that are published by others during review or revision are not a criterion for rejection. Should you decide to submit a revised version, I do ask that you get in touch after three months if you have not completed it, to update us on the status.

Please note: When submitting your revision you will be prompted to enter your funding and payment information. This will allow Wiley to send you a quote for the article processing charge (APC) in case of acceptance. This quote takes into account any reduction or fee waivers that you may be eligible for. Authors do not need to pay any fees before their manuscript is accepted and transferred to the publisher.

EMBO Press participates in many Publish and Read agreements that allow authors to publish Open Access with reduced/no publication charges. Check your eligibility: https://authorservices.wiley.com/author-resources/Journal-Authors/open-access/affiliation-policies-payments/index.html

***** Reviewer's comments *****

Referee #1 (Comments on Novelty/Model System for Author):

No issues re ethics of using the models used in this study.

Referee #1 (Remarks for Author):

This study addresses a very challenging clinical condition - intrauterine adhesions (IUA) - which contribute to uterine-factor infertility and increased risk of poor pregnancy outcomes in women. IUAs generally derive after uterine surgery including dilatation & curettage (D&C) (especially postpartum), or post-infection (TB, endometritis), and can obliterate the entire uterine endometrium or portions thereof. Focus of the study is on a subpopulation of endometrial macrophages, CD301+, discovered by scRNAseq of endometrium obtained under direct visualization at hysteroscopy in the late proliferative phase in 3 patients with IUA (cases) and 3 controls. CellPhone DB shows interactions between CD301+ cells with endometrial stromal fibroblasts (eSF), and pseudo-time trajectory analysis shows that myofibroblasts derive from the eSF. Bulk RNAseq revealed GAS6 and AXL/NF b signaling pathways involved in the setting of IUA, and FACS isolation and magnetic bead isolation confirmed an increase in CD301+ cells in IUA cases and that the AS6AXL/NF B axis promotes the fibrotic eSF phenotype (biomarkers) in vitro. Moreover, targeted depletion of CD301+ equivalent macrophages in a mouse model of IUA supports a profibrotic role for these cells in IUA, and Bemcentinib, an inhibitor of AXL signaling, inhibited IUA progression a few days after induction of fibrosis in the mouse model and improved pregnancy/live pup rates. A few items for the authors to consider:

1. In other scRNAseq analyses, there are several eSF phenotypes identified. Did the authors find such in the cases and controls, understanding that the scRNAseq was limited to 3 samples in each group?
2. 2. How did the author rule out infectious causes of IUA among the patient samples used in the various studies described in this manuscript, and please comment on absence of uterine malformations (which are also associated with IUA)?
3. While the clinical metadata assure uniformity of cycle phase, key in transcriptomic analyses of human endometrium, could the authors please comment on duration of IUA in patients and whether such had any unique phenotypes in the CD301+ cells or other cell types, as IUA can be progressive.
4. The authors identify endometrial epithelial cells, but more information is warranted about them in cases vs. controls, as they play a major role in re-epithelialization of endometrium after trauma or scarring. Also, as epithelia and stroma interactions between each other and with macrophages occur in the endometrium, did the authors find anything in their CellPhone DB analyses of such?
5. As adhesion formation in IUA can result from aberrant endometrial repair after injury, an analysis of interactions among cell types more comprehensively (e.g., endothelial cells, progenitors) is warranted.
6. Were epithelial (or eSF or endothelial) progenitors identified in cases and controls - particularly relevant to potential for them as targets for treating IUA/scarred endometrium.
7. The timing of Bemventinib warrants more clarification - why did the authors not administer it at the time of IUA induction for analysis of prevention of adhesions rather than several days later?
8. In the mouse model, did Bemcentinib have any off-target effects, as these could limit this drug class for further use in reproductive age women and fetuses.
9. With inhibition of AXL signaling as a potential clinical approach to resolving or preventing IUA in patients with existing IUA or

those at high risk for this condition, a bit more discussion on current clinical indications and side effects of Bemcentinib or drugs in this class is warranted.
10. It would be of value for the authors to mention auto-crosslinked hyaluronic acid after D&C, e.g., as one of the few available treatments to date to reduce incidence and severity of IUA in high-risk patients (see SantaMaria et al 2018).
11. In the Discussion, line 268, endometrial scarring is not the leading cause of uterine-factor infertility. Rather there are abundant data for intrauterine inflammation and congenital anomalies. I suggest deleting that phrase.
12. Table S6, what does "time for D&C "refer to? And what are the units - years? months?

Referee #2 (Remarks for Author):

Lv et al., conducted single cell RNA-seq analysis on IUA patients to predict CD301+ macrophages interact with stromal fibroblasts to support fibrosis in the disease. The authors follow up this analysis with in vitro studies and mouse models that target CD301b+ macrophages to implicate GAS6/AXL signaling as mechanism of cellular communication. The manuscript is well written and of interest to the journal's audience.

In general, there are many statements in the figure titles are not substantiated by the data presented in the figures and should be changed. A few of these specific instances are pointed out below. Additionally, many of the findings from in vitro stimulation of hESCs should be distinguished from in vivo experiments in the titles. The authors frequently state "of IUA" for the figure titles when most of the mechanistic support is from stimulated hESCs in vitro.

Below are some of the more significant concerns and questions regarding the data presented in the manuscript.

Figure 1:

1) SMA staining suggests there are more myofibroblasts or myofibroblast-like cells in IUA patients, the quantification in D is not significant and the relative difference in myofiboblast-like cells could be due to differences in immune cell numbers. Additionally, the subcluster analysis from H indicates that the samples obtained do not have significant differences in populations of myofibroblast-like cells. This is surprising since it is likely that one population (or a few clusters) would be enriched with fibrogenic potential and CD301+ macrophages have been associated with increased numbers of myofibroblasts with a unique gene expression profile and specific identity markers (Shook et al., 2018). This could likely be due to incomplete digestion of the highly fibrotic tissue section, which would significantly impact the cells being captured for downstream analysis. Quantification or subtype identification should be confirmed in tissue sections. This can also be observed in the lack of significantly increased CD301+ macrophage numbers in Figure 1M with a marked increase in CD301+ macrophages quantified in Figure 2A and 2B.

2) N = 6 is misleading since n =3 for each group. For Figure 1 K and L, is the predicted signaling enriched in the UIA patients? If predicted CD301+ macrophage signaling with myofibroblast-like cells is in the control and IUA patients how do the authors speculate that this contributes to fibrosis in patients? The authors should show what signaling is enriched in the disease patients based on gene expression that is upregulated in disease Cd301+ macrophages relative to controls.

3) CellPhoneDB is a great starting point for cellular communication, but there are more advanced tools to predict cellular communication, such as CellChat. The data analysis and key findings presented in this figure is quite basic and superficial. The authors should elaborate more on the findings, especially as they relate to macrophage-fibroblast interactions.

4) The title is not substantiated by the data presented in the figure.

5) Macrophage gene expression is highly context and timing dependent. It might be surprising to some that CD301+ macrophages to have greater enrichment for many pro-inflammatory genes. The number of macrophages should be sufficient to validate the RNA-seq predicted changes in gene expression with qPCR or western blot analysis to confirm this hybrid pro-inflammatory, pro-fibrotic transcriptomic state that could contribute to the pathology. Additionally, one would expect this transcriptional state to be specific to the CD301+ macrophages in IUA patients, but this analysis was not shown.

Figure 2:

1) Are there more CD301+ macrophages near SMA dense areas? CD301+ macrophages are frequently observed throughout many tissues, so their presence near SMA regions is not surprising. It would be noteworthy if their numbers were increased in fibrotic regions but SMA- regions are not shown and no quantification was performed to substantiate the claim.

2) The title is not substantiated by the data presented in the figure. To determine a profibrotic phenotype, more than just collagen I should be examined. Especially considering the change in SMA is modest (though statistically significant).

Figure 3:

1) The in vitro GAS6/AXL findings are quite exciting; however, the majority of GAS6/AXL signaling in vivo may be between immune cells. While CD10 allows pathologists to distinguish the stromal and epithelial fraction of endometrial neoplasms, it is not used to determine fibroblast-like cells compared to immune cells in the stromal fraction of tumors. Why didn't the authors perform SMA and AXL staining? At the very least, it should be demonstrated that the CD10 staining is largely restricted to non-immune cells (CD45-).

2) There are concerns regarding the AXL antibody used and its suitability for immunostaining cells and tissue sections. The majority of AXL staining does not colocalize with SMA+ cells in Figure S4G. In fact, most of the staining does not colocalize with any DAPI+ nuclei.

Figure 5:

1) The curettage model is an acute injury model that the authors do not show has adhesions. Are adhesions observed in tile scanned or more zoomed out fields of view from this injury model? To confirm that the curettage model is similar to the human IUA model and that this time point is appropriate a more thorough analysis of the immune cell composition is needed in Figure 5D, especially given the dramatic reduction in the relative abundance of macrophages compared to other CD45+ immune cells. More detailed characterization should be compared to immune cell composition in Figure 1.

Referee #3 (Remarks for Author):

This manuscript by Lv et al provides new insights into the role of CD310+ macrophages in regulating endometrial fibrosis. Targeting CD301+ macrophages revealed that fibrotic phenotype could be modulated leading to improved pregnancy outcome. The studies are well designed by incorporating single cell RNA seq RNA approach, flow cytometry and immunohistology on IUA patient and control samples. Mechanistic studies were conducted in primary endometrial stromal cells. Finally, they conducted interventional studies by targeting CD301+ macrophages in C57Bl6 mouse model. In general, the manuscript is well written. Experiments are well designed with proper controls. Results support the conclusion. This manuscript provides new insights into the complexity of immune associated fibrosis and its overall impact on pregnancy outcome. Some clarifications are required as below.

• They should provide clarity on proliferating macrophage subtype as mentioned in the single cell RNA seq data in Fig 1 J. Does that mean the other subtypes did not proliferate? This is relevant as macrophage proliferation is low at steady state.
• Clarify the significance of lower numbers of CD163+ and CD24+ macrophages (Fig 1M) in IUA patients?
• They have very clearly articulated sample numbers for each experiment as well as patient demographic in Supplemental table 1. They should clarify parity information if available for the patients included in the study.
• Some caution should be exercised in linking pathogenic role of CD301+ macrophages as outcome of single cell RNA seq is observational.
• In Fig 1F, how did they define proliferating cell subset and was it unique within the cluster?
• What is the significance of lower number of CD14+ cells in IUA patients?
• Representative images in Fig 2B (colocalization of CD68 with CD301) does not really capture significantly different numbers of CD301 as shown in other figures.
• Fig 3B: Clarify the rational for selecting those specific genes for Q-RT PCR and what do they mean by genes were upregulated.

Point-by-point response to referees' comments:

We deeply appreciate the referees for their highly insightful and constructive comments and suggestions!

Referee #1 (Comments on Novelty/Model System for Author):

No issues re ethics of using the models used in this study.

Referee #1 (Remarks for Author):

This study addresses a very challenging clinical condition - intrauterine adhesions (IUA) - which contribute to uterine-factor infertility and increased risk of poor pregnancy outcomes in women. IUAs generally derive after uterine surgery including dilatation & curettage (D&C) (especially postpartum), or post-infection (TB, endometritis), and can obliterate the entire uterine endometrium or portions thereof. Focus of the study is on a subpopulation of endometrial macrophages, CD301+, discovered by scRNAseq of endometrium obtained under direct visualization at hysteroscopy in the late proliferative phase in 3 patients with IUA (cases) and 3 controls. CellPhone DB shows interactions between CD301+ cells with endometrial stromal fibroblasts (eSF), and pseudo-time trajectory analysis shows that myofibroblasts derive from the eSF. Bulk RNAseq revealed GAS6 and AXL/NFκb signaling pathways involved in the setting of IUA, and FACS isolation and magnetic bead isolation confirmed an increase in CD301+ cells in IUA cases and that the GAS6AXL/NFκB axis promotes the fibrotic eSF phenotype (biomarkers) in vitro. Moreover, targeted depletion of CD301+ equivalent macrophages in a mouse model of IUA supports a profibrotic role for these cells in IUA, and Bemcentinib, an inhibitor of AXL signaling, inhibited IUA progression a few days after induction of fibrosis in the mouse model and improved pregnancy/live pup rates. A few items for the authors to consider:

1. In other scRNAseq analyses, there are several eSF phenotypes identified. Did the authors find such in the cases and controls, understanding that the scRNAseq was limited to 3 samples in each group?
Response to point 1: Thanks for the comment. Yes, we found such several eSF (stromal cell) phenotypes in our scRNAseq analyses. Proliferating cells, normal cell, and progenitor-like cells were identified referring to the published scRNAseq analyses (PMID: 35169075, 36185612, 33710643). We also identified PAI1high cells and pro-inflammatory cells that had an increased tendency in IUA patients which has not been reported previously. We supplemented the analysis of eSF (stromal cells) in Appendix Fig S2A-C.

2. How did the author rule out infectious causes of IUA among the patient samples used in the various studies described in this manuscript, and please comment on absence of uterine malformations (which are also associated with IUA)?
Response to point 2: Thanks for pointing these out. We have ruled out infectious and uterine-malformational causes of IUA by the following exclusion criteria. Women were excluded from the tissue collection if they were under any of the following condition: positive serological tests for

human immunodeficiency virus, hepatitis B/C virus, syphilis; history of tuberculosis; chronic endometritis; vaginal bacterial or fungal. Patients with uterine malformations determined by ultrasonography have been ruled out before sample collection. We added the above description in Human samples section of Method in the revised manuscript.

3. While the clinical metadata assure uniformity of cycle phase, key in transcriptomic analyses of human endometrium, could the authors please comment on duration of IUA in patients and whether such had any unique phenotypes in the CD301+ cells or other cell types, as IUA can be progressive.
Response to point 3: We appreciate for your question. As the reviewer indicated, IUA can be progressive and has a non-negligible variation between individuals, since some people suffered from severe IUA even when they experienced only one time of previous curettage, but others may just begin to have IUA after several times of curettage. Therefore, in order to acquire the credible and representative data, we have not only ensured the uniformity of cycle phase to be late proliferative phase, but also strictly selected the samples of patients who were diagnosed IUA with scores 9-12 based on criteria recommended by the American Fertility Society (PMID: 3371491), shown in Appendix Table S1 and S4. Therefore, all the samples were in the relatively similar grade of disease at same cycle phase which had unique phenotypes in the CD301+ cells and other cell types.

4. The authors identify endometrial epithelial cells, but more information is warranted about them in cases vs. controls, as they play a major role in re-epithelialization of endometrium after trauma or scarring. Also, as epithelia and stroma interactions between each other and with macrophages occur in the endometrium, did the authors find anything in their CellPhone DB analyses of such?
Response to point 4: We thank the reviewer for the insightful comment. More information has been supplemented in Appendix Fig S2. We identified the subpopulation of epithelial cells and performed CellPhoneDB among epithelial cells, stromal cells, and macrophages shown in Appendix Fig S2.

As shown in the figure, pro-inflammatory stromal cells had more communication with epithelial cells, especially progenitor-like epithelial cells in IUA patients, indicated abnormal response to inflammation may exist and affect the progenitor's function in epithelia (Appendix Fig S2J).

CD163+ macrophage played an important role in endometrial angiogenic remodeling and tissue regeneration (PMID: 22882270) and epithelial progenitor-like cell and glandular cell were reported to participate in re-epithelization of endometrium at proliferative phase (PMID: 34486650). Our results showed that CD163+ macrophage predominantly interacted with progenitor-like cell and glandular cell in epithelia (Appendix Fig S2K) while the number of CD163+ macrophage was significantly reduced in IUA patients (Fig 1M and 2A). This result indicated that the epithelial progenitor-like cell and glandular cell could not receive the beneficial signaling sufficiently to function during the re-epithelization of endometrium at proliferative phase in IUA patients.

Moreover, CD301+ macrophage has more communication with pro-inflammatory stromal cells which were significantly increased in IUA patients (Appendix Fig S2C and K), suggesting that CD301+ macrophage participated more in stroma inflammation and the inflammation was higher in IUA patients. The aberrant inflammation in both epithelia and stroma were consistent with the previous document that adhesiogenesis represents the culmination process of an abnormal response to inflammation, to damage the endometrial niches and stimulate the formation of fibrotic tissue and decrease vascularization (PMID: 31986212; PMID: 30503114).

5. As adhesion formation in IUA can result from aberrant endometrial repair after injury, an analysis of interactions among cell types more comprehensively (e.g., endothelial cells, progenitors) is warranted.

Response to point 5: Thanks for pointing these out. We have added analysis of interactions among epithelial cells, stromal cells, endothelial cells including their progenitor-like cells in Appendix Fig S2.

6. Were epithelial (or eSF or endothelial) progenitors identified in cases and controls - particularly relevant to potential for them as targets for treating IUA/scarred endometrium.

Response to point 6: Yes. As suggested by the reviewer, we identified progenitor-like cells in epithelial, eSF and endothelial cells (Appendix Fig S2 A, D, G). The markers for each type of progenitor-like cells were shown in Appendix Fig S2 B, E, H. There were no obvious changes of cell number in progenitor-like cells between cases and controls (Appendix Fig S2 C, F, I), but progenitor-like endothelial cells interacted more with other progenitors in cases. As we mainly focused on the interaction between macrophage and myofibroblast-like cell in this study, the effect of progenitors in IUA/scarred endometrium and the relevant potential targets for the treatment warrant future investigation.

7. The timing of Bemcentinib warrants more clarification - why did the authors not administer it at the time of IUA induction for analysis of prevention of adhesions rather than several days later?

Response to point 7: Thanks for the comment. Initially, we administered Bemcentinib after IUA model establishment to simulate the common situation of clinic: we usually perform some treatments during hysteroscopic adhesiolysis which the adhesion has happened, to prevent re-adhesion, inhibit fibrosis, and improve the endometrial micro-environment to promote its regeneration clinically. Thus, the treatment is administered after adhesion. On the other hand, it is worth seeing whether the drug has the effect on prevention of first adhesion, so we added the experiments to administer Bemcentinib begun at the time of IUA induction shown in Appendix Fig S8. Consistently, Bemcentinib could effectively prevent endometrial fibrosis and improve the pregnancy outcome.

8. In the mouse model, did Bemcentinib have any off-target effects, as these could limit this drug class for further use in reproductive age women and fetuses.

Response to point 8: This is an insightful question and highly related to the specificity of Bemcentinib. *In vitro* study, Bemcentinib has minimal off-target antiproliferative or cytotoxic activity in two-dimensional assays in several cell types and is unique in its nanomolar on-target activity and restricted selectivity profile (PMID: 20145120). Another *in vitro* study pointed that Bemcentinib at 1 and 2.5 uM inhibited AXL without off-targets (PMID: 34429509). The off-target effect of Bemcentinib *in vivo* has not been documented. In our study, we found that Bemcentinib administration can increase live pups and decrease absorbed embryos, at least in part, demonstrating that it may have minimal toxicity for reproduction at this dose. In future studies one may be able to demonstrate and minimize off-target effects of Bemcentinib *in vivo* by dose optimization.

9. With inhibition of AXL signaling as a potential clinical approach to resolving or preventing IUA in patients with existing IUA or those at high risk for this condition, a bit more discussion on current clinical indications and side effects of Bemcentinib or drugs in this class is warranted.

Response to point 9: Thanks for pointing these out. In clinical trials, Bemcentinib exerts effective anti-leukaemic activity in acute myeloid leukemia patients and the common side effects are ECG QT prolongation and diarrhea which are manageable and/or reversible (Sonja, et al.,2019). It is also feasible and tolerable to treat non-small alveolar lung cancer patients by abrogating resistance to EGFR inhibitors confirmed in PhI/II study with the mainly side effects of diarrhea and nausea (Lauren, et al., 2021). There are some other clinical trials of Bemcentinib registered on ClinicalTrials.gov in progress of which results have not been reported. The relevant description has been added in Discussion in the revised manuscript.

10. It would be of value for the authors to mention auto-crosslinked hyaluronic acid after D&C, e.g., as one of the few available treatments to date to reduce incidence and severity of IUA in high-risk patients (see SantaMaria et al 2018).
Response to point 10: Thanks for the suggestion. We agreed that application of auto-crosslinked hyaluronic acid after D&C could reduce the incidence and severity of IUA in high-risk patients with at least a previous D&C, which acts through different mechanisms of action that provide physical support and mechanical protection, but also regulate the inflammatory response and promote vascular regeneration and wound healing, modulating inflammation in the adhesiogenesis process. We added it in Discussion in the revised manuscript.

11. In the Discussion, line 268, endometrial scarring is not the leading cause of uterine-factor infertility. Rather there are abundant data for intrauterine inflammation and congenital anomalies. I suggest deleting that phrase.
Response to point 11: Thanks for reminding. We have deleted the phrase in the revised manuscript.

12. Table S6, what does "time for D&C "refer to? And what are the units - years? months?
Response to point 12: Time represents number. Since some patients may undergo D&C more than once, we recorded the times for the reference of treatment.

Referee #2 (Remarks for Author):

Lv et al., conducted single cell RNA-seq analysis on IUA patients to predict CD301+ macrophages interact with stromal fibroblasts to support fibrosis in the disease. The authors follow up this analysis with in vitro studies and mouse models that target CD301b+ macrophages to implicate GAS6/AXL signaling as mechanism of cellular communication. The manuscript is well written and of interest to the journal's audience.

In general, there are many statements in the figure titles are not substantiated by the data presented in the figures and should be changed. A few of these specific instances are pointed out below.

Additionally, many of the findings from in vitro stimulation of hESCs should be distinguished from in vivo experiments in the titles. The authors frequently state "of IUA" for the figure titles when most of the mechanistic support is from stimulated hESCs in vitro.
Response: We apologize much for the inaccurate titles. We have thoroughly revised the figure titles to ensure that it is accurate to summarize figures.

Below are some of the more significant concerns and questions regarding the data presented in the manuscript.

Figure 1:

1) SMA staining suggests there are more myofibroblasts or myofibroblast-like cells in IUA patients, the quantification in D is not significant and the relative difference in myofiboblast-like cells could be due to differences in immune cell numbers. Additionally, the subcluster analysis from H indicates that the samples obtained do not have significant differences in populations of myofibroblast-like cells. This is surprising since it is likely that one population (or a few clusters) would be enriched with fibrogenic potential and CD301+ macrophages have been associated with increased numbers of myofibroblasts with a unique gene expression profile and specific identity markers (Shook et al., 2018). This could likely be due to incomplete digestion of the highly fibrotic tissue section, which would significantly impact the cells being captured for downstream analysis. Quantification or subtype identification should be confirmed in tissue sections. This can also be observed in the lack of significantly increased CD301+ macrophage numbers in Figure 1M with a marked increase in CD301+ macrophages quantified in Figure 2A and 2B.
Response to point 1: Thanks for the comment. We got the reasonable tendency of changes in myofibroblast-like cells and its subclusters, and macrophages by scRNA-seq. To get robust validation of phenotypic changes analyzed by the sequencing, we performed immunostaining and flow cytometry using larger number of samples, *in vitro* cell culture and *in vivo* animal models in the following experiment. As suggested by the reviewer, the quantification of Fig 1F were added and the subtype identification were shown in Fig 1F (for ACTA2^{high} cell, COL1A1^{high} cell), Fig 2A-B (for CD301+ macrophage, CD163+ macrophage), Appendix Fig S1E (for adventitial cell, pericyte, CD163+ macrophage and CD24+ macrophage).

2) N = 6 is misleading since n =3 for each group. For Fig 1 K and L, is the predicted signaling enriched in the IUA patients? If predicted CD301+ macrophage signaling with myofibroblast-like cells is in the control and IUA patients how do the authors speculate that this contributes to fibrosis in patients? The authors should show what signaling is enriched in the disease patients based on gene expression that is upregulated in disease CD301+ macrophages relative to controls.
Response to point 2: We appreciate the reviewer's concern very much. The result of CellPhoneDB in Fig 1K and L was integrated controls' and IUA patients' data. No significant change of CD301+ macrophage was found in CellPhoneDB analysis between control and IUA patients, so we did not put the separate figures on. But noteworthily, the number of CD301+ macrophage was increased in the endometrium of IUA patients, shown in Fig 1M, Fig 2A-C, so CD301+ macrophage intrinsically exists in normal endometrium but supra-physiological over-increase do harm to endometrial

regeneration. At this point, we focused more on the specific phenotype of CD301+ macrophage compared to CD301- macrophage, referring to Shook et al, 2018 (PMID: 30467144).

As suggested by the reviewer, we have added CellChat analysis between CD301+ macrophage and myofibroblast-like cells to mine the signaling that was enriched in CD301+ macrophage based on upregulated gene expression in IUA patients shown in Appendix Fig S3E-H. Additionally, the signaling of upregulated genes which was enriched in CD301+ macrophage of the disease patients compared to controls by KEGG analysis were supplemented in Appendix Fig S3D.

3) CellPhoneDB is a great starting point for cellular communication, but there are more advanced tools to predict cellular communication, such as CellChat. The data analysis and key findings presented in this figure is quite basic and superficial. The authors should elaborate more on the findings, especially as they relate to macrophage-fibroblast interactions.

Response to point 3: Thanks for the valuable suggestion. We elaborated more on the findings, using both CellPhoneDB and CellChat, shown in Appendix Fig S2 and S3. We subclustered and identified stromal cells (fibroblasts) and elaborated cell-to-cell communication between stromal cells and macrophages, shown in Appendix Fig S2A-C, K. As shown in the figure, CD301+ macrophage had more communication with pro-inflammatory stromal cells which were significantly increased in IUA patients (Appendix Fig S2C and K), suggesting that CD301+ macrophage participated more in stroma inflammation and the inflammation was higher in IUA patients. This result was consistent with the previous document that adhesiogenesis represents the culmination process of an abnormal response to inflammation, to damage the endometrial niches and stimulate the formation of fibrotic tissue and decrease vascularization (PMID: 31986212; PMID: 30503114), thus increased number of CD301+ macrophage and more interaction with pro-inflammatory stromal cells in IUA patient made CD301+ macrophage liable to accelerate endometrial adhesion and fibrosis. We have added CellChat analysis between CD301+ macrophage and myofibroblast-like cells in Appendix Fig S3E-H. We found that CD301+ macrophage was more active on TWEAK signaling pathway which specifically targeted on pro-fibrotic subclusters, ACTA2^{high} cell and COL1A1^{high} cell, and IGF signaling pathway which specifically targeted on fibrocyte, especially in IUA group. Both TWEAK and IGF signaling pathway are recognized to positively regulate cell proliferation and growth (PMID: 35169075, PMID: 35938159), suggesting that CD301+ macrophage promoted the increase of pro-fibrotic myofibroblast-like cells, especially in IUA group. These findings were consistent with our following experimental validation. The relevant description has been supplemented in Result in the revised manuscript.

4) The title is not substantiated by the data presented in the figure.

Response to point 4: Thanks for the critique. We changed the title into 'scRNA-seq cartography of myofibroblast-like cell and macrophage in human endometrium between IUA patients and healthy controls'.

5) Macrophage gene expression is highly context and timing dependent. It might be surprising to some that CD301+ macrophages to have greater enrichment for many pro-inflammatory genes. The number of macrophages should be sufficient to validate the RNA-seq predicted changes in gene expression with qPCR or western blot analysis to confirm this hybrid pro-inflammatory, pro-fibrotic transcriptomic state that could contribute to the pathology. Additionally, one would expect this

transcriptional state to be specific to the CD301+ macrophages in IUA patients, but this analysis was not shown.

Response to point 5: We appreciate the insightful thoughts of the reviewer. CD301+ macrophages indeed have greater enrichment for many pro-inflammatory genes and have more communication with pro-inflammatory stromal cells with the increased number of pro-inflammatory stromal cells in IUA patients shown in Appendix Fig S2C and K. Following the reviewer's suggestion, we supplemented the upregulated genes in CD301+ macrophages to be specific to IUA patients compared to controls as shown in Appendix Fig S3B. The gene expression difference between CD301+ macrophages and CD301- macrophages was validated by qPCR shown in Appendix Fig S3C. The signaling which were enriched in CD301+ macrophages of the patients compared to controls were supplemented in Appendix Fig S3D.

Figure 2:

1) Are there more CD301+ macrophages near SMA dense areas? CD301+ macrophages are frequently observed throughout many tissues, so their presence near SMA regions is not surprising. It would be noteworthy if their numbers were increased in fibrotic regions but SMA- regions are not shown and no quantification was performed to substantiate the claim.

Response to point 1: Yes, there are more CD301+ macrophages near SMA dense areas and SMA-negative regions almost do not express CD301 especially in control samples, shown in Fig 2C. Quantification was supplemented in the revised figure.

2) The title is not substantiated by the data presented in the figure. To determine a profibrotic phenotype, more than just collagen I should be examined. Especially considering the change in SMA is modest (though statistically significant).

Response to point 2: We apologized for the inappropriate title and has changed it. We added the experiments to probe two other acknowledged markers, fibronectin and CTGF, by western blotting to determine the profibrotic phenotype. Both results were consistent with the results of collagen 1 (Fig 2G).

Figure 3:

1) The in vitro GAS6/AXL findings are quite exciting; however, the majority of GAS6/AXL signaling in vivo may be between immune cells. While CD10 allows pathologists to distinguish the stromal and epithelial fraction of endometrial neoplasms, it is not used to determine fibroblast-like cells compared to immune cells in the stromal fraction of tumors. Why didn't the authors perform SMA and AXL staining? At the very least, it should be demonstrated that the CD10 staining is largely restricted to non-immune cells (CD45-).

Response to point 1: Thanks much for pointing these out. We replaced with SMA and AXL staining in Fig 3E.

2) There are concerns regarding the AXL antibody used and its suitability for immunostaining cells and tissue sections. The majority of AXL staining does not colocalize with SMA+ cells in Figure S4G. In fact, most of the staining does not colocalize with any DAPI+ nuclei.

Response to point 2: We appreciate for your concern. In cell, AXL is located on membrane; α-SMA is located on cytoplasm and DAPI stains nuclei, so the immunostaining results show that they seem to be not co-stained, but they colocalized in the same cell.

Figure 5:

1) The curettage model is an acute injury model that the authors do not show has adhesions. Are adhesions observed in tile scanned or more zoomed out fields of view from this injury model? To confirm that the curettage model is similar to the human IUA model and that this time point is appropriate a more thorough analysis of the immune cell composition is needed in Figure 5D, especially given the dramatic reduction in the relative abundance of macrophages compared to other CD45+ immune cells. More detailed characterization should be compared to immune cell composition in Figure 1.

Response to point 1: Thanks for your comment. We have tried several model schemes to simulate the human IUA situation and finally chose this scheme shown in our manuscript which was relatively superior for simulation. The results of Fig 5 showed this model could induce endometrial fibrosis, increased expression of Collagen 1, GAS6 and AXL and changes of macrophage and its subclusters which are similar to the pathologic alterations in the endometrium of IUA patients compared to controls but do not show adhesion. This mouse model has been widely used in the previous IUA studies (PMID: 35196191, 34132637, 26384164, 24819371).

We added the flow cytometry results of other cells in the mouse model compared to immune cell composition in Fig 1M and Fig 2A. In revised Fig 5D, CD86 which was mostly used for labeling M1-like macrophage in mouse was increased and CD163, usually labeling M2-like macrophage was decreased. These results have the consistent tendency with human's data in Fig 1M and Fig 2A.

Referee #3 (Remarks for Author):

This manuscript by Lv et al provides new insights into the role of CD310+ macrophages in regulating endometrial fibrosis. Targeting CD301+ macrophages revealed that fibrotic phenotype could be modulated leading to improved pregnancy outcome. The studies are well designed by incorporating single cell RNA seq RNA approach, flow cytometry and immunohistology on IUA patient and control samples. Mechanistic studies were conducted in primary endometrial stromal cells. Finally, they conducted interventional studies by targeting CD301+ macrophages in C57Bl6 mouse model. In general, the manuscript is well written. Experiments are well designed with proper controls. Results support the conclusion. This manuscript provides new insights into the complexity of immune associated fibrosis and its overall impact on pregnancy outcome. Some clarifications are required as below.

- They should provide clarity on proliferating macrophage subtype as mentioned in the single cell RNA seq data in Fig 1 J. Does that mean the other subtypes did not proliferate? This is relevant as macrophage proliferation is low at steady state.

Response: Thanks for the comment. Proliferating macrophage was identified because they expressed high-level of proliferation marker, MKI67, CCNB1, PCNA with low expression of macrophage polarization markers, like CD163, MRC1 (CD206), CLEC10A (CD301) shown in Appendix Fig S3A. As indicated by the reviewer, other subtypes expressed low-level of proliferation markers, suggesting that most of macrophages were low proliferative and at steady state.

• Clarify the significance of lower numbers of CD163+ and CD24+ macrophages (Fig 1M) in IUA patients?

Response: CD163+ endometrial macrophages played an important role in host defense and the regulation of tissue homeostasis including tissue breakdown, clearance, and angiogenic remodeling (PMID: 22882270). CD24 interacted with Siglec-10 on innate immune cells to dampen damaging inflammatory responses to infection (PMID: 31367043). CD24+ macrophage also expressed high-level of GATA6 shown in Appendix Fig S3A which was reported to have a phenotype for reparative immune response and anti-fibrotic functions (PMID: 31315031). The lower number of CD163+ and CD24+ macrophage may aggravate the disorder of endometrial microenvironment in IUA patients. We have supplemented the description in Result in the revised manuscript.

• They have very clearly articulated sample numbers for each experiment as well as patient demographic in Supplemental table 1. They should clarify parity information if available for the patients included in the study.

Response: Thanks for the comment. We have added parity information in Appendix Table S1.

• Some caution should be exercised in linking pathogenic role of CD301+ macrophages as outcome of single cell RNA seq is observational.

Response: We apologized for the inappropriate description. We have cautiously revised our text to ensure the statement to be explicit and clear.

• In Fig 1F, how did they define proliferating cell subset and was it unique within the cluster?

Response: We defined it as proliferating cell subset because they expressed high level of MKI67 which was a classic marker for proliferation. In all endometrial cells, as shown in Fig 1A and Appendix Fig S1B, we first identified cell types, including myofibroblast-like cell using their acknowledged markers (Appendix Fig S1B) and then we captured the myofibroblast-like cell and subclustered them into several subsets and identified proliferating cell within it (Fig 1F), so this proliferating cell in Fig 1F should be unique within myofibroblast-like cell.

• What is the significance of lower number of CD14+ cells in IUA patients?

Response: The lower number of CD14+ cells might contribute to the insufficient proliferation and growth of endometrial stromal cells which we documented previously (PMID: 35169075). Consistently, the decreased number of proliferating stromal cells were also observed in IUA patients shown in Appendix Fig S2C. We added the description in Result in revised manuscript.

• Representative images in Fig 2B (colocalization of CD68 with CD301) does not really capture significantly different numbers of CD301 as shown in other figures.

Response: We appreciate for your concern. We have replaced the images to show the representative and consistent results in Fig 2B.

• Fig 3B: Clarify the rational for selecting those specific genes for Q-RT PCR and what do they mean by genes were upregulated.

Response: The genes for Q-RT PCR in Fig 3B were selected from RNA-seq in Fig 3A on the basis that they encoded secretory proteins and conformed to fragments per kilobase per million (FPKM)>100 in CD301+ macrophage, at least a factor of 1.5 and false discovery rate of <0.0001. We chose secretory proteins because we would like to see the communication from CD301+ macrophage to stroma niches and secretory proteins secreted from CD301+ macrophage had more possibility to act on other cells. We set the threshold of genes that had fragments per kilobase per million (FPKM)>100 in CD301+ macrophage, at least a factor of 1.5 and false discovery rate of <0.0001 in order to efficiently select genes that had high and unique expression in CD301+ macrophage compared to other cells.

We apologized for not describing the Q-RT PCR results clearly. We added the black line in Fig 3B. Black lines indicated expression levels in CD301- macrophage which had been normalized to 1, and the bar level indicated fold upregulated changes of gene expression levels in CD301+ macrophage compared to CD301- macrophage. We would like to validate genes selected from RNA-seq that highly expressed in CD301+ macrophage compared to CD301- macrophage by Q-RT PCR.

6th Jul 2023

Dear Dr. Hu,

Thank you for the submission of your revised manuscript to EMBO Molecular Medicine. I am pleased to inform you that we will be able to accept your manuscript pending the following final amendments:

1) Please address all the minor suggestions raised by both referees. Referee #1 point #1 about the title seems to be a confusion as Referee #2 suggested a change of the title for the Figure 1 and not change of the manuscript title. Please explain this in your response and maintain the changed title for the Figure 1. Referee #2 suggests citing of 2 manuscripts, please consider it only if appropriate.
2) Title: Please correct "macrophage" to "macrophages".
3) Please make sure to pay particular attention to the grammar and syntax and I would recommend running the article by a native English speaker.
4) In the main manuscript file, please do the following:
- Correct/answer the track changes suggested by our data editors by working from the attached document.
- Remove font color.
- Please make sure thar figure callouts are in a sequential order. Currently Fig 2B is called out before Fig 2A, please correct.
- In M&M, add statistical paragraph that should reflect all information that you have filled in the Authors Checklist, especially regarding randomization, blinding, replication.
- In M&M, please include statement that the informed consent was obtained from all human subjects and that the experiments conformed to the principles set out in the WMA Declaration of Helsinki and the Department of Health and Human Services Belmont Report.
- Please rename "Conflict of Interest" to "Disclosure Statement & Competing Interests". We updated our journal's competing interests policy in January 2022 and request authors to consider both actual and perceived competing interests. Please review the policy https://www.embopress.org/competing-interests and update your competing interests if necessary.
- Author contributions: Please remove it from the manuscript and specify author contributions in our submission system. CRediT has replaced the traditional author contributions section because it offers a systematic machine-readable author contributions format that allows for more effective research assessment. You are encouraged to use the free text boxes beneath each contributing author's name to add specific details on the author's contribution. More information is available in our guide to authors:
https://www.embopress.org/page/journal/17574684/authorguide#authorshipguidelines
- Data availability: Please use the following format to report the accession number of your data:

The datasets produced in this study are available in the following databases:
[data type]: [full name of the resource] [accession number/identifier] ([doi or URL or identifiers.org/DATABASE:ACCESSION])

Please check "Author Guidelines" for more information.
https://www.embopress.org/page/journal/17574684/authorguide#availabilityofpublishedmaterial
- Correct the reference citation in the reference list. Where there are more than 10 authors on a paper, 10 will be listed, followed by "et al.". Please check "Author Guidelines" for more information.
https://www.embopress.org/page/journal/17574684/authorguide#referencesformat
5) Funding: Please make sure that information about all sources of funding are complete in both our submission system and in the manuscript. Currently National Key R&D Program of China (2021YFC2701603) and Jiangsu Biobank of Clinical Resources (BM2015004) are missing in our submission system.
6) Appendix: Please list all the figures and tables in the table of content and add page numbers.
7) The Paper Explained: I have gone through your text and included some changes (see below). Please review it, amend as you see fit and add it to main manuscript file.
Problem:
Intrauterine adhesion (IUA) is a prevalent condition causing endometrial fibrosis and uterine infertility. However, effective treatment options remain limited due to the high recurrence rate after treatment and a lack of comprehensive understanding regarding its pathogenesis.

Results:
Through single-cell RNA sequencing, we identified and characterized CD301+ macrophages, which play an important role in the progression of IUA. We confirmed an increased presence of CD301+ macrophages in the endometrium of individuals with IUA. This increase in CD301+ macrophages promotes the differentiation of endometrial stromal cells into myofibroblasts, exacerbating endometrial fibrosis. Mechanistically, CD301+ macrophages secrete GAS6, which binds to its receptor AXL, subsequently activating the NF-κB pathway and up-regulating the synthesis of profibrotic proteins. Notably, targeted deletion of CD301+ macrophages or the use of pharmacological inhibitors of AXL effectively prevented endometrial fibrosis and significantly improved pregnancy outcomes in mice.

Impact:
Our findings highlight the therapeutic potential of modulating macrophages to suppress endometrial fibrosis, offering promising avenues for enhancing pregnancy success rates in individuals affected by IUA.
8) Synopsis:
- Synopsis image: Please adjust the size of the synopsis image to 550 px-wide x (250-400)-px high and submit it as a high-resolution jpeg file.
- Synopsis text: I have gone through your text and included some changes (see below). Please review it, amend as you see fit and upload as a separate file.

This study highlights the role of CD301+ macrophages in facilitating endometrial fibrosis in intrauterine adhesion (IUA) through the GAS6/AXL/NF-κB pathway. By depleting CD301+ macrophages or employing the pharmacological inhibitor Bemcentinib to target AXL, the progression of fibrosis can be suppressed, ultimately improving pregnancy outcomes in mice.
• CD301+ macrophages exhibit elevated levels in the endometrial fibrosis of IUA patients.
• CD301+ macrophages secrete GAS6, activating the AXL/NF-κB pathway and promoting fibrosis.
• Depletion of CD301+ macrophages or treatment with the AXL inhibitor Bemcentinib shows promising potential for treating endometrial fibrosis and enhancing pregnancy outcomes.

- Please check your synopsis text and image before submission with your revised manuscript. Please be aware that in the proof stage minor corrections only are allowed (e.g., typos).
9) For more information: This space should be used to list relevant web links for further consultation by our readers. Could you identify some relevant ones and provide such information as well? Some examples are patient associations, relevant databases, OMIM/proteins/genes links, author's websites, etc...
10) As part of the EMBO Publications transparent editorial process initiative (see our Editorial at http://embomolmed.embopress.org/content/2/9/329), EMBO Molecular Medicine will publish online a Review Process File (RPF) to accompany accepted manuscripts. This file will be published in conjunction with your paper and will include the anonymous referee reports, your point-by-point response and all pertinent correspondence relating to the manuscript. Let us know whether you agree with the publication of the RPF and as here, if you want to remove or not any figures from it prior to publication. Please note that the Authors checklist will be published at the end of the RPF.
11) Please provide a point-by-point letter INCLUDING my comments as well as the reviewer's reports and your detailed responses (as Word file).

I look forward to reading a new revised version of your manuscript as soon as possible.

Yours sincerely,

Zeljko Durdevic

Zeljko Durdevic
Editor
EMBO Molecular Medicine

*** Instructions to submit your revised manuscript ***

*** PLEASE NOTE *** As part of the EMBO Publications transparent editorial process initiative (see our Editorial at https://www.embopress.org/doi/pdf/10.1002/emmm.201000094), EMBO Molecular Medicine will publish online a Review Process File to accompany accepted manuscripts.

In the event of acceptance, this file will be published in conjunction with your paper and will include the anonymous referee reports, your point-by-point response and all pertinent correspondence relating to the manuscript. If you do NOT want this file to be published, please inform the editorial office at contact@embomolmed.org.

When submitting your revised manuscript, please include:

1) a .docx formatted version of the manuscript text (including Figure legends and tables)

2) Separate figure files*

3) supplemental information as Expanded View and/or Appendix. Please carefully check the authors guidelines for formatting Expanded view and Appendix figures and tables at
https://www.embopress.org/page/journal/17574684/authorguide#expandedview

4) a letter INCLUDING the reviewer's reports and your detailed responses to their comments (as Word file).

5) The paper explained: EMBO Molecular Medicine articles are accompanied by a summary of the articles to emphasize the major findings in the paper and their medical implications for the non-specialist reader. Please provide a draft summary of your article highlighting
- the medical issue you are addressing,
- the results obtained and
- their clinical impact.
This may be edited to ensure that readers understand the significance and context of the research.
Please refer to any of our published articles for an example.

6) For more information: There is space at the end of each article to list relevant web links for further consultation by our readers. Could you identify some relevant ones and provide such information as well? Some examples are patient associations, relevant databases, OMIM/proteins/genes links, author's websites, etc...

7) Author contributions: the contribution of every author must be detailed in a separate section.

8) EMBO Molecular Medicine now requires a complete author checklist (https://www.embopress.org/page/journal/17574684/authorguide) to be submitted with all revised manuscripts. Please use the checklist as guideline for the sort of information we need WITHIN the manuscript. The checklist should only be filled with page numbers were the information can be found. This is particularly important for animal reporting, antibody dilutions (missing) and exact values and n that should be indicted instead of a range.

9) Every published paper now includes a 'Synopsis' to further enhance discoverability. Synopses are displayed on the journal webpage and are freely accessible to all readers. They include a short stand first (maximum of 300 characters, including space) as well as 2-5 one sentence bullet points that summarise the paper. Please write the bullet points to summarise the key NEW findings. They should be designed to be complementary to the abstract - i.e. not repeat the same text. We encourage inclusion of key acronyms and quantitative information (maximum of 30 words / bullet point). Please use the passive voice. Please attach these in a separate file or send them by email, we will incorporate them accordingly.

You are also welcome to suggest a striking image or visual abstract to illustrate your article. If you do please provide a jpeg file 550 px-wide x 400-px high.

10) A Conflict of Interest statement should be provided in the main text

11) Please note that we now mandate that all corresponding authors list an ORCID digital identifier. This takes <90 seconds to complete. We encourage all authors to supply an ORCID identifier, which will be linked to their name for unambiguous name identification.

Currently, our records indicate that the ORCID for your account is 0000-0001-5475-7840.

Please click the link below to modify this ORCID:
Link Not Available

12) The system will prompt you to fill in your funding and payment information. This will allow Wiley to send you a quote for the article processing charge (APC) in case of acceptance. This quote takes into account any reduction or fee waivers that you may be eligible for. Authors do not need to pay any fees before their manuscript is accepted and transferred to our publisher.

*Additional important information regarding Figures

Each figure should be given in a separate file and should have the following resolution:
Graphs 800-1,200 DPI
Photos 400-800 DPI
Colour (only CMYK) 300-400 DPI"

Figures are not edited by the production team. All lettering should be the same size and style; figure panels should be indicated by capital letters (A, B, C etc). Gridlines are not allowed except for log plots. Figures should be numbered in the order of their appearance in the text with Arabic numerals. Each Figure must have a separate legend and a caption is needed for each panel.

*Additional important information regarding figures and illustrations can be found at https://bit.ly/EMBOPressFigurePreparationGuideline. See also figure legend preparation guidelines: https://www.embopress.org/page/journal/17574684/authorguide#figureformat

The system will prompt you to fill in your funding and payment information. This will allow Wiley to send you a quote for the article processing charge (APC) in case of acceptance. This quote takes into account any reduction or fee waivers that you may be eligible for. Authors do not need to pay any fees before their manuscript is accepted and transferred to our publisher.

***** Reviewer's comments *****

Referee #1 (Comments on Novelty/Model System for Author):

Using endometrium from patients with and without IUA and the mouse model of IUA are strengths of this manuscript.
No ethical issues raised regarding the mouse model.

Referee #1 (Remarks for Author):

I believe this is an important contribution to the medical literature and women's health, underscoring mechanisms involved in IUA, a condition that compromises fertility and can adversely affect pregnancy outcomes. Furthermore, their animal model demonstrates prevention as well as reduction of existing lesions - important to possible clinical translation. In addition, it has broader appeal with regard to inibiting or reducing fibrosis in various settings beyond reproductive.

In response to the reviewers' comments, the authors have provided considerable additional data that have enriched interpretation of the scRNAseq data. Some examples include validation of scRNAseq marker genes by IHC in normal endometrium, mining the # of fibroblast subtypes, updating the analysis (e.g., using CellChat) for interactions between endometrial CD301+ macrophages and myofibroblasts, and demonstrating in the IUA animal model that Bencentinib prevents adhesion formation, addition of additional markers of the profibrotic phenotype by Western blotting. In toto, the authors have responded to the reviewers' comments with a manuscript with congruent findings in the analyses of human tissues and mouse model.

A few comments for the authors to consider:
1. Reviewer 2 suggested a change from the title "Targeting CD301+ macrophage inhibits endometrial fibrosis and improves pregnancy outcome", as this was not reflective of the findings. The authors indicated they changed the title to "Single cell RNA-seq cartography of myofibroblast-like cell and macrophage in human endometrium between IUA patients and healthy controls". However, this has not been done on the revised manuscript or in the supplementary materials. This reviewer believes that this new title does not do justice to the messaging of the manuscript and suggests reverting to the original title.
2. In Figure S1, was the number of samples analyzed for which this is presumed representative?
3. Appendix Table S4 - "Tines for D&C" was clarified by the authors in their response to the reviewers' comments, to mean the # of D&Cs. This should be corrected in Table S4 and the "times for D&C" is ambiguous in its meaning - more common is # of D&Cs or # transcervical procedures - in that Table.
4. The issue of increased CD301+ macrophages in IUA endometrium in human samples - was this replicated in the animal model?

Referee #2 (Comments on Novelty/Model System for Author):

No ethical issues with the model system. A perfect model system does not exist to mimic the human disease.

Referee #2 (Remarks for Author):

The authors have substantially modified their original manuscript. Some editing will be necessary to correct errors during the proof stage, but there are no egregious issues that warrant serious concern. The authors should include details in the methods to describe how CellChat was utilized.

It is interesting that the authors predict IGF signaling may occur between CD301+ macrophages and fibroblasts. Since this was explored in Shook et al., 2018 (PMID 30467144), the manuscript should be integrated into the discussion of the finding. Additionally, the same lab published a manuscript describing the role of CD301+ macrophages in regulating tissue repair/fibrosis

that should be referenced in this manuscript (PMID 27287183). Otherwise, the authors have addressed my initial concerns sufficiently.

***** Reviewer's comments *****

Referee #1 (Remarks for Author):

I believe this is an important contribution to the medical literature and women's health, underscoring mechanisms involved in IUA, a condition that compromises fertility and can adversely affect pregnancy outcomes. Furthermore, their animal model demonstrates prevention as well as reduction of existing lesions - important to possible clinical translation. In addition, it has broader appeal with regard to inhibiting or reducing fibrosis in various settings beyond reproductive.

In response to the reviewers' comments, the authors have provided considerable additional data that have enriched interpretation of the scRNAseq data. Some examples include validation of scRNAseq marker genes by IHC in normal endometrium, mining the # of fibroblast subtypes, updating the analysis (e.g., using CellChat) for interactions between endometrial CD301+ macrophages and myofibroblasts, and demonstrating in the IUA animal model that Bencentinib prevents adhesion formation, addition of additional markers of the profibrotic phenotype by Western blotting. In total, the authors have responded to the reviewers' comments with a manuscript with congruent findings in the analyses of human tissues and mouse model.
Thank you.

A few comments for the authors to consider:

1. Reviewer 2 suggested a change from the title "Targeting CD301+ macrophage inhibits endometrial fibrosis and improves pregnancy outcome", as this was not reflective of the findings. The authors indicated they changed the title to "Single cell RNA-seq cartography of myofibroblast-like cell and macrophage in human endometrium between IUA patients and healthy controls". However, this has not been done on the revised manuscript or in the supplementary materials. This reviewer believes that this new title does not do justice to the messaging of the manuscript and suggests reverting to the original title.

Response to point 1: We appreciate the reviewer's concern. Reviewer #2 suggested a change of the title for the Figure 1 and not change of the manuscript title. So in our final manuscript, the changed title for Figure 1 and the original manuscript title will be maintained.

2. In Figure S1, was the number of samples analyzed for which this is presumed representative?

Response to point 2: Yes, it was representative. We selected the human samples strictly according to the criteria described in M&M and the results from scRNA-seq data has also been validated by the following experiments.

3. Appendix Table S4 - "Times for D&C" was clarified by the authors in their response to the reviewers' comments, to mean the # of D&Cs. This should be corrected in Table S4 and the "times for D&C" is ambiguous in its meaning - more common is # of D&Cs or # transcervical procedures - in that Table.

Response to point 3: Thanks for the suggestion. However, we may maintain Times rather than #, because # may also have different meanings.

4. The issue of increased CD301+ macrophages in IUA endometrium in human samples - was this replicated in the animal model?

Response to point 4: Yes, it was replicated in the animal model. As shown in Figure 5D-E, CD301+ macrophages in the uterus of the IUA mouse model were increased examined by both flow cytometry and immunofluorescence which was consistent with human's data.

Referee #2 (Comments on Novelty/Model System for Author):

No ethical issues with the model system. A perfect model system does not exist to mimic the human disease.

Thank you.

Referee #2 (Remarks for Author):

The authors have substantially modified their original manuscript. Some editing will be necessary to correct errors during the proof stage, but there are no egregious issues that warrant serious concern. The authors should include details in the methods to describe how CellChat was utilized.

Response: Thanks much for pointing these out. We have carefully proof-read the manuscript to minimize typographical, grammatical and syntax errors. The description of CellChat has been added in the method of the revised manuscript.

It is interesting that the authors predict IGF signaling may occur between CD301+ macrophages and fibroblasts. Since this was explored in Shook et al., 2018 (PMID 30467144), the manuscript should be integrated into the discussion of the finding. Additionally, the same lab published a manuscript describing the role of CD301+ macrophages in regulating tissue repair/fibrosis that should be referenced in this manuscript (PMID 27287183). Otherwise, the authors have addressed my initial concerns sufficiently.

Response: Thanks for the valuable suggestion. We have integrated the finding of IGF signaling from both our work and Shook et al.'s work (PMID 30467144) into the discussion of the revised manuscript. We agreed that this manuscript, PMID 27287183, was highly related to our work so we added it into the discussion of the revised manuscript.

Yours sincerely,

Yali Hu

Chief and Professor
Department of Obstetrics and Gynecology
Nanjing Drum Tower Hospital
321 Zhongshan Road
Nanjing 210008, China
E-mail: yalihu@nju.edu.cn

18th Jul 2023

Dear Dr. Hu,

We are pleased to inform you that your manuscript is accepted for publication and is now being sent to our publisher to be included in the next available issue of EMBO Molecular Medicine.

Please read below for additional IMPORTANT information regarding your article, its publication and the production process.

Congratulations on your interesting work,

Zeljko Durdevic

Zeljko Durdevic
Editor
EMBO Molecular Medicine

Follow us on Twitter @EmboMolMed
Sign up for eTOCs at embopress.org/alertsfeeds

*** *** *** IMPORTANT INFORMATION *** *** ***

SPEED OF PUBLICATION
The journal aims for rapid publication of papers, using using the advance online publication "Early View" to expedite the process: A properly copy-edited and formatted version will be published as "Early View" after the proofs have been corrected. Please help the Editors and publisher avoid delays by providing e-mail address(es), telephone and fax numbers at which author(s) can be contacted.

Should you be planning a Press Release on your article, please get in contact with embomolmed@wiley.com as early as possible, in order to coordinate publication and release dates.

LICENSE AND PAYMENT:

All articles published in EMBO Molecular Medicine are fully open access: immediately and freely available to read, download and share.

EMBO Molecular Medicine charges an article processing charge (APC) to cover the publication costs. You, as the corresponding author for this manuscript, should have already received a quote with the article processing fee separately. Please let us know in case this quote has not been received.

Once your article is at Wiley for editorial production you will receive an email from Wiley's Author Services system, which will ask you to log in and will present you with the publication license form for completion. Within the same system the publication fee can be paid by credit card, an invoice, pro forma invoice or purchase order can be requested.

Payment of the publication charge and the signed Open Access Agreement form must be received before the article can be published online.

PROOFS

You will receive the proofs by e-mail approximately 2 weeks after all relevant files have been sent o our Production Office. Please return them within 48 hours and if there should be any problems, please contact the production office at embopressproduction@wiley.com.

Please inform us if there is likely to be any difficulty in reaching you at the above address at that time. Failure to meet our

deadlines may result in a delay of publication.

All further communications concerning your paper proofs should quote reference number EMM-2023-17601-V3 and be directed to the production office at embopressproduction@wiley.com.

Thank you,

Zeljko Durdevic
Editor
EMBO Molecular Medicine

EMBO Press Author Checklist

Corresponding Author Name: Yali Hu, Guangfeng Zhao	
Journal Submitted to: EMBO Molecular Medicine	
Manuscript Number: EMM-2023-17601	

USEFUL LINKS FOR COMPLETING THIS FORM
The EMBO Journal - Author Guidelines
EMBO Reports - Author Guidelines
Molecular Systems Biology - Author Guidelines
EMBO Molecular Medicine - Author Guidelines

Reporting Checklist for Life Science Articles (updated January
This checklist is adapted from Materials Design Analysis Reporting (MDAR) Checklist for Authors. MDAR establishes a minimum set of requirements in transparent reporting in the life sciences (see Statement of Task: 10.31222/osf.io/9sm4x). Please follow the journal's guidelines in preparing your
Please note that a copy of this checklist will be published alongside your article.

Abridged guidelines for figures
1. Data
The data shown in figures should satisfy the following conditions:
- the data were obtained and processed according to the field's best practice and are presented to reflect the results of the experiments in an accurate and unbiased manner.
- ideally, figure panels should include only measurements that are directly comparable to each other and obtained with the same assay.
- plots include clearly labeled error bars for independent experiments and sample sizes. Unless justified, error bars should not be shown for technical
- if n<5, the individual data points from each experiment should be plotted. Any statistical test employed should be justified.
- Source Data should be included to report the data underlying figures according to the guidelines set out in the authorship guidelines on Data

2. Captions
Each figure caption should contain the following information, for each panel where they are relevant:
- a specification of the experimental system investigated (eg cell line, species name).
- the assay(s) and method(s) used to carry out the reported observations and measurements.
- an explicit mention of the biological and chemical entity(ies) that are being measured.
- an explicit mention of the biological and chemical entity(ies) that are altered/varied/perturbed in a controlled manner.
- the exact sample size (n) for each experimental group/condition, given as a number, not a range;
- a description of the sample collection allowing the reader to understand whether the samples represent technical or biological replicates (including how many animals, litters, cultures, etc.).
- a statement of how many times the experiment shown was independently replicated in the laboratory.
- definitions of statistical methods and measures:
  - common tests, such as t-test (please specify whether paired vs. unpaired), simple χ2 tests, Wilcoxon and Mann-Whitney tests, can be unambiguously identified by name only, but more complex techniques should be described in the methods section;
  - are tests one-sided or two-sided?
  - are there adjustments for multiple comparisons?
  - exact statistical test results, e.g., P values = x but not P values < x;
  - definition of 'center values' as median or average;
  - definition of error bars as s.d. or s.e.m.

Please complete ALL of the questions below.
Select "Not Applicable" only when the requested information is not relevant for your study.

Materials

Newly Created Materials	Information included in the manuscript?	In which section is the information available? (Reagents and Tools Table, Materials and Methods, Figures, Data Availability Section)
New materials and reagents need to be available; do any restrictions apply?	Not Applicable	

Antibodies	Information included in the manuscript?	In which section is the information available? (Reagents and Tools Table, Materials and Methods, Figures, Data Availability Section)
For **antibodies** provide the following information:<br>- Commercial antibodies: RRID (if possible) or supplier name, catalogue number and or/clone number<br>- Non-commercial: RRID or citation	Yes	Appendix Table S6

DNA and RNA sequences	Information included in the manuscript?	In which section is the information available? (Reagents and Tools Table, Materials and Methods, Figures, Data Availability Section)
**Short novel DNA or RNA including primers, probes**: provide the sequences.	Yes	Appendix Table S5

Cell materials	Information included in the manuscript?	In which section is the information available? (Reagents and Tools Table, Materials and Methods, Figures, Data Availability Section)
**Cell lines:** Provide species information, strain. Provide accession number in repository **OR** supplier name, catalog number, clone number, and/**OR** RRID.	Not Applicable	
**Primary cultures:** Provide species, strain, sex of origin, genetic modification status.	Yes	Materials and Methods
Report if the cell lines were recently **authenticated** (e.g., by STR profiling) and tested for mycoplasma contamination.	Not Applicable	

Experimental animals	Information included in the manuscript?	In which section is the information available? (Reagents and Tools Table, Materials and Methods, Figures, Data Availability Section)
**Laboratory animals or Model organisms:** Provide species, strain, sex, age, genetic modification status. Provide accession number in repository **OR** supplier name, catalog number, clone number, **OR** RRID.	Yes	Materials and Methods
**Animal observed in or captured from the field:** Provide species, sex, and age where possible**.**	Not Applicable	
Please detail **housing and husbandry conditions**.	Yes	Materials and Methods

Plants and microbes	Information included in the manuscript?	In which section is the information available? (Reagents and Tools Table, Materials and Methods, Figures, Data Availability Section)
**Plants:** provide species and strain, ecotype and cultivar where relevant, unique accession number if available, and source (including location for collected wild specimens).	Not Applicable	
**Microbes:** provide species and strain, unique accession number if available, and source.	Not Applicable	

Human research participants	Information included in the manuscript?	In which section is the information available? (Reagents and Tools Table, Materials and Methods, Figures, Data Availability Section)
If collected and within the bounds of privacy constraints report on age, sex and gender or ethnicity for all study participants.	Yes	Materials and Methods

Core facilities	Information included in the manuscript?	In which section is the information available? (Reagents and Tools Table, Materials and Methods, Figures, Data Availability Section)
If your work benefited from core facilities, was their service mentioned in the acknowledgments section?	Not Applicable	

Design

Study protocol	Information included in the manuscript?	In which section is the information available? (Reagents and Tools Table, Materials and Methods, Figures, Data Availability Section)
If study protocol has been **pre-registered, provide DOI in the manuscript**. For clinical trials, provide the trial registration number **OR** cite DOI.	Not Applicable	
Report the **clinical trial registration number** (at ClinicalTrials.gov or equivalent), where applicable.	Not Applicable	

Laboratory protocol	Information included in the manuscript?	In which section is the information available? (Reagents and Tools Table, Materials and Methods, Figures, Data Availability Section)
Provide DOI OR other citation details if **external detailed step-by-step protocols** are available.	Not Applicable	

Experimental study design and statistics	Information included in the manuscript?	In which section is the information available? (Reagents and Tools Table, Materials and Methods, Figures, Data Availability Section)
Include a statement about **sample size** estimate even if no statistical methods were used.	Yes	Materials and Methods and Figure legends
Were any steps taken to minimize the effects of subjective bias when allocating animals/samples to treatment (e.g. **randomization procedure**)? If yes, have they been described?	Not Applicable	
Include a statement about **blinding** even if no blinding was done.	Yes	Materials and Methods
Describe **inclusion/exclusion criteria** if samples or animals were excluded from the analysis. Were the criteria pre-established?<br><br>If sample or data points were omitted from analysis, report if this was due to attrition or intentional exclusion and provide justification.	Not Applicable	
For every figure, are **statistical tests** justified as appropriate? Do the data meet the assumptions of the tests (e.g., normal distribution)? Describe any methods used to assess it. Is there an estimate of variation within each group of data? Is the variance similar between the groups that are being statistically compared?	Yes	Materials and Methods and Figure legends

Sample definition and in-laboratory replication	Information included in the manuscript?	In which section is the information available? (Reagents and Tools Table, Materials and Methods, Figures, Data Availability Section)
In the figure legends: state number of times the experiment was **replicated** in laboratory.	Yes	Figure legends
In the figure legends: define whether data describe **technical or biological replicates**.	Yes	Figure legends

Ethics

Ethics	Information included in the manuscript?	In which section is the information available? (Reagents and Tools Table, Materials and Methods, Figures, Data Availability Section)
Studies involving **human participants**: State details of **authority granting ethics approval** (IRB or equivalent committee(s), provide reference number for approval.	Yes	Materials and Methods
Studies involving **human participants**: Include a statement confirming that **informed consent** was obtained from all subjects and that the experiments conformed to the principles set out in the WMA Declaration of Helsinki and the Department of Health and Human Services Belmont Report.	Yes	Materials and Methods
Studies involving **human participants:** For publication of **patient photos**, include a statement confirming that consent to publish was obtained.	Not Applicable	
Studies involving experimental **animals**: State details of **authority granting ethics approval** (IRB or equivalent committee(s), provide reference number for approval. Include a statement of compliance with ethical regulations.	Yes	Materials and Methods
Studies involving **specimen and field samples:** State if relevant **permits** obtained, provide details of authority approving study; if none were required, explain why.	Yes	Materials and Methods

Dual Use Research of Concern (DURC)	Information included in the manuscript?	In which section is the information available? (Reagents and Tools Table, Materials and Methods, Figures, Data Availability Section)
Could your study fall under dual use research restrictions? Please check biosecurity documents and list of **select agents and toxins** (CDC): https://www.selectagents.gov/sat/list.htm	Not Applicable	
If you used a select agent, is the security level of the lab appropriate and reported in the manuscript?	Not Applicable	
If a study is subject to dual use research of concern regulations, is the name of the **authority granting approval and reference number** for the regulatory approval provided in the manuscript?	Not Applicable	

Reporting

The MDAR framework recommends adoption of discipline-specific guidelines, established and endorsed through community initiatives. Journals have their own policy about requiring specific guidelines and recommendations to complement MDAR.

Adherence to community standards	Information included in the manuscript?	In which section is the information available? (Reagents and Tools Table, Materials and Methods, Figures, Data Availability Section)
State if relevant guidelines or checklists (e.g., **ICMJE, MIBBI, ARRIVE, PRISMA**) have been followed or provided.	Not Applicable	
For **tumor marker prognostic studies,** we recommend that you follow the **REMARK** reporting guidelines (see link list at top right). See author guidelines, under 'Reporting Guidelines'. Please confirm you have followed these guidelines.	Not Applicable	
For **phase II and III randomized controlled trials**, please refer to the **CONSORT** flow diagram (see link list at top right) and submit the CONSORT checklist (see link list at top right) with your submission. See author guidelines, under 'Reporting Guidelines'. Please confirm you have submitted this list.	Not Applicable	

Data Availability

Data availability	Information included in the manuscript?	In which section is the information available? (Reagents and Tools Table, Materials and Methods, Figures, Data Availability Section)
Have **primary datasets** been deposited according to the journal's guidelines (see 'Data Deposition' section) and the respective accession numbers provided in the Data Availability Section?	Yes	Data availability
Were **human clinical and genomic datasets** deposited in a public access-controlled repository in accordance to ethical obligations to the patients and to the applicable consent agreement?	Yes	Data availability
Are **computational models** that are central and integral to a study available without restrictions in a machine-readable form? Were the relevant accession numbers or links provided?	Not Applicable	
If publicly available data were reused, provide the respective **data citations in the reference list**.	Yes	Materials and Methods and Reference
